# Case report of a Li–Fraumeni syndrome-like phenotype with a de novo mutation in *CHEK2*

**DOI:** 10.1097/MD.0000000000004251

**Published:** 2016-07-22

**Authors:** Xuehan Zhuang, Yongping Li, Hongzhi Cao, Ting Wang, Jianghao Chen, Jiayun Liu, Liya Lin, Rui Ye, Xinyang Li, Shuang Liu, Weiyang Li, Yonggang Lv, Juliang Zhang, Chenyang He, Xun Xu, Zhen Wang, Chen Huang, Xiao Liu, Ling Wang

**Affiliations:** a BGI-Shenzhen, Shenzhen; b Department of Vascular and Endocrine Surgery, Xijing Hospital, Fourth Military Medical University, Xi’an, Shaanxi, China; c Department of Biology, University of Copenhagen, Copenhagen, Denmark; d Institute of Clinical Laboratory Medicine; e Department of Orthopedic; f Department of Nephrology, Xijing Hospital, Fourth Military Medical University, Xi’an, Shaanxi, China.

**Keywords:** *CHEK2*, de novo mutation, Li–Fraumeni syndrome-like, whole exome sequencing

## Abstract

**Background:**

Cases of multiple tumors are rarely reported in China. In our study, a 57-year-old female patient had concurrent squamous cell carcinoma, mucoepidermoid carcinoma, brain cancer, bone cancer, and thyroid cancer, which has rarely been reported to date.

**Methods:**

To determine the relationship among these multiple cancers, available DNA samples from the thyroid, lung, and skin tumors and from normal thyroid tissue were sequenced using whole exome sequencing.

**Results:**

The notable discrepancies of somatic mutations among the 3 tumor tissues indicated that they arose independently, rather than metastasizing from 1 tumor. A novel deleterious germline mutation (chr22:29091846, G->A, p.H371Y) was identified in *CHEK2*, a Li–Fraumeni syndrome causal gene. Examining the status of this novel mutation in the patient's healthy siblings revealed its de novo origin.

**Conclusion:**

Our study reports the first case of Li–Fraumeni syndrome-like in Chinese patients and demonstrates the important contribution of de novo mutations in this type of rare disease.

## Abstract 2

Supplemental Digital Content is available in the text

## 1 Introduction

Currently, researchers’ understanding of the pathogenic and developmental mechanisms of cancer is improving.^[1]^ The detection and treatment of cancer is becoming increasingly precise. The overall cure rate and survival associated with cancer are significantly improved compared with those in the past.^[2,3]^ However, it is undeniable that the vast majority of cancer treatments only slow the progress of cancer, and most cancer patients eventually die from recurrence or metastasis. Given this situation, we cannot refrain from thinking that all of these “failures” may be not only due to poor treatment or prognosis but also due to the incomplete understanding of cancer.

There have been many studies regarding metastatic carcinomas, such as brain metastasis from breast cancer,^[4]^ lymph node metastasis from a primary acral melanoma,^[5]^ and lung metastases from hepatocellular carcinoma,^[6]^ and the high similarity of the mutation spectrum and the large frequency of the shared driver mutations between primary and metastatic cancers are the major genomic features. In contrast, multiple primary carcinoma, which could arise in the same organ or various organs, has rarely been extensively studied because of its low incidence and high complexity. The most studied multiple primary carcinoma is Li–Fraumeni syndrome (LFS), which is a rare disorder that greatly increases the risk of developing several types of cancer, such as breast cancer, osteosarcoma, and brain tumors. Identifying and understanding the origin of the causal mutation in each affected family could provide valuable guidance for disease diagnosis and prevention.

To date, over half of the cases have been ascribed to germline mutations of the *TP53* tumor suppressor gene, which encodes a transcription factor (p53) that normally regulates the cell cycle and prevents genomic mutations.^[7–9]^ Sequencing the DNA of several families with LFS showed an autosomal dominant inheritance of the mutated *TP53* gene.^[9]^ Meanwhile, the frequency of new (de novo) *TP53* mutations is estimated to be at least 7% and may be as high as 20%.^[10]^ Another gene associated with LFS is *CHEK2*, which remains somewhat controversial. The potential correlation between *CHEK2* and LFS was first described by Bell et al, in 1999. After that, although the possibility of *CHEK2*'s contribution to LFS and Li–Fraumeni syndrome-like (LFS-L) has been inferred by many researchers, there was no evidence indicating *CHEK2* is a major gene involved in LFS and LFS-L.^[11–20]^ To date, 4 mutations in *CHEK2* have been reported to be associated with LFS or LFS-L: c.1100delC frameshift mutation, c.470T>C nonsynonymous mutation, c.1422delT frameshift deletion, and c.983T>C nonsynonymous mutation,^[11,14,15,20]^ which confirms the relationship between *CHEK2* and LFS. De novo original causal mutations in *TP53* have been revealed. However, a causal mutation in *CHEK2* has not been reported in patients with LFS-L.

Spontaneous germline mutation, which occurs due to an error in copying genetic material or an error in cell division, is estimated to occur at 1.18 × 10^−8^ per position and 1.5 in the exonic region of the human genome, on average. It plays an important role in human diseases, and highly penetrant alleles are under strong negative selection. Such alleles can frequently be observed as de novo mutations (DNMs) in affected individuals who harbor neurodevelopmental disorders.^[21,22]^

LFS-L are considered rare, and, at present, they have been reported in approximately 500 families, none of which are in China.^[23]^ Very few studies have utilized next-generation sequencing (NGS) to obtain genetic profiles of the multiple tumors to pinpoint pathogenesis.^[24]^ In our study, a 57-year-old female patient had concurrent squamous cell carcinoma (SCC), mucoepidermoid carcinoma, brain cancer, bone cancer, and thyroid cancer, which has seldom been reported to date. To verify the relationship among these cancers and identify the mutations, we performed an exome-wide genetic investigation on the different cancerous and normal tissues that we could obtain. We found that the somatic mutations of these 3 cancer tissues were notably different, indicating that the multiple carcinomas might originate independently, instead of occurring by metastasis. By systematic examination of the genomic information of this patient and her unaffected siblings, we identified a de novo nonsynonymous mutation in *CHEK2*, the well-known LFS-associated gene. Thus, by combining the clinical picture with the genomic information provided by NGS and the literature, we diagnosed and reported the first case of LFS-L in China to have a *TP53*-negative and *CHEK2*-positive mutation.

## 2 Materials and methods

### 2.1 Library preparation and whole exome sequencing

We used a QIAamp DNA formalin-fixed and paraffin-embedded tissue kit from Qiagen to collect DNA. The DNA was subsequently used for library preparations, exon capturing, and HiSeq2000 sequencing. The library construction of the HiSeq platform included 5 steps: DNA was sheared to a size of 150 to 200 bp and the end repair was performed, after which adenine and the HiSeq adapter were added and the polymerase chain reaction (PCR) reaction was carried out. Finally, the library was ready for hybridization capture. Agilent 44M was the probe for the whole human exon. There was biotin on the probe, which could bind the M-280 beads. Therefore, we could collect the whole exon regions of the DNA. The DNA was amplified using PCR, and then the library was sequenced using HiSeq2000 for type PE91+8+91.

The study was approved by the Ethics Committee of Xijing Hospital, Fourth Military Medical University.

### 2.2 Read mapping and germline variation detection

After removing the reads with sequencing adapters and low-quality reads with >5 ambiguous bases, high-quality reads were aligned to the National Center for Biotechnology Information (NCBI) human reference genome (hg19) using BWA (v0.5.9)^[25]^ with its default parameters. Picard (v1.54) (http://picard.sourceforge.net/) was used to mark duplicates, and the process was followed using the Genome Analysis Toolkit (v1.0.6076, GATK IndelRealigner)^[26]^ to improve the alignment accuracy. We determined the final BAM file, and we used GATK to detect the single nucleotide polymorphism (SNP) with the parameters of -stand_call_conf 50 and -stand_emit_conf 10.0. The short insertions and deletions (Indels) were also detected by GATK with the same parameters. Specific SNPs and Indels were then filtered for if they met any of the following criteria: 1, the altered allele support reads <4; 2, genotype quality <20; 3, significant strand bias (*P* < 0.05, Fisher test). All of the SNPs and Indels were finally annotated with ANNOVAR (released October 2, 2011).^[27]^

### 2.3 Purity estimation and somatic mutation detection

A purity estimation was performed using allele-specific copy number analysis of tumors, through which we could not only accurately dissect the allele-specific copy number of solid tumors but also simultaneously estimate and adjust both tumor ploidy and nonaberrant cell admixture.^[28]^ Somatic point mutations were detected by VarScan2.2.5 (SAMtools [v0.1.18]^[29]^ mpileup –Q 0 && VarScan2.2.5 somatic –min-coverage 10 –min-coverage-normal 10 –min-coverage-tumour 10 –min-var-freq 0.1 –min-avg-qual 0)^[30]^ and MuTect.^[31]^ Somatic Indels were predicted using the GATK Somatic Indel Detector with its default parameters. All of the high-confident mutations were obtained using an in-house pipeline coupled with visual inspection, and they were then annotated with ANNOVAR.

### 2.4 Variant validation

We designed oligos to collect the target regions by PCR for candidate mutation. The PCR products were sequenced using the ABI 3730 platform.

## 3 Results

### 3.1 History and clinical investigation of the case

In October 2011, a 57-year-old female was admitted to our hospital with a 1-month history of pain in the left iliac waist and discomfort in the neck area. After a detailed examination, several nodules were found in the subcutaneous tissue of the trunk of the patient. Among these nodules, the 1 in the left iliac waist was found invading the skin, seeming to be a furuncle (Supplementary Fig. S1). An oval-shaped nodule was found in her right lateral neck by color Doppler ultrasound examination. The 6.0 × 1.6 cm–sized nodule had an ill-defined margin, a heterogeneous hypoechogenicity, and an incomplete capsule.

**Figure 1:**
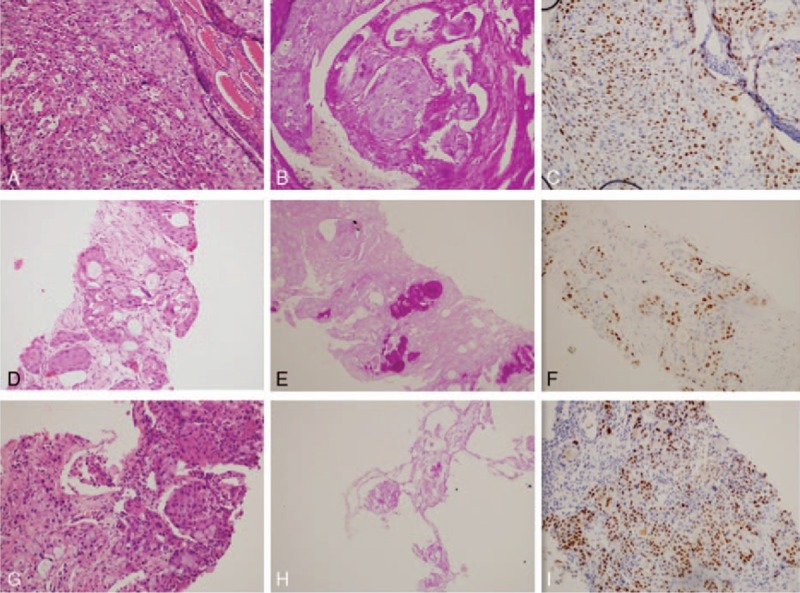
Immunohistochemical staining results of thyroid, skin, and lung tumor samples. (A–C) The thyroid tumor cells in HEX200, which were positive for P63 and PAS; (D–F) immunohistochemical testing of the maculopapular eruption of the left iliac waist skin revealed the same immunohistochemical profile as that of the thyroid lesion; (G–I) the immunohistochemical testing of the lung tumor also revealed the same immunohistochemical profile. PAS, periodic acid-Schiff.

A total thyroidectomy and neck level VI lymph node dissection were performed, and the postoperative pathological examination showed the presence of a poorly differentiated SCC, which was positive for P63 (a tumor suppressor protein) (Fig. 1A and C). Meanwhile, the tumor cells had a scattering of mucus-producing epithelial components, and immunohistochemical staining revealed that the tumor cells were positive for periodic acid-Schiff stain (Fig. 1B). A staging FDG PET/CT scan showed that a lung nodule, the right frontal lobe of the brain, and multiple bones were FDG-avid (Supplementary Figs. S2–S4). In addition, there was a hypermetabolic focus in the subcutaneous tissue of the trunk (Supplementary Fig. S5). A fine-needle aspiration biopsy and immunohistochemical testing on the 5 maculopapular eruptions of left iliac waist and the lung tumor tissue revealed the same immunohistochemical patterns as the thyroid lesion (Fig. 1D–I). In the auxiliary examinations, laboratory investigations indicated a serum carcino-embryonic antigen (CEA) level of 5.4 ng/mL (normal range, 0–5 ng/mL), a serum CA153 level of 54.96 units/mL (normal range, 0–25 units/mL), a serum CA19-9 level of 558.40 units/mL (normal range, 0–29 units/mL), and a serum CA125 level of 407.60 units/mL (normal range, 0–35 units/mL).

Combined treatment could not be performed because of the extent of the predicted radiotherapy field. As the patient refused to be treated with chemotherapy or any other targeted therapies, she received supportive care and was treated with Euthyrox.

The patient's general condition deteriorated soon after, and she died 7 weeks after the total thyroidectomy. No autopsy was performed.

All of this patient's first-degree relatives were healthy.

A case of multiple tumors is rarely reported in China. The existence of concurrent SCC, mucoepidermoid carcinoma, brain cancer, bone cancer, and thyroid cancer has been seldom reported to date. Before the genetic profiling, the clinical diagnosis for this case was likely to be synchronous multiple primary lung cancers, accompanied by brain, skin, and thyroid carcinomas and osteomas. We could not, however, exclude other possibilities, such as primary thyroid carcinoma with metastasis or synchronous primary carcinomas in multiple tissues.

### 3.2 Whole exome sequencing, variant calling, and tumor purity estimation

DNA samples from the thyroid, lung, and skin tumors and from the normal thyroid tissue were sequenced. The average sequencing depth of the 4 tissues was approximately 126.34-fold, and the coverage of the whole exome was 99.5% (Supplementary Tables S1 and S2). We detected approximately 30k SNPs for each tissue with a relatively high dbSNP rate of 98.3%, and the number of Indels was approximately 2.2k with a dbSNP rate of 72% (Supplementary Tables S3 and S4). For a more precise somatic mutation detection excluding the pollution of normal tissues, we estimated the purity of these 3 tumor tissues using the SNP data. The purity of these 3 samples was 87% (lung), 75% (thyroid), and 56% (skin), with a relatively high fit and reliability score (Supplementary Fig. S6).

We eventually detected 511, 1341, and 3247 somatic single nucleotide variants (SNVs) for the thyroid tumor, lung tumor, and skin tumors, respectively, and the mutation spectrum can be observed in Supplementary Fig. S7. The average Ti/Tv was 1.44, and the average NS/SS was approximately 2.23. We determined 40 (4 in exonic), 50 (2 in exonic), and 41 (2 in exonic) somatic Indels for the thyroid, lung, and skin tumors, respectively (Supplementary Tables S10–S12).

### 3.3 The genomic profile of the multiple carcinomas revealed their nonmetastatic nature

**Figure 2:**
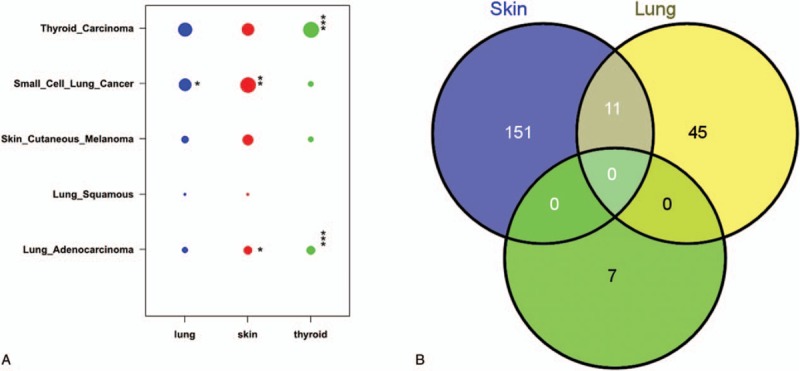
The molecular-based taxonomy and the relationship among the somatic mutations of the 3 tumors. (A) The vertical axis denotes the 5 tumor types and subtypes that we selected from the TCGA database, and the lateral axis indicates the 3 tumor types in our study. The size of the dot indicates the number of genes belonging to a certain tumor type. The Fisher test was performed to investigate the enrichment significance of each dot. The asterisk indicates the *P* value level: (∗) 0.01–0.05, (∗∗) 0.001–0.01, and (∗∗∗) <0.001. (B) The Venn diagram presents the association of the cancer-related somatic mutations among the 3 tumors. Except for 6 mutations shared between the skin and the lung, no mutation was shared by the 3 tumors.

We undertook a molecular taxonomy of these three tumor tissues using the The Cancer Genome Atlas (TCGA) database.^[32]^ Five tumor types that were likely to be associated with our study were selected: thyroid carcinoma, small cell lung cancer, skin cutaneous melanoma, lung squamous, and lung adenocarcinoma. The overlapped mutated genes were filtered. After comparing the tissues from our study to the data we obtained via the above process, it was determined that the lung cancer tissue in our study was potentially linked to small cell lung cancer, as the cancers shared 26 genes with somatic mutations, such as *ABCC2*, *MST1*, and *GRM4*. The thyroid cancer tissue might be associated with both thyroid carcinoma and lung adenocarcinoma by 15 and 8 shared mutated genes, separately. The skin tumor could not be ascribed to skin cutaneous melanoma but was more likely to arise from small cell lung cancer with 65 common mutated genes, such as *CORIN* and *ASPM* (Fig. 2A). This observation indicated that the genomic signatures were shared across tumor tissues, and the genomic profiles were not thoroughly identical to their tissue-of-tumor counterparts.

The relationship of the somatic mutations in these 3 tissues may potentially explain their evolutionary histories. In the thyroid tissue, there were 283 SNVs (269 novel) located in the exonic regions, 214 (205 novel) of which were nonsynonymous mutations, and 7 SNVs were recorded in Catalogue of Somatic Mutations in Cancer (v67_241013) (Supplementary Table S6). In the lung carcinoma tissue, 914 SNVs (724 novel) were in exonic regions; 570 (472 novel) were nonsynonymous, and 56 SNVs were potentially associated with cancer (Supplementary Table S7). In the exonic regions of the skin tumor, there were 2058 SNVs (991 novel), and 1226 (688 novel) were nonsynonymous, while 162 SNVs located within cancer-related genes were found (Supplementary Table S8). The T:A->G:C was the highest mutated type in the spectrum of thyroid tumor SNVs, while the C:G->T:A was the maximum type in the mutation spectrum of both skin and lung tumors (Supplementary Fig. S7). Next, we focused on these cancer-related genes and found that there were no mutations in any of the 3 tumor tissues, but 11 SNVs were shared by the skin tumor and the lung tumor (Fig. 2B; Supplementary Table S9). The tremendous discrepancies of somatic mutations among the 3 tumor tissues in this case suggested that they had arisen from independent tumor origins rather than metastasis.

### 3.4 The de novo mutation in *CHEK2*

To identify the potential pathogenesis of this rare case, we carefully examined the patient's genomic information. In total, we detected 31,840 germline mutations, including 30,022 SNPs and 1818 Indels, among which 17,606 (440 novel) were located in exome regions, and 7915 (259 novel) mutations were nonsynonymous or frameshift mutations. We then focused on the germline mutations in cancer-related functional genes (Supplementary Table S5) and found a novel deleterious heterozygous nonsynonymous mutation (chr22:29091846, G->A, p.H371Y) in *CHEK2*, which is not recorded in the 1000 genome database and dbSNP137. Interestingly, a nonsynonymous 2-bp mutation located nearby was also related to multiple cancers in the clivar database.^[34]^ Previous publications showed that LFS and LFS-L can be ascribed mostly to *TP53* and *CHEK2* mutations. However, we did not find any germline mutations in *TP53*, which means that our case is a *TP53*-negative and *CHEK2*-positive LFS-L. *CHEK2* is a cell cycle checkpoint kinase involved in DNA repair, cell death, and cell cycle control by stabilizing the p53 protein.^[33]^ The mutation in our case was discovered for the first time, and the sorting tolerant from intolerant predicts that this amino acid substitution may have damaging effects on the protein function of *CHEK2*.

The father of the patient passed away prior to the patient without a known clinical history, and the other first- or second-degree relatives of the patient were healthy. To confirm the originality of the identified mutation in *CHEK2*, we performed Sanger sequencing on this locus for the patient and her 2 sisters, whose genomic DNA we obtained.

**Figure 3:**
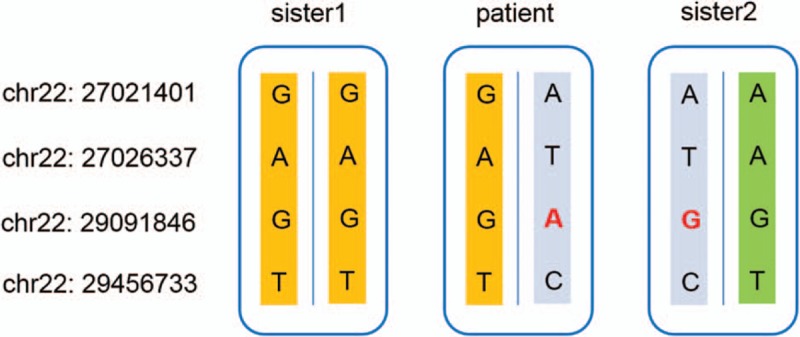
The genotype and haplotype results of the casual mutation in *CHEK2* and 3 nearby positions in our patient's thyroid and normal tissues and the blood of her 2 sisters.

The genotype results validated the heterozygous G->A mutation in the patient and showed that both of her 2 healthy sisters were homozygous G. Moreover, 3 nearby mutations were also genotyped by the Sanger method to determine a parental origin. To our surprise, the phased haplotype results demonstrated that the deleterious nonsynonymous G->A mutations in the studied patients originated from DNM rather than inherited mutations (Fig. 3). Thus, our result revealed that the DNM (p.H371Y) in *CHEK2* was the pathogenic mutation in our patients.

## 4 Discussion

LFS and LFS-L have rarely been extensively studied because of their low incidence and high complexity, and notably few studies have utilized NGS to genetically profile and pinpoint their pathogenesis. Although our case included only 1 patient, for the first time, we used NGS-based method to investigate LFS-L in a Chinese patient. Furthermore, unlike previous publications, we evaluated not only the patient's normal tissue or blood but also the tumor tissues, and from the genomic level demonstrated that the multiple tumors in the patient with LFS-L were not metastatic but had different origins.

We have found a new mutation in *CHEK2*, which sorting tolerant from intolerant suggests to be damaging, and we confirmed the mutation to be de novo, rather than inherited, using the haplotype inferences from the surrounding mutations from samples of family members. This mutation is the first reported DNM in *CHEK2* causing LFS-L in a Chinese patient. The mutation was discovered for the first time because of the difference in the ethnic backgrounds and populations between the samples in our research and in previous studies. The c.1100delC variant in *CHEK2* was reported to be a major LFS susceptibility mutation in the United States but not in Dutch populations. The c.983T>C was found to cause the Dutch mutation but was not found in patients from the USA or Finland.^[11,20]^ Additionally, a germline mutation in chr22:29091848 has been reported to be associated with hereditary cancer-predisposing syndrome, which, to a certain extent, demonstrated that the mutation we found is indeed associated with cancer-related diseases.^[34]^

NGS is showing its capacity to contribute to the molecular taxonomy of cancer based on known mutational profiles, and this could be clinically useful. In our case, each tumor tissue had somatic mutations reported in TCGA, from which we hypothesize that although the mutation in *CHEK2* is the pathogenic mutation in the patients with LFS or LFS-L, finally either the tumor is more associated with the accumulation of somatic mutations in a particular organization or the mutation in *CHEK2* induces chromosomal instability and somatic mutation accumulation.

In summary, although this retrospective analysis did not help our patient due to the rapid and aggressive development of the disease, our discoveries provide an important contribution to future research examining LFS-L in the Chinese population.

## Supplementary Material

Supplemental Digital Content

## Supplementary Material

Supplemental Digital Content
