# Case report of a Li–Fraumeni syndrome-like phenotype with a de novo mutation in *CHEK2*: Erratum

**DOI:** 10.1097/MD.0000000000005665

**Published:** 2016-12-09

In the article “Case report of a Li–Fraumeni syndrome-like phenotype with a de novo mutation in *CHEK2*”,^[1]^ which appeared in Volume 95, Issue 29 of *Medicine*, Dr. Zhuang's affiliation was originally given incorrectly. The correct affiliation is as follows: Department of Vascular and Endocrine Surgery, Xijing Hospital, Fourth Military Medical University, Xi’an, Shaanxi, China.
