# Supplementary material for: Case report of a Li–Fraumeni syndrome-like phenotype with a de novo mutation in CHEK2
Source: Medicine (Baltimore). 2016 Jul 22;95(29):e4251. doi: 10.1097/MD.0000000000004251 (PMC5265769; doi:10.1097/MD.0000000000004251)
Supplement: Supplemental Digital Content [file medi-95-e4251-s001.doc]

**Supplementary Figure S1:** The appearance picture of the skin nodule in left iliac waist. The skin nodule in the left iliac waist was red and inflamed , which seemed to be a furuncle.


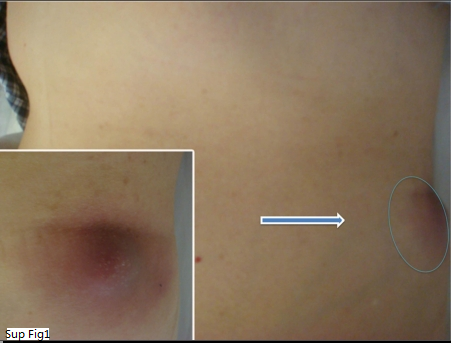


**Supplementary Figure S2** Results of whole body PET/CT scan. A staging FDG PET/CT scan showed multiple tumor lesions in the left lung (S2), right frontal lobe of the brain(S3), rib, thoracic vertebra(s4), and the subcutaneous tissue of the trunk(S5)to be FDG-avid.

**
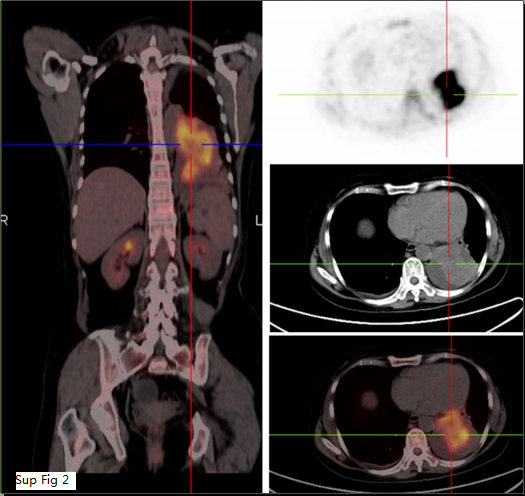
**

**Supplementary Figure S3** Results of whole body PET/CT scan. A staging FDG PET/CT scan showed multiple tumor lesions in the left lung (S2), right frontal lobe of the brain(S3), rib, thoracic vertebra(s4), and the subcutaneous tissue of the trunk(S5)to be FDG-avid.

**
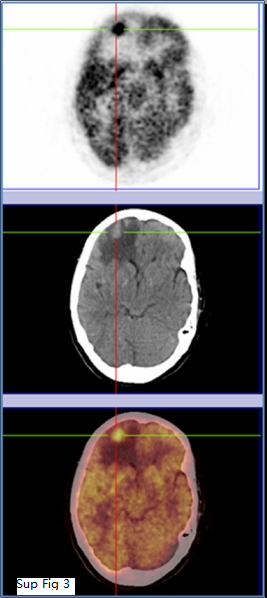
**

**Supplementary Figure S4** Results of whole body PET/CT scan. A staging FDG PET/CT scan showed multiple tumor lesions in the left lung (S2), right frontal lobe of the brain(S3), rib, thoracic vertebra(s4), and the subcutaneous tissue of the trunk(S5)to be FDG-avid.

**
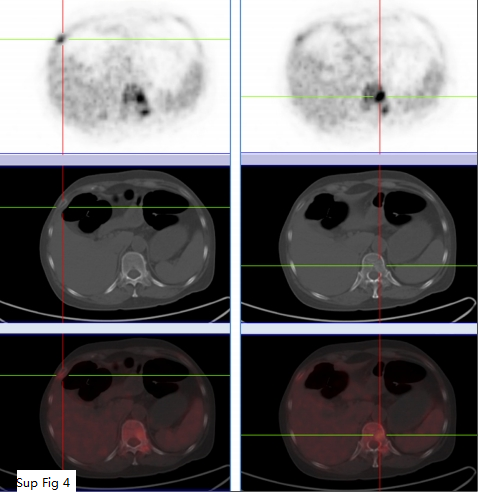
**

**Supplementary Figure S5** Results of whole body PET/CT scan. A staging FDG PET/CT scan showed multiple tumor lesions in the left lung (S2), right frontal lobe of the brain(S3), rib, thoracic vertebra(s4), and the subcutaneous tissue of the trunk(S5)to be FDG-avid.

**
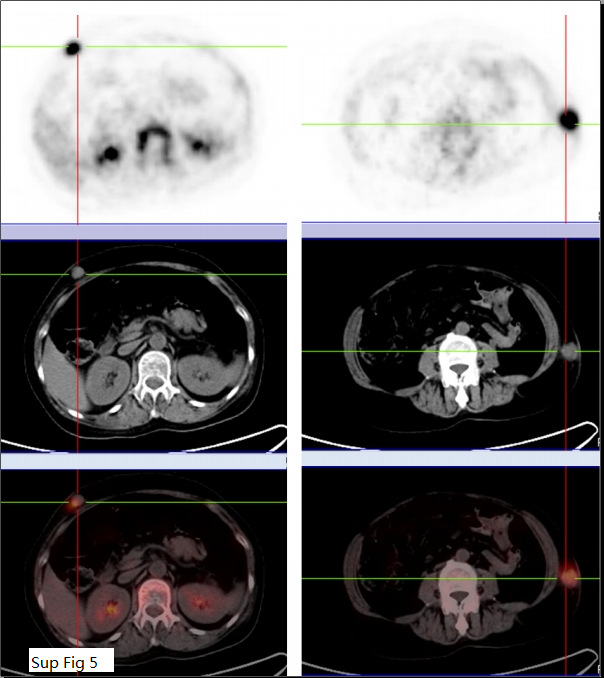
**

**Supplementary Figure S6:** The purity estimation results of thyroid and skin. Sup Fig 6a, 6e and 6i showed the allele-specific copy number across the genome of lung, thyroid and skin tumor tissues.The copy number of both alleles was shown. The aberration reliability score plots in Sup Fig 6b, 6f and 6j showed the confidence in each detected aberration, compared to the hypothesis of no aberration. Sup Fig 6c, 6d, 6f, 6g, 6k and 6l showed the data after application of the ASPCF segmentation algorithm. LogR: the total signal intensity; BAF: B allele frequency.

**
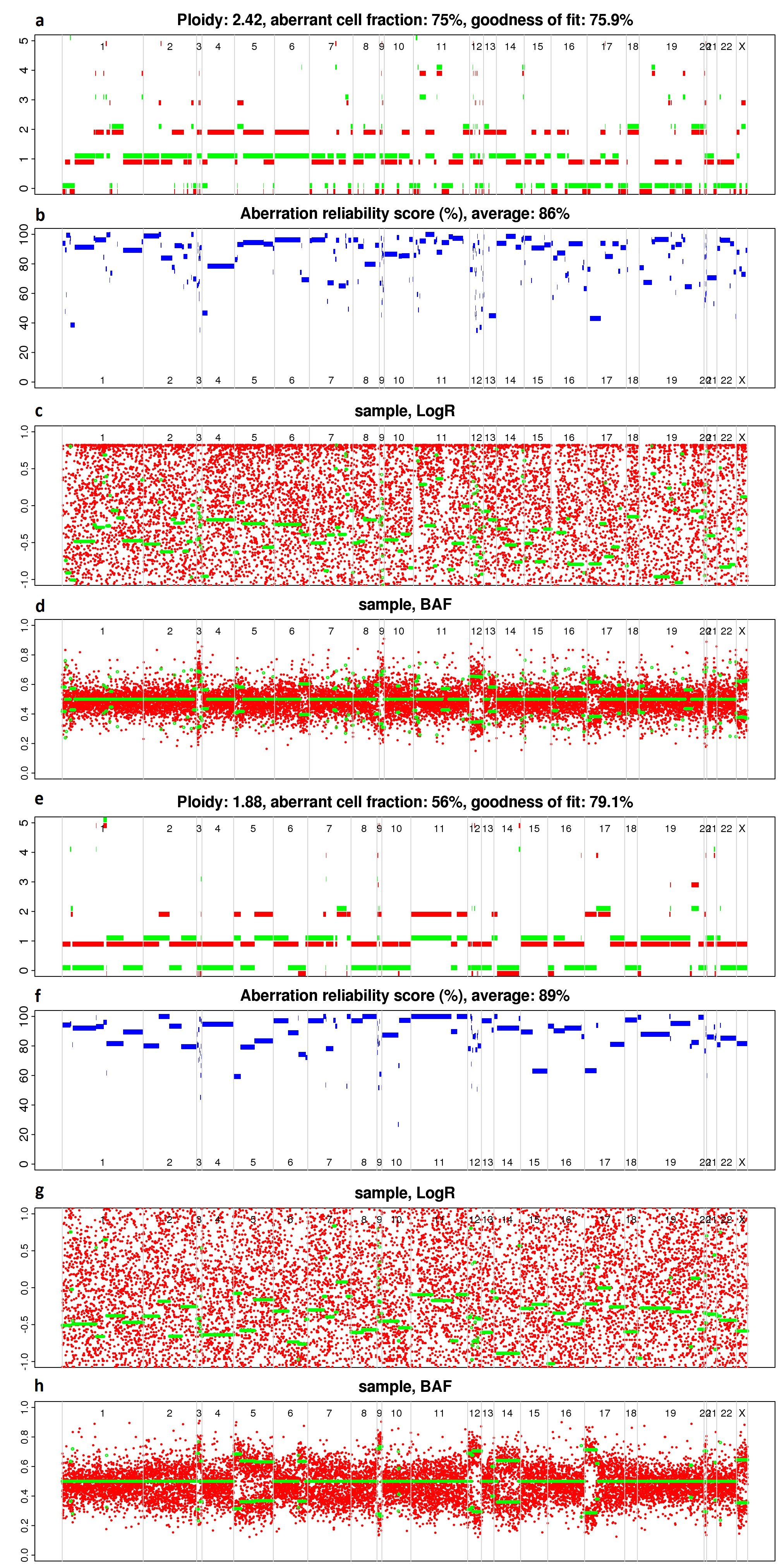
**

**Supplementary Figure S7:** The mutation spetrum of somatic snv in three cancer tissues.

**
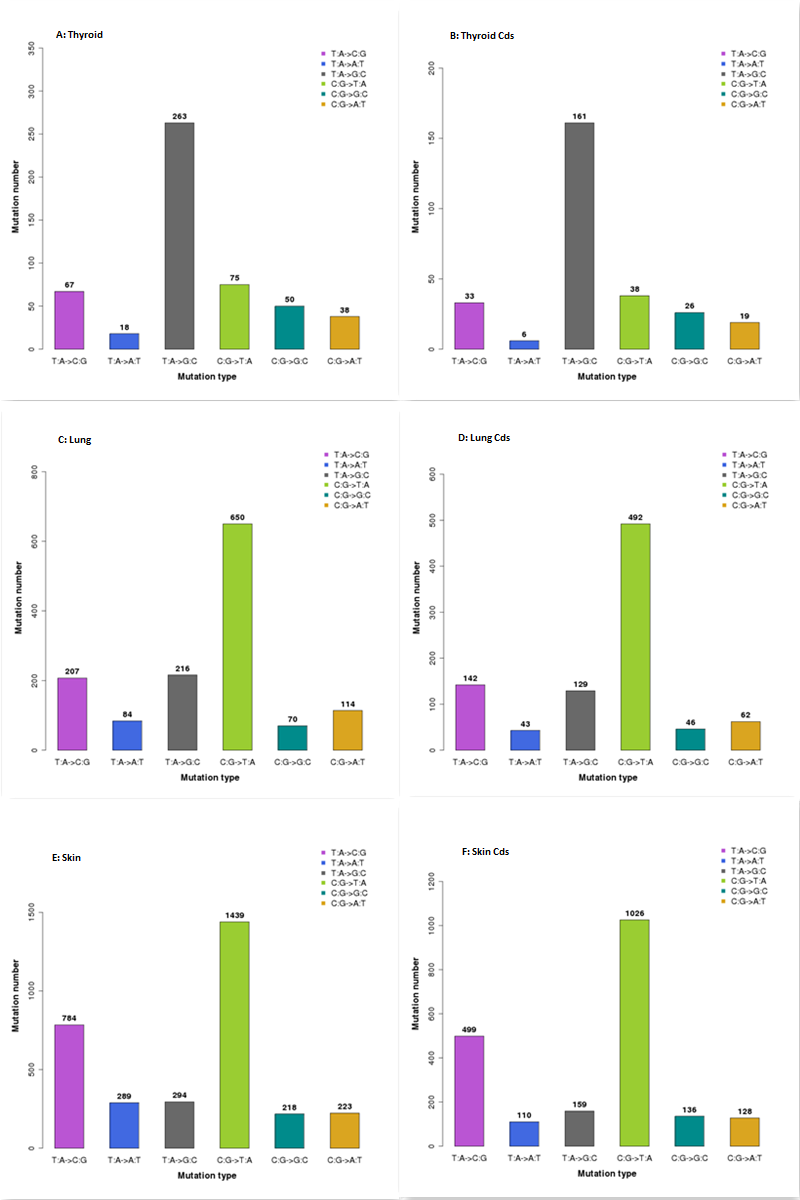
**
