# Supplementary material for: Case report of a Li–Fraumeni syndrome-like phenotype with a de novo mutation in CHEK2
Source: Medicine (Baltimore). 2016 Jul 22;95(29):e4251. doi: 10.1097/MD.0000000000004251 (PMC5265769; doi:10.1097/MD.0000000000004251)
Supplement: Supplemental Digital Content [file medi-95-e4251-s002.doc]

| **Table S1: the basic data production** | | | | | | | | |
| --- | --- | --- | --- | --- | --- | --- | --- | --- |
| **Sample** | **lung** | | **thyroid** | | **skin** | | **normal** | |
| Type | Raw data | Clean data | Raw data | Clean data | Raw data | Clean data | Raw data | Clean data |
| Number of Reads | 179031212 | 169161880 | 173388362 | 167780856 | 203548666 | 193151878 | 191987758 | 185490440 |
| Data Size | 16112809080 | 15224569200 | 15604952580 | 15100277040 | 18319379940 | 17383669020 | 17278898220 | 16694139600 |
| N of fq1 | 496611 | 395977 | 417354 | 313281 | 589884 | 362625 | 495906 | 392812 |
| N of fq2 | 3430638 | 69017 | 2341874 | 62917 | 2700039 | 80441 | 3267421 | 71796 |
| GC(%) of fq1 | 49.57 | 49.35 | 45.9 | 45.79 | 50.54 | 50.36 | 45.96 | 45.77 |
| GC(%) of fq2 | 49.6 | 49.31 | 45.97 | 45.86 | 50.57 | 50.36 | 46.04 | 45.85 |
| Q20(%) of fq1 | 95.82 | 96.82 | 97.31 | 97.86 | 95.68 | 96.59 | 96.6 | 97.3 |
| Q20(%) of fq2 | 92.53 | 95.26 | 94.34 | 96.32 | 92.95 | 95.25 | 94.06 | 95.97 |
| Q30(%) of fq1 | 88.98 | 90.23 | 92.32 | 93.06 | 88.3 | 89.46 | 90.4 | 91.31 |
| Q30(%) of fq2 | 85.08 | 87.8 | 88.76 | 90.75 | 85.05 | 87.36 | 87.31 | 89.23 |
| Discard Reads related to N | 122218 | | 95192 | | 120662 | | 121424 | |
| Discard Reads related to low qual | 7559102 | | 5065930 | | 7168356 | | 5576196 | |
| Discard Reads related to Adapter | 2188012 | | 446384 | | 3107770 | | 799698 | |
| Clean data/Raw data | 94.49% | | 96.77% | | 94.89% | | 96.62% | |
|  |  |  |  |  |  |  |  |  |

| **Table S2: the alignmental QC result** | | | | |
| --- | --- | --- | --- | --- |
| **fsuppSample** | **lung** | **thyroid** | **skin** | **normal** |
| Initial bases on target | 46019796 | 46019796 | 46019796 | 46019796 |
| Initial bases near target | 67405926 | 67405926 | 67405926 | 67405926 |
| Initial bases on or near target | 113425722 | 113425722 | 113425722 | 113425722 |
| Total effective reads | 97307090 | 141190414 | 92384614 | 128707336 |
| Total effective yield(Mb) | 8472.02 | 12515.1 | 8062.46 | 11259.62 |
| Average read length(bp) | 87.06 | 88.64 | 87.27 | 87.48 |
| Effective sequences on target(Mb) | 5195.86 | 6515.44 | 5030.72 | 6514.88 |
| Effective sequences near target(Mb) | 1174.07 | 2193.38 | 1019.77 | 1938.89 |
| Effective sequences on or near target(Mb) | 6369.94 | 8708.82 | 6050.49 | 8453.77 |
| Number of reads uniquely mapped to target | 61404859 | 79684206 | 58836819 | 78045722 |
| Number of reads uniquely mapped to genome | 84322988 | 127322515 | 80080869 | 112117390 |
| Fraction of effective bases on target | 61.30% | 52.10% | 62.40% | 57.90% |
| Fraction of uniquely mapped on target | 72.80% | 62.60% | 73.50% | 69.60% |
| Fraction of effective bases on or near target | 75.20% | 69.60% | 75.00% | 75.10% |
| Average sequencing depth on target | 112.9 | 141.58 | 109.32 | 141.57 |
| Average sequencing depth near target | 17.42 | 32.54 | 15.13 | 28.76 |
| Mismatch rate in target region | 0.30% | 0.22% | 0.28% | 0.26% |
| Mismatch rate in all effective sequence | 0.39% | 0.25% | 0.37% | 0.31% |
| Base covered on target | 45789792 | 45684140 | 45825983 | 45807394 |
| Coverage of target region | 99.50% | 99.30% | 99.60% | 99.50% |
| Base covered near target | 57310965 | 64775529 | 52771424 | 64291023 |
| Coverage of flanking region | 85.00% | 96.10% | 78.30% | 95.40% |
| Fraction of target covered with at least 20x | 92.40% | 89.80% | 91.10% | 92.80% |
| Fraction of target covered with at least 10x | 96.30% | 94.30% | 95.80% | 96.70% |
| Fraction of target covered with at least 4x | 98.50% | 97.50% | 98.40% | 98.70% |
| Fraction of flanking region covered with at least 20x | 25.60% | 43.70% | 21.60% | 39.00% |
| Fraction of flanking region covered with at least 10x | 39.30% | 61.80% | 33.60% | 56.50% |
| Fraction of flanking region covered with at least 4x | 58.70% | 82.20% | 51.30% | 78.40% |
| Mapping rate | 94.90% | 98.48% | 94.58% | 96.90% |
| Duplicate rate | 39.39% | 14.55% | 49.43% | 28.39% |
|  |  |  |  |  |

| **Table S3: the snp annotation result** | | | | |
| --- | --- | --- | --- | --- |
| **Sample** | **lung** | **thyroid** | **skin** | **normal** |
| Total | 30888 | 30442 | 30779 | 30998 |
| 1000genome and dbsnp137 | 29402 | 28964 | 29284 | 29495 |
| 1000genome specific | 1 | 1 | 1 | 1 |
| dbSNP137 specific | 990 | 988 | 981 | 979 |
| dbSNP rate | 98.39% | 98.39% | 98.33% | 98.31% |
| Novel | 495 | 489 | 513 | 523 |
| Hom | 12744 | 12763 | 12017 | 12945 |
| Het | 18144 | 17679 | 18762 | 18053 |
| Synonymous | 9842 | 9666 | 9873 | 9864 |
| Missense | 8210 | 8046 | 8185 | 8218 |
| Stopgain | 62 | 66 | 63 | 64 |
| Stoploss | 28 | 26 | 27 | 26 |
| Exonic | 17891 | 17552 | 17901 | 17921 |
| Exonic and splicing | 251 | 252 | 247 | 251 |
| Splicing | 51 | 49 | 50 | 51 |
| NcRNA | 472 | 477 | 463 | 480 |
| UTR5 | 444 | 408 | 448 | 457 |
| UTR5 and UTR3 | 3 | 3 | 3 | 3 |
| UTR3 | 681 | 668 | 679 | 685 |
| Intronic | 10185 | 10080 | 10100 | 10207 |
| Upstream | 84 | 83 | 84 | 85 |
| Upstream and downstream | 2 | 2 | 2 | 2 |
| Downstream | 39 | 40 | 38 | 40 |
| Intergenic | 785 | 828 | 764 | 816 |
| SIFT | 1704 | 1679 | 1701 | 1727 |
| Ti/Tv | 2.811 | 2.8005 | 2.8202 | 2.7955 |
| dbSNP Ti/Tv | 2.8195 | 2.8126 | 2.8325 | 2.8102 |
| Novel Ti/Tv | 2.3446 | 2.1753 | 2.2062 | 2.0947 |
|  |  |  |  |  |

| **Table S4: the indel annotation result** | | | | |
| --- | --- | --- | --- | --- |
| **Sample** | **lung** | **thyroid** | **skin** | **normal** |
| Total | 2327 | 2254 | 2088 | 2383 |
| 1000genome and dbsnp137 | 901 | 879 | 865 | 908 |
| 1000genome specific | 143 | 138 | 135 | 142 |
| dbSNP137 specific | 748 | 768 | 678 | 787 |
| dbSNP rate | 70.86% | 73.07% | 73.90% | 71.13% |
| Novel | 535 | 496 | 410 | 546 |
| Hom | 871 | 941 | 818 | 966 |
| Het | 1456 | 1313 | 1270 | 1417 |
| Frameshift Insertion | 94 | 94 | 105 | 98 |
| Non-frameshift Insertion | 59 | 55 | 61 | 60 |
| Frameshift Deletion | 79 | 70 | 78 | 77 |
| Non-frameshift Deletion | 81 | 79 | 82 | 82 |
| Frameshift block substitution | 0 | 0 | 0 | 0 |
| Non-frameshift block substitution | 0 | 0 | 0 | 0 |
| Stopgain | 3 | 3 | 3 | 3 |
| Stoploss | 1 | 1 | 1 | 1 |
| Exonic | 314 | 299 | 326 | 316 |
| Exonic and splicing | 3 | 3 | 4 | 5 |
| Splicing | 31 | 32 | 27 | 28 |
| NcRNA | 47 | 43 | 36 | 49 |
| UTR5 | 42 | 39 | 40 | 44 |
| UTR5 and UTR3 | 0 | 0 | 0 | 0 |
| UTR3 | 88 | 95 | 79 | 91 |
| Intronic | 1733 | 1674 | 1512 | 1778 |
| Upstream | 11 | 12 | 10 | 12 |
| Upstream and downstream | 0 | 0 | 0 | 0 |
| Downstream | 6 | 6 | 4 | 6 |
| Intergenic | 52 | 51 | 50 | 54 |
|  |  |  |  |  |

| **Table S5: The germlinemutaions in cancer related gene** | | | | | | | | |
| --- | --- | --- | --- | --- | --- | --- | --- | --- |
| **Gene** | **Chr** | **Function** | **Exonic function** | **1000genome** | **Dbsnp137** | **Position** | **REF** | **ALT** |
| ALK(--) | chr2 | exonic | nonsynonymous SNV | 0.003 | rs35073634 | 29449820 | G | A |
| APC(suppressor gene) | chr5 | exonic | nonsynonymous SNV |  | | 112174665 | T | C |
| BLM(suppressor gene) | chr15 | exonic | nonsynonymous SNV | 0.02 | rs28384991 | 91295110 | C | T |
| BRCA1(suppressorgene) | chr17 | exonic | nonsynonymous SNV |  | | 41244376 | T | C |
| BRCA1(suppressorgene) | chr17 | exonic | nonsynonymous SNV | 0.27 | rs16941 | 41244435 | T | C |
| BRCA1(suppressorgene) | chr17 | exonic | nonsynonymous SNV | 0.3 | rs16942 | 41244000 | T | C |
| BRCA1(suppressorgene) | chr17 | exonic | nonsynonymous SNV | 0.3 | rs1799966 | 41223094 | T | C |
| CHEK2(suppressorgene) | chr22 | exonic | nonsynonymous SNV |  | | 29091846 | G | A |
| EGFR(Oncogene) | chr7 | exonic | nonsynonymous SNV | 0.25 | rs2227983 | 55229255 | G | A |
| ERCC5(--) | chr13 | exonic | nonsynonymous SNV | 0.39 | rs17655 | 103528002 | G | C |
| FANCA(--) | chr16 | exonic | nonsynonymous SNV | 0.07 | rs11646374 | 89857935 | G | A |
| FANCA(--) | chr16 | exonic | nonsynonymous SNV | 0.6 | rs2239359 | 89849480 | C | T |
| MSH6(suppressor gene) | chr2 | exonic | nonsynonymous SNV | 0.18 | rs1042821 | 48010488 | G | A |
| PMS2(other) | chr7 | exonic | nonsynonymous SNV | 0.04 | rs74902811 | 6026864 | G | A |
| SETBP1(--) | chr18 | exonic | nonsynonymous SNV | 0.11 | rs1064204 | 42532693 | C | A |
|  |  |  |  |  |  |  |  |  |

| **Table S6: The somatic SNV of thyroid tumor tissue** | | | | | | |  |  |  |  |  |  |  |  |  |  |  |  |  |  |  |  |  |  |  |  |  |  |  |  |  |  |  |  |
| --- | --- | --- | --- | --- | --- | --- | --- | --- | --- | --- | --- | --- | --- | --- | --- | --- | --- | --- | --- | --- | --- | --- | --- | --- | --- | --- | --- | --- | --- | --- | --- | --- | --- | --- |
| **Func** | | **Gene** | **ExonicFunc** | **AAChange** | **Conserved** | **SegDup** | **1000G_ALL** | **1000G_ALL** | **1000G_ALL** | **dbSNP137** | **SIFT** | **PolyPhen2** | **LJB_PhyloP** | **LJB_MutationTaster** | **LJB_LRT** | **Chr** | **Start** | **End** | **Ref** | **Obs** | **Genotype** | **Cosmic** |  |  | **normal_reads1** | **normal_reads2** | **normal_var_freq** | **normal_gt** | **tumor_reads1** | **tumor_reads2** | **tumor_var_freq** | **tumor_gt** | **somatic_status** | **somatic_p_value** |
| intergenic | | TMEM121(dist=177251),KIAA0125(dist=210048) |  |  |  | 0.97 | 0.48 | 0.48 | 0.48 | rs8709 |  |  |  |  |  | chr14 | 106173790 | 106173790 | A | G | hom | . | . | . | 176 | 3 | 1.68% | A | 77 | 772 | 90.93% | G | Somatic | 1.60E-131 |
| intergenic | | TMEM121(dist=113013),KIAA0125(dist=274286) |  |  | 289;Name=lod=20 | 0.96 |  |  |  | rs1051611 |  |  |  |  |  | chr14 | 106109552 | 106109552 | A | G | het | . | . | . | 151 | 2 | 1.31% | A | 75 | 105 | 58.33% | R | Somatic | 2.97E-34 |
| intergenic | | TMEM121(dist=177581),KIAA0125(dist=209718) |  |  | 265;Name=lod=16 | 0.97 |  |  |  |  |  |  |  |  |  | chr14 | 106174120 | 106174120 | A | T | het | . | . | . | 61 | 0 | 0% | A | 41 | 46 | 52.87% | W | Somatic | 2.37E-14 |
| intergenic | | TMEM121(dist=211283),KIAA0125(dist=176016) |  |  | 366;Name=lod=41 | 0.96 |  |  |  | rs1051611 |  |  |  |  |  | chr14 | 106207822 | 106207822 | A | G | het | . | . | . | 184 | 3 | 1.60% | A | 134 | 122 | 47.66% | R | Somatic | 3.82E-32 |
| intergenic | | TMEM121(dist=239047),KIAA0125(dist=148252) |  |  |  | 0.95 | 0.29 | 0.29 | 0.29 | rs1051611 |  |  |  |  |  | chr14 | 106235586 | 106235586 | A | G | het | . | . | . | 1448 | 11 | 0.75% | A | 245 | 206 | 45.68% | R | Somatic | 4.94E-132 |
| intergenic | | TMEM121(dist=211394),KIAA0125(dist=175905) |  |  | 387;Name=lod=50 | 0.96 | 0.15 | 0.15 | 0.15 | rs17841087 |  |  |  |  |  | chr14 | 106207933 | 106207933 | G | A | het | . | . | . | 476 | 8 | 1.65% | G | 299 | 227 | 43.16% | R | Somatic | 1.48E-65 |
| exonic | | SLC22A5 | nonsynonymous SNV | NM_003060:c.G259C:p.A87P |  |  |  |  |  |  | 0.15 | 0.003 | 0.990047 | 0.749254 | 0.879283 | chr5 | 131705923 | 131705923 | G | C | het | . | . | . | 10 | 0 | 0% | G | 6 | 4 | 40% | S | Somatic | 0.0433437 |
| intronic | | MYOF |  |  |  |  |  |  |  |  |  |  |  |  |  | chr10 | 95107336 | 95107336 | G | A | het | . | . | . | 29 | 0 | 0% | G | 14 | 9 | 39.13% | R | Somatic | 2.22E-04 |
| exonic | | MUC4 | nonsynonymous SNV | NM_018406:c.G2857C:p.E953Q |  |  | 0.76 | 0.76 | 0.76 | rs13095016 | 0.33 |  |  |  |  | chr3 | 195515594 | 195515594 | C | G | het | . | . | . | 17 | 0 | 0% | C | 33 | 21 | 38.89% | S | Somatic | 9.51E-04 |
| intronic | | C3orf25 |  |  |  |  | 0.4 | 0.4 | 0.4 | rs6764765 |  |  |  |  |  | chr3 | 129120743 | 129120743 | A | G | het | . | . | . | 14 | 0 | 0% | A | 18 | 11 | 37.93% | R | Somatic | 0.0060148 |
| exonic | | TNXB | nonsynonymous SNV | NM_019105:c.T9332G:p.V3111G |  | 0.94 |  |  |  |  | 0.01 |  |  |  |  | chr6 | 32017876 | 32017876 | A | C | het | . | . | . | 23 | 0 | 0% | A | 7 | 4 | 36.36% | M | Somatic | 0.0071158 |
| intronic | | TRIM24 |  |  |  |  |  |  |  |  |  |  |  |  |  | chr7 | 138189190 | 138189190 | C | T | het | . | . | . | 32 | 0 | 0% | C | 35 | 20 | 36.36% | Y | Somatic | 2.13E-05 |
| intronic | | SREBF1 |  |  |  |  |  |  |  |  |  |  |  |  |  | chr17 | 17723367 | 17723367 | A | C | het | . | . | . | 14 | 0 | 0% | A | 7 | 4 | 36.36% | M | Somatic | 0.026087 |
| intergenic | | TMEM121(dist=57020),KIAA0125(dist=330279) |  |  |  | 0.97 | 0.18 | 0.18 | 0.18 | rs8709 |  |  |  |  |  | chr14 | 106053559 | 106053559 | A | G | het | . | . | . | 51 | 0 | 0% | A | 30 | 17 | 36.17% | R | Somatic | 6.00E-07 |
| intergenic | | TMEM121(dist=178466),KIAA0125(dist=208833) |  |  |  | 0.97 |  |  |  |  |  |  |  |  |  | chr14 | 106175005 | 106175005 | G | A | het | . | . | . | 35 | 0 | 0% | G | 25 | 14 | 35.90% | R | Somatic | 3.31E-05 |
| intergenic | | TMEM121(dist=211543),KIAA0125(dist=175756) |  |  |  | 0.96 | 0.07 | 0.07 | 0.07 | rs11621259 |  |  |  |  |  | chr14 | 106208082 | 106208082 | G | T | het | . | . | . | 289 | 4 | 1.37% | G | 178 | 91 | 33.83% | K | Somatic | 2.56E-28 |
| intergenic | | MIR4436A(dist=887388),LOC654342(dist=1825353) |  |  |  | 0.99 |  |  |  |  |  |  |  |  |  | chr2 | 89999356 | 89999356 | A | G | het | . | . | . | 144 | 0 | 0% | A | 206 | 105 | 33.76% | R | Somatic | 4.50E-21 |
| exonic | | COL22A1 | nonsynonymous SNV | NM_152888:c.A1892C:p.Q631P | 363;Name=lod=40 |  |  |  |  |  | 0.23 | 0.242094 | 0.200895 | 0.013001 | 0.497769 | chr8 | 139772496 | 139772496 | T | G | het | . | . | . | 12 | 0 | 0% | T | 8 | 4 | 33.33% | K | Somatic | 0.0465839 |
| intronic | | NANP |  |  |  |  |  |  |  |  |  |  |  |  |  | chr20 | 25604483 | 25604483 | T | G | het | . | . | . | 39 | 0 | 0% | T | 8 | 4 | 33.33% | K | Somatic | 0.0019808 |
| intergenic | | TMEM121(dist=177362),KIAA0125(dist=209937) |  |  |  | 0.97 |  |  |  |  |  |  |  |  |  | chr14 | 106173901 | 106173901 | T | G | het | . | . | . | 76 | 0 | 0% | T | 49 | 24 | 32.88% | K | Somatic | 3.63E-09 |
| intergenic | | LINC00221(dist=218640),NONE(dist=NONE) |  |  |  |  |  |  |  |  | 0.04 |  |  |  |  | chr14 | 107170169 | 107170169 | T | C | het | . | . | . | 434 | 0 | 0% | T | 302 | 142 | 31.98% | Y | Somatic | 1.43E-48 |
| intergenic | | TMEM121(dist=178153),KIAA0125(dist=209146) |  |  |  | 0.97 |  |  |  | rs111425248 |  |  |  |  |  | chr14 | 106174692 | 106174692 | T | G | het | . | . | . | 110 | 0 | 0% | T | 47 | 21 | 30.88% | K | Somatic | 1.80E-10 |
| intergenic | | LINC00221(dist=218633),NONE(dist=NONE) |  |  | 235;Name=lod=12 |  |  |  |  |  |  |  |  |  |  | chr14 | 107170162 | 107170162 | G | A | het | . | . | . | 452 | 0 | 0% | G | 303 | 135 | 30.82% | R | Somatic | 1.06E-47 |
| intergenic | | DUX2(dist=2842),DUX4L4(dist=2142) |  |  |  | 1 |  |  |  |  | 0.19 |  |  |  |  | chr4 | 191003131 | 191003131 | G | C | het | . | . | . | 19 | 0 | 0% | G | 7 | 3 | 30% | S | Somatic | 0.0328407 |
| exonic | | HCN4 | synonymous SNV | NM_005477:c.T3072G:p.G1024G | 496;Name=lod=138 |  |  |  |  |  |  |  |  |  |  | chr15 | 73615362 | 73615362 | A | C | het | . | . | . | 16 | 0 | 0% | A | 7 | 3 | 30% | M | Somatic | 0.0461538 |
| intronic | | DNAH17 |  |  |  |  |  |  |  |  |  |  |  |  |  | chr17 | 76487688 | 76487688 | A | C | het | . | . | . | 16 | 0 | 0% | A | 7 | 3 | 30% | M | Somatic | 0.0461538 |
| intergenic | | LINC00221(dist=218627),NONE(dist=NONE) |  |  |  |  |  |  |  |  |  |  |  |  |  | chr14 | 107170156 | 107170156 | C | T | het | . | . | . | 455 | 0 | 0% | C | 314 | 134 | 29.91% | Y | Somatic | 1.18E-46 |
| exonic | | GSN | nonsynonymous SNV | NM_000177:c.A1687C:p.T563P | 572;Name=lod=282 |  |  |  |  |  | 0 | 0.001 | 0.99793 | 0.994097 | 0.999991 | chr9 | 124088907 | 124088907 | A | C | het | . | . | . | 22 | 0 | 0% | A | 17 | 7 | 29.17% | M | Somatic | 0.0064663 |
| intergenic | | MIR4436A(dist=409413),NONE(dist=NONE) |  |  |  | 0.99 |  |  |  |  | 0.11 |  |  |  |  | chr2 | 89521381 | 89521381 | T | C | het | . | . | . | 117 | 0 | 0% | T | 239 | 97 | 28.87% | Y | Somatic | 3.45E-15 |
| intronic | | ARHGAP9 |  |  |  |  |  |  |  |  |  |  |  |  |  | chr12 | 57869427 | 57869427 | T | G | het | . | . | . | 37 | 0 | 0% | T | 10 | 4 | 28.57% | K | Somatic | 0.0040056 |
| intergenic | | TMEM121(dist=57131),KIAA0125(dist=330168) |  |  |  | 0.97 |  |  |  |  |  |  |  |  |  | chr14 | 106053670 | 106053670 | T | G | het | . | . | . | 43 | 0 | 0% | T | 20 | 8 | 28.57% | K | Somatic | 2.92E-04 |
| intronic | | TMEM101 |  |  | 259;Name=lod=15 |  |  |  |  |  |  |  |  |  |  | chr17 | 42090542 | 42090542 | A | C | het | . | . | . | 24 | 0 | 0% | A | 10 | 4 | 28.57% | M | Somatic | 0.0135609 |
| intergenic | | LINC00221(dist=218616),NONE(dist=NONE) |  |  |  |  |  |  |  | rs189122962 | 0.46 |  |  |  |  | chr14 | 107170145 | 107170145 | C | T | het | . | . | . | 425 | 2 | 0.47% | C | 326 | 130 | 28.51% | Y | Somatic | 4.07E-39 |
| intergenic | | LINC00221(dist=218611),NONE(dist=NONE) |  |  | 259;Name=lod=15 |  |  |  |  |  | 0.05 |  |  |  |  | chr14 | 107170140 | 107170140 | A | G | het | . | . | . | 347 | 0 | 0% | A | 344 | 131 | 27.58% | R | Somatic | 8.72E-36 |
| ncRNA_exonic | | LOC642846 |  |  |  | 0.99 |  |  |  | rs2536755 | 0.29 |  |  |  |  | chr12 | 9447492 | 9447492 | T | C | het | . | . | . | 21 | 0 | 0% | T | 16 | 6 | 27.27% | Y | Somatic | 0.0122388 |
| intergenic | | LINC00221(dist=218607),NONE(dist=NONE) |  |  | 259;Name=lod=15 |  |  |  |  |  | 0.67 |  |  |  |  | chr14 | 107170136 | 107170136 | C | G | het | . | . | . | 332 | 1 | 0.30% | C | 347 | 130 | 27.25% | S | Somatic | 1.71E-32 |
| intronic | | ULK1 |  |  |  |  |  |  |  |  |  |  |  |  |  | chr12 | 132394385 | 132394385 | A | C | het | . | . | . | 43 | 0 | 0% | A | 11 | 4 | 26.67% | M | Somatic | 0.0032173 |
| intronic | | H1FOO |  |  |  |  |  |  |  |  |  |  |  |  |  | chr3 | 129268231 | 129268231 | T | G | het | . | . | . | 20 | 0 | 0% | T | 14 | 5 | 26.32% | K | Somatic | 0.020196 |
| exonic | | HLA-DPB1 | nonsynonymous SNV | NM_002121:c.G700A:p.V234M | 272;Name=lod=17 |  | 0.13 | 0.13 | 0.13 | rs11551421 | 0.02 | 0 | 0.962419 | 0.54672 | 0.99806 | chr6 | 33053609 | 33053609 | G | A | het | . | . | . | 16 | 0 | 0% | G | 14 | 5 | 26.32% | R | Somatic | 0.035819 |
| exonic | | SH2D2A | nonsynonymous SNV | NM_001161443:c.A983C:p.H328P |  |  |  |  |  |  | 0.02 | 0.969 | 0.948835 | 0.296847 | 0.016398 | chr1 | 156777073 | 156777073 | T | G | het | . | . | . | 25 | 0 | 0% | T | 31 | 11 | 26.19% | K | Somatic | 0.003331 |
| exonic | | CLCN1 | nonsynonymous SNV | NM_000083:c.G152C:p.G51A |  |  |  |  |  | rs75643846 | 0.13 | 0.001 | 0.988519 | 0.016364 | 0.650654 | chr7 | 143013457 | 143013457 | G | C | het | . | . | . | 80 | 0 | 0% | G | 147 | 52 | 26.13% | S | Somatic | 2.46E-09 |
| intergenic | | TMEM121(dist=178152),KIAA0125(dist=209147) |  |  |  | 0.97 |  |  |  |  |  |  |  |  |  | chr14 | 106174691 | 106174691 | C | G | het | . | . | . | 109 | 0 | 0% | C | 47 | 16 | 25.40% | S | Somatic | 2.68E-08 |
| intronic | | ATP2A3 |  |  |  |  |  |  |  |  |  |  |  |  |  | chr17 | 3833765 | 3833765 | T | G | het | . | . | . | 17 | 0 | 0% | T | 12 | 4 | 25% | K | Somatic | 0.044477 |
| exonic | | COPE | nonsynonymous SNV | NM_007263:c.A352C:p.T118P | 573;Name=lod=285 |  |  |  |  |  | 0.04 | 0.255 | 0.995366 | 0.996719 | 1 | chr19 | 19017860 | 19017860 | T | G | het | . | . | . | 22 | 0 | 0% | T | 9 | 3 | 25% | K | Somatic | 0.0367647 |
| UTR5 | | GRIK1 |  |  | 395;Name=lod=54 |  |  |  |  |  |  |  |  |  |  | chr21 | 31311877 | 31311877 | A | C | het | . | . | . | 20 | 0 | 0% | A | 18 | 6 | 25% | M | Somatic | 0.0190672 |
| exonic | | ELK1 | nonsynonymous SNV | NM_005229:c.A487C:p.T163P | 541;Name=lod=211 | 0.93 |  |  |  |  | 0 | 0.98 | 0.996959 | 0.999574 | 1 | chrX | 47498461 | 47498461 | T | G | het | . | . | . | 36 | 0 | 0% | T | 15 | 5 | 25% | K | Somatic | 0.0040588 |
| intergenic | | LINC00221(dist=218683),NONE(dist=NONE) |  |  |  |  |  |  |  |  | 0.02 |  |  |  |  | chr14 | 107170212 | 107170212 | C | G | het | . | . | . | 300 | 2 | 0.66% | C | 278 | 90 | 24.46% | S | Somatic | 1.58E-23 |
| intronic | | ARL6IP1 |  |  |  |  |  |  |  |  |  |  |  |  |  | chr16 | 18804752 | 18804752 | A | T | het | . | . | . | 16 | 0 | 0% | A | 25 | 8 | 24.24% | W | Somatic | 0.0307868 |
| intergenic | | TMEM121(dist=178151),KIAA0125(dist=209148) |  |  |  | 0.97 |  |  |  |  |  |  |  |  |  | chr14 | 106174690 | 106174690 | T | A | het | . | . | . | 108 | 0 | 0% | T | 47 | 15 | 24.19% | W | Somatic | 8.03E-08 |
| ncRNA_exonic | | MIR663B |  |  |  | 0.91 |  |  |  |  |  |  |  |  |  | chr2 | 133014617 | 133014617 | A | G | het | . | . | . | 47 | 0 | 0% | A | 13 | 4 | 23.53% | R | Somatic | 0.0037458 |
| exonic | | ADAD2 | nonsynonymous SNV | NM_001145400:c.A1063C:p.T355P |  |  |  |  |  |  | 0.19 | 0.09 | 0.025761 | 0.018445 | 0.476572 | chr16 | 84229431 | 84229431 | A | C | het | . | . | . | 51 | 0 | 0% | A | 13 | 4 | 23.53% | M | Somatic | 0.0029225 |
| intergenic | | LINC00221(dist=218597),NONE(dist=NONE) |  |  |  |  |  |  |  |  |  |  |  |  |  | chr14 | 107170126 | 107170126 | A | G | het | . | . | . | 345 | 2 | 0.58% | A | 374 | 113 | 23.20% | R | Somatic | 1.28E-26 |
| UTR3 | | PTMA |  |  | 588;Name=lod=328 |  |  |  |  |  |  |  |  |  |  | chr2 | 232577585 | 232577585 | C | A | het | . | . | . | 21 | 0 | 0% | C | 10 | 3 | 23.08% | M | Somatic | 0.0477941 |
| UTR3 | | PTMA |  |  | 588;Name=lod=328 |  |  |  |  |  |  |  |  |  |  | chr2 | 232577586 | 232577586 | T | A | het | . | . | . | 21 | 0 | 0% | T | 10 | 3 | 23.08% | W | Somatic | 0.0477941 |
| exonic | | CEBPD | nonsynonymous SNV | NM_005195:c.A217C:p.T73P | 676;Name=lod=744 |  |  |  |  |  | 0.15 | 0.322424 | 0.979443 | 5.14E-04 | 0.355999 | chr8 | 48650466 | 48650466 | T | G | het | . | . | . | 41 | 0 | 0% | T | 10 | 3 | 23.08% | K | Somatic | 0.0115304 |
| intronic | | SLC13A5 |  |  |  |  |  |  |  |  |  |  |  |  |  | chr17 | 6596495 | 6596495 | G | A | het | . | . | . | 22 | 0 | 0% | G | 20 | 6 | 23.08% | R | Somatic | 0.0187613 |
| intronic | | DCAF15 |  |  |  |  |  |  |  |  |  |  |  |  |  | chr19 | 14066889 | 14066889 | G | C | het | . | . | . | 60 | 1 | 1.64% | G | 10 | 3 | 23.08% | S | Somatic | 0.0157836 |
| intergenic | | MED15(dist=80618),POM121L4P(dist=21306) |  |  |  | 0.97 |  |  |  |  |  |  |  |  |  | chr22 | 21022537 | 21022537 | G | T | het | . | . | . | 27 | 0 | 0% | G | 10 | 3 | 23.08% | K | Somatic | 0.0289474 |
| intergenic | | MED15(dist=80625),POM121L4P(dist=21299) |  |  |  | 0.97 |  |  |  |  |  |  |  |  |  | chr22 | 21022544 | 21022544 | G | T | het | . | . | . | 26 | 0 | 0% | G | 10 | 3 | 23.08% | K | Somatic | 0.0312945 |
| intronic | | HSPG2 |  |  | 462;Name=lod=101 |  |  |  |  |  |  |  |  |  |  | chr1 | 22215206 | 22215206 | T | G | het | . | . | . | 31 | 0 | 0% | T | 25 | 7 | 21.88% | K | Somatic | 0.0060836 |
|  | intronic | NPC1 |  |  |  |  |  |  |  |  |  |  |  |  |  | chr18 | 21120398 | 21120398 | G | A | het | . | . | . | 20 | 0 | 0% | G | 18 | 5 | 21.74% | R | Somatic | 0.0349564 |
|  | exonic | TGFBR3 | nonsynonymous SNV | NM_001195683:c.T2467C:p.S823P | 510;Name=lod=158 |  |  |  |  |  | 0.13 | 0.687 | 0.906681 | 0.819556 | 0.996965 | chr1 | 92149382 | 92149382 | A | G | het | . | . | . | 43 | 0 | 0% | A | 11 | 3 | 21.43% | R | Somatic | 0.0124402 |
|  | exonic | DNM3 | nonsynonymous SNV | NM_001136127:c.C142A:p.L48I | 690;Name=lod=847 |  |  |  |  |  | 0.01 |  |  |  |  | chr1 | 171810938 | 171810938 | C | A | het | . | . | . | 25 | 0 | 0% | C | 11 | 3 | 21.43% | M | Somatic | 0.0398293 |
|  | intronic | DUX4L3,DUX4L5,DUX4L6,DUX4L7 |  |  | 417;Name=lod=66 | 1 |  |  |  |  |  |  |  |  |  | chr10 | 135491299 | 135491299 | G | A | het | . | . | . | 33 | 0 | 0% | G | 11 | 3 | 21.43% | R | Somatic | 0.0224484 |
|  | exonic | TMEM161A | nonsynonymous SNV | NM_017814:c.T986G:p.V329G | 606;Name=lod=388 |  |  |  |  |  | 0 | 0.963 | 0.962849 | 0.898651 | 0.999813 | chr19 | 19231904 | 19231904 | A | C | het | . | . | . | 48 | 0 | 0% | A | 22 | 6 | 21.43% | M | Somatic | 0.0017233 |
|  | intergenic | MIR4436A(dist=887457),LOC654342(dist=1825284) |  |  |  | 0.99 |  |  |  |  |  |  |  |  |  | chr2 | 89999425 | 89999425 | G | C | het | . | . | . | 165 | 0 | 0% | G | 280 | 76 | 21.35% | S | Somatic | 1.51E-14 |
|  | UTR3 | PGAM2 |  |  |  |  |  |  |  |  |  |  |  |  |  | chr7 | 44102338 | 44102338 | T | G | het | . | . | . | 26 | 0 | 0% | T | 19 | 5 | 20.83% | K | Somatic | 0.0200608 |
|  | intergenic | MIR4436A(dist=887450),LOC654342(dist=1825291) |  |  | 309;Name=lod=24 | 0.99 |  |  |  |  |  |  |  |  |  | chr2 | 89999418 | 89999418 | T | C | het | . | . | . | 159 | 1 | 0.62% | T | 279 | 73 | 20.74% | Y | Somatic | 2.59E-12 |
|  | intergenic | MIR4436A(dist=409344),NONE(dist=NONE) |  |  |  | 0.99 |  |  |  |  | 1 |  |  |  |  | chr2 | 89521312 | 89521312 | C | G | het | . | . | . | 144 | 0 | 0% | C | 295 | 76 | 20.49% | S | Somatic | 1.31E-12 |
|  | intronic | FBLN2 |  |  |  |  |  |  |  |  |  |  |  |  |  | chr3 | 13663426 | 13663426 | G | C | het | . | . | . | 42 | 0 | 0% | G | 8 | 2 | 20% | S | Somatic | 0.0339367 |
|  | intronic | DGKQ |  |  |  |  |  |  |  |  |  |  |  |  |  | chr4 | 960325 | 960325 | T | G | het | . | . | . | 49 | 0 | 0% | T | 8 | 2 | 20% | K | Somatic | 0.0263004 |
|  | upstream | ULBP3 |  |  |  |  |  |  |  |  |  |  |  |  |  | chr6 | 150390204 | 150390204 | G | A | het | . | . | . | 34 | 0 | 0% | G | 8 | 2 | 20% | R | Somatic | 0.0475687 |
|  | intronic | RELN |  |  |  |  |  |  |  |  |  |  |  |  |  | chr7 | 103202405 | 103202405 | C | A | het | . | . | . | 36 | 0 | 0% | C | 16 | 4 | 20% | M | Somatic | 0.0131912 |
|  | exonic | FAM83A | synonymous SNV | NM_032899:c.A1095C:p.P365P |  |  |  |  |  |  |  |  |  |  |  | chr8 | 124219718 | 124219718 | A | C | het | . | . | . | 30 | 0 | 0% | A | 12 | 3 | 20% | M | Somatic | 0.0320648 |
|  | exonic | RECQL4 | nonsynonymous SNV | NM_004260:c.G2707C:p.G903R | 355;Name=lod=37 |  |  |  |  |  |  |  |  |  |  | chr8 | 145738279 | 145738279 | C | G | het | . | . | . | 37 | 0 | 0% | C | 8 | 2 | 20% | S | Somatic | 0.0416281 |
|  | intronic | B4GALNT4 |  |  |  |  |  |  |  |  |  |  |  |  |  | chr11 | 373423 | 373423 | A | C | het | . | . | . | 32 | 0 | 0% | A | 12 | 3 | 20% | M | Somatic | 0.0280604 |
|  | exonic | USP5 | nonsynonymous SNV | NM_001098536:c.A754C:p.T252P | 493;Name=lod=135 |  |  |  |  |  | 0 | 0.961 | 0.997906 | 0.999995 | 1 | chr12 | 6966040 | 6966040 | A | C | het | . | . | . | 24 | 0 | 0% | A | 52 | 13 | 20% | M | Somatic | 0.0116792 |
|  | exonic | GLTP | nonsynonymous SNV | NM_016433:c.A86C:p.H29P | 617;Name=lod=431 |  |  |  |  |  | 0.02 | 0.996 | 0.934842 | 0.991123 | 0.999997 | chr12 | 110318094 | 110318094 | T | G | het | . | . | . | 33 | 0 | 0% | T | 8 | 2 | 20% | K | Somatic | 0.0498339 |
|  | exonic | CRAMP1L | nonsynonymous SNV | NM_020825:c.A1550G:p.Q517R |  |  |  |  |  |  | 0.01 |  |  |  |  | chr16 | 1706308 | 1706308 | A | G | het | . | . | . | 41 | 0 | 0% | A | 8 | 2 | 20% | R | Somatic | 0.0352941 |
|  | intronic | ABCC6 |  |  |  |  |  |  |  |  |  |  |  |  |  | chr16 | 16248710 | 16248710 | A | C | het | . | . | . | 28 | 0 | 0% | A | 12 | 3 | 20% | M | Somatic | 0.036869 |
|  | exonic | ZNF407 | nonsynonymous SNV | NM_017757:c.T6536G:p.V2179G |  |  |  |  |  |  |  |  |  |  |  | chr18 | 72776213 | 72776213 | T | G | het | . | . | . | 25 | 0 | 0% | T | 12 | 3 | 20% | K | Somatic | 0.0460526 |
|  | exonic | CELF5 | nonsynonymous SNV | NM_001172673:c.G198C:p.E66D | 668;Name=lod=689 |  |  |  |  |  | 0.01 | 0.294 | 0.958507 | 0.768233 | 0.844055 | chr19 | 3224935 | 3224935 | G | C | het | . | . | . | 34 | 0 | 0% | G | 8 | 2 | 20% | S | Somatic | 0.0475687 |
|  | exonic | CIC | nonsynonymous SNV | NM_015125:c.T2258G:p.V753G | 573;Name=lod=285 |  |  |  |  |  |  | 0.137 | 0.996216 | 0.103472 | 0.852081 | chr19 | 42795178 | 42795178 | T | G | het | . | . | . | 56 | 1 | 1.75% | T | 20 | 5 | 20% | K | Somatic | 0.0091544 |
|  | exonic | PPP2R3B | nonsynonymous SNV | NM_013239:c.A1034C:p.H345P | 654;Name=lod=604 | 1 |  |  |  |  | 0.08 |  |  |  |  | chrX | 306253 | 306253 | T | G | het | . | . | . | 44 | 0 | 0% | T | 8 | 2 | 20% | K | Somatic | 0.0314465 |
|  | intergenic | MIR4436A(dist=887440),LOC654342(dist=1825301) |  |  |  | 0.99 |  |  |  |  |  |  |  |  |  | chr2 | 89999408 | 89999408 | G | A | het | . | . | . | 159 | 1 | 0.62% | G | 251 | 62 | 19.81% | R | Somatic | 1.93E-11 |
|  | intergenic | LINC00221(dist=218592),NONE(dist=NONE) |  |  |  |  | 0.003 | 0.003 | 0.003 | rs1064564 | 0.17 |  |  |  |  | chr14 | 107170121 | 107170121 | C | G | het | . | . | . | 349 | 1 | 0.29% | C | 391 | 93 | 19.21% | S | Somatic | 7.59E-23 |
|  | intergenic | MIR4436A(dist=409351),NONE(dist=NONE) |  |  |  | 0.99 |  |  |  |  | 0.01 |  |  |  |  | chr2 | 89521319 | 89521319 | A | G | het | . | . | . | 137 | 0 | 0% | A | 293 | 69 | 19.06% | R | Somatic | 3.26E-11 |
|  | exonic | C8orf80 | nonsynonymous SNV | NM_001010906:c.T2093G:p.V698G | 477;Name=lod=116 |  |  |  |  |  | 0.01 |  |  |  |  | chr8 | 27886844 | 27886844 | A | C | het | . | . | . | 24 | 0 | 0% | A | 34 | 8 | 19.05% | M | Somatic | 0.02055 |
|  | exonic | FAM71F2 | nonsynonymous SNV | NM_001012454:c.T259C:p.S87P | 501;Name=lod=145 |  |  |  |  |  | 0.05 |  |  |  |  | chr7 | 128315807 | 128315807 | T | C | het | . | . | . | 54 | 1 | 1.82% | T | 60 | 14 | 18.92% | Y | Somatic | 0.0017989 |
|  | intronic | DAP3 |  |  |  |  |  |  |  |  |  |  |  |  |  | chr1 | 155671935 | 155671935 | C | T | het | . | . | . | 27 | 0 | 0% | C | 26 | 6 | 18.75% | Y | Somatic | 0.0201119 |
|  | exonic | CHRD | nonsynonymous SNV | NM_003741:c.T1124G:p.V375G | 621;Name=lod=446 |  |  |  |  |  | 0.14 | 0.001 | 0.823815 | 0.023758 | 0.997062 | chr3 | 184100862 | 184100862 | T | G | het | . | . | . | 26 | 0 | 0% | T | 13 | 3 | 18.75% | K | Somatic | 0.0487805 |
|  | exonic | C6orf174 | synonymous SNV | NM_001012279:c.T51C:p.S17S |  |  |  |  |  |  |  |  |  |  |  | chr6 | 127837709 | 127837709 | A | G | het | . | . | . | 47 | 0 | 0% | A | 13 | 3 | 18.75% | R | Somatic | 0.0141019 |
|  | exonic | C7orf52 | nonsynonymous SNV | NM_198571:c.T356G:p.V119G | 481;Name=lod=120 |  |  |  |  |  | 0.05 | 0.064 | 0.236032 | 0.006284 | 0.967387 | chr7 | 100816758 | 100816758 | A | C | het | . | . | . | 42 | 0 | 0% | A | 13 | 3 | 18.75% | M | Somatic | 0.0181488 |
|  | exonic | PPP1R26 | nonsynonymous SNV | NM_014811:c.G2237C:p.G746A |  | 0.9 |  |  |  |  | 0.33 | 0.016 | 0.943249 | 0.003923 | 0.663402 | chr9 | 138378593 | 138378593 | G | C | het | . | . | . | 36 | 0 | 0% | G | 13 | 3 | 18.75% | S | Somatic | 0.0253394 |
|  | intronic | GRIN1 |  |  |  |  |  |  |  |  |  |  |  |  |  | chr9 | 140055722 | 140055722 | A | C | het | . | . | . | 59 | 1 | 1.67% | A | 13 | 3 | 18.75% | M | Somatic | 0.0276077 |
|  | exonic | MED16 | synonymous SNV | NM_005481:c.G1335C:p.G445G | 395;Name=lod=54 |  |  |  |  |  |  |  |  |  |  | chr19 | 879955 | 879955 | C | G | het | . | . | . | 50 | 1 | 1.96% | C | 13 | 3 | 18.75% | S | Somatic | 0.0396357 |
|  | intronic | ADAMTS10 |  |  |  |  |  |  |  |  |  |  |  |  |  | chr19 | 8651149 | 8651149 | T | G | het | . | . | . | 50 | 0 | 0% | T | 13 | 3 | 18.75% | K | Somatic | 0.0122378 |
|  | exonic | TFE3 | nonsynonymous SNV | NM_006521:c.G442T:p.A148S | 583;Name=lod=312 |  |  |  |  |  | 0.34 | 0.379439 | 0.975398 | 0.022445 | 0.996999 | chrX | 48896724 | 48896724 | C | A | het | . | . | . | 28 | 0 | 0% | C | 13 | 3 | 18.75% | M | Somatic | 0.0422833 |
|  | intergenic | PNMA6D(dist=96700),MAGEA1(dist=138752) |  |  |  | 1 |  |  |  |  | 0.01 |  |  |  |  | chrX | 152342770 | 152342770 | A | C | het | . | . | . | 35 | 0 | 0% | A | 13 | 3 | 18.75% | M | Somatic | 0.0268908 |
|  | intergenic | MIR4436A(dist=887441),LOC654342(dist=1825300) |  |  |  | 0.99 |  |  |  |  |  |  |  |  |  | chr2 | 89999409 | 89999409 | C | G | het | . | . | . | 161 | 0 | 0% | C | 256 | 59 | 18.73% | S | Somatic | 3.39E-12 |
|  | intronic | EXOG |  |  |  |  |  |  |  |  |  |  |  |  |  | chr3 | 38539096 | 38539096 | T | C | het | . | . | . | 31 | 0 | 0% | T | 22 | 5 | 18.52% | Y | Somatic | 0.0176185 |
|  | ncRNA_intronic | FAM7A1,FAM7A2,FAM7A3 |  |  | 428;Name=lod=73 | 1 |  |  |  | rs77132839 |  |  |  |  |  | chr15 | 32716918 | 32716918 | A | T | het | . | . | . | 63 | 1 | 1.56% | A | 71 | 16 | 18.39% | W | Somatic | 6.64E-04 |
|  | intronic | UCKL1 |  |  |  |  |  |  |  |  |  |  |  |  |  | chr20 | 62576980 | 62576980 | T | G | het | . | . | . | 95 | 0 | 0% | T | 40 | 9 | 18.37% | K | Somatic | 3.61E-05 |
|  | exonic | ZSWIM5 | nonsynonymous SNV | NM_020883:c.G170C:p.R57P | 742;Name=lod=1387 |  |  |  |  |  | 0.13 | 0.938 | 0.989161 | 0.016843 | 0.989585 | chr1 | 45671853 | 45671853 | C | G | het | . | . | . | 43 | 0 | 0% | C | 9 | 2 | 18.18% | S | Somatic | 0.0384347 |
|  | exonic | PLXND1 | nonsynonymous SNV | NM_015103:c.C654G:p.S218R | 697;Name=lod=911 |  |  |  |  |  | 0.04 | 0.849 | 0.949769 | 0.771293 | 0.598794 | chr3 | 129324829 | 129324829 | G | C | het | . | . | . | 37 | 0 | 0% | G | 9 | 2 | 18.18% | S | Somatic | 0.0487589 |
|  | splicing | IDUA(NM_000203:exon2:c.159-1G>C) |  |  | 395;Name=lod=54 |  |  |  |  |  |  |  |  |  |  | chr4 | 981596 | 981596 | G | C | het | . | . | . | 49 | 0 | 0% | G | 9 | 2 | 18.18% | S | Somatic | 0.0310734 |
|  | intronic | AGPAT5 |  |  |  |  |  |  |  |  |  |  |  |  |  | chr8 | 6566416 | 6566416 | G | A | het | . | . | . | 41 | 0 | 0% | G | 9 | 2 | 18.18% | R | Somatic | 0.0414781 |
|  | exonic | KIAA0368 | nonsynonymous SNV | NM_001080398:c.A197G:p.E66G | 538;Name=lod=204 |  |  |  |  |  | 0.25 |  |  |  |  | chr9 | 114246716 | 114246716 | T | C | het | . | . | . | 50 | 0 | 0% | T | 9 | 2 | 18.18% | Y | Somatic | 0.0300546 |
|  | exonic | DUX2 | nonsynonymous SNV | NM_012147:c.T1076G:p.V359G |  | 1 |  |  |  |  |  |  |  |  |  | chr10 | 135494775 | 135494775 | T | G | het | . | . | . | 37 | 0 | 0% | T | 9 | 2 | 18.18% | K | Somatic | 0.0487589 |
|  | exonic | C15orf52 | nonsynonymous SNV | NM_207380:c.T712G:p.W238G | 428;Name=lod=73 |  |  |  |  |  | 0 | 0.999 | 0.996436 | 0.819736 | 0.998612 | chr15 | 40630028 | 40630028 | A | C | het | . | . | . | 37 | 0 | 0% | A | 9 | 2 | 18.18% | M | Somatic | 0.0487589 |
|  | exonic | PDIA2 | nonsynonymous SNV | NM_006849:c.T521G:p.V174G |  |  |  |  |  |  | 0 | 0.753 | 0.991854 | 0.830852 | 0.959211 | chr16 | 334773 | 334773 | T | G | het | . | . | . | 39 | 0 | 0% | T | 9 | 2 | 18.18% | K | Somatic | 0.044898 |
|  | exonic | GUCY2D | synonymous SNV | NM_000180:c.G708A:p.G236G |  |  |  |  |  |  |  |  |  |  |  | chr17 | 7907073 | 7907073 | G | A | het | . | . | . | 41 | 0 | 0% | G | 9 | 2 | 18.18% | R | Somatic | 0.0414781 |
|  | exonic | FAM134C | nonsynonymous SNV | NM_178126:c.T182G:p.V61G | 689;Name=lod=839 |  |  |  |  |  |  | 0.984 | 0.998839 | 0.994375 | 0.999999 | chr17 | 40761161 | 40761161 | A | C | het | . | . | . | 39 | 0 | 0% | A | 9 | 2 | 18.18% | M | Somatic | 0.044898 |
|  | exonic | HMHA1 | nonsynonymous SNV | NM_012292:c.T2756G:p.V919G | 524;Name=lod=180 |  |  |  |  |  | 0.45 | 0.022 | 0.915283 | 0.013947 | 0.985396 | chr19 | 1083153 | 1083153 | T | G | het | . | . | . | 38 | 0 | 0% | T | 9 | 2 | 18.18% | K | Somatic | 0.0467687 |
|  | exonic | HMHA1 | nonsynonymous SNV | NM_012292:c.C2827A:p.P943T | 361;Name=lod=39 |  |  |  |  |  | 0.02 | 0.841 | 0.994044 | 0.951457 | 1 | chr19 | 1083224 | 1083224 | C | A | het | . | . | . | 40 | 0 | 0% | C | 9 | 2 | 18.18% | M | Somatic | 0.0431373 |
|  | intergenic | ZNF813(dist=9367),ZNF331(dist=17264) |  |  |  | 0.94 |  |  |  | rs59572887 |  |  |  |  |  | chr19 | 54006913 | 54006913 | T | A | het | . | . | . | 24 | 0 | 0% | T | 36 | 8 | 18.18% | W | Somatic | 0.0239762 |
|  | exonic | SRC | nonsynonymous SNV | NM_005417:c.T851G:p.V284G | 642;Name=lod=540 |  |  |  |  |  | 0 | 0.998 | 0.995841 | 1 | 1 | chr20 | 36026249 | 36026249 | T | G | het | . | . | . | 37 | 0 | 0% | T | 9 | 2 | 18.18% | K | Somatic | 0.0487589 |
|  | intronic | PDXK |  |  |  |  |  |  |  |  |  |  |  |  |  | chr21 | 45175822 | 45175822 | T | C | het | . | . | . | 38 | 0 | 0% | T | 9 | 2 | 18.18% | Y | Somatic | 0.0467687 |
|  | intronic | PLXNB2 |  |  |  |  |  |  |  |  |  |  |  |  |  | chr22 | 50716513 | 50716513 | C | T | het | . | . | . | 38 | 0 | 0% | C | 9 | 2 | 18.18% | Y | Somatic | 0.0467687 |
|  | exonic | RREB1 | nonsynonymous SNV | NM_001003698:c.T4355G:p.V1452G |  |  |  |  |  |  | 0.32 | 0 | 0.058072 | 0 | 0.581943 | chr6 | 7247203 | 7247203 | T | G | het | . | . | . | 66 | 1 | 1.49% | T | 23 | 5 | 17.86% | K | Somatic | 0.0080099 |
|  | intronic | POLR2J |  |  |  | 0.99 |  |  |  |  |  |  |  |  |  | chr7 | 102119224 | 102119224 | A | C | het | . | . | . | 59 | 1 | 1.67% | A | 14 | 3 | 17.65% | M | Somatic | 0.0319078 |
|  | intronic | MCPH1 |  |  |  |  |  |  |  |  |  |  |  |  |  | chr8 | 6264217 | 6264217 | T | G | het | . | . | . | 56 | 0 | 0% | T | 14 | 3 | 17.65% | K | Somatic | 0.0109332 |
|  | exonic | SCYL1 | nonsynonymous SNV | NM_001048218:c.T2333G:p.V778G | 496;Name=lod=138 |  |  |  |  |  | 0 | 0.001 | 0.130909 | 7.60E-05 | 0.999803 | chr11 | 65305994 | 65305994 | T | G | het | . | . | . | 36 | 0 | 0% | T | 14 | 3 | 17.65% | K | Somatic | 0.0290276 |
|  | intronic | PKD1 |  |  |  | 0.98 |  |  |  |  |  |  |  |  |  | chr16 | 2147997 | 2147997 | G | C | het | . | . | . | 42 | 0 | 0% | G | 14 | 3 | 17.65% | S | Somatic | 0.0209173 |
|  | exonic | ZNF768 | nonsynonymous SNV | NM_024671:c.A1348C:p.T450P | 562;Name=lod=257 |  |  |  |  |  |  | 0.519 | 0.996336 | 0.484361 | 0.991794 | chr16 | 30536113 | 30536113 | T | G | het | . | . | . | 57 | 1 | 1.72% | T | 14 | 3 | 17.65% | K | Somatic | 0.034407 |
|  | intergenic | ANKRD62P1-PARP4P3(dist=16403),XKR3(dist=91473) |  |  |  | 1 |  |  |  |  |  |  |  |  |  | chr22 | 17172833 | 17172833 | G | T | het | . | . | . | 49 | 1 | 2% | G | 56 | 12 | 17.65% | K | Somatic | 0.0056848 |
|  | intronic | MATN1 |  |  |  |  |  |  |  |  |  |  |  |  |  | chr1 | 31189600 | 31189600 | A | C | het | . | . | . | 27 | 0 | 0% | A | 33 | 7 | 17.50% | M | Somatic | 0.021438 |
|  | intronic | SLC1A5 |  |  |  |  |  |  |  |  |  |  |  |  |  | chr19 | 47285618 | 47285618 | A | C | het | . | . | . | 21 | 0 | 0% | A | 33 | 7 | 17.50% | M | Somatic | 0.0427339 |
|  | intronic | SLC12A4 |  |  |  |  |  |  |  |  |  |  |  |  |  | chr16 | 67980269 | 67980269 | A | C | het | . | . | . | 55 | 1 | 1.79% | A | 19 | 4 | 17.39% | M | Somatic | 0.0234954 |
|  | exonic | EPN2 | nonsynonymous SNV | NM_001102664:c.A800C:p.N267T | 548;Name=lod=224 |  |  |  |  |  | 0 | 0.961 | 0.998473 | 0.99847 | 1 | chr17 | 19237296 | 19237296 | A | C | het | . | . | . | 30 | 0 | 0% | A | 19 | 4 | 17.39% | M | Somatic | 0.0302399 |
|  | exonic | TSEN54 | nonsynonymous SNV | NM_207346:c.A814G:p.R272G |  |  |  |  |  |  | 0.06 | 0.594 | 0.886768 | 0.301993 | 0.701592 | chr17 | 73517976 | 73517976 | A | G | het | . | . | . | 37 | 0 | 0% | A | 19 | 4 | 17.39% | R | Somatic | 0.0181591 |
|  | intergenic | MIR4436A(dist=409361),NONE(dist=NONE) |  |  |  | 0.99 |  |  |  |  |  |  |  |  |  | chr2 | 89521329 | 89521329 | C | T | het | . | . | . | 135 | 1 | 0.74% | C | 288 | 60 | 17.24% | Y | Somatic | 1.05E-08 |
|  | intronic | C20orf27 |  |  |  |  |  |  |  |  |  |  |  |  |  | chr20 | 3735199 | 3735199 | T | C | het | . | . | . | 66 | 1 | 1.49% | T | 39 | 8 | 17.02% | Y | Somatic | 0.0034607 |
|  | intergenic | MIR4436A(dist=409360),NONE(dist=NONE) |  |  |  | 0.99 |  |  |  |  | 0.07 |  |  |  |  | chr2 | 89521328 | 89521328 | G | C | het | . | . | . | 135 | 0 | 0% | G | 286 | 58 | 16.86% | S | Somatic | 1.02E-09 |
|  | exonic | SKI | nonsynonymous SNV | NM_003036:c.T1318C:p.S440P |  |  |  |  |  |  | 0.36 | 0 | 0.83367 | 0.19732 | 0.930114 | chr1 | 2235385 | 2235385 | T | C | het | . | . | . | 43 | 0 | 0% | T | 10 | 2 | 16.67% | Y | Somatic | 0.0444444 |
|  | intronic | CHD5 |  |  |  |  |  |  |  |  |  |  |  |  |  | chr1 | 6191853 | 6191853 | G | C | het | . | . | . | 26 | 0 | 0% | G | 20 | 4 | 16.67% | S | Somatic | 0.0461398 |
|  | intronic | COL16A1 |  |  |  |  |  |  |  |  |  |  |  |  |  | chr1 | 32140567 | 32140567 | A | C | het | . | . | . | 25 | 0 | 0% | A | 25 | 5 | 16.67% | M | Somatic | 0.0409646 |
|  | intronic | PDE4DIP |  |  |  | 0.95 |  |  |  |  |  |  |  |  |  | chr1 | 144906012 | 144906012 | C | A | het | . | . | . | 51 | 1 | 1.92% | C | 40 | 8 | 16.67% | M | Somatic | 0.011197 |
|  | UTR5 | RPS6KC1 |  |  | 624;Name=lod=457 |  |  |  |  |  |  |  |  |  |  | chr1 | 213224744 | 213224744 | A | G | het | . | . | . | 41 | 0 | 0% | A | 15 | 3 | 16.67% | R | Somatic | 0.0251007 |
|  | ncRNA_exonic | MIR663B |  |  |  | 0.91 |  |  |  |  |  |  |  |  |  | chr2 | 133014616 | 133014616 | C | T | het | . | . | . | 47 | 0 | 0% | C | 15 | 3 | 16.67% | Y | Somatic | 0.0186813 |
|  | splicing | AQP12B(NM_001102467:exon3:c.724+2T>G) |  |  | 455;Name=lod=94 | 0.99 |  |  |  |  |  |  |  |  |  | chr2 | 241619767 | 241619767 | A | C | het | . | . | . | 30 | 0 | 0% | A | 15 | 3 | 16.67% | M | Somatic | 0.0471785 |
|  | exonic | MST1 | synonymous SNV | NM_020998:c.T939C:p.T313T |  | 0.98 | 0.07 | 0.07 | 0.07 | rs7431215 |  |  |  |  |  | chr3 | 49723823 | 49723823 | A | G | het | . | . | . | 39 | 0 | 0% | A | 20 | 4 | 16.67% | R | Somatic | 0.0178389 |
|  | exonic | SCAMP1 | nonsynonymous SNV | NM_004866:c.C259A:p.L87I | 662;Name=lod=656 |  |  |  |  |  | 0 |  |  |  |  | chr5 | 77712389 | 77712389 | C | A | het | . | . | . | 48 | 0 | 0% | C | 10 | 2 | 16.67% | M | Somatic | 0.0372881 |
|  | exonic | SYNJ2 | nonsynonymous SNV | NM_001178088:c.A1678C:p.T560P | 529;Name=lod=189 |  |  |  |  |  | 0.01 | 0.059 | 0.998282 | 0.259544 | 0.999992 | chr6 | 158497754 | 158497754 | A | C | het | . | . | . | 56 | 1 | 1.75% | A | 75 | 15 | 16.67% | M | Somatic | 0.002899 |
|  | intronic | VAV2 |  |  |  |  |  |  |  |  |  |  |  |  |  | chr9 | 136652340 | 136652340 | T | G | het | . | . | . | 33 | 0 | 0% | T | 20 | 4 | 16.67% | K | Somatic | 0.0269006 |
|  | exonic | ZFP36L1 | nonsynonymous SNV | NM_001244701:c.A46C:p.T16P |  |  |  |  |  |  |  | 0.981 | 0.005847 | 0.002388 | 0.613058 | chr14 | 69262821 | 69262821 | T | G | het | . | . | . | 47 | 0 | 0% | T | 20 | 4 | 16.67% | K | Somatic | 0.0109362 |
|  | intronic | SOLH |  |  |  |  |  |  |  |  |  |  |  |  |  | chr16 | 601701 | 601701 | G | C | het | . | . | . | 49 | 0 | 0% | G | 15 | 3 | 16.67% | S | Somatic | 0.0170337 |
|  | exonic | VASN | nonsynonymous SNV | NM_138440:c.T284G:p.V95G | 387;Name=lod=50 |  |  |  |  |  | 0 | 0.961 | 0.998 | 0.943522 | 0.999952 | chr16 | 4431162 | 4431162 | T | G | het | . | . | . | 51 | 0 | 0% | T | 10 | 2 | 16.67% | K | Somatic | 0.0337942 |
|  | exonic;splicing | MYO1C;MYO1C | nonsynonymous SNV | NM_001080779:c.G546T:p.E182D | 467;Name=lod=105 |  |  |  |  |  | 0 | 0.989 | 0.998437 | 0.999968 | 1 | chr17 | 1386155 | 1386155 | C | A | het | . | . | . | 62 | 0 | 0% | C | 10 | 2 | 16.67% | M | Somatic | 0.0244354 |
|  | exonic | MYO18A | nonsynonymous SNV | NM_078471:c.T5083G:p.S1695A | 548;Name=lod=225 |  |  |  |  |  | 0.39 |  |  |  |  | chr17 | 27419465 | 27419465 | A | C | het | . | . | . | 27 | 0 | 0% | A | 20 | 4 | 16.67% | M | Somatic | 0.042521 |
|  | intronic | LGALS3BP |  |  |  |  |  |  |  |  |  |  |  |  |  | chr17 | 76970686 | 76970686 | T | G | het | . | . | . | 36 | 0 | 0% | T | 15 | 3 | 16.67% | K | Somatic | 0.0328979 |
|  | exonic | PRR12 | synonymous SNV | NM_020719:c.A696C:p.P232P | 703;Name=lod=962 |  |  |  |  |  |  |  |  |  |  | chr19 | 50098288 | 50098288 | A | C | het | . | . | . | 44 | 0 | 0% | A | 10 | 2 | 16.67% | M | Somatic | 0.0428571 |
|  | exonic | MYH7B | nonsynonymous SNV | NM_020884:c.T3785G:p.V1262G | 590;Name=lod=332 |  |  |  |  |  | 0 | 0.439099 | 0.954399 | 0.057295 | 0.945963 | chr20 | 33585355 | 33585355 | T | G | het | . | . | . | 35 | 0 | 0% | T | 25 | 5 | 16.67% | K | Somatic | 0.0172528 |
|  | intronic | CNKSR2 |  |  |  |  |  |  |  |  |  |  |  |  |  | chrX | 21666938 | 21666938 | G | T | het | . | . | . | 43 | 0 | 0% | G | 30 | 6 | 16.67% | K | Somatic | 0.0070074 |
|  | intergenic | FOXD4L4(dist=31384),AQP7P3(dist=106426) |  |  |  | 1 |  |  |  | rs3894117 |  |  |  |  |  | chr9 | 42751726 | 42751726 | T | C | het | . | . | . | 55 | 1 | 1.79% | T | 66 | 13 | 16.46% | Y | Somatic | 0.0042027 |
|  | exonic | PSD2 | nonsynonymous SNV | NM_032289:c.A1951C:p.T651P | 579;Name=lod=301 |  |  |  |  |  | 0.21 | 0.001 | 0.906466 | 0.134431 | 0.999926 | chr5 | 139218340 | 139218340 | A | C | het | . | . | . | 33 | 0 | 0% | A | 41 | 8 | 16.33% | M | Somatic | 0.0126532 |
|  | exonic | PLCXD1 | nonsynonymous SNV | NM_018390:c.A425G:p.E142G | 325;Name=lod=28 | 1 |  |  |  |  | 0.01 |  |  |  |  | chrX | 208197 | 208197 | A | G | het | . | . | . | 46 | 0 | 0% | A | 31 | 6 | 16.22% | R | Somatic | 0.0061592 |
|  | exonic | TIGD5 | nonsynonymous SNV | NM_032862:c.T1847G:p.V616G | 426;Name=lod=72 |  |  |  |  |  | 0.02 | 0.992 | 0.861971 | 0.003036 | 0.865551 | chr8 | 144681920 | 144681920 | T | G | het | . | . | . | 51 | 1 | 1.92% | T | 21 | 4 | 16% | K | Somatic | 0.0359822 |
|  | intronic | MMP21 |  |  |  |  |  |  |  |  |  |  |  |  |  | chr10 | 127464192 | 127464192 | G | T | het | . | . | . | 51 | 0 | 0% | G | 21 | 4 | 16% | K | Somatic | 0.0098599 |
|  | exonic | SPHK2 | nonsynonymous SNV | NM_001204160:c.A1096C:p.T366P | 352;Name=lod=36 |  |  |  |  |  | 0.24 | 0.243 | 0.880884 | 0.003491 | 0.790555 | chr19 | 49132269 | 49132269 | A | C | het | . | . | . | 82 | 1 | 1.20% | A | 21 | 4 | 16% | M | Somatic | 0.0098958 |
|  | intergenic | ZNF813(dist=9369),ZNF331(dist=17262) |  |  |  | 0.94 |  |  |  | rs57872010 |  |  |  |  |  | chr19 | 54006915 | 54006915 | T | G | het | . | . | . | 26 | 0 | 0% | T | 37 | 7 | 15.91% | K | Somatic | 0.0319664 |
|  | ncRNA_exonic | MIR663B |  |  |  | 0.91 |  |  |  |  |  |  |  |  |  | chr2 | 133014603 | 133014603 | G | T | het | . | . | . | 54 | 1 | 1.82% | G | 16 | 3 | 15.79% | K | Somatic | 0.0496869 |
|  | intronic | EBF1 |  |  |  |  |  |  |  |  |  |  |  |  |  | chr5 | 158523960 | 158523960 | C | G | het | . | . | . | 36 | 0 | 0% | C | 16 | 3 | 15.79% | S | Somatic | 0.0369354 |
|  | exonic | ADCY1 | nonsynonymous SNV | NM_021116:c.T572G:p.V191G | 627;Name=lod=470 |  |  |  |  |  |  | 0.847 | 0.991251 | 0.876946 | 0.999999 | chr7 | 45614714 | 45614714 | T | G | het | . | . | . | 60 | 0 | 0% | T | 16 | 3 | 15.79% | K | Somatic | 0.0122536 |
|  | exonic | DEGS2 | nonsynonymous SNV | NM_206918:c.T182G:p.V61G | 483;Name=lod=122 |  |  |  |  |  | 0 | 0.338 | 0.863237 | 0.999908 | 0.999987 | chr14 | 100615948 | 100615948 | A | C | het | . | . | . | 35 | 0 | 0% | A | 16 | 3 | 15.79% | M | Somatic | 0.0390663 |
|  | intronic | PCSK4 |  |  |  |  |  |  |  |  | 0 |  |  |  |  | chr19 | 1487127 | 1487127 | G | T | het | . | . | . | 45 | 0 | 0% | G | 16 | 3 | 15.79% | K | Somatic | 0.0232575 |
|  | exonic | ZBTB45 | synonymous SNV | NM_032792:c.A1071C:p.P357P |  | 0.91 |  |  |  |  |  |  |  |  |  | chr19 | 59027970 | 59027970 | T | G | het | . | . | . | 44 | 0 | 0% | T | 16 | 3 | 15.79% | K | Somatic | 0.0244013 |
|  | intergenic | MIR4436A(dist=887548),LOC654342(dist=1825193) |  |  | 513;Name=lod=162 | 0.99 |  |  |  |  |  |  |  |  |  | chr2 | 89999516 | 89999516 | G | A | het | . | . | . | 225 | 1 | 0.44% | G | 338 | 62 | 15.50% | R | Somatic | 3.49E-12 |
|  | exonic | BAI2 | nonsynonymous SNV | NM_001703:c.T2308C:p.S770P | 550;Name=lod=230 |  |  |  |  |  | 0.11 | 0.007 | 0.861014 | 0.076903 | 0.837693 | chr1 | 32205096 | 32205096 | A | G | het | . | . | . | 58 | 0 | 0% | A | 22 | 4 | 15.38% | R | Somatic | 0.0077481 |
|  | exonic | COL4A4 | nonsynonymous SNV | NM_000092:c.G365A:p.G122D | 544;Name=lod=216 |  |  |  |  |  | 0 | 0.997 | 0.028443 | 0.99806 | 0.75887 | chr2 | 227984618 | 227984618 | C | T | het | . | . | . | 45 | 0 | 0% | C | 11 | 2 | 15.38% | Y | Somatic | 0.0471869 |
|  | exonic | PPP2CB | synonymous SNV | NM_001009552:c.G45A:p.E15E | 611;Name=lod=404 |  |  |  |  |  |  |  |  |  |  | chr8 | 30669893 | 30669893 | C | T | het | . | . | . | 63 | 0 | 0% | C | 11 | 2 | 15.38% | Y | Somatic | 0.0273684 |
|  | exonic | EPPK1 | nonsynonymous SNV | NM_031308:c.C1325T:p.T442M | 289;Name=lod=20 |  |  |  |  |  |  |  |  |  |  | chr8 | 144946097 | 144946097 | G | A | het | . | . | . | 50 | 0 | 0% | G | 11 | 2 | 15.38% | R | Somatic | 0.0399386 |
|  | exonic | OPLAH | nonsynonymous SNV | NM_017570:c.A3715C:p.T1239P | 355;Name=lod=37 |  |  |  |  |  | 0.64 |  |  |  |  | chr8 | 145106637 | 145106637 | T | G | het | . | . | . | 44 | 0 | 0% | T | 11 | 2 | 15.38% | K | Somatic | 0.0488722 |
|  | exonic | PPP1R16A | nonsynonymous SNV | NM_032902:c.C1064A:p.T355N | 566;Name=lod=267 |  |  |  |  |  | 0.23 | 0.001 | 0.959824 | 0.216492 | 0.997484 | chr8 | 145726538 | 145726538 | C | A | het | . | . | . | 70 | 0 | 0% | C | 11 | 2 | 15.38% | M | Somatic | 0.022921 |
|  | intronic | FBXW5 |  |  |  |  |  |  |  |  |  |  |  |  |  | chr9 | 139837173 | 139837173 | G | A | het | . | . | . | 75 | 0 | 0% | G | 11 | 2 | 15.38% | R | Somatic | 0.0203762 |
|  | exonic | SFRP5 | nonsynonymous SNV | NM_003015:c.G234T:p.E78D | 770;Name=lod=1792 |  |  |  |  |  | 0.44 | 0.008 | 0.957443 | 0.807466 | 0.99992 | chr10 | 99531357 | 99531357 | C | A | het | . | . | . | 47 | 0 | 0% | C | 11 | 2 | 15.38% | M | Somatic | 0.0440678 |
|  | exonic | EMX2 | nonsynonymous SNV | NM_001165924:c.A230C:p.H77P | 781;Name=lod=1985 |  |  |  |  |  | 0.11 | 0.999 | 0.984189 | 0.754868 | 1 | chr10 | 119303008 | 119303008 | A | C | het | . | . | . | 44 | 0 | 0% | A | 11 | 2 | 15.38% | M | Somatic | 0.0488722 |
|  | exonic | IRS2 | nonsynonymous SNV | NM_003749:c.T833G:p.V278G | 666;Name=lod=679 |  |  |  |  |  | 0 | 0.995 | 0.993975 | 0.011412 | 0.999998 | chr13 | 110437568 | 110437568 | A | C | het | . | . | . | 54 | 0 | 0% | A | 11 | 2 | 15.38% | M | Somatic | 0.0352782 |
|  | UTR3 | ZNF219 |  |  | 545;Name=lod=219 |  |  |  |  |  |  |  |  |  |  | chr14 | 21558643 | 21558643 | T | C | het | . | . | . | 60 | 0 | 0% | T | 22 | 4 | 15.38% | Y | Somatic | 0.0070401 |
|  | exonic | TMEM30B | nonsynonymous SNV | NM_001017970:c.A623C:p.N208T | 607;Name=lod=390 |  |  |  |  | rs76886675 | 0 | 0.934 | 0.997195 | 0.992387 | 1 | chr14 | 61747243 | 61747243 | T | G | het | . | . | . | 69 | 0 | 0% | T | 11 | 2 | 15.38% | K | Somatic | 0.0234869 |
|  | UTR5 | CINP |  |  |  |  |  |  |  |  |  |  |  |  |  | chr14 | 102829232 | 102829232 | A | C | het | . | . | . | 62 | 1 | 1.59% | A | 22 | 4 | 15.38% | M | Somatic | 0.0242758 |
|  | intergenic | TMEM121(dist=177580),KIAA0125(dist=209719) |  |  | 265;Name=lod=16 | 0.97 |  |  |  |  |  |  |  |  |  | chr14 | 106174119 | 106174119 | C | T | het | . | . | . | 60 | 0 | 0% | C | 44 | 8 | 15.38% | Y | Somatic | 0.0015827 |
|  | intergenic | XYLT1(dist=873033),MIR3180-1(dist=58264) |  |  | 607;Name=lod=392 | 0.99 |  |  |  |  |  |  |  |  |  | chr16 | 18437771 | 18437771 | G | C | het | . | . | . | 54 | 0 | 0% | G | 11 | 2 | 15.38% | S | Somatic | 0.0352782 |
|  | exonic | NKD1 | nonsynonymous SNV | NM_033119:c.G170A:p.G57D | 455;Name=lod=94 |  |  |  |  |  | 0.27 | 0.009 | 0.999018 | 0.04247 | 0.993531 | chr16 | 50583444 | 50583444 | G | A | het | . | . | . | 46 | 0 | 0% | G | 11 | 2 | 15.38% | R | Somatic | 0.0455874 |
|  | exonic | TMEM132E | nonsynonymous SNV | NM_207313:c.A337C:p.T113P | 594;Name=lod=346 |  |  |  |  |  | 0.05 | 0 | 0.997453 | 0.986894 | 0.999996 | chr17 | 32953415 | 32953415 | A | C | het | . | . | . | 46 | 0 | 0% | A | 11 | 2 | 15.38% | M | Somatic | 0.0455874 |
|  | exonic | PFKL | nonsynonymous SNV | NM_002626:c.T1349A:p.V450E | 412;Name=lod=63 |  |  |  |  |  | 0.02 | 0.065 | 0.988252 | 0.998576 | 0.999995 | chr21 | 45742026 | 45742026 | T | A | het | . | . | . | 60 | 0 | 0% | T | 11 | 2 | 15.38% | W | Somatic | 0.0296804 |
|  | exonic | IGDCC4 | nonsynonymous SNV | NM_020962:c.A232C:p.T78P |  |  |  |  |  |  | 0.26 | 0 | 0.00113 | 0.030188 | 0.832791 | chr15 | 65703547 | 65703547 | T | G | het | . | . | . | 32 | 0 | 0% | T | 50 | 9 | 15.25% | K | Somatic | 0.0160324 |
|  | exonic | TRPA1 | nonsynonymous SNV | NM_007332:c.T1576G:p.Y526D | 602;Name=lod=372 |  |  |  |  |  | 0 | 0.998 | 0.998164 | 0.968959 | 1 | chr8 | 72966056 | 72966056 | A | C | het | . | . | . | 52 | 1 | 1.89% | A | 34 | 6 | 15% | M | Somatic | 0.0234417 |
|  | exonic | LINGO1 | synonymous SNV | NM_032808:c.A1089C:p.T363T | 585;Name=lod=319 |  |  |  |  |  |  |  |  |  |  | chr15 | 77907160 | 77907160 | T | G | het | . | . | . | 62 | 0 | 0% | T | 17 | 3 | 15% | K | Somatic | 0.0128726 |
|  | exonic | PKMYT1 | synonymous SNV | NM_004203:c.A1251C:p.P417P |  |  |  |  |  |  |  |  |  |  |  | chr16 | 3024060 | 3024060 | T | G | het | . | . | . | 54 | 0 | 0% | T | 17 | 3 | 15% | K | Somatic | 0.0175861 |
|  | intronic | MEIS3 |  |  | 655;Name=lod=614 |  |  |  |  |  | 0 |  |  |  |  | chr19 | 47918144 | 47918144 | T | G | het | . | . | . | 33 | 0 | 0% | T | 17 | 3 | 15% | K | Somatic | 0.0486639 |
|  | exonic | CDC42EP1 | nonsynonymous SNV | NM_152243:c.A119C:p.H40P | 504;Name=lod=149 |  |  |  |  |  | 1 | 0 | 0.974416 | 2.51E-04 | 0.999997 | chr22 | 37962475 | 37962475 | A | C | het | . | . | . | 41 | 0 | 0% | A | 17 | 3 | 15% | M | Somatic | 0.0316755 |
|  | exonic | SELO | nonsynonymous SNV | NM_031454:c.A1883C:p.H628P |  |  |  |  |  |  | 0.22 | 0.63 | 0.076203 | 0.995639 | 0.047134 | chr22 | 50655671 | 50655671 | A | C | het | . | . | . | 88 | 1 | 1.12% | A | 17 | 3 | 15% | M | Somatic | 0.0191084 |
|  | exonic | SF3B3 | synonymous SNV | NM_012426:c.C375A:p.P125P | 685;Name=lod=808 |  |  |  |  |  |  |  |  |  |  | chr16 | 70563080 | 70563080 | C | A | het | . | . | . | 51 | 1 | 1.92% | C | 57 | 10 | 14.93% | M | Somatic | 0.0134506 |
|  | UTR3 | TMEM184B |  |  |  |  |  |  |  |  |  |  |  |  |  | chr22 | 38617419 | 38617419 | T | G | het | . | . | . | 52 | 1 | 1.89% | T | 23 | 4 | 14.81% | K | Somatic | 0.0420499 |
|  | UTR5 | TFB1M |  |  |  |  |  |  |  |  |  |  |  |  |  | chr6 | 155635602 | 155635602 | A | C | het | . | . | . | 46 | 0 | 0% | A | 29 | 5 | 14.71% | M | Somatic | 0.0115747 |
|  | exonic | SEZ6L | synonymous SNV | NM_001184773:c.A267C:p.A89A |  |  |  |  |  |  |  |  |  |  |  | chr22 | 26688544 | 26688544 | A | C | het | . | . | . | 34 | 0 | 0% | A | 29 | 5 | 14.71% | M | Somatic | 0.0266935 |
|  | intronic | COPG2 |  |  |  |  |  |  |  |  |  |  |  |  |  | chr7 | 130148881 | 130148881 | C | A | het | . | . | . | 26 | 0 | 0% | C | 41 | 7 | 14.58% | M | Somatic | 0.0409146 |
|  | exonic | SPG11 | nonsynonymous SNV | NM_001160227:c.G6611C:p.G2204A |  |  |  |  |  | rs79186522 | 0.34 | 0.006 | 0.95023 | 0.07104 | 0.92384 | chr15 | 44858101 | 44858101 | C | G | het | . | . | . | 32 | 0 | 0% | C | 41 | 7 | 14.58% | S | Somatic | 0.0231777 |
|  | exonic | PPP2R1B | synonymous SNV | NM_001177562:c.T45G:p.G15G |  |  |  |  |  |  |  |  |  |  |  | chr11 | 111637041 | 111637041 | A | C | het | . | . | . | 128 | 1 | 0.78% | A | 47 | 8 | 14.55% | M | Somatic | 2.99E-04 |
|  | exonic | ARHGAP6 | synonymous SNV | NM_006125:c.T591G:p.G197G | 527;Name=lod=184 |  |  |  |  |  |  |  |  |  |  | chrX | 11272825 | 11272825 | A | C | het | . | . | . | 24 | 0 | 0% | A | 47 | 8 | 14.55% | M | Somatic | 0.0466701 |
|  | intronic | AGRN |  |  |  |  |  |  |  |  |  |  |  |  |  | chr1 | 978898 | 978898 | T | G | het | . | . | . | 71 | 0 | 0% | T | 12 | 2 | 14.29% | K | Somatic | 0.0254902 |
|  | ncRNA_intronic | CROCCP2 |  |  |  | 0.94 |  |  |  |  |  |  |  |  |  | chr1 | 16950339 | 16950339 | C | T | het | . | . | . | 49 | 0 | 0% | C | 12 | 2 | 14.29% | Y | Somatic | 0.046595 |
|  | intronic | C1orf173 |  |  | 343;Name=lod=33 |  |  |  |  |  |  |  |  |  |  | chr1 | 75139143 | 75139143 | A | C | het | . | . | . | 54 | 1 | 1.82% | A | 24 | 4 | 14.29% | M | Somatic | 0.0421708 |
|  | UTR3 | ACADM |  |  |  |  |  |  |  |  |  |  |  |  |  | chr1 | 76228470 | 76228470 | G | A | het | . | . | . | 77 | 1 | 1.28% | G | 18 | 3 | 14.29% | R | Somatic | 0.0291483 |
|  | exonic | GREB1 | nonsynonymous SNV | NM_014668:c.A1676G:p.D559G | 419;Name=lod=67 |  |  |  |  |  | 0 | 0.941 | 0.997601 | 0.244249 | 0.99944 | chr2 | 11733232 | 11733232 | A | G | het | . | . | . | 55 | 0 | 0% | A | 12 | 2 | 14.29% | R | Somatic | 0.0387894 |
|  | UTR5 | EPAS1 |  |  | 469;Name=lod=107 |  |  |  |  |  |  |  |  |  |  | chr2 | 46525046 | 46525046 | C | T | het | . | . | . | 50 | 0 | 0% | C | 12 | 2 | 14.29% | Y | Somatic | 0.0451389 |
|  | exonic | GLI2 | nonsynonymous SNV | NM_005270:c.A3611C:p.H1204P |  |  |  |  |  |  | 0.18 | 0.009 | 0.751704 | 0.273475 | 0.58634 | chr2 | 121747101 | 121747101 | A | C | het | . | . | . | 37 | 0 | 0% | A | 18 | 3 | 14.29% | M | Somatic | 0.0431034 |
|  | exonic | MAP3K2 | synonymous SNV | NM_006609:c.A1035C:p.P345P | 625;Name=lod=463 |  |  |  |  |  |  |  |  |  |  | chr2 | 128079632 | 128079632 | T | G | het | . | . | . | 49 | 1 | 2% | T | 30 | 5 | 14.29% | K | Somatic | 0.0408245 |
|  | exonic | AIM1 | nonsynonymous SNV | NM_001624:c.T176G:p.V59G | 363;Name=lod=40 |  |  |  |  |  | 0 | 0.995 | 0.913907 | 0.002532 | 0.994423 | chr6 | 106960392 | 106960392 | T | G | het | . | . | . | 49 | 0 | 0% | T | 12 | 2 | 14.29% | K | Somatic | 0.046595 |
|  | intronic | POR |  |  |  |  |  |  |  |  |  |  |  |  |  | chr7 | 75615473 | 75615473 | C | A | het | . | . | . | 61 | 0 | 0% | C | 12 | 2 | 14.29% | M | Somatic | 0.0327928 |
|  | intronic | EIF2C2 |  |  |  |  |  |  |  |  |  |  |  |  |  | chr8 | 141542478 | 141542478 | A | C | het | . | . | . | 31 | 0 | 0% | A | 30 | 5 | 14.29% | M | Somatic | 0.0363248 |
|  | exonic | BRD3 | synonymous SNV | NM_007371:c.G72C:p.P24P |  |  |  |  |  |  |  |  |  |  |  | chr9 | 136918528 | 136918528 | C | G | het | . | . | . | 68 | 1 | 1.45% | C | 36 | 6 | 14.29% | S | Somatic | 0.0114516 |
|  | exonic | PLCB3 | synonymous SNV | NM_001184883:c.T2829G:p.G943G |  |  |  |  |  |  |  |  |  |  |  | chr11 | 64032969 | 64032969 | T | G | het | . | . | . | 52 | 0 | 0% | T | 18 | 3 | 14.29% | K | Somatic | 0.021384 |
|  | exonic | HNF1A | nonsynonymous SNV | NM_000545:c.A1687C:p.T563P | 499;Name=lod=142 |  |  |  |  |  | 0.21 | 0 | 0.830578 | 0.07833 | 0.946344 | chr12 | 121437349 | 121437349 | A | C | het | . | . | . | 42 | 0 | 0% | A | 18 | 3 | 14.29% | M | Somatic | 0.033492 |
|  | intronic | SCARB1 |  |  |  |  |  |  |  |  |  |  |  |  |  | chr12 | 125299664 | 125299664 | T | G | het | lung | carcinoma | adenocarcinoma | 64 | 1 | 1.54% | T | 42 | 7 | 14.29% | K | Somatic | 0.0109651 |
|  | intergenic | LOC645752(dist=14694),LOC91450(dist=51693) |  |  |  | 0.95 | 0.006 | 0.006 | 0.006 | rs142683584 |  |  |  |  |  | chr15 | 78233882 | 78233882 | A | T | het | . | . | . | 39 | 0 | 0% | A | 24 | 4 | 14.29% | W | Somatic | 0.026713 |
|  | ncRNA_intronic | PKD1P1 |  |  |  | 0.99 |  |  |  | rs2914408 |  |  |  |  |  | chr16 | 16422171 | 16422171 | C | T | het | . | . | . | 69 | 1 | 1.43% | C | 18 | 3 | 14.29% | Y | Somatic | 0.0370734 |
|  | intronic | DNAH3 |  |  |  |  |  |  |  |  |  |  |  |  |  | chr16 | 20963705 | 20963705 | T | G | het | . | . | . | 33 | 0 | 0% | T | 30 | 5 | 14.29% | K | Somatic | 0.0311424 |
|  | exonic | SBK1 | nonsynonymous SNV | NM_001024401:c.G719A:p.G240D | 674;Name=lod=733 |  |  |  |  |  | 0 | 0.997 | 0.996938 | 0.999969 | 1 | chr16 | 28331686 | 28331686 | G | A | het | . | . | . | 73 | 0 | 0% | G | 12 | 2 | 14.29% | R | Somatic | 0.024325 |
|  | intronic | MVP |  |  |  |  |  |  |  |  |  |  |  |  |  | chr16 | 29853192 | 29853192 | T | G | het | . | . | . | 51 | 1 | 1.92% | T | 30 | 5 | 14.29% | K | Somatic | 0.036643 |
|  | intronic | SLC7A5 |  |  |  |  |  |  |  |  |  |  |  |  |  | chr16 | 87870073 | 87870073 | T | G | het | . | . | . | 48 | 0 | 0% | T | 12 | 2 | 14.29% | K | Somatic | 0.0481227 |
|  | exonic | ZC3H18 | nonsynonymous SNV | NM_144604:c.A1978C:p.T660P |  |  |  |  |  |  | 0.02 | 0.328895 | 0.013614 | 3.25E-04 | 0.802355 | chr16 | 88691089 | 88691089 | A | C | het | . | . | . | 59 | 0 | 0% | A | 12 | 2 | 14.29% | M | Somatic | 0.0346271 |
|  | exonic | DIRAS1 | synonymous SNV | NM_145173:c.C48G:p.G16G | 753;Name=lod=1525 |  |  |  |  |  |  |  |  |  |  | chr19 | 2717757 | 2717757 | G | C | het | . | . | . | 68 | 0 | 0% | G | 12 | 2 | 14.29% | S | Somatic | 0.0274014 |
|  | intronic | DOCK6 |  |  |  |  |  |  |  |  |  |  |  |  |  | chr19 | 11319547 | 11319547 | A | C | het | . | . | . | 40 | 0 | 0% | A | 18 | 3 | 14.29% | M | Somatic | 0.0369547 |
|  | intronic | TFAP2C |  |  | 272;Name=lod=17 |  |  |  |  |  |  |  |  |  |  | chr20 | 55204670 | 55204670 | C | T | het | . | . | . | 52 | 0 | 0% | C | 12 | 2 | 14.29% | Y | Somatic | 0.0424242 |
|  | exonic | COL9A3 | nonsynonymous SNV | NM_001853:c.C977T:p.A326V |  |  |  |  |  |  | 0.56 | 0.002 | 0.222828 | 3.60E-05 | 0.95638 | chr20 | 61460297 | 61460297 | C | T | het | . | . | . | 55 | 0 | 0% | C | 12 | 2 | 14.29% | Y | Somatic | 0.0387894 |
|  | intronic | RAB17 |  |  |  |  |  |  |  |  |  |  |  |  |  | chr2 | 238485885 | 238485885 | T | G | het | . | . | . | 60 | 1 | 1.64% | T | 67 | 11 | 14.10% | K | Somatic | 0.0077728 |
|  | intronic | REV1 |  |  |  |  |  |  |  |  |  |  |  |  |  | chr2 | 100058739 | 100058739 | C | A | het | . | . | . | 38 | 0 | 0% | C | 43 | 7 | 14% | M | Somatic | 0.0157339 |
|  | UTR3 | GARS |  |  |  |  |  |  |  |  |  |  |  |  |  | chr7 | 30673514 | 30673514 | A | T | het | . | . | . | 43 | 0 | 0% | A | 37 | 6 | 13.95% | W | Somatic | 0.0129669 |
|  | exonic | COMMD5 | nonsynonymous SNV | NM_001081003:c.T476G:p.V159G | 419;Name=lod=67 |  |  |  |  |  | 0 | 0.999 | 0.998 | 0.999114 | 1 | chr8 | 146076248 | 146076248 | A | C | het | . | . | . | 68 | 1 | 1.45% | A | 31 | 5 | 13.89% | M | Somatic | 0.0173737 |
|  | exonic | MAST4 | synonymous SNV | NM_015183:c.C6285A:p.A2095A |  |  |  |  |  |  |  |  |  |  |  | chr5 | 66461859 | 66461859 | C | A | het | . | . | . | 35 | 0 | 0% | C | 25 | 4 | 13.79% | M | Somatic | 0.037381 |
|  | intronic | GRM4 |  |  |  |  |  |  |  |  |  |  |  |  |  | chr6 | 34024491 | 34024491 | A | C | het | . | . | . | 34 | 0 | 0% | A | 25 | 4 | 13.79% | M | Somatic | 0.0398731 |
|  | exonic | IMP5 | nonsynonymous SNV | NM_175882:c.A394C:p.T132P |  |  |  |  |  |  | 0.22 | 0.216 | 0.781239 | 0.204377 | 0.506393 | chr17 | 43922666 | 43922666 | A | C | het | . | . | . | 37 | 0 | 0% | A | 25 | 4 | 13.79% | M | Somatic | 0.0329545 |
|  | UTR3 | ECH1 |  |  |  |  |  |  |  |  |  |  |  |  |  | chr19 | 39306226 | 39306226 | T | C | het | . | . | . | 44 | 0 | 0% | T | 25 | 4 | 13.79% | Y | Somatic | 0.0218213 |
|  | intronic | CHCHD10 |  |  |  |  |  |  |  |  |  |  |  |  |  | chr22 | 24108242 | 24108242 | T | G | het | . | . | . | 36 | 0 | 0% | T | 25 | 4 | 13.79% | K | Somatic | 0.0350806 |
|  | exonic | RFX2 | synonymous SNV | NM_134433:c.T561G:p.G187G | 464;Name=lod=103 |  |  |  |  |  |  |  |  |  |  | chr19 | 6016244 | 6016244 | A | C | het | lung | carcinoma | adenocarcinoma | 58 | 1 | 1.69% | A | 69 | 11 | 13.75% | M | Somatic | 0.0101815 |
|  | intronic | SIPA1L2 |  |  |  |  |  |  |  |  |  |  |  |  |  | chr1 | 232551229 | 232551229 | T | G | het | . | . | . | 70 | 1 | 1.41% | T | 113 | 18 | 13.74% | K | Somatic | 0.0020995 |
|  | exonic | NTHL1 | nonsynonymous SNV | NM_002528:c.T482G:p.V161G |  |  |  |  |  |  | 0.01 | 0.963 | 0.978915 | 0.998932 | 0.999962 | chr16 | 2094698 | 2094698 | A | C | het | . | . | . | 46 | 0 | 0% | A | 19 | 3 | 13.64% | M | Somatic | 0.0307287 |
|  | intronic | EPN3 |  |  |  |  |  |  |  |  |  |  |  |  |  | chr17 | 48615387 | 48615387 | T | G | het | . | . | . | 51 | 1 | 1.92% | T | 38 | 6 | 13.64% | K | Somatic | 0.0340116 |
|  | exonic | PIAS4 | nonsynonymous SNV | NM_015897:c.T701G:p.V234G | 454;Name=lod=93 |  |  |  |  |  | 0 | 0.069 | 0.995236 | 0.999926 | 1 | chr19 | 4028746 | 4028746 | T | G | het | . | . | . | 74 | 1 | 1.33% | T | 19 | 3 | 13.64% | K | Somatic | 0.0354461 |
|  | intronic | C3 |  |  |  |  |  |  |  |  |  |  |  |  |  | chr19 | 6678071 | 6678071 | T | G | het | . | . | . | 108 | 2 | 1.82% | T | 57 | 9 | 13.64% | K | Somatic | 0.0026932 |
|  | exonic | LRFN1 | nonsynonymous SNV | NM_020862:c.G1213C:p.E405Q | 493;Name=lod=135 |  |  |  |  |  | 0.23 |  |  |  |  | chr19 | 39804764 | 39804764 | C | G | het | . | . | . | 48 | 0 | 0% | C | 19 | 3 | 13.64% | S | Somatic | 0.028133 |
|  | exonic | SYNGAP1 | synonymous SNV | NM_006772:c.A2388C:p.P796P | 289;Name=lod=20 |  |  |  |  | rs145844559 |  |  |  |  |  | chr6 | 33410717 | 33410717 | A | C | het | . | . | . | 95 | 1 | 1.04% | A | 70 | 11 | 13.58% | M | Somatic | 9.16E-04 |
|  | exonic | C1orf210 | nonsynonymous SNV | NM_001164829:c.A164C:p.H55P | 478;Name=lod=117 |  |  |  |  |  |  | 0.992 | 0.998222 | 0.775204 | 0.995306 | chr1 | 43748634 | 43748634 | T | G | het | . | . | . | 67 | 1 | 1.47% | T | 51 | 8 | 13.56% | K | Somatic | 0.0092174 |
|  | intronic | AGRN |  |  |  |  |  |  |  |  |  |  |  |  |  | chr1 | 982643 | 982643 | G | T | het | . | . | . | 55 | 0 | 0% | G | 13 | 2 | 13.33% | K | Somatic | 0.0434783 |
|  | UTR5 | CLDND1 |  |  | 560;Name=lod=252 |  |  |  |  |  |  |  |  |  |  | chr3 | 98241687 | 98241687 | A | C | het | . | . | . | 59 | 0 | 0% | A | 26 | 4 | 13.33% | M | Somatic | 0.0112241 |
|  | exonic | SNAPC4 | nonsynonymous SNV | NM_003086:c.G1877A:p.S626N |  |  |  |  |  |  | 0.79 | 0.042 | 0.847605 | 0.00195 | 0 | chr9 | 139277744 | 139277744 | C | T | het | . | . | . | 53 | 0 | 0% | C | 13 | 2 | 13.33% | Y | Somatic | 0.0460931 |
|  | exonic | PFKFB3 | nonsynonymous SNV | NM_001145443:c.A1265C:p.H422P | 490;Name=lod=131 |  |  |  |  |  | 0 | 0.995 | 0.997634 | 0.999994 | 1 | chr10 | 6266160 | 6266160 | A | C | het | . | . | . | 51 | 1 | 1.92% | A | 52 | 8 | 13.33% | M | Somatic | 0.026905 |
|  | exonic | EPS8L2 | nonsynonymous SNV | NM_022772:c.A1015T:p.S339C | 522;Name=lod=177 |  |  |  |  |  | 0 | 0.963 | 0.99119 | 0.684237 | 0.88057 | chr11 | 722121 | 722121 | A | T | het | . | . | . | 76 | 0 | 0% | A | 13 | 2 | 13.33% | W | Somatic | 0.025641 |
|  | intronic | TSPAN4 |  |  |  |  |  |  |  |  |  |  |  |  |  | chr11 | 865505 | 865505 | A | C | het | . | . | . | 51 | 0 | 0% | A | 13 | 2 | 13.33% | M | Somatic | 0.048951 |
|  | exonic | CDAN1 | nonsynonymous SNV | NM_138477:c.T2069G:p.V690G | 410;Name=lod=62 |  |  |  |  |  | 0 | 0.953 | 0.998916 | 0.943843 | 1 | chr15 | 43022901 | 43022901 | A | C | het | . | . | . | 60 | 0 | 0% | A | 26 | 4 | 13.33% | M | Somatic | 0.0107252 |
|  | intronic | EXOC3L1 |  |  |  |  |  |  |  |  |  |  |  |  |  | chr16 | 67218549 | 67218549 | G | A | het | . | . | . | 52 | 0 | 0% | G | 13 | 2 | 13.33% | R | Somatic | 0.0474898 |
|  | intronic | PLXNB2 |  |  |  |  |  |  |  |  |  |  |  |  |  | chr22 | 50722669 | 50722669 | A | C | het | . | . | . | 97 | 1 | 1.02% | A | 26 | 4 | 13.33% | M | Somatic | 0.0106899 |
|  | intronic | NACA |  |  |  |  |  |  |  |  |  |  |  |  |  | chr12 | 57112284 | 57112284 | T | G | het | . | . | . | 44 | 0 | 0% | T | 33 | 5 | 13.16% | K | Somatic | 0.018396 |
|  | exonic | KIAA0930 | nonsynonymous SNV | NM_001009880:c.A386G:p.D129G | 535;Name=lod=200 |  |  |  |  |  | 0.01 | 0.952 | 0.996019 | 0.999902 | 0.999955 | chr22 | 45601539 | 45601539 | T | C | het | . | . | . | 50 | 1 | 1.96% | T | 33 | 5 | 13.16% | Y | Somatic | 0.0488029 |
|  | intergenic | KGFLP2(dist=247809),ANKRD20A2(dist=100910) |  |  |  | 1 |  |  |  | rs75656085 |  |  |  |  |  | chr9 | 42267393 | 42267393 | C | G | het | . | . | . | 53 | 1 | 1.85% | C | 53 | 8 | 13.11% | S | Somatic | 0.0250796 |
|  | intronic | CYP11B2 |  |  |  | 0.93 |  |  |  |  |  |  |  |  |  | chr8 | 143995658 | 143995658 | T | G | het | . | . | . | 67 | 1 | 1.47% | T | 40 | 6 | 13.04% | K | Somatic | 0.0167806 |
|  | intronic | VAV2 |  |  |  |  |  |  |  |  |  |  |  |  |  | chr9 | 136652361 | 136652361 | T | G | het | . | . | . | 42 | 0 | 0% | T | 20 | 3 | 13.04% | K | Somatic | 0.0405449 |
|  | exonic | ANKRD11 | nonsynonymous SNV | NM_013275:c.T7229G:p.V2410G | 475;Name=lod=114 |  |  |  |  |  |  | 0.551 | 0.997471 | 0.106008 | 0.993844 | chr16 | 89345721 | 89345721 | A | C | het | . | . | . | 72 | 1 | 1.37% | A | 20 | 3 | 13.04% | M | Somatic | 0.0415833 |
|  | exonic | MYH3 | nonsynonymous SNV | NM_002470:c.T4009C:p.S1337P | 531;Name=lod=192 |  |  |  |  |  |  | 0.157 | 0.923269 | 0.089836 | 0.839345 | chr17 | 10538847 | 10538847 | A | G | het | . | . | . | 89 | 1 | 1.11% | A | 107 | 16 | 13.01% | R | Somatic | 8.17E-04 |
|  | exonic | SLC22A23 | nonsynonymous SNV | NM_015482:c.A1687C:p.T563P | 440;Name=lod=82 |  |  |  |  |  | 0.04 | 0.995 | 0.996845 | 0.894459 | 0.999999 | chr6 | 3284102 | 3284102 | T | G | het | . | . | . | 28 | 0 | 0% | T | 47 | 7 | 12.96% | K | Somatic | 0.0465839 |
|  | exonic | GP5 | nonsynonymous SNV | NM_004488:c.A898C:p.T300P |  |  |  |  |  |  | 0.02 | 0.998 | 0.99448 | 0.840589 | 0.999199 | chr3 | 194118114 | 194118114 | T | G | het | . | . | . | 33 | 0 | 0% | T | 27 | 4 | 12.90% | K | Somatic | 0.0495219 |
|  | exonic | FAM22D | nonsynonymous SNV | NM_001009610:c.A241C:p.T81P |  | 0.99 |  |  |  |  | 0.2 | 0.001 | 0.107887 | 8.20E-05 | 0.596033 | chr10 | 89120312 | 89120312 | A | C | het | . | . | . | 62 | 0 | 0% | A | 27 | 4 | 12.90% | M | Somatic | 0.0107767 |
|  | exonic | WDR74 | nonsynonymous SNV | NM_018093:c.T38G:p.V13G | 500;Name=lod=144 |  |  |  |  |  | 0 |  |  |  |  | chr11 | 62607005 | 62607005 | A | C | het | . | . | . | 55 | 1 | 1.79% | A | 34 | 5 | 12.82% | M | Somatic | 0.0408523 |
|  | exonic | KCTD10 | nonsynonymous SNV | NM_031954:c.C841G:p.R281G | 460;Name=lod=99 |  |  |  |  |  | 0.29 | 0.003 | 0.99967 | 0.999847 | 1 | chr12 | 109889501 | 109889501 | G | C | het | . | . | . | 63 | 1 | 1.56% | G | 34 | 5 | 12.82% | S | Somatic | 0.0280528 |
|  | upstream | PTX4 |  |  |  |  |  |  |  |  |  |  |  |  |  | chr16 | 1538495 | 1538495 | A | C | het | . | . | . | 51 | 0 | 0% | A | 34 | 5 | 12.82% | M | Somatic | 0.0131005 |
|  | exonic | TRIP10 | nonsynonymous SNV | NM_004240:c.A904C:p.T302P | 475;Name=lod=114 |  |  |  |  |  | 0.01 | 0.011 | 0.97394 | 0.991647 | 0.846484 | chr19 | 6744925 | 6744925 | A | C | het | . | . | . | 125 | 1 | 0.79% | A | 68 | 10 | 12.82% | M | Somatic | 3.43E-04 |
|  | exonic | GTPBP3 | nonsynonymous SNV | NM_001128855:c.A488G:p.D163G | 449;Name=lod=89 |  |  |  |  |  | 0 | 0.999 | 0.998081 | 0.999814 | 1 | chr19 | 17449447 | 17449447 | A | G | het | . | . | . | 125 | 1 | 0.79% | A | 34 | 5 | 12.82% | R | Somatic | 0.0029653 |
|  | intronic | LRRK1 |  |  | 437;Name=lod=80 |  |  |  |  |  |  |  |  |  |  | chr15 | 101567592 | 101567592 | T | G | het | . | . | . | 56 | 1 | 1.75% | T | 41 | 6 | 12.77% | K | Somatic | 0.0317715 |
|  | exonic | NUP62 | nonsynonymous SNV | NM_001193357:c.G1387C:p.A463P | 309;Name=lod=24 |  |  |  |  |  | 0.01 | 0.017 | 0.942194 | 6.61E-04 | 0.999971 | chr19 | 50411678 | 50411678 | C | G | het | . | . | . | 135 | 2 | 1.46% | C | 82 | 12 | 12.77% | S | Somatic | 5.16E-04 |
|  | exonic | RPS6KA4 | synonymous SNV | NM_001006944:c.A2157C:p.A719A | 571;Name=lod=279 |  |  |  |  |  |  |  |  |  |  | chr11 | 64138808 | 64138808 | A | C | het | . | . | . | 125 | 1 | 0.79% | A | 48 | 7 | 12.73% | M | Somatic | 0.0010968 |
|  | intronic | PNPLA6 |  |  |  |  |  |  |  |  |  |  |  |  |  | chr19 | 7621485 | 7621485 | A | C | het | . | . | . | 89 | 1 | 1.11% | A | 48 | 7 | 12.73% | M | Somatic | 0.0048926 |
|  | intronic | KIAA1274 |  |  |  |  |  |  |  |  |  |  |  |  |  | chr10 | 72285908 | 72285908 | A | G | het | . | . | . | 38 | 0 | 0% | A | 55 | 8 | 12.70% | R | Somatic | 0.0191637 |
|  | exonic | GJB3 | nonsynonymous SNV | NM_001005752:c.T694C:p.S232P |  |  |  |  |  |  | 0.14 | 0.565 | 0.99759 | 0.298565 | 0.001195 | chr1 | 35251057 | 35251057 | T | C | het | . | . | . | 52 | 0 | 0% | T | 21 | 3 | 12.50% | Y | Somatic | 0.0287909 |
|  | exonic | IGFN1 | synonymous SNV | NM_001164586:c.A9375C:p.P3125P |  |  |  |  |  |  |  |  |  |  |  | chr1 | 201185661 | 201185661 | A | C | het | . | . | . | 54 | 0 | 0% | A | 21 | 3 | 12.50% | M | Somatic | 0.026605 |
|  | exonic | STARD7 | nonsynonymous SNV | NM_020151:c.A1048C:p.T350P |  |  |  |  |  |  | 0.23 |  |  |  |  | chr2 | 96852533 | 96852533 | T | G | het | . | . | . | 70 | 1 | 1.41% | T | 91 | 13 | 12.50% | K | Somatic | 0.0055794 |
|  | exonic | SYNPO | synonymous SNV | NM_001109974:c.A1374C:p.S458S |  |  |  |  |  |  |  |  |  |  |  | chr5 | 150029211 | 150029211 | A | C | het | . | . | . | 74 | 0 | 0% | A | 28 | 4 | 12.50% | M | Somatic | 0.0072388 |
|  | exonic | MYOZ3 | nonsynonymous SNV | NM_001122853:c.T511G:p.F171V | 387;Name=lod=50 |  |  |  |  |  | 0.4 | 0.983 | 0.879505 | 0.279519 | 0.990814 | chr5 | 150051988 | 150051988 | T | G | het | . | . | . | 46 | 0 | 0% | T | 21 | 3 | 12.50% | K | Somatic | 0.0369748 |
|  | exonic | ZMIZ2 | nonsynonymous SNV | NM_174929:c.A1435C:p.T479P | 643;Name=lod=548 |  |  |  |  |  | 0 | 0.995 | 0.997667 | 0.999977 | 0.999996 | chr7 | 44801421 | 44801421 | A | C | het | . | . | . | 74 | 1 | 1.33% | A | 21 | 3 | 12.50% | M | Somatic | 0.0431482 |
|  | exonic | KIAA1161 | nonsynonymous SNV | NM_020702:c.C787T:p.H263Y | 371;Name=lod=43 |  |  |  |  |  | 0.49 |  |  |  |  | chr9 | 34372155 | 34372155 | G | A | het | . | . | . | 62 | 0 | 0% | G | 14 | 2 | 12.50% | R | Somatic | 0.03996 |
|  | intronic | CA9 |  |  |  |  |  |  |  |  |  |  |  |  |  | chr9 | 35675743 | 35675743 | A | C | het | . | . | . | 59 | 1 | 1.67% | A | 28 | 4 | 12.50% | M | Somatic | 0.047969 |
|  | exonic | ANKRD20A1,ANKRD20A3 | nonsynonymous SNV | NM_001012419:c.G1447C:p.V483L |  | 0.99 |  |  |  | rs2982946 | 1 | 0.036 | 0.210848 | 3.91E-04 | 0.654467 | chr9 | 67966723 | 67966723 | G | C | het | . | . | . | 40 | 0 | 0% | G | 21 | 3 | 12.50% | S | Somatic | 0.0485791 |
|  | exonic;splicing | NOTCH1;NOTCH1 | synonymous SNV | NM_017617:c.G2589A:p.G863G |  |  |  |  |  |  |  |  |  |  |  | chr9 | 139405256 | 139405256 | C | T | het | . | . | . | 72 | 0 | 0% | C | 14 | 2 | 12.50% | Y | Somatic | 0.031348 |
|  | exonic | IRF7 | nonsynonymous SNV | NM_004031:c.T1171G:p.W391G | 468;Name=lod=106 |  |  |  |  |  | 0 | 1 | 0.997867 | 0.856884 | 0.998909 | chr11 | 613311 | 613311 | A | C | het | . | . | . | 66 | 0 | 0% | A | 21 | 3 | 12.50% | M | Somatic | 0.0172285 |
|  | UTR3 | SYT8 |  |  |  |  |  |  |  |  |  |  |  |  |  | chr11 | 1858688 | 1858688 | C | A | het | . | . | . | 54 | 0 | 0% | C | 14 | 2 | 12.50% | M | Somatic | 0.0496894 |
|  | exonic | SKOR1 | nonsynonymous SNV | NM_001031807:c.T998G:p.V333G | 572;Name=lod=281 |  |  |  |  |  | 0 | 0.986 | 0.992094 | 0.005307 | 0.961922 | chr15 | 68119296 | 68119296 | T | G | het | . | . | . | 68 | 0 | 0% | T | 14 | 2 | 12.50% | K | Somatic | 0.0344234 |
|  | intronic | ISL2 |  |  |  |  |  |  |  |  |  |  |  |  |  | chr15 | 76633968 | 76633968 | A | C | het | . | . | . | 50 | 0 | 0% | A | 21 | 3 | 12.50% | M | Somatic | 0.031223 |
|  | exonic | BAIAP3 | nonsynonymous SNV | NM_001199096:c.A2414T:p.Q805L | 478;Name=lod=117 |  |  |  |  |  | 1 | 0.995 | 0.966889 | 0.970715 | 1 | chr16 | 1396471 | 1396471 | A | T | het | . | . | . | 61 | 0 | 0% | A | 14 | 2 | 12.50% | W | Somatic | 0.0410116 |
|  | exonic | NTHL1 | synonymous SNV | NM_002528:c.C474G:p.G158G | 399;Name=lod=56 |  |  |  |  |  |  |  |  |  |  | chr16 | 2094706 | 2094706 | G | C | het | . | . | . | 43 | 0 | 0% | G | 21 | 3 | 12.50% | S | Somatic | 0.0422503 |
|  | exonic | ANKRD11 | synonymous SNV | NM_013275:c.T6918A:p.P2306P |  |  |  |  |  |  |  |  |  |  |  | chr16 | 89346032 | 89346032 | A | T | het | . | . | . | 67 | 0 | 0% | A | 14 | 2 | 12.50% | W | Somatic | 0.035263 |
|  | exonic | GAA | nonsynonymous SNV | NM_000152:c.A158C:p.H53P |  |  |  |  |  |  | 0.11 | 0.947 | 0.018534 | 5.25E-04 | 0.002649 | chr17 | 78078543 | 78078543 | A | C | het | . | . | . | 43 | 0 | 0% | A | 21 | 3 | 12.50% | M | Somatic | 0.0422503 |
|  | exonic | ZNF516 | nonsynonymous SNV | NM_014643:c.T487G:p.S163A |  |  |  |  |  |  | 0.07 |  |  |  |  | chr18 | 74154524 | 74154524 | A | C | het | . | . | . | 53 | 0 | 0% | A | 21 | 3 | 12.50% | M | Somatic | 0.0276692 |
|  | exonic | GATAD2A | nonsynonymous SNV | NM_017660:c.A1006C:p.T336P |  |  |  |  |  |  | 0.02 | 0.024 | 0.051401 | 0.001611 | 0.97832 | chr19 | 19609333 | 19609333 | A | C | het | . | . | . | 79 | 1 | 1.25% | A | 42 | 6 | 12.50% | M | Somatic | 0.0111647 |
|  | intergenic | MIR4436A(dist=409253),NONE(dist=NONE) |  |  | 346;Name=lod=34 | 0.99 |  |  |  |  |  |  |  |  |  | chr2 | 89521221 | 89521221 | C | T | het | . | . | . | 207 | 1 | 0.48% | C | 380 | 54 | 12.44% | Y | Somatic | 4.19E-09 |
|  | intronic | SHANK1 |  |  |  |  |  |  |  |  |  |  |  |  |  | chr19 | 51217040 | 51217040 | T | G | het | . | . | . | 114 | 2 | 1.72% | T | 64 | 9 | 12.33% | K | Somatic | 0.0035327 |
|  | UTR3 | PTPRF |  |  | 622;Name=lod=450 |  |  |  |  |  |  |  |  |  |  | chr1 | 44087682 | 44087682 | T | C | het | . | . | . | 91 | 1 | 1.09% | T | 57 | 8 | 12.31% | Y | Somatic | 0.0039252 |
|  | exonic | PCOLCE | nonsynonymous SNV | NM_002593:c.A248C:p.D83A | 550;Name=lod=229 |  |  |  |  |  | 0 | 0.997 | 0.997568 | 0.999914 | 1 | chr7 | 100201625 | 100201625 | A | C | het | . | . | . | 98 | 2 | 2% | A | 50 | 7 | 12.28% | M | Somatic | 0.011728 |
|  | intronic | MUC20 |  |  |  | 0.98 |  |  |  |  | 0 |  |  |  |  | chr3 | 195452361 | 195452361 | G | C | het | . | . | . | 88 | 1 | 1.12% | G | 43 | 6 | 12.24% | S | Somatic | 0.0082116 |
|  | intronic | CHD5 |  |  |  |  |  |  |  |  |  |  |  |  |  | chr1 | 6190241 | 6190241 | T | G | het | . | . | . | 66 | 1 | 1.49% | T | 79 | 11 | 12.22% | K | Somatic | 0.0100615 |
|  | intronic | NBPF15,NBPF16 |  |  |  | 1 |  |  |  |  |  |  |  |  |  | chr1 | 148593567 | 148593567 | G | C | het | . | . | . | 75 | 1 | 1.32% | G | 36 | 5 | 12.20% | S | Somatic | 0.0196479 |
|  | exonic | GJB1 | nonsynonymous SNV | NM_000166:c.A512C:p.Y171S | 561;Name=lod=253 |  |  |  |  |  | 0.45 | 0.966 | 0.968013 | 0.999539 | 0.999999 | chrX | 70444069 | 70444069 | A | C | het | . | . | . | 35 | 0 | 0% | A | 36 | 5 | 12.20% | M | Somatic | 0.0405632 |
|  | exonic | MYSM1 | nonsynonymous SNV | NM_001085487:c.T44G:p.V15G |  |  |  |  |  |  |  | 0.283326 | 0.918159 | 0.074193 | 0.067391 | chr1 | 59165681 | 59165681 | A | C | het | . | . | . | 56 | 0 | 0% | A | 29 | 4 | 12.12% | M | Somatic | 0.0167593 |
|  | exonic | ARHGEF26 | nonsynonymous SNV | NM_015595:c.T20G:p.V7G | 363;Name=lod=40 |  |  |  |  |  | 0 |  |  |  |  | chr3 | 153839801 | 153839801 | T | G | het | . | . | . | 60 | 1 | 1.64% | T | 29 | 4 | 12.12% | K | Somatic | 0.0497979 |
|  | exonic | TUBB8 | synonymous SNV | NM_177987:c.C751A:p.R251R | 849;Name=lod=3750 | 0.97 |  |  |  |  |  |  |  |  |  | chr10 | 93581 | 93581 | G | T | het | . | . | . | 60 | 1 | 1.64% | G | 58 | 8 | 12.12% | K | Somatic | 0.0218578 |
|  | exonic | DUX2,DUX4,DUX4L3,DUX4L5,DUX4L6,DUX4L7 | synonymous SNV | NM_001127387:c.T753G:p.L251L |  | 1 |  |  |  |  |  |  |  |  |  | chr10 | 135481224 | 135481224 | T | G | het | . | . | . | 81 | 0 | 0% | T | 29 | 4 | 12.12% | K | Somatic | 0.0061323 |
|  | exonic | TAF1C | synonymous SNV | NM_001243158:c.A1560C:p.T520T |  |  |  |  |  |  |  |  |  |  |  | chr16 | 84212601 | 84212601 | T | G | het | . | . | . | 63 | 1 | 1.56% | T | 29 | 4 | 12.12% | K | Somatic | 0.0443195 |
|  | exonic | TP53INP2 | synonymous SNV | NM_021202:c.T225C:p.P75P | 631;Name=lod=487 |  |  |  |  |  |  |  |  |  |  | chr20 | 33297140 | 33297140 | T | C | het | . | . | . | 69 | 1 | 1.43% | T | 29 | 4 | 12.12% | Y | Somatic | 0.0354317 |
|  | exonic | MAST2 | nonsynonymous SNV | NM_015112:c.A4438C:p.T1480P | 472;Name=lod=110 |  |  |  |  |  | 0.01 | 0.194 | 0.911495 | 0.002586 | 0.997782 | chr1 | 46500779 | 46500779 | A | C | het | . | . | . | 75 | 0 | 0% | A | 51 | 7 | 12.07% | M | Somatic | 0.002417 |
|  | ncRNA_intronic | GOLGA6L5 |  |  |  | 0.98 |  |  |  |  |  |  |  |  |  | chr15 | 85057542 | 85057542 | C | T | het | . | . | . | 38 | 0 | 0% | C | 51 | 7 | 12.07% | Y | Somatic | 0.025226 |
|  | UTR3 | CCDC97 |  |  |  |  |  |  |  |  |  |  |  |  |  | chr19 | 41828629 | 41828629 | A | C | het | . | . | . | 35 | 0 | 0% | A | 51 | 7 | 12.07% | M | Somatic | 0.031738 |
|  | intronic | CAMK2A |  |  | 570;Name=lod=276 |  |  |  |  |  |  |  |  |  |  | chr5 | 149630419 | 149630419 | A | C | het | . | . | . | 35 | 0 | 0% | A | 44 | 6 | 12% | M | Somatic | 0.0363338 |
|  | splicing | AARS2(NM_020745:exon2:c.243+2T>G) |  |  | 493;Name=lod=134 |  |  |  |  |  |  |  |  |  |  | chr6 | 44280816 | 44280816 | A | C | het | . | . | . | 45 | 0 | 0% | A | 22 | 3 | 12% | M | Somatic | 0.0420168 |
|  | exonic | LIMK1 | nonsynonymous SNV | NM_001204426:c.T1718C:p.L573P | 488;Name=lod=128 |  |  |  |  |  | 0 | 0.992 | 0.994112 | 0.999883 | 1 | chr7 | 73535507 | 73535507 | T | C | het | . | . | . | 82 | 0 | 0% | T | 22 | 3 | 12% | Y | Somatic | 0.0115878 |
|  | exonic | TRIP6 | nonsynonymous SNV | NM_003302:c.T823G:p.Y275D | 482;Name=lod=121 |  |  |  |  |  | 0.01 | 0.986 | 0.998134 | 0.961222 | 0.999986 | chr7 | 100468079 | 100468079 | T | G | het | . | . | . | 43 | 0 | 0% | T | 44 | 6 | 12% | K | Somatic | 0.0208472 |
|  | exonic | TOP1MT | nonsynonymous SNV | NM_052963:c.T1616G:p.V539G |  |  |  |  |  |  | 0 | 0.634 | 0.821364 | 0.764442 | 0.23227 | chr8 | 144392325 | 144392325 | A | C | het | . | . | . | 48 | 0 | 0% | A | 22 | 3 | 12% | M | Somatic | 0.0369799 |
|  | exonic | TLX1 | nonsynonymous SNV | NM_001195517:c.A487C:p.M163L | 652;Name=lod=597 |  |  |  |  |  | 0.81 | 0.192 | 0.969099 | 0.079096 | 0.997755 | chr10 | 102891785 | 102891785 | A | C | het | . | . | . | 51 | 0 | 0% | A | 22 | 3 | 12% | M | Somatic | 0.0327169 |
|  | intronic | PTS |  |  | 597;Name=lod=355 |  |  |  |  | rs61900919 |  |  |  |  |  | chr11 | 112099312 | 112099312 | G | T | het | . | . | . | 45 | 0 | 0% | G | 22 | 3 | 12% | K | Somatic | 0.0420168 |
|  | exonic | ACACB | nonsynonymous SNV | NM_001093:c.A7240C:p.T2414P | 545;Name=lod=218 |  |  |  |  |  | 0.09 | 0.003 | 0.827428 | 0.027544 | 0.931736 | chr12 | 109703320 | 109703320 | A | C | het | . | . | . | 47 | 0 | 0% | A | 22 | 3 | 12% | M | Somatic | 0.0385647 |
|  | splicing | PCYT2(NM_002861:exon13:c.1058+2T>G,NM_001184917:exon14:c.1112+2T>G) |  |  | 472;Name=lod=110 |  |  |  |  |  |  |  |  |  |  | chr17 | 79862940 | 79862940 | A | C | het | . | . | . | 54 | 0 | 0% | A | 22 | 3 | 12% | M | Somatic | 0.0290848 |
|  | intronic | PNPLA6 |  |  |  |  |  |  |  |  |  |  |  |  |  | chr19 | 7604970 | 7604970 | T | G | het | . | . | . | 48 | 0 | 0% | T | 22 | 3 | 12% | K | Somatic | 0.0369799 |
|  | splicing | ZNF543(NM_213598:exon1:c.18+2T>G) |  |  | 272;Name=lod=17 |  |  |  |  |  |  |  |  |  |  | chr19 | 57832241 | 57832241 | T | G | het | . | . | . | 45 | 0 | 0% | T | 22 | 3 | 12% | K | Somatic | 0.0420168 |
|  | intronic | SYNGR1 |  |  |  |  |  |  |  |  |  |  |  |  |  | chr22 | 39777663 | 39777663 | T | C | het | . | . | . | 54 | 0 | 0% | T | 22 | 3 | 12% | Y | Somatic | 0.0290848 |
|  | exonic | PIF1 | synonymous SNV | NM_025049:c.C888G:p.G296G | 534;Name=lod=198 |  |  |  |  |  |  |  |  |  |  | chr15 | 65113649 | 65113649 | G | C | het | . | . | . | 59 | 1 | 1.67% | G | 81 | 11 | 11.96% | S | Somatic | 0.0176433 |
|  | exonic | HSPG2 | nonsynonymous SNV | NM_005529:c.A10924C:p.T3642P | 428;Name=lod=73 |  |  |  |  | rs75765186 | 0.03 | 0.967 | 0.995147 | 0.997003 | 0.999346 | chr1 | 22160014 | 22160014 | T | G | het | . | . | . | 36 | 0 | 0% | T | 37 | 5 | 11.90% | K | Somatic | 0.0402948 |
|  | exonic | POT1 | nonsynonymous SNV | NM_001042594:c.T194G:p.V65G | 409;Name=lod=61 |  |  |  |  |  | 0.05 | 0.048 | 0.999002 | 0.968493 | 0.999999 | chr7 | 124499126 | 124499126 | A | C | het | . | . | . | 130 | 2 | 1.52% | A | 74 | 10 | 11.90% | M | Somatic | 0.0016758 |
|  | exonic | ABCC2 | synonymous SNV | NM_000392:c.C1992G:p.G664G | 336;Name=lod=31 |  |  |  |  |  |  |  |  |  |  | chr10 | 101572799 | 101572799 | C | G | het | . | . | . | 95 | 1 | 1.04% | C | 157 | 21 | 11.80% | S | Somatic | 6.71E-04 |
|  | intronic | CC2D1B |  |  |  |  |  |  |  |  |  |  |  |  |  | chr1 | 52826009 | 52826009 | T | G | het | . | . | . | 53 | 0 | 0% | T | 30 | 4 | 11.76% | K | Somatic | 0.0208348 |
|  | exonic | STX1A | synonymous SNV | NM_001165903:c.G411A:p.T137T | 510;Name=lod=158 |  |  |  |  |  |  |  |  |  |  | chr7 | 73118533 | 73118533 | C | T | het | . | . | . | 85 | 0 | 0% | C | 30 | 4 | 11.76% | Y | Somatic | 0.0058403 |
|  | exonic | FAM84B | nonsynonymous SNV | NM_174911:c.C880G:p.R294G |  |  |  |  |  |  | 0 | 0.448381 | 0.939887 | 0.107051 | 0.295303 | chr8 | 127568755 | 127568755 | G | C | het | . | . | . | 64 | 0 | 0% | G | 15 | 2 | 11.76% | S | Somatic | 0.0419753 |
|  | exonic | FAM83H | synonymous SNV | NM_198488:c.A3009G:p.S1003S |  |  |  |  |  |  |  |  |  |  |  | chr8 | 144808622 | 144808622 | T | C | het | . | . | . | 73 | 0 | 0% | T | 15 | 2 | 11.76% | Y | Somatic | 0.0339576 |
|  | exonic | RASSF10 | nonsynonymous SNV | NM_001080521:c.A1186C:p.T396P | 668;Name=lod=693 |  |  |  |  |  | 0.04 |  |  |  |  | chr11 | 13032309 | 13032309 | A | C | het | . | . | . | 63 | 0 | 0% | A | 15 | 2 | 11.76% | M | Somatic | 0.043038 |
|  | intronic | NOS1 |  |  |  |  |  |  |  |  |  |  |  |  |  | chr12 | 117696190 | 117696190 | T | G | het | . | . | . | 32 | 0 | 0% | T | 45 | 6 | 11.76% | K | Somatic | 0.0477139 |
|  | exonic | SLX4 | synonymous SNV | NM_032444:c.G3783C:p.P1261P |  |  |  |  |  |  |  |  |  |  |  | chr16 | 3639856 | 3639856 | C | G | het | . | . | . | 110 | 2 | 1.79% | C | 45 | 6 | 11.76% | S | Somatic | 0.0120952 |
|  | exonic | ZNF768 | nonsynonymous SNV | NM_024671:c.G1333C:p.A445P | 355;Name=lod=37 |  |  |  |  |  |  | 0.831 | 0.998151 | 0.552768 | 0.999696 | chr16 | 30536128 | 30536128 | C | G | het | . | . | . | 61 | 0 | 0% | C | 15 | 2 | 11.76% | S | Somatic | 0.045288 |
|  | exonic | PPP1R29 | nonsynonymous SNV | NM_052906:c.A575C:p.D192A | 667;Name=lod=687 |  |  |  |  |  | 0.03 | 0.063 | 0.996831 | 0.813809 | 0.999275 | chr22 | 37771000 | 37771000 | T | G | het | . | . | . | 109 | 2 | 1.80% | T | 30 | 4 | 11.76% | K | Somatic | 0.0271289 |
|  | exonic | DDX49 | nonsynonymous SNV | NM_019070:c.A973C:p.T325P | 524;Name=lod=179 |  |  |  |  |  | 0.02 | 0.001 | 0.997418 | 0.999896 | 1 | chr19 | 19035734 | 19035734 | A | C | het | . | . | . | 57 | 1 | 1.72% | A | 53 | 7 | 11.67% | M | Somatic | 0.0341188 |
|  | exonic | SYT15 | synonymous SNV | NM_031912:c.A132C:p.T44T |  |  |  |  |  |  |  |  |  |  |  | chr10 | 46969329 | 46969329 | T | G | het | . | . | . | 75 | 1 | 1.32% | T | 38 | 5 | 11.63% | K | Somatic | 0.022839 |
|  | exonic | NR1D1 | nonsynonymous SNV | NM_021724:c.A731C:p.H244P | 405;Name=lod=59 |  |  |  |  |  | 0.08 | 0.154 | 0.996764 | 0.787985 | 0.238517 | chr17 | 38252214 | 38252214 | T | G | het | . | . | . | 42 | 0 | 0% | T | 38 | 5 | 11.63% | K | Somatic | 0.0293461 |
|  | intergenic | GOLGA8G(dist=109372),HERC2P9(dist=12073) |  |  |  | 0.99 |  |  |  | rs443481 |  |  |  |  |  | chr15 | 28887515 | 28887515 | T | C | het | . | . | . | 45 | 0 | 0% | T | 61 | 8 | 11.59% | Y | Somatic | 0.0151933 |
|  | intronic | CCDC153 |  |  |  |  |  |  |  |  |  |  |  |  |  | chr11 | 119063848 | 119063848 | T | G | het | . | . | . | 39 | 0 | 0% | T | 84 | 11 | 11.58% | K | Somatic | 0.0189961 |
|  | UTR5 | HOXD9 |  |  | 762;Name=lod=1665 |  |  |  |  |  |  |  |  |  |  | chr2 | 176987489 | 176987489 | A | G | het | . | . | . | 48 | 0 | 0% | A | 23 | 3 | 11.54% | R | Somatic | 0.0401086 |
|  | exonic | LTF | nonsynonymous SNV | NM_001199149:c.T967G:p.C323G | 343;Name=lod=33 |  |  |  |  |  | 0 | 1 | 0.995312 | 1 | 1 | chr3 | 46490467 | 46490467 | A | C | het | . | . | . | 45 | 0 | 0% | A | 23 | 3 | 11.54% | M | Somatic | 0.0454903 |
|  | intronic | SEMA3B |  |  |  |  |  |  |  |  |  |  |  |  |  | chr3 | 50310895 | 50310895 | A | C | het | . | . | . | 76 | 1 | 1.30% | A | 23 | 3 | 11.54% | M | Somatic | 0.0486624 |
|  | exonic | ZNF784 | nonsynonymous SNV | NM_203374:c.T950G:p.V317G |  |  |  |  |  |  | 0 | 0.137 | 0.820539 | 0.030738 | 0.569584 | chr19 | 56133139 | 56133139 | A | C | het | . | . | . | 43 | 0 | 0% | A | 23 | 3 | 11.54% | M | Somatic | 0.049624 |
|  | exonic | FLNA | nonsynonymous SNV | NM_001110556:c.T4739C:p.L1580P | 468;Name=lod=106 |  |  |  |  |  | 0 | 0.989 | 0.998676 | 0.999415 | 0.999994 | chrX | 153586583 | 153586583 | A | G | het | . | . | . | 69 | 0 | 0% | A | 23 | 3 | 11.54% | R | Somatic | 0.0187841 |
|  | exonic | CDX1 | nonsynonymous SNV | NM_001804:c.A700C:p.T234P |  |  |  |  |  |  | 0.07 | 0.001 | 0.13273 | 0.050631 | 0.834061 | chr5 | 149563145 | 149563145 | A | C | het | . | . | . | 63 | 1 | 1.56% | A | 54 | 7 | 11.48% | M | Somatic | 0.0262428 |
|  | exonic | SMAP1 | synonymous SNV | NM_021940:c.A1131C:p.A377A | 475;Name=lod=114 |  |  |  |  |  |  |  |  |  |  | chr6 | 71567875 | 71567875 | A | C | het | . | . | . | 86 | 1 | 1.15% | A | 108 | 14 | 11.48% | M | Somatic | 0.0027785 |
|  | exonic | C4A,C4B,LOC100293534 | nonsynonymous SNV | NM_001002029:c.T2752G:p.S918A |  | 1 |  |  |  |  | 0.08 | 0.024 | 0.071116 | 0 | 0.003181 | chr6 | 31962434 | 31962434 | T | G | het | . | . | . | 62 | 0 | 0% | T | 31 | 4 | 11.43% | K | Somatic | 0.0151118 |
|  | intronic | ABCA2 |  |  |  |  |  |  |  |  |  |  |  |  |  | chr9 | 139905204 | 139905204 | T | G | het | . | . | . | 131 | 2 | 1.50% | T | 31 | 4 | 11.43% | K | Somatic | 0.0176796 |
|  | UTR3 | ZFYVE19 |  |  |  |  |  |  |  |  |  |  |  |  |  | chr15 | 41106429 | 41106429 | A | C | het | . | . | . | 41 | 0 | 0% | A | 62 | 8 | 11.43% | M | Somatic | 0.0213811 |
|  | exonic | SUPT5H | nonsynonymous SNV | NM_001130825:c.A2203C:p.T735P | 575;Name=lod=289 |  |  |  |  |  | 0.14 | 0 | 0.996643 | 0.999996 | 1 | chr19 | 39963711 | 39963711 | A | C | het | lung | carcinoma | adenocarcinoma | 87 | 1 | 1.14% | A | 31 | 4 | 11.43% | M | Somatic | 0.0228272 |
|  | intronic | BPIFB3 |  |  |  |  |  |  |  |  |  |  |  |  |  | chr20 | 31659988 | 31659988 | G | T | het | . | . | . | 55 | 1 | 1.79% | G | 62 | 8 | 11.43% | K | Somatic | 0.0360547 |
|  | intronic | PDE4DIP |  |  |  | 0.95 |  |  |  |  |  |  |  |  |  | chr1 | 144906010 | 144906010 | T | A | het | . | . | . | 46 | 0 | 0% | T | 39 | 5 | 11.36% | W | Somatic | 0.0247105 |
|  | exonic | SH3TC1 | synonymous SNV | NM_018986:c.G1026T:p.L342L | 366;Name=lod=41 |  |  |  |  |  |  |  |  |  |  | chr4 | 8221171 | 8221171 | G | T | het | . | . | . | 113 | 1 | 0.88% | G | 39 | 5 | 11.36% | K | Somatic | 0.006667 |
|  | exonic | ADAM12 | synonymous SNV | NM_003474:c.A2484C:p.A828A |  |  |  |  |  |  |  |  |  |  |  | chr10 | 127724769 | 127724769 | T | G | het | . | . | . | 68 | 1 | 1.45% | T | 78 | 10 | 11.36% | K | Somatic | 0.0137332 |
|  | exonic | RPL3L | nonsynonymous SNV | NM_005061:c.T614G:p.V205G | 493;Name=lod=135 |  |  |  |  |  | 0 | 0.063 | 0.998274 | 0.999647 | 0.999997 | chr16 | 1997269 | 1997269 | A | C | het | . | . | . | 104 | 2 | 1.89% | A | 39 | 5 | 11.36% | M | Somatic | 0.0232234 |
|  | exonic | COL18A1 | nonsynonymous SNV | NM_030582:c.T1087G:p.F363V |  |  |  |  |  |  | 0.4 | 0.274668 | 0.001242 | 0.29864 | 0.81339 | chr21 | 46888596 | 46888596 | T | G | het | . | . | . | 88 | 1 | 1.12% | T | 39 | 5 | 11.36% | K | Somatic | 0.0151262 |
|  | exonic | TFAP4 | nonsynonymous SNV | NM_003223:c.T704G:p.V235G | 536;Name=lod=202 |  |  |  |  |  | 0 | 0.626494 | 0.997832 | 0.285828 | 0.999997 | chr16 | 4310209 | 4310209 | A | C | het | . | . | . | 42 | 0 | 0% | A | 47 | 6 | 11.32% | M | Somatic | 0.026415 |
|  | exonic | FLCN | nonsynonymous SNV | NM_144606:c.T884G:p.V295G |  |  |  |  |  |  | 0.27 | 0.321093 | 0.852779 | 1.10E-05 | 0.819012 | chr17 | 17124838 | 17124838 | A | C | het | . | . | . | 69 | 1 | 1.43% | A | 55 | 7 | 11.29% | M | Somatic | 0.0205314 |
|  | intronic | MAST2 |  |  |  |  |  |  |  |  |  |  |  |  |  | chr1 | 46474075 | 46474075 | A | C | het | . | . | . | 60 | 1 | 1.64% | A | 63 | 8 | 11.27% | M | Somatic | 0.0285157 |
|  | intronic | RNF10 |  |  |  |  |  |  |  |  |  |  |  |  |  | chr12 | 121001560 | 121001560 | A | G | het | . | . | . | 52 | 1 | 1.89% | A | 63 | 8 | 11.27% | R | Somatic | 0.0449877 |
|  | intergenic | LINC00221(dist=218542),NONE(dist=NONE) |  |  |  |  |  |  |  |  | 0.09 |  |  |  |  | chr14 | 107170071 | 107170071 | T | G | het | . | . | . | 452 | 0 | 0% | T | 497 | 63 | 11.25% | K | Somatic | 1.23E-17 |
|  | exonic | ZBTB4 | synonymous SNV | NM_001128833:c.T1311C:p.A437A | 295;Name=lod=21 |  |  |  |  |  |  |  |  |  |  | chr17 | 7366990 | 7366990 | A | G | het | . | . | . | 117 | 2 | 1.68% | A | 71 | 9 | 11.25% | R | Somatic | 0.0050062 |
|  | intronic | CDK11B,SLC35E2B |  |  |  | 0.98 |  |  |  |  |  |  |  |  |  | chr1 | 1606896 | 1606896 | C | G | het | . | . | . | 56 | 0 | 0% | C | 24 | 3 | 11.11% | S | Somatic | 0.0318347 |
|  | UTR3 | ESPN |  |  |  |  |  |  |  |  |  |  |  |  |  | chr1 | 6520214 | 6520214 | A | T | het | . | . | . | 70 | 0 | 0% | A | 16 | 2 | 11.11% | W | Somatic | 0.0399687 |
|  | intronic | CLSTN1 |  |  |  |  |  |  |  |  |  |  |  |  |  | chr1 | 9791775 | 9791775 | A | G | het | . | . | . | 57 | 0 | 0% | A | 24 | 3 | 11.11% | R | Somatic | 0.0306977 |
|  | upstream | USP34 |  |  | 634;Name=lod=503 |  |  |  |  |  |  |  |  |  |  | chr2 | 61697858 | 61697858 | T | C | het | . | . | . | 75 | 0 | 0% | T | 16 | 2 | 11.11% | Y | Somatic | 0.0357644 |
|  | exonic | SLAIN2 | nonsynonymous SNV | NM_020846:c.A1349G:p.Q450R | 654;Name=lod=609 |  |  |  |  |  | 0.15 |  |  |  |  | chr4 | 48385790 | 48385790 | A | G | het | . | . | . | 62 | 0 | 0% | A | 24 | 3 | 11.11% | R | Somatic | 0.0257564 |
|  | exonic | C5orf35 | nonsynonymous SNV | NM_001171990:c.T67C:p.W23R | 445;Name=lod=86 |  |  |  |  |  | 0 | 0.999 | 0.8896 | 0.971375 | 1 | chr5 | 56205539 | 56205539 | T | C | het | . | . | . | 72 | 0 | 0% | T | 16 | 2 | 11.11% | Y | Somatic | 0.0382022 |
|  | exonic | DND1 | synonymous SNV | NM_194249:c.A135C:p.P45P | 578;Name=lod=297 | 0.95 |  |  |  |  |  |  |  |  |  | chr5 | 140052863 | 140052863 | T | G | het | . | . | . | 179 | 3 | 1.65% | T | 80 | 10 | 11.11% | K | Somatic | 0.0011937 |
|  | exonic | KLF14 | nonsynonymous SNV | NM_138693:c.C749T:p.T250M | 601;Name=lod=371 |  |  |  |  |  | 0 | 0.999 | 0.999238 | 0.770332 | 0.997895 | chr7 | 130418112 | 130418112 | G | A | het | . | . | . | 65 | 0 | 0% | G | 16 | 2 | 11.11% | R | Somatic | 0.0449603 |
|  | exonic | GADD45G | nonsynonymous SNV | NM_006705:c.C412T:p.L138F | 569;Name=lod=275 |  |  |  |  |  | 0.01 | 0.547 | 0.998168 | 0.999972 | 1 | chr9 | 92220926 | 92220926 | C | T | het | . | . | . | 63 | 0 | 0% | C | 16 | 2 | 11.11% | Y | Somatic | 0.0472222 |
|  | exonic | TUBB8 | nonsynonymous SNV | NM_177987:c.G97T:p.A33S | 583;Name=lod=311 | 0.97 |  |  |  |  | 0.09 | 0 | 0.833287 | 5.57E-04 | 0.967179 | chr10 | 94813 | 94813 | C | A | het | . | . | . | 57 | 0 | 0% | C | 24 | 3 | 11.11% | M | Somatic | 0.0306977 |
|  | exonic | ADAMTS14 | nonsynonymous SNV | NM_080722:c.A3547C:p.T1183P |  |  |  |  |  |  | 0.03 | 0 | 0.774072 | 0.166821 | 0.959002 | chr10 | 72520484 | 72520484 | A | C | het | . | . | . | 61 | 1 | 1.61% | A | 64 | 8 | 11.11% | M | Somatic | 0.0283564 |
|  | intronic | MUC6 |  |  |  |  |  |  |  |  |  |  |  |  |  | chr11 | 1027540 | 1027540 | A | C | het | . | . | . | 62 | 0 | 0% | A | 16 | 2 | 11.11% | M | Somatic | 0.0484177 |
|  | exonic | VEGFB | nonsynonymous SNV | NM_003377:c.A428C:p.H143P | 507;Name=lod=153 |  |  |  |  |  | 0.09 | 0.676 | 0.996742 | 0.166734 | 2.78E-04 | chr11 | 64004909 | 64004909 | A | C | het | . | . | . | 57 | 0 | 0% | A | 32 | 4 | 11.11% | M | Somatic | 0.0201748 |
|  | intronic | CIITA |  |  |  |  |  |  |  |  |  |  |  |  |  | chr16 | 11000329 | 11000329 | A | C | het | . | . | . | 45 | 0 | 0% | A | 32 | 4 | 11.11% | M | Somatic | 0.0354052 |
|  | exonic | ASPHD1 | nonsynonymous SNV | NM_181718:c.C382G:p.P128A |  |  |  |  |  |  | 0.07 | 0.028 | 0.866042 | 0.001084 | 0.909952 | chr16 | 29912674 | 29912674 | C | G | het | . | . | . | 95 | 1 | 1.04% | C | 32 | 4 | 11.11% | S | Somatic | 0.0195005 |
|  | intronic | GNAO1 |  |  | 265;Name=lod=16 |  |  |  |  |  |  |  |  |  |  | chr16 | 56226291 | 56226291 | T | C | het | . | . | . | 49 | 0 | 0% | T | 24 | 3 | 11.11% | Y | Somatic | 0.0416074 |
|  | exonic | GPRC5C | nonsynonymous SNV | NM_022036:c.T67G:p.S23A |  |  |  |  |  |  | 0 | 0.373142 | 0.953122 | 0.008114 | 0.001955 | chr17 | 72428244 | 72428244 | T | G | het | . | . | . | 63 | 0 | 0% | T | 16 | 2 | 11.11% | K | Somatic | 0.0472222 |
|  | exonic | KIAA0195 | nonsynonymous SNV | NM_014738:c.A742C:p.T248P | 385;Name=lod=49 |  |  |  |  |  | 0 | 0.97 | 0.997872 | 0.999946 | 1 | chr17 | 73484969 | 73484969 | A | C | het | . | . | . | 58 | 1 | 1.69% | A | 80 | 10 | 11.11% | M | Somatic | 0.0274853 |
|  | exonic | GNAL | nonsynonymous SNV | NM_002071:c.A1040C:p.Y347S | 478;Name=lod=117 |  |  |  |  |  |  | 1 | 0.998433 | 0.99999 | 1 | chr18 | 11881028 | 11881028 | A | C | het | . | . | . | 62 | 0 | 0% | A | 32 | 4 | 11.11% | M | Somatic | 0.0163069 |
|  | exonic | SMOX | synonymous SNV | NM_175839:c.T888G:p.G296G |  |  |  |  |  |  |  |  |  |  |  | chr20 | 4163014 | 4163014 | T | G | het | . | . | . | 89 | 1 | 1.11% | T | 24 | 3 | 11.11% | K | Somatic | 0.0378758 |
|  | exonic | RRP1 | nonsynonymous SNV | NM_003683:c.T190G:p.W64G | 485;Name=lod=125 |  |  |  |  |  | 0 | 1 | 0.995301 | 0.999988 | 1 | chr21 | 45211287 | 45211287 | T | G | het | . | . | . | 63 | 1 | 1.56% | T | 56 | 7 | 11.11% | K | Somatic | 0.0293075 |
|  | intronic | STARD8 |  |  |  |  |  |  |  |  |  |  |  |  |  | chrX | 67939043 | 67939043 | A | C | het | . | . | . | 43 | 0 | 0% | A | 32 | 4 | 11.11% | M | Somatic | 0.0392046 |
|  | exonic | DCAF12L1 | nonsynonymous SNV | NM_178470:c.T179G:p.V60G |  |  |  |  |  |  | 0 | 0.76 | 0.952688 | 0.002478 | 0.857055 | chrX | 125686413 | 125686413 | A | C | het | . | . | . | 149 | 3 | 1.97% | A | 48 | 6 | 11.11% | M | Somatic | 0.0110348 |
|  | exonic | PDZD4 | nonsynonymous SNV | NM_032512:c.T392G:p.V131G | 588;Name=lod=326 |  |  |  |  |  | 0 | 0.998 | 0.998367 | 0.999512 | 1 | chrX | 153072291 | 153072291 | A | C | het | . | . | . | 75 | 1 | 1.32% | A | 40 | 5 | 11.11% | M | Somatic | 0.026279 |
|  | UTR5 | ANKRD42 |  |  |  |  |  |  |  |  | 0.02 |  |  |  |  | chr11 | 82905704 | 82905704 | T | G | het | . | . | . | 61 | 1 | 1.61% | T | 73 | 9 | 10.98% | K | Somatic | 0.0264618 |
|  | intergenic | ANKRD62P1-PARP4P3(dist=16391),XKR3(dist=91485) |  |  |  | 1 |  |  |  |  |  |  |  |  |  | chr22 | 17172821 | 17172821 | G | T | het | . | . | . | 44 | 0 | 0% | G | 57 | 7 | 10.94% | K | Somatic | 0.0222792 |
|  | UTR3 | KIF13A |  |  |  |  |  |  |  |  |  |  |  |  |  | chr6 | 17761076 | 17761076 | T | G | het | . | . | . | 39 | 0 | 0% | T | 49 | 6 | 10.91% | K | Somatic | 0.0356044 |
|  | intronic | PDIA2 |  |  |  |  |  |  |  |  |  |  |  |  |  | chr16 | 336497 | 336497 | T | G | het | . | . | . | 135 | 2 | 1.46% | T | 41 | 5 | 10.87% | K | Somatic | 0.0116181 |
|  | exonic | UMODL1 | nonsynonymous SNV | NM_001199527:c.A2260C:p.T754P |  |  | 0.19 | 0.19 | 0.19 | rs220130 | 0.04 | 0.205 | 0.038104 | 0.003861 | 0.231022 | chr21 | 43531808 | 43531808 | A | C | het | . | . | . | 62 | 1 | 1.59% | A | 41 | 5 | 10.87% | M | Somatic | 0.0472707 |
|  | exonic | ZNF496 | nonsynonymous SNV | NM_032752:c.T1703C:p.L568P | 505;Name=lod=151 |  |  |  |  |  | 0 | 0.184 | 0.997761 | 0.316738 | 0.999764 | chr1 | 247463882 | 247463882 | A | G | het | . | . | . | 112 | 1 | 0.88% | A | 66 | 8 | 10.81% | R | Somatic | 0.0028618 |
|  | splicing | PKHD1L1(NM_177531:exon76:c.12331-2A>G) |  |  | 515;Name=lod=165 |  |  |  |  |  |  |  |  |  |  | chr8 | 110535460 | 110535460 | A | G | het | . | . | . | 109 | 1 | 0.91% | A | 66 | 8 | 10.81% | R | Somatic | 0.0032381 |
|  | intronic | ATM |  |  |  |  |  |  |  |  |  |  |  |  |  | chr11 | 108196734 | 108196734 | A | T | het | . | . | . | 64 | 0 | 0% | A | 33 | 4 | 10.81% | W | Somatic | 0.0161759 |
|  | exonic | KCNG4 | nonsynonymous SNV | NM_172347:c.A568C:p.T190P |  |  |  |  |  |  | 0.24 | 0.059 | 0.88572 | 0.008666 | 1.27E-04 | chr16 | 84270524 | 84270524 | T | G | het | . | . | . | 104 | 2 | 1.89% | T | 33 | 4 | 10.81% | K | Somatic | 0.0389613 |
|  | exonic | CHRFAM7A | nonsynonymous SNV | NM_148911:c.A413C:p.H138P | 516;Name=lod=167 | 1 |  |  |  |  | 0 | 0.998 | 0.978324 | 0.999856 | 1 | chr15 | 30659655 | 30659655 | T | G | het | . | . | . | 37 | 0 | 0% | T | 58 | 7 | 10.77% | K | Somatic | 0.0376993 |
|  | exonic | TOR2A | nonsynonymous SNV | NM_001085347:c.T386C:p.F129S | 422;Name=lod=69 |  |  |  |  | rs74960400 | 0 | 1 | 0.9987 | 0.999973 | 1 | chr9 | 130496609 | 130496609 | A | G | het | . | . | . | 69 | 0 | 0% | A | 25 | 3 | 10.71% | R | Somatic | 0.0222192 |
|  | exonic | INCENP | nonsynonymous SNV | NM_020238:c.T1664G:p.V555G | 574;Name=lod=287 |  |  |  |  |  | 0 | 0.455815 | 0.998329 | 0.786144 | 0.998636 | chr11 | 61912524 | 61912524 | T | G | het | . | . | . | 88 | 1 | 1.12% | T | 50 | 6 | 10.71% | K | Somatic | 0.0135236 |
|  | intronic | GATC |  |  |  |  |  |  |  |  |  |  |  |  |  | chr12 | 120884437 | 120884437 | T | C | het | . | . | . | 53 | 0 | 0% | T | 25 | 3 | 10.71% | Y | Somatic | 0.0383966 |
|  | ncRNA_exonic | SLC7A5P1 |  |  | 731;Name=lod=1243 | 0.98 |  |  |  |  |  |  |  |  |  | chr16 | 29624745 | 29624745 | T | G | het | . | . | . | 54 | 0 | 0% | T | 25 | 3 | 10.71% | K | Somatic | 0.0369919 |
|  | exonic | PLEKHM1 | nonsynonymous SNV | NM_014798:c.T1907G:p.V636G | 405;Name=lod=59 | 0.97 |  |  |  |  | 0.01 | 0.707 | 0.998125 | 0.697279 | 0.999999 | chr17 | 43531311 | 43531311 | A | C | het | . | . | . | 68 | 0 | 0% | A | 25 | 3 | 10.71% | M | Somatic | 0.0229283 |
|  | intronic | COL6A1 |  |  |  |  |  |  |  |  |  |  |  |  |  | chr21 | 47412061 | 47412061 | A | C | het | . | . | . | 60 | 0 | 0% | A | 25 | 3 | 10.71% | M | Somatic | 0.0298535 |
|  | exonic | EDA | nonsynonymous SNV | NM_001005609:c.T226G:p.S76A | 346;Name=lod=34 |  |  |  |  |  | 0.26 | 0.553619 | 0.95822 | 0.003361 | 0.978945 | chrX | 68836378 | 68836378 | T | G | het | . | . | . | 59 | 0 | 0% | T | 25 | 3 | 10.71% | K | Somatic | 0.0309071 |
|  | exonic | HEXA | synonymous SNV | NM_000520:c.A1383G:p.G461G | 608;Name=lod=395 |  |  |  |  |  |  |  |  |  |  | chr15 | 72638614 | 72638614 | T | C | het | . | . | . | 59 | 1 | 1.67% | T | 67 | 8 | 10.67% | Y | Somatic | 0.0364057 |
|  | exonic | VCPIP1 | nonsynonymous SNV | NM_025054:c.T263G:p.V88G | 736;Name=lod=1309 |  |  |  |  |  | 0 | 0.992 | 0.99917 | 0.925996 | 1 | chr8 | 67578931 | 67578931 | A | C | het | . | . | . | 68 | 1 | 1.45% | A | 42 | 5 | 10.64% | M | Somatic | 0.0392893 |
|  | exonic | VPS37B | synonymous SNV | NM_024667:c.T597C:p.P199P |  |  |  |  |  |  |  |  |  |  |  | chr12 | 123351924 | 123351924 | A | G | het | . | . | . | 66 | 1 | 1.49% | A | 42 | 5 | 10.64% | R | Somatic | 0.0425627 |
|  | intergenic | LINC00221(dist=218564),NONE(dist=NONE) |  |  | 414;Name=lod=64 |  |  |  |  |  |  |  |  |  |  | chr14 | 107170093 | 107170093 | C | T | het | . | . | . | 389 | 1 | 0.26% | C | 462 | 55 | 10.64% | Y | Somatic | 2.65E-13 |
|  | splicing | MBD1(NM_001204139:exon15:c.1716+2T>G,NM_001204141:exon14:c.1566+2T>G,NM_001204142:exon14:c.1578+2T>G,NM_001204136:exon15:c.1716+2T>G,NM_001204143:exon13:c.1410+2T>G,NM_002384:exon13:c.1410+2T>G,NM_015844:exon14:c.1548+2T>G,NM_015847:exon14:c.1569+2T>G,NM_015846:exon15:c.1716+2T>G,NM_015845:exon13:c.1509+2T>G,NM_001204137:exon16:c.1791+2T>G,NM_001204138:exon16:c.1788+2T>G,NM_001204140:exon15:c.1623+2T>G,NM_001204151:exon12:c.1509+2T>G) |  |  | 419;Name=lod=67 |  |  |  |  |  |  |  |  |  |  | chr18 | 47799192 | 47799192 | A | C | het | . | . | . | 71 | 1 | 1.39% | A | 101 | 12 | 10.62% | M | Somatic | 0.0127097 |
|  | ncRNA_intronic | TPTE2P1 |  |  |  | 0.96 |  |  |  |  |  |  |  |  |  | chr13 | 25510727 | 25510727 | T | A | het | . | . | . | 63 | 1 | 1.56% | T | 59 | 7 | 10.61% | W | Somatic | 0.0342241 |
|  | exonic | SNX33 | nonsynonymous SNV | NM_153271:c.A452C:p.Y151S | 459;Name=lod=98 |  |  |  |  |  | 0.4 | 0.019 | 0.972396 | 0.99404 | 0.999988 | chr15 | 75941895 | 75941895 | A | C | het | . | . | . | 82 | 1 | 1.20% | A | 59 | 7 | 10.61% | M | Somatic | 0.0141422 |
|  | ncRNA_intronic | LOC100505666 |  |  | 272;Name=lod=17 |  |  |  |  |  |  |  |  |  |  | chr1 | 155025869 | 155025869 | T | G | het | . | . | . | 64 | 1 | 1.54% | T | 76 | 9 | 10.59% | K | Somatic | 0.0255582 |
|  | intronic | MEGF6 |  |  |  |  |  |  |  |  |  |  |  |  |  | chr1 | 3427526 | 3427526 | A | C | het | . | . | . | 66 | 0 | 0% | A | 17 | 2 | 10.53% | M | Somatic | 0.0478992 |
|  | exonic | KBTBD5 | nonsynonymous SNV | NM_152393:c.T188A:p.L63Q | 620;Name=lod=440 |  |  |  |  |  | 0 | 0.548 | 0.996742 | 0.999965 | 1 | chr3 | 42727298 | 42727298 | T | A | het | . | . | . | 67 | 0 | 0% | T | 17 | 2 | 10.53% | W | Somatic | 0.0467852 |
|  | exonic | MFSD10 | nonsynonymous SNV | NM_001120:c.T203G:p.V68G | 420;Name=lod=68 |  |  |  |  |  | 0 | 0.994 | 0.994988 | 0.998533 | 1 | chr4 | 2935359 | 2935359 | A | C | het | . | . | . | 71 | 1 | 1.39% | A | 34 | 4 | 10.53% | M | Somatic | 0.0475247 |
|  | exonic | SLC12A7 | nonsynonymous SNV | NM_006598:c.A902C:p.D301A | 641;Name=lod=539 |  |  |  |  |  | 0.93 | 0 | 0.864802 | 0.101669 | 0.999956 | chr5 | 1085362 | 1085362 | T | G | het | . | . | . | 69 | 0 | 0% | T | 17 | 2 | 10.53% | K | Somatic | 0.0446708 |
|  | intronic | PLEC |  |  |  |  |  |  |  |  |  |  |  |  |  | chr8 | 145003762 | 145003762 | T | C | het | . | . | . | 74 | 0 | 0% | T | 17 | 2 | 10.53% | Y | Somatic | 0.039972 |
|  | exonic | JAG2 | nonsynonymous SNV | NM_145159:c.T1961C:p.L654P | 473;Name=lod=112 |  |  |  |  |  | 0.02 | 0.995 | 0.995301 | 0.809919 | 0.999998 | chr14 | 105614722 | 105614722 | A | G | het | . | . | . | 76 | 0 | 0% | A | 17 | 2 | 10.53% | R | Somatic | 0.0382979 |
|  | splicing | RPUSD1 |  |  |  |  |  |  |  |  |  |  |  |  |  | chr16 | 837743 | 837743 | G | C | het | . | . | . | 87 | 0 | 0% | G | 17 | 2 | 10.53% | S | Somatic | 0.0307278 |
|  | intronic | NOTCH3 |  |  |  |  |  |  |  |  |  |  |  |  |  | chr19 | 15300028 | 15300028 | A | C | het | . | . | . | 122 | 1 | 0.81% | A | 51 | 6 | 10.53% | M | Somatic | 0.00438 |
|  | intronic | FAM83C |  |  |  |  |  |  |  |  |  |  |  |  |  | chr20 | 33876413 | 33876413 | A | C | het | . | . | . | 46 | 0 | 0% | A | 34 | 4 | 10.53% | M | Somatic | 0.038256 |
|  | ncRNA_intronic | LOC100287944 |  |  |  |  |  |  |  |  |  |  |  |  |  | chr12 | 107080631 | 107080631 | C | T | het | . | . | . | 59 | 1 | 1.67% | C | 103 | 12 | 10.43% | Y | Somatic | 0.028523 |
|  | intronic | IFNGR1 |  |  |  |  |  |  |  |  | 0 |  |  |  |  | chr6 | 137524836 | 137524836 | G | A | het | . | . | . | 71 | 0 | 0% | G | 43 | 5 | 10.42% | R | Somatic | 0.0093754 |
|  | exonic | GPER | nonsynonymous SNV | NM_001098201:c.T100C:p.S34P |  |  |  |  |  |  | 0.06 | 0 | 0.096821 | 0.086684 | 0.943365 | chr7 | 1131464 | 1131464 | T | C | het | . | . | . | 99 | 2 | 1.98% | T | 43 | 5 | 10.42% | Y | Somatic | 0.0355233 |
|  | intronic | SKA2 |  |  |  |  |  |  |  |  |  |  |  |  |  | chr17 | 57232262 | 57232262 | A | C | het | . | . | . | 58 | 1 | 1.69% | A | 86 | 10 | 10.42% | M | Somatic | 0.034867 |
|  | exonic | PDE6B | nonsynonymous SNV | NM_000283:c.A50T:p.D17V |  |  |  |  |  |  | 0.3 | 0 | 0.113493 | 0.032658 | 0.728911 | chr4 | 619465 | 619465 | A | T | het | . | . | . | 159 | 3 | 1.85% | A | 52 | 6 | 10.34% | W | Somatic | 0.0114926 |
|  | exonic | TRIL | nonsynonymous SNV | NM_014817:c.C495A:p.N165K | 498;Name=lod=141 |  |  |  |  |  |  |  |  |  |  | chr7 | 28997168 | 28997168 | G | T | het | . | . | . | 61 | 0 | 0% | G | 26 | 3 | 10.34% | K | Somatic | 0.0311032 |
|  | exonic | ADAM8 | nonsynonymous SNV | NM_001164490:c.A1648C:p.T550P | 333;Name=lod=30 |  |  |  |  |  | 0 | 0.616998 | 0.939191 | 0.651431 | 0.970281 | chr10 | 135083884 | 135083884 | T | G | het | . | . | . | 73 | 0 | 0% | T | 26 | 3 | 10.34% | K | Somatic | 0.0212813 |
|  | exonic | PKD1 | nonsynonymous SNV | NM_000296:c.A7717C:p.T2573P |  | 0.98 |  |  |  |  | 0.08 | 0.472 | 0.010256 | 1.94E-04 | 0.09665 | chr16 | 2156012 | 2156012 | T | G | het | . | . | . | 91 | 0 | 0% | T | 26 | 3 | 10.34% | K | Somatic | 0.013011 |
|  | intronic | LOC653550,TP53TG3,TP53TG3B |  |  |  | 1 |  |  |  |  |  |  |  |  |  | chr16 | 33206023 | 33206023 | A | C | het | . | . | . | 99 | 2 | 1.98% | A | 52 | 6 | 10.34% | M | Somatic | 0.02794 |
|  | intronic | UPF1 |  |  |  |  |  |  |  |  |  |  |  |  |  | chr19 | 18966066 | 18966066 | G | A | het | . | . | . | 71 | 0 | 0% | G | 26 | 3 | 10.34% | R | Somatic | 0.0225974 |
|  | exonic | TMEM143 | nonsynonymous SNV | NM_018273:c.A1319C:p.D440A | 329;Name=lod=29 |  |  |  |  |  | 0.01 | 0.186 | 0.996681 | 0.615656 | 0.999496 | chr19 | 48836537 | 48836537 | T | G | het | . | . | . | 50 | 0 | 0% | T | 52 | 6 | 10.34% | K | Somatic | 0.0211519 |
|  | intronic | SULT1C4 |  |  |  |  |  |  |  |  |  |  |  |  |  | chr2 | 108999519 | 108999519 | C | T | het | . | . | . | 64 | 1 | 1.54% | C | 87 | 10 | 10.31% | Y | Somatic | 0.0256785 |
|  | exonic | PCDHB16 | synonymous SNV | NM_020957:c.T1620G:p.A540A | 436;Name=lod=79 | 0.95 | 0.07 | 0.07 | 0.07 | rs17844656 |  |  |  |  |  | chr5 | 140563754 | 140563754 | T | G | het | . | . | . | 174 | 1 | 0.57% | T | 61 | 7 | 10.29% | K | Somatic | 6.60E-04 |
|  | exonic | SBF1 | nonsynonymous SNV | NM_002972:c.T431G:p.V144G | 463;Name=lod=102 |  |  |  |  |  | 0 | 0.215 | 0.970558 | 0.997157 | 0.999999 | chr22 | 50905968 | 50905968 | A | C | het | . | . | . | 128 | 2 | 1.54% | A | 61 | 7 | 10.29% | M | Somatic | 0.008527 |
|  | exonic | TLE1 | nonsynonymous SNV | NM_005077:c.T2048G:p.V683G | 429;Name=lod=74 |  |  |  |  |  | 0 | 0.998 | 0.998561 | 0.999996 | 1 | chr9 | 84200500 | 84200500 | A | C | het | . | . | . | 66 | 1 | 1.49% | A | 96 | 11 | 10.28% | M | Somatic | 0.0213031 |
|  | exonic | HSPA6 | nonsynonymous SNV | NM_002155:c.T158G:p.V53G | 521;Name=lod=175 | 0.98 |  |  |  |  |  | 0.995 | 0.987469 | 0.009025 | 0.967561 | chr1 | 161494606 | 161494606 | T | G | het | . | . | . | 102 | 1 | 0.97% | T | 35 | 4 | 10.26% | K | Somatic | 0.0201923 |
|  | UTR5 | POLR1B |  |  |  |  |  |  |  |  |  |  |  |  |  | chr2 | 113300057 | 113300057 | T | G | het | . | . | . | 87 | 0 | 0% | T | 35 | 4 | 10.26% | K | Somatic | 0.0082176 |
|  | exonic | CLIP2 | synonymous SNV | NM_003388:c.C672T:p.R224R |  |  |  |  |  |  |  |  |  |  |  | chr7 | 73753328 | 73753328 | C | T | het | . | . | . | 66 | 0 | 0% | C | 35 | 4 | 10.26% | Y | Somatic | 0.0172065 |
|  | exonic | GBX1 | synonymous SNV | NM_001098834:c.A495C:p.P165P | 542;Name=lod=213 |  |  |  |  |  |  |  |  |  |  | chr7 | 150864141 | 150864141 | T | G | het | . | . | . | 103 | 2 | 1.90% | T | 35 | 4 | 10.26% | K | Somatic | 0.0460191 |
|  | exonic | VPS4A | nonsynonymous SNV | NM_013245:c.T626G:p.V209G | 623;Name=lod=452 |  |  |  |  |  | 0 |  |  |  |  | chr16 | 69354049 | 69354049 | T | G | het | . | . | . | 47 | 0 | 0% | T | 35 | 4 | 10.26% | K | Somatic | 0.0387327 |
|  | intronic | PCNT |  |  |  |  |  |  |  |  |  |  |  |  |  | chr21 | 47845734 | 47845734 | T | C | het | . | . | . | 73 | 1 | 1.35% | T | 35 | 4 | 10.26% | Y | Somatic | 0.0474645 |
|  | exonic | PANX2 | nonsynonymous SNV | NM_001160300:c.T824G:p.V275G | 470;Name=lod=108 |  |  |  |  |  | 0 | 0.83 | 0.993588 | 0.375565 | 0.997404 | chr22 | 50615965 | 50615965 | T | G | het | . | . | . | 109 | 2 | 1.80% | T | 35 | 4 | 10.26% | K | Somatic | 0.0398205 |
|  | intronic | SYNE2 |  |  |  |  |  |  |  |  |  |  |  |  |  | chr14 | 64688454 | 64688454 | A | C | het | . | . | . | 75 | 1 | 1.32% | A | 114 | 13 | 10.24% | M | Somatic | 0.010936 |
|  | exonic | CHPF2 | synonymous SNV | NM_019015:c.A1905C:p.S635S | 387;Name=lod=50 |  |  |  |  |  |  |  |  |  |  | chr7 | 150935353 | 150935353 | A | C | het | . | . | . | 98 | 1 | 1.01% | A | 44 | 5 | 10.20% | M | Somatic | 0.0153932 |
|  | intergenic | TMEM121(dist=239158),KIAA0125(dist=148141) |  |  | 343;Name=lod=33 | 0.95 |  |  |  | rs13050 |  |  |  |  |  | chr14 | 106235697 | 106235697 | G | A | het | . | . | . | 1536 | 2 | 0.13% | G | 352 | 40 | 10.20% | R | Somatic | 2.25E-26 |
|  | exonic | IGF1R | nonsynonymous SNV | NM_000875:c.A695C:p.H232P | 552;Name=lod=234 |  |  |  |  |  | 0 | 0.883 | 0.998433 | 0.999975 | 0.999994 | chr15 | 99434608 | 99434608 | A | C | het | . | . | . | 67 | 1 | 1.47% | A | 44 | 5 | 10.20% | M | Somatic | 0.0459305 |
|  | exonic | DUSP4 | nonsynonymous SNV | NM_001394:c.T448G:p.F150V | 589;Name=lod=331 |  |  |  |  |  | 0 | 0.999 | 0.998893 | 0.999998 | 1 | chr8 | 29197746 | 29197746 | A | C | het | . | . | . | 46 | 0 | 0% | A | 53 | 6 | 10.17% | M | Somatic | 0.0279974 |
|  | splicing | VAV1(NM_005428:exon11:c.1092+2T>G) |  |  | 459;Name=lod=98 |  |  |  |  |  |  |  |  |  |  | chr19 | 6828500 | 6828500 | T | G | het | . | . | . | 45 | 0 | 0% | T | 62 | 7 | 10.14% | K | Somatic | 0.0262206 |
|  | exonic | GNA12 | nonsynonymous SNV | NM_007353:c.C970A:p.L324M | 460;Name=lod=99 |  |  |  |  |  | 0.04 | 0.003 | 0.970558 | 0.997611 | 0.998458 | chr7 | 2770991 | 2770991 | G | T | het | . | . | . | 60 | 1 | 1.64% | G | 71 | 8 | 10.13% | K | Somatic | 0.0410412 |
|  | exonic | PEX10 | nonsynonymous SNV | NM_002617:c.T491G:p.V164G |  |  |  |  |  |  | 0 | 0.161 | 0.008974 | 0.996238 | 0.883044 | chr1 | 2340000 | 2340000 | A | C | het | . | . | . | 77 | 0 | 0% | A | 27 | 3 | 10% | M | Somatic | 0.0204549 |
|  | exonic | C1orf187 | nonsynonymous SNV | NM_198545:c.A170C:p.H57P | 355;Name=lod=37 |  |  |  |  |  | 0 | 0.998 | 0.958982 | 0.835454 | 0.979773 | chr1 | 11766485 | 11766485 | A | C | het | . | . | . | 53 | 0 | 0% | A | 27 | 3 | 10% | M | Somatic | 0.0441876 |
|  | splicing | DARS(NM_001349:exon2:c.66+2T>G) |  |  | 560;Name=lod=251 |  |  |  |  |  |  |  |  |  |  | chr2 | 136742971 | 136742971 | A | C | het | . | . | . | 76 | 0 | 0% | A | 27 | 3 | 10% | M | Somatic | 0.021045 |
|  | intronic | ABTB1 |  |  |  |  |  |  |  |  |  |  |  |  |  | chr3 | 127396494 | 127396494 | A | C | het | . | . | . | 79 | 0 | 0% | A | 27 | 3 | 10% | M | Somatic | 0.0193394 |
|  | exonic | PLXND1 | nonsynonymous SNV | NM_015103:c.T2795G:p.V932G | 361;Name=lod=39 |  |  |  |  |  | 0 | 0.988 | 0.997777 | 0.999757 | 0.995316 | chr3 | 129292479 | 129292479 | A | C | het | . | . | . | 77 | 0 | 0% | A | 18 | 2 | 10% | M | Somatic | 0.0408076 |
|  | exonic | FAM46A | synonymous SNV | NM_017633:c.T147G:p.G49G |  |  |  |  |  |  |  |  |  |  |  | chr6 | 82461712 | 82461712 | A | C | het | . | . | . | 119 | 1 | 0.83% | A | 36 | 4 | 10% | M | Somatic | 0.01417 |
|  | intronic | FAM115C |  |  | 428;Name=lod=73 | 1 |  |  |  |  | 0 |  |  |  |  | chr7 | 143344560 | 143344560 | A | C | het | . | . | . | 44 | 0 | 0% | A | 36 | 4 | 10% | M | Somatic | 0.0473646 |
|  | exonic | TOX | nonsynonymous SNV | NM_014729:c.A1283C:p.H428P | 562;Name=lod=257 |  |  |  |  |  | 0 | 0.991 | 0.998514 | 0.982389 | 1 | chr8 | 59728006 | 59728006 | T | G | het | . | . | . | 66 | 0 | 0% | T | 63 | 7 | 10% | K | Somatic | 0.0082139 |
|  | exonic | NTNG2 | nonsynonymous SNV | NM_032536:c.A934C:p.T312P | 566;Name=lod=266 |  |  |  |  |  | 0 | 0.419 | 0.995973 | 0.776937 | 1 | chr9 | 135102312 | 135102312 | A | C | het | . | . | . | 53 | 0 | 0% | A | 27 | 3 | 10% | M | Somatic | 0.0441876 |
|  | exonic | LRRC32 | synonymous SNV | NM_001128922:c.A444C:p.A148A |  |  |  |  |  |  |  |  |  |  |  | chr11 | 76372193 | 76372193 | T | G | het | . | . | . | 86 | 0 | 0% | T | 36 | 4 | 10% | K | Somatic | 0.0091307 |
|  | UTR5 | PICALM |  |  | 623;Name=lod=453 |  |  |  |  |  |  |  |  |  |  | chr11 | 85779838 | 85779838 | A | C | het | . | . | . | 50 | 0 | 0% | A | 27 | 3 | 10% | M | Somatic | 0.0494158 |
|  | splicing | AICDA(NM_020661:exon5:c.428-1G>A) |  |  | 528;Name=lod=186 |  |  |  |  |  |  |  |  |  |  | chr12 | 8757519 | 8757519 | C | T | het | . | . | . | 102 | 2 | 1.92% | C | 72 | 8 | 10% | Y | Somatic | 0.0189411 |
|  | exonic | CKB | nonsynonymous SNV | NM_001823:c.G71T:p.S24I | 535;Name=lod=199 |  |  |  |  |  | 0.01 | 0.047 | 0.996831 | 0.999157 | 0.999926 | chr14 | 103988760 | 103988760 | C | A | het | . | . | . | 72 | 0 | 0% | C | 18 | 2 | 10% | M | Somatic | 0.0453894 |
|  | intronic | SEMA4B |  |  |  |  |  |  |  |  |  |  |  |  |  | chr15 | 90771104 | 90771104 | A | C | het | . | . | . | 56 | 0 | 0% | A | 36 | 4 | 10% | M | Somatic | 0.0275109 |
|  | exonic | WFIKKN1 | stopgain SNV | NM_053284:c.C673T:p.Q225X | 516;Name=lod=166 |  |  |  |  |  | 0.52 | 0.734425 | 0.997672 | 1 | 0.995733 | chr16 | 683083 | 683083 | C | T | het | . | . | . | 102 | 0 | 0% | C | 18 | 2 | 10% | Y | Somatic | 0.0257418 |
|  | intronic | CTU2 |  |  |  |  |  |  |  |  |  |  |  |  |  | chr16 | 88779677 | 88779677 | G | C | het | . | . | . | 61 | 0 | 0% | G | 27 | 3 | 10% | S | Somatic | 0.0334198 |
|  | intronic | AURKB |  |  |  |  |  |  |  |  |  |  |  |  |  | chr17 | 8110016 | 8110016 | A | C | het | . | . | . | 68 | 1 | 1.45% | A | 72 | 8 | 10% | M | Somatic | 0.0286268 |
|  | exonic | MED16 | synonymous SNV | NM_005481:c.C861T:p.A287A | 558;Name=lod=248 |  |  |  |  |  |  |  |  |  |  | chr19 | 885788 | 885788 | G | A | het | . | . | . | 79 | 0 | 0% | G | 18 | 2 | 10% | R | Somatic | 0.0391672 |
|  | exonic | S1PR5 | nonsynonymous SNV | NM_001166215:c.T32G:p.V11G | 490;Name=lod=131 |  |  |  |  |  | 0.06 | 0.254 | 0.997089 | 0.188153 | 0.016458 | chr19 | 10625656 | 10625656 | A | C | het | . | . | . | 77 | 0 | 0% | A | 27 | 3 | 10% | M | Somatic | 0.0204549 |
|  | intronic | DCAF15 |  |  |  |  |  |  |  |  |  |  |  |  |  | chr19 | 14070741 | 14070741 | C | G | het | . | . | . | 90 | 0 | 0% | C | 18 | 2 | 10% | S | Somatic | 0.0316931 |
|  | intronic | DCAF15 |  |  |  |  |  |  |  |  |  |  |  |  |  | chr19 | 14070742 | 14070742 | T | G | het | . | . | . | 90 | 0 | 0% | T | 18 | 2 | 10% | K | Somatic | 0.0316931 |
|  | exonic | CIC | synonymous SNV | NM_015125:c.G3240C:p.L1080L | 504;Name=lod=149 |  |  |  |  |  |  |  |  |  |  | chr19 | 42796782 | 42796782 | G | C | het | . | . | . | 88 | 0 | 0% | G | 27 | 3 | 10% | S | Somatic | 0.0152108 |
|  | exonic | PRMT1 | nonsynonymous SNV | NM_001207042:c.A544C:p.T182P | 581;Name=lod=306 |  |  |  |  |  | 0.02 | 0.731197 | 0.997562 | 0.986633 | 1 | chr19 | 50189392 | 50189392 | A | C | het | . | . | . | 84 | 1 | 1.18% | A | 36 | 4 | 10% | M | Somatic | 0.0359277 |
|  | exonic | KCNC3 | nonsynonymous SNV | NM_004977:c.G1190T:p.G397V | 666;Name=lod=676 |  |  |  |  |  | 0.09 | 0.945 | 0.991149 | 0.659986 | 0.989439 | chr19 | 50827020 | 50827020 | C | A | het | lung | carcinoma | adenocarcinoma | 115 | 2 | 1.71% | C | 63 | 7 | 10% | M | Somatic | 0.0146819 |
|  | intronic | BRSK1 |  |  |  |  |  |  |  |  |  |  |  |  |  | chr19 | 55819981 | 55819981 | G | T | het | . | . | . | 52 | 0 | 0% | G | 27 | 3 | 10% | K | Somatic | 0.0458446 |
|  | exonic | C20orf134 | nonsynonymous SNV | NM_001024675:c.C227T:p.A76V | 525;Name=lod=181 |  |  |  |  |  | 0.06 | 0 | 0.977198 | 0.008744 | 0.799925 | chr20 | 32255530 | 32255530 | C | T | het | . | . | . | 99 | 0 | 0% | C | 18 | 2 | 10% | Y | Somatic | 0.0270617 |
|  | exonic | LAMA5 | nonsynonymous SNV | NM_005560:c.T10589G:p.V3530G |  |  |  |  |  |  | 0.33 | 0.006 | 0.149613 | 0.130238 | 0.294623 | chr20 | 60885486 | 60885486 | A | C | het | . | . | . | 63 | 0 | 0% | A | 36 | 4 | 10% | M | Somatic | 0.0206705 |
|  | ncRNA_intronic | CHKB-CPT1B |  |  |  |  |  |  |  |  |  |  |  |  |  | chr22 | 51014576 | 51014576 | T | G | het | . | . | . | 59 | 1 | 1.67% | T | 72 | 8 | 10% | K | Somatic | 0.045013 |
|  | exonic | MAPK8IP2 | nonsynonymous SNV | NM_016431:c.C1586G:p.A529G |  |  |  |  |  |  |  |  |  |  |  | chr22 | 51043395 | 51043395 | C | G | het | . | . | . | 85 | 0 | 0% | C | 18 | 2 | 10% | S | Somatic | 0.0347985 |
|  | exonic | CCDC18 | nonsynonymous SNV | NM_206886:c.G113A:p.S38N | 583;Name=lod=311 |  |  |  |  |  | 0.33 | 0.425712 | 0.190972 | 0 | 0.789343 | chr1 | 93649031 | 93649031 | G | A | het | . | . | . | 185 | 0 | 0% | G | 110 | 4 | 3.51% | R | Somatic | 0.0204451 |
|  | exonic | TACC1 | nonsynonymous SNV | NM_001122824:c.G298A:p.A100T | 493;Name=lod=134 |  |  |  |  |  | 0.02 | 0.991 | 0.999692 | 0.983247 | 0.999998 | chr8 | 38684761 | 38684761 | G | A | het | . | . | . | 161 | 0 | 0% | G | 139 | 5 | 3.47% | R | Somatic | 0.0226019 |
|  | intronic | PLA2R1 |  |  |  |  |  |  |  |  |  |  |  |  |  | chr2 | 160879150 | 160879150 | T | C | het | . | . | . | 139 | 0 | 0% | T | 125 | 4 | 3.10% | Y | Somatic | 0.0523842 |
|  | exonic | SCYL2 | stopgain SNV | NM_017988:c.C298T:p.R100X | 704;Name=lod=967 |  |  |  |  |  | 1 | 0.731072 | 0.972712 | 1 | 1 | chr12 | 100685401 | 100685401 | C | T | het | . | . | . | 230 | 0 | 0% | C | 148 | 4 | 2.63% | Y | Somatic | 0.024473 |
|  | exonic | DHX15 | nonsynonymous SNV | NM_001358:c.C317T:p.A106V | 570;Name=lod=277 |  |  |  |  |  | 0.08 | 0.477729 | 0.999542 | 3.16E-04 | 0.997558 | chr4 | 24578056 | 24578056 | G | A | het | . | . | . | 269 | 0 | 0% | G | 242 | 6 | 2.42% | R | Somatic | 0.0118018 |
|  | exonic | LRRC23 | nonsynonymous SNV | NM_006992:c.G816A:p.M272I |  |  |  |  |  |  | 0.27 | 0.281136 | 0.00826 | 5.40E-05 | 0.578382 | chr12 | 7023112 | 7023112 | G | A | het | . | . | . | 250 | 0 | 0% | G | 180 | 4 | 2.17% | R | Somatic | 0.0317015 |
|  | exonic | RB1CC1 | nonsynonymous SNV | NM_001083617:c.G824A:p.G275D | 371;Name=lod=43 |  |  |  |  |  | 0.21 | 0.27 | 0.977717 | 0.981543 | 0.956507 | chr8 | 53586583 | 53586583 | C | T | het | . | . | . | 298 | 1 | 0.33% | C | 360 | 8 | 2.17% | Y | Somatic | 0.0384027 |
|  | exonic | PDE1A | synonymous SNV | NM_005019:c.T273C:p.S91S |  |  |  |  |  |  |  |  |  |  |  | chr2 | 183104962 | 183104962 | A | G | het | . | . | . | 235 | 0 | 0% | A | 193 | 4 | 2.03% | R | Somatic | 0.0425277 |
|  | exonic | API5 | nonsynonymous SNV | NM_001243747:c.G718A:p.V240I | 606;Name=lod=387 |  |  |  |  |  | 0.04 | 0.118 | 0.999658 | 0.76989 | 0.999961 | chr11 | 43352073 | 43352073 | G | A | het | . | . | . | 347 | 0 | 0% | G | 227 | 4 | 1.73% | R | Somatic | 0.025114 |
|  | exonic | ZNF765 | synonymous SNV | NM_001040185:c.T552C:p.P184P |  | 0.91 |  |  |  |  |  |  |  |  |  | chr19 | 53911360 | 53911360 | T | C | het | . | . | . | 342 | 0 | 0% | T | 251 | 4 | 1.57% | Y | Somatic | 0.0328376 |
|  | exonic | SACS | nonsynonymous SNV | NM_014363:c.G2741T:p.S914I | 541;Name=lod=210 |  |  |  |  |  | 0.01 | 0.024 | 0.999238 | 0.255359 | 0.999996 | chr13 | 23915274 | 23915274 | C | A | het | . | . | . | 318 | 0 | 0% | C | 253 | 4 | 1.56% | M | Somatic | 0.0393928 |
|  | exonic | TCF12 | nonsynonymous SNV | NM_207040:c.C1471T:p.P491S | 645;Name=lod=555 |  |  |  |  |  | 0.05 | 0.563 | 0.999121 | 0.99922 | 1 | chr15 | 57574717 | 57574717 | C | T | het | . | . | . | 413 | 0 | 0% | C | 374 | 5 | 1.32% | Y | Somatic | 0.0247491 |
|  | exonic | UBE3A | synonymous SNV | NM_130838:c.A2182C:p.R728R | 716;Name=lod=1085 |  |  |  |  |  |  |  |  |  |  | chr15 | 25599713 | 25599713 | T | G | het | . | . | . | 553 | 0 | 0% | T | 304 | 4 | 1.30% | K | Somatic | 0.0161707 |
|  | intronic | VPS13B |  |  | 713;Name=lod=1050 |  |  |  |  |  |  |  |  |  |  | chr8 | 100830893 | 100830893 | C | T | het | . | . | . | 510 | 0 | 0% | C | 336 | 4 | 1.18% | Y | Somatic | 0.0253291 |
|  | exonic | FAM55D | synonymous SNV | NM_001077639:c.G1119A:p.L373L | 300;Name=lod=22 |  |  |  |  |  |  |  |  |  |  | chr11 | 114442176 | 114442176 | C | T | het | . | . | . | 464 | 0 | 0% | C | 366 | 4 | 1.08% | Y | Somatic | 0.038389 |
|  | exonic | DACH1 | nonsynonymous SNV | NM_004392:c.C1348T:p.R450W | 669;Name=lod=697 |  |  |  |  |  | 0 | 0.788562 | 0.999553 | 0.997701 | 1 | chr13 | 72049904 | 72049904 | G | A | het | . | . | . | 735 | 0 | 0% | G | 475 | 5 | 1.04% | R | Somatic | 0.0095023 |
|  | exonic | GIMAP1,GIMAP1-GIMAP5 | synonymous SNV | NM_001199577:c.G69A:p.R23R |  |  |  |  |  |  |  |  |  |  |  | chr7 | 150417161 | 150417161 | G | A | het | . | . | . | 802 | 0 | 0% | G | 594 | 6 | 1% | R | Somatic | 0.0060559 |
|  | exonic | AHNAK2 | nonsynonymous SNV | NM_138420:c.C7471T:p.P2491S | 415;Name=lod=65 | 0.91 |  |  |  |  | 0 |  |  |  |  | chr14 | 105414317 | 105414317 | G | A | het | large_intestine | carcinoma | adenocarcinoma | 981 | 1 | 0.10% | G | 424 | 4 | 0.93% | R | Somatic | 0.0318941 |
|  | exonic | HRNR | stopgain SNV | NM_001009931:c.G5623T:p.E1875X |  | 0.98 |  |  |  |  | 0.87 | 0.737221 | 0.995503 | 1 | 1 | chr1 | 152188482 | 152188482 | C | A | het | lung | carcinoma | adenocarcinoma | 4320 | 2 | 0.05% | C | 3280 | 7 | 0.21% | M | Somatic | 0.0392432 |
|  |  |  |  |  |  |  |  |  |  |  |  |  |  |  |  |  |  |  |  |  |  |  |  |  |  |  |  |  |  |  |  |  |  |  |

| **Table S7: The somatic SNV of lung tumor tissue.** | | | | | | | | |  |  |  |  |  |  |  |  |  |  |  |  |  |  |  |  |  |  |  |  |  |  |  |  |  |
| --- | --- | --- | --- | --- | --- | --- | --- | --- | --- | --- | --- | --- | --- | --- | --- | --- | --- | --- | --- | --- | --- | --- | --- | --- | --- | --- | --- | --- | --- | --- | --- | --- | --- |
| **Func** | **Gene** | **ExonicFunc** | **AAChange** | **Conserved** | **SegDup** | **1000G_ALL** | **1000G_ALL** | **1000G_ALL** | **dbSNP137** | **SIFT** | **PolyPhen2** | **LJB_PhyloP** | **LJB_MutationTaster** | **LJB_LRT** | **Chr** | **Start** | **End** | **Ref** | **Obs** | **Genotype** | **Cosmic** |  |  | **normal_reads1** | **normal_reads2** | **normal_var_freq** | **normal_gt** | **tumor_reads1** | **tumor_reads2** | **tumor_var_freq** | **tumor_gt** | **somatic_status** | **somatic_p_value** |
| exonic | PANK4 | synonymous SNV | NM_018216:c.C375A:p.G125G | 592;Name=lod=341 |  |  |  |  |  |  |  |  |  |  | chr1 | 2452587 | 2452587 | G | T | het | . | . | . | 322 | 0 | 0% | G | 245 | 4 | 1.61% | K | Somatic | 0.0356707 |
| intronic | KRI1 |  |  |  |  |  |  |  |  |  |  |  |  |  | chr19 | 10665950 | 10665950 | C | T | het | . | . | . | 75 | 0 | 0% | C | 59 | 20 | 25.32% | Y | Somatic | 4.16E-07 |
| exonic | KRI1 | nonsynonymous SNV | NM_023008:c.A1697G:p.Y566C | 583;Name=lod=311 |  |  |  |  |  | 0.04 | 0.992 | 0.996689 | 0.999621 | 0.999995 | chr19 | 10665964 | 10665964 | T | C | het | . | . | . | 70 | 0 | 0% | T | 50 | 13 | 20.63% | Y | Somatic | 2.93E-05 |
| exonic | UBE4B | nonsynonymous SNV | NM_006048:c.C1466T:p.T489I | 609;Name=lod=398 |  |  |  |  |  | 0 | 0.783304 | 0.999041 | 0.999359 | 1 | chr1 | 10190813 | 10190813 | C | T | het | . | . | . | 255 | 0 | 0% | C | 188 | 4 | 2.08% | Y | Somatic | 0.0334322 |
| exonic | KRI1 | nonsynonymous SNV | NM_023008:c.T1696C:p.Y566H | 583;Name=lod=311 |  |  |  |  |  | 0.35 | 0.103 | 0.997842 | 0.990452 | 0.999995 | chr19 | 10665965 | 10665965 | A | G | het | . | . | . | 70 | 0 | 0% | A | 50 | 13 | 20.63% | R | Somatic | 2.93E-05 |
| exonic | PLOD1 | synonymous SNV | NM_000302:c.G1467A:p.Q489Q | 385;Name=lod=49 |  |  |  |  |  |  |  |  |  |  | chr1 | 12024839 | 12024839 | G | A | het | . | . | . | 178 | 0 | 0% | G | 195 | 6 | 2.99% | R | Somatic | 0.0214735 |
| exonic | NBPF1 | synonymous SNV | NM_017940:c.C2820T:p.G940G |  | 0.96 |  |  |  |  |  |  |  |  |  | chr1 | 16893693 | 16893693 | G | A | het | . | . | . | 2304 | 1 | 0.04% | G | 939 | 4 | 0.42% | R | Somatic | 0.0271766 |
| exonic | SEPN1 | nonsynonymous SNV | NM_206926:c.C658T:p.R220W | 560;Name=lod=251 |  |  |  |  |  | 0.01 | 0.998 | 0.979584 | 0.927019 | 0.999975 | chr1 | 26135529 | 26135529 | C | T | het | . | . | . | 228 | 0 | 0% | C | 186 | 6 | 3.12% | Y | Somatic | 0.0087424 |
| intronic | WASF2 |  |  | 243;Name=lod=13 |  |  |  |  |  |  |  |  |  |  | chr1 | 27741509 | 27741509 | C | G | het | . | . | . | 111 | 1 | 0.89% | C | 40 | 4 | 9.09% | S | Somatic | 0.0225732 |
| exonic | AHDC1 | nonsynonymous SNV | NM_001029882:c.G1766A:p.R589Q | 489;Name=lod=130 |  |  |  |  | rs149970389 | 0 | 0.932 | 0.998873 | 0.074933 | 0.999962 | chr1 | 27876861 | 27876861 | C | T | het | . | . | . | 257 | 0 | 0% | C | 198 | 5 | 2.46% | Y | Somatic | 0.0162783 |
| exonic | ZNF17 | nonsynonymous SNV | NM_006959:c.G992A:p.G331E |  |  |  |  |  |  | 1 | 0.003 | 0.115337 | 0.029005 | 0.630749 | chr19 | 57931852 | 57931852 | G | A | het | . | . | . | 185 | 3 | 1.60% | G | 54 | 11 | 16.92% | R | Somatic | 3.00E-05 |
| intronic | NUPL1 |  |  |  |  |  |  |  |  |  |  |  |  |  | chr13 | 25899108 | 25899108 | A | T | het | . | . | . | 169 | 3 | 1.74% | A | 56 | 10 | 15.15% | W | Somatic | 2.13E-04 |
| exonic | ZNF345 | nonsynonymous SNV | NM_001242472:c.T710A:p.M237K | 420;Name=lod=68 |  |  |  |  |  | 1 | 0.048 | 0.205575 | 0.085889 | 0.665928 | chr19 | 37368442 | 37368442 | T | A | het | . | . | . | 188 | 3 | 1.57% | T | 62 | 11 | 15.07% | W | Somatic | 6.85E-05 |
| exonic | INPP5B | synonymous SNV | NM_005540:c.G720A:p.K240K | 476;Name=lod=115 |  |  |  |  |  |  |  |  |  |  | chr1 | 38355306 | 38355306 | C | T | het | . | . | . | 251 | 0 | 0% | C | 115 | 4 | 3.36% | Y | Somatic | 0.0103355 |
| exonic | ZNF432 | synonymous SNV | NM_014650:c.C1398T:p.F466F | 410;Name=lod=62 |  |  |  |  |  |  |  |  |  |  | chr19 | 52537534 | 52537534 | G | A | het | . | . | . | 205 | 2 | 0.97% | G | 57 | 10 | 14.93% | R | Somatic | 1.90E-05 |
| exonic | HRNR | synonymous SNV | NM_001009931:c.G8340A:p.G2780G |  | 0.98 |  |  |  |  |  |  |  |  |  | chr1 | 152185765 | 152185765 | C | T | het | . | . | . | 109 | 2 | 1.80% | C | 97 | 17 | 14.91% | Y | Somatic | 2.71E-04 |
| exonic | ZMPSTE24 | synonymous SNV | NM_005857:c.T651C:p.D217D | 684;Name=lod=806 |  | 0.11 | 0.11 | 0.11 | rs2076697 |  |  |  |  |  | chr1 | 40737589 | 40737589 | T | C | het | . | . | . | 259 | 0 | 0% | T | 123 | 7 | 5.38% | Y | Somatic | 4.17E-04 |
| exonic | FOXJ3 | synonymous SNV | NM_001198850:c.G75A:p.T25T | 820;Name=lod=2868 |  |  |  |  |  |  |  |  |  |  | chr1 | 42744313 | 42744313 | C | T | het | . | . | . | 288 | 0 | 0% | C | 139 | 4 | 2.80% | Y | Somatic | 0.0117794 |
| exonic | FOXD2 | synonymous SNV | NM_004474:c.G609C:p.L203L | 719;Name=lod=1112 |  |  |  |  |  |  |  |  |  |  | chr1 | 47904416 | 47904416 | G | C | het | . | . | . | 337 | 0 | 0% | G | 301 | 6 | 1.95% | S | Somatic | 0.011437 |
| splicing | C8B(NM_000066:exon6:c.534-2A>G) |  |  | 423;Name=lod=70 |  |  |  |  |  |  |  |  |  |  | chr1 | 57417855 | 57417855 | T | C | het | . | . | . | 243 | 0 | 0% | T | 193 | 5 | 2.53% | Y | Somatic | 0.0177383 |
| exonic | INF2 | nonsynonymous SNV | NM_001031714:c.A1672G:p.N558D | 272;Name=lod=17 |  |  |  |  |  | 0.12 |  |  |  |  | chr14 | 105174276 | 105174276 | A | G | het | . | . | . | 73 | 1 | 1.35% | A | 52 | 9 | 14.75% | R | Somatic | 0.0034876 |
| exonic | ZNF607 | nonsynonymous SNV | NM_001172677:c.C838T:p.H280Y |  |  |  |  |  |  | 1 | 0.002 | 0.811214 | 0.00387 | 0.817592 | chr19 | 38190191 | 38190191 | G | A | het | . | . | . | 198 | 3 | 1.49% | G | 58 | 10 | 14.71% | R | Somatic | 9.09E-05 |
| exonic | CCBL2 | synonymous SNV | NM_001008662:c.C633T:p.S211S | 607;Name=lod=389 |  | 0.4 | 0.4 | 0.4 | rs3738055 |  |  |  |  |  | chr1 | 89426902 | 89426902 | G | A | het | stomach | carcinoma | adenocarcinoma | 296 | 0 | 0% | G | 111 | 4 | 3.48% | R | Somatic | 0.0059005 |
| exonic | GBP2 | nonsynonymous SNV | NM_004120:c.G1472A:p.R491H |  |  | 0.002 | 0.002 | 0.002 | rs139053989 | 0.03 | 0.096 | 0.958316 | 0.063482 | 0.765017 | chr1 | 89575547 | 89575547 | C | T | het | . | . | . | 82 | 0 | 0% | C | 43 | 4 | 8.51% | Y | Somatic | 0.0162012 |
| exonic | ZNF326 | nonsynonymous SNV | NM_182976:c.G302C:p.S101T | 855;Name=lod=3972 |  |  |  |  |  | 0.49 | 0 | 0.99222 | 0.035641 | 0.999972 | chr1 | 90472996 | 90472996 | G | C | het | . | . | . | 580 | 0 | 0% | G | 299 | 6 | 1.97% | S | Somatic | 0.0016219 |
| exonic | ZNF432 | synonymous SNV | NM_014650:c.C1411A:p.R471R |  |  |  |  |  |  |  |  |  |  |  | chr19 | 52537521 | 52537521 | G | T | het | . | . | . | 192 | 2 | 1.03% | G | 53 | 9 | 14.52% | K | Somatic | 6.43E-05 |
| exonic | ZNF17 | synonymous SNV | NM_006959:c.C982A:p.R328R | 417;Name=lod=66 |  |  |  |  |  |  |  |  |  |  | chr19 | 57931842 | 57931842 | C | A | het | . | . | . | 183 | 2 | 1.08% | C | 59 | 10 | 14.49% | M | Somatic | 5.30E-05 |
| exonic | AMY2A | nonsynonymous SNV | NM_000699:c.C175T:p.P59S | 459;Name=lod=98 | 0.94 |  |  |  |  | 0 | 1 | 0.925532 | 0.999993 | 1 | chr1 | 104160582 | 104160582 | C | T | het | urinary_tract | carcinoma | NS | 563 | 1 | 0.18% | C | 171 | 4 | 2.29% | Y | Somatic | 0.0124636 |
| exonic | SLC25A24 | synonymous SNV | NM_013386:c.A1323G:p.P441P |  |  |  |  |  |  |  |  |  |  |  | chr1 | 108679386 | 108679386 | T | C | het | . | . | . | 215 | 0 | 0% | T | 74 | 4 | 5.13% | Y | Somatic | 0.0047415 |
| exonic | GSTM1 | nonsynonymous SNV | NM_000561:c.C41T:p.A14V |  | 0.96 |  |  |  |  | 0 | 0.003 | 0.874406 | 0.954289 | 0.999247 | chr1 | 110230796 | 110230796 | C | T | het | . | . | . | 418 | 0 | 0% | C | 551 | 6 | 1.08% | Y | Somatic | 0.0343608 |
| exonic | KCNA3 | nonsynonymous SNV | NM_002232:c.G877A:p.D293N | 617;Name=lod=431 |  |  |  |  |  | 0.04 | 0.013 | 0.99793 | 0.999358 | 1 | chr1 | 111216555 | 111216555 | C | T | het | ovary | carcinoma | serous_carcinoma | 307 | 0 | 0% | C | 280 | 5 | 1.75% | Y | Somatic | 0.0253893 |
| exonic | CEPT1 | stopgain SNV | NM_001007794:c.G594A:p.W198X | 696;Name=lod=902 |  |  |  |  |  | 0 | 0.735493 | 0.999592 | 1 | 1 | chr1 | 111703883 | 111703883 | G | A | het | . | . | . | 450 | 1 | 0.22% | G | 198 | 5 | 2.46% | R | Somatic | 0.0124579 |
| exonic | ZNF17 | synonymous SNV | NM_006959:c.T972C:p.H324H | 417;Name=lod=66 |  |  |  |  |  |  |  |  |  |  | chr19 | 57931832 | 57931832 | T | C | het | . | . | . | 175 | 1 | 0.57% | T | 61 | 10 | 14.08% | Y | Somatic | 2.01E-05 |
| exonic | HIPK1 | synonymous SNV | NM_152696:c.G63A:p.A21A | 718;Name=lod=1108 |  |  |  |  |  |  |  |  |  |  | chr1 | 114483068 | 114483068 | G | A | het | . | . | . | 534 | 0 | 0% | G | 430 | 8 | 1.83% | R | Somatic | 0.0016409 |
| exonic | TTF2 | nonsynonymous SNV | NM_003594:c.A499G:p.K167E |  |  | 0.49 | 0.49 | 0.49 | rs998532 | 0.26 | 0.012 | 0.829404 | 0.887461 | 0.990754 | chr1 | 117617705 | 117617705 | A | G | het | . | . | . | 279 | 0 | 0% | A | 159 | 6 | 3.64% | R | Somatic | 0.0024855 |
| exonic | TTF2 | synonymous SNV | NM_003594:c.C861T:p.N287N |  |  | 0.48 | 0.48 | 0.48 | rs1289673 |  |  |  |  |  | chr1 | 117618067 | 117618067 | C | T | het | . | . | . | 278 | 1 | 0.36% | C | 194 | 8 | 3.96% | Y | Somatic | 0.0050981 |
| exonic | ZNF432 | synonymous SNV | NM_014650:c.G1416T:p.L472L |  |  |  |  |  |  |  |  |  |  |  | chr19 | 52537516 | 52537516 | C | A | het | . | . | . | 185 | 2 | 1.07% | C | 50 | 8 | 13.79% | M | Somatic | 1.98E-04 |
| intronic | SPAG17,WDR3 |  |  |  |  |  |  |  |  |  |  |  |  |  | chr1 | 118499834 | 118499834 | C | A | het | . | . | . | 360 | 0 | 0% | C | 163 | 4 | 2.40% | M | Somatic | 0.0098371 |
| exonic | ZNF607 | synonymous SNV | NM_001172677:c.A837C:p.P279P |  |  |  |  |  |  |  |  |  |  |  | chr19 | 38190192 | 38190192 | T | G | het | . | . | . | 195 | 2 | 1.02% | T | 57 | 9 | 13.64% | K | Somatic | 8.97E-05 |
| exonic | PDE4DIP | nonsynonymous SNV | NM_001002811:c.G1960A:p.A654T | 519;Name=lod=171 |  |  |  |  |  | 0.01 | 0.33 | 0.986666 | 6.20E-05 | 0.844061 | chr1 | 144917633 | 144917633 | C | T | het | . | . | . | 583 | 1 | 0.17% | C | 256 | 4 | 1.54% | Y | Somatic | 0.033508 |
| exonic | PDE4DIP | nonsynonymous SNV | NM_001002811:c.T248A:p.L83Q | 590;Name=lod=333 |  | 0.17 | 0.17 | 0.17 | rs41315685 | 0 | 0.785799 | 0.99794 | 1 | 1 | chr1 | 144931461 | 144931461 | A | T | het | . | . | . | 400 | 0 | 0% | A | 245 | 5 | 2% | W | Somatic | 0.0082102 |
| exonic | ZNF432 | nonsynonymous SNV | NM_014650:c.G1412A:p.R471Q |  |  |  |  |  | rs183343359 | 0.61 | 0.999 | 0.752275 | 0.605165 | 0.923613 | chr19 | 52537520 | 52537520 | C | T | het | . | . | . | 191 | 3 | 1.55% | C | 52 | 8 | 13.33% | Y | Somatic | 5.88E-04 |
| exonic | RORC | nonsynonymous SNV | NM_001001523:c.G1063A:p.V355I | 515;Name=lod=165 |  |  |  |  |  | 0.01 | 0.493 | 0.9985 | 0.967377 | 0.999963 | chr1 | 151785763 | 151785763 | C | T | het | . | . | . | 364 | 0 | 0% | C | 525 | 7 | 1.32% | Y | Somatic | 0.0255986 |
| exonic | HRNR | synonymous SNV | NM_001009931:c.C3075T:p.G1025G |  | 0.98 |  |  |  |  |  |  |  |  |  | chr1 | 152191030 | 152191030 | G | A | het | . | . | . | 674 | 0 | 0% | G | 610 | 6 | 0.97% | R | Somatic | 0.0117057 |
| exonic | FLG2 | synonymous SNV | NM_001014342:c.C4530T:p.H1510H |  |  | 0.28 | 0.28 | 0.28 | rs12022217 |  |  |  |  |  | chr1 | 152325732 | 152325732 | G | A | het | . | . | . | 619 | 1 | 0.16% | G | 317 | 5 | 1.55% | R | Somatic | 0.0196975 |
| exonic | FLG2 | nonsynonymous SNV | NM_001014342:c.C3841T:p.H1281Y |  |  |  |  |  |  | 0 | 0.421592 | 0.992002 | 0.027662 | 0.861366 | chr1 | 152326421 | 152326421 | G | A | het | . | . | . | 503 | 0 | 0% | G | 204 | 5 | 2.39% | R | Somatic | 0.0021063 |
| exonic | FLG2 | nonsynonymous SNV | NM_001014342:c.G3419A:p.G1140D |  |  | 0.08 | 0.08 | 0.08 | rs78399057 | 0.32 | 0.257223 | 0.142223 | 0.08196 | 0.630378 | chr1 | 152326843 | 152326843 | C | T | het | stomach | carcinoma | adenocarcinoma | 496 | 1 | 0.20% | C | 311 | 5 | 1.58% | Y | Somatic | 0.0354573 |
| intronic | LCE1E |  |  |  | 0.96 |  |  |  |  |  |  |  |  |  | chr1 | 152759707 | 152759707 | G | A | het | . | . | . | 705 | 0 | 0% | G | 444 | 15 | 3.27% | R | Somatic | 7.53E-07 |
| exonic | S100A13 | synonymous SNV | NM_001024211:c.C108T:p.N36N |  |  |  |  |  |  |  |  |  |  |  | chr1 | 153598841 | 153598841 | G | A | het | . | . | . | 255 | 0 | 0% | G | 333 | 6 | 1.77% | R | Somatic | 0.0338968 |
| exonic | NUP210L | synonymous SNV | NM_001159484:c.C3963T:p.F1321F | 521;Name=lod=174 |  |  |  |  |  |  |  |  |  |  | chr1 | 153998177 | 153998177 | G | A | het | . | . | . | 300 | 0 | 0% | G | 247 | 5 | 1.98% | R | Somatic | 0.0194026 |
| exonic | ADAR | nonsynonymous SNV | NM_001025107:c.A266G:p.K89R |  |  | 0.59 | 0.59 | 0.59 | rs2229857 | 0.47 | 0.002 | 0.211334 | 0.002445 | 0.0101 | chr1 | 154573967 | 154573967 | T | C | het | . | . | . | 264 | 0 | 0% | T | 187 | 5 | 2.60% | Y | Somatic | 0.0128363 |
| exonic | POU2F1 | nonsynonymous SNV | NM_001198786:c.G1169A:p.R390H | 705;Name=lod=980 |  |  |  |  |  | 0 | 0.789113 | 0.999791 | 1 | 1 | chr1 | 167368511 | 167368511 | G | A | het | . | . | . | 322 | 1 | 0.31% | G | 204 | 5 | 2.39% | R | Somatic | 0.036935 |
| exonic | ZNF432 | nonsynonymous SNV | NM_014650:c.T1403G:p.L468W |  |  |  |  |  |  | 0.25 | 0.936 | 0.884397 | 0.690623 | 0.954841 | chr19 | 52537529 | 52537529 | A | C | het | . | . | . | 197 | 2 | 1.01% | A | 54 | 8 | 12.90% | M | Somatic | 2.08E-04 |
| splicing | GART(NM_001136006:exon19:c.2452+2T>G,NM_001136005:exon19:c.2452+2T>G,NM_000819:exon19:c.2452+2T>G) |  |  | 514;Name=lod=164 |  |  |  |  |  |  |  |  |  |  | chr21 | 34882088 | 34882088 | A | C | het | . | . | . | 81 | 1 | 1.22% | A | 55 | 8 | 12.70% | M | Somatic | 0.0056301 |
| exonic | HMCN1 | nonsynonymous SNV | NM_031935:c.G5383A:p.A1795T | 575;Name=lod=290 |  |  |  |  |  | 0.23 | 0.017 | 0.993877 | 0.996007 | 1 | chr1 | 185987397 | 185987397 | G | A | het | . | . | . | 314 | 1 | 0.32% | G | 108 | 4 | 3.57% | R | Somatic | 0.018093 |
| exonic | TPR | synonymous SNV | NM_003292:c.C1956T:p.S652S | 409;Name=lod=61 |  |  |  |  |  |  |  |  |  |  | chr1 | 186324833 | 186324833 | G | A | het | . | . | . | 268 | 1 | 0.37% | G | 137 | 4 | 2.84% | R | Somatic | 0.0496365 |
| splicing | ANKRD13B(NM_152345:exon2:c.250+2T>G) |  |  | 568;Name=lod=272 |  |  |  |  |  |  |  |  |  |  | chr17 | 27934897 | 27934897 | T | G | het | . | . | . | 111 | 2 | 1.77% | T | 62 | 9 | 12.68% | K | Somatic | 0.0034794 |
| exonic | F13B | nonsynonymous SNV | NM_001994:c.C1931T:p.P644L | 304;Name=lod=23 |  |  |  |  |  | 0 | 0.999 | 0.9998 | 0.995024 | 0.997192 | chr1 | 197009673 | 197009673 | G | A | het | . | . | . | 216 | 1 | 0.46% | G | 98 | 4 | 3.92% | R | Somatic | 0.0376935 |
| exonic | FAM58BP | nonsynonymous SNV | NM_001105517:c.C251T:p.A84V | 622;Name=lod=451 | 0.93 |  |  |  |  |  |  |  |  |  | chr1 | 200182942 | 200182942 | C | T | het | . | . | . | 334 | 1 | 0.30% | C | 226 | 5 | 2.16% | Y | Somatic | 0.0439801 |
| exonic | LSM3 | nonsynonymous SNV | NM_014463:c.A8G:p.D3G | 562;Name=lod=256 |  |  |  |  |  | 0.01 | 0.063 | 0.998703 | 0.999391 | 1 | chr3 | 14220368 | 14220368 | A | G | het | . | . | . | 86 | 1 | 1.15% | A | 49 | 7 | 12.50% | R | Somatic | 0.0060785 |
| UTR5 | AVPR1B |  |  |  |  |  |  |  |  |  |  |  |  |  | chr1 | 206224425 | 206224425 | C | T | het | . | . | . | 776 | 0 | 0% | C | 495 | 4 | 0.80% | Y | Somatic | 0.0232902 |
| exonic | AVPR1B | nonsynonymous SNV | NM_000707:c.C56T:p.A19V |  |  |  |  |  |  | 0.35 | 0 | 0.025408 | 0.002296 | 0.100018 | chr1 | 206224496 | 206224496 | C | T | het | . | . | . | 599 | 0 | 0% | C | 588 | 5 | 0.84% | Y | Somatic | 0.0302133 |
| exonic | MAPKAPK2 | nonsynonymous SNV | NM_004759:c.C553T:p.R185W | 484;Name=lod=124 |  |  |  |  |  | 0 | 1 | 0.998333 | 1 | 1 | chr1 | 206902809 | 206902809 | C | T | het | . | . | . | 248 | 0 | 0% | C | 273 | 8 | 2.85% | Y | Somatic | 0.0060456 |
| exonic | ZNF23 | synonymous SNV | NM_145911:c.T1014C:p.C338C | 439;Name=lod=81 |  |  |  |  |  |  |  |  |  |  | chr16 | 71482914 | 71482914 | A | G | het | . | . | . | 177 | 2 | 1.12% | A | 56 | 8 | 12.50% | R | Somatic | 4.61E-04 |
| exonic | PPP2R1B | synonymous SNV | NM_001177562:c.T45G:p.G15G |  |  |  |  |  |  |  |  |  |  |  | chr11 | 111637041 | 111637041 | A | C | het | . | . | . | 128 | 1 | 0.78% | A | 100 | 14 | 12.28% | M | Somatic | 1.42E-04 |
| splicing | SIPA1L2(NM_020808:exon13:c.3642+1G>A) |  |  | 492;Name=lod=133 |  |  |  |  |  |  |  |  |  |  | chr1 | 232577036 | 232577036 | C | T | het | . | . | . | 668 | 1 | 0.15% | C | 307 | 4 | 1.29% | Y | Somatic | 0.0374516 |
| exonic | PCNXL2 | synonymous SNV | NM_014801:c.G321A:p.E107E | 317;Name=lod=26 |  |  |  |  |  |  |  |  |  |  | chr1 | 233398742 | 233398742 | C | T | het | . | . | . | 270 | 0 | 0% | C | 115 | 4 | 3.36% | Y | Somatic | 0.0084527 |
| intronic | KRI1 |  |  |  |  |  |  |  | rs144204938 |  |  |  |  |  | chr19 | 10665944 | 10665944 | G | C | het | . | . | . | 76 | 0 | 0% | G | 50 | 7 | 12.28% | S | Somatic | 0.0021253 |
| exonic | ZNF345 | nonsynonymous SNV | NM_001242472:c.C701A:p.A234E |  |  |  |  |  |  | 1 | 0 | 0.119666 | 0.043005 | 0.633794 | chr19 | 37368433 | 37368433 | C | A | het | . | . | . | 183 | 3 | 1.61% | C | 65 | 9 | 12.16% | M | Somatic | 8.43E-04 |
| exonic | ZNF83 | nonsynonymous SNV | NM_018300:c.C823T:p.H275Y |  |  |  |  |  |  | 0.68 | 0 | 0.012646 | 0.004101 | 0.589247 | chr19 | 53116995 | 53116995 | G | A | het | . | . | . | 168 | 3 | 1.75% | G | 65 | 9 | 12.16% | R | Somatic | 0.0013691 |
| exonic | AHCTF1 | nonsynonymous SNV | NM_015446:c.G1135A:p.V379M | 669;Name=lod=696 |  |  |  |  |  | 0.35 | 0 | 0.248296 | 2.39E-04 | 0.999808 | chr1 | 247065836 | 247065836 | C | T | het | . | . | . | 390 | 0 | 0% | C | 217 | 4 | 1.81% | Y | Somatic | 0.0168199 |
| exonic | ITGA8 | stopgain SNV | NM_003638:c.T1572A:p.C524X | 329;Name=lod=29 |  |  |  |  |  | 0.02 | 0.73206 | 0.97872 | 1 | 1 | chr10 | 15650271 | 15650271 | A | T | het | . | . | . | 224 | 0 | 0% | A | 124 | 4 | 3.12% | W | Somatic | 0.0169649 |
| exonic | ZNF17 | nonsynonymous SNV | NM_006959:c.A1013T:p.Y338F |  |  |  |  |  |  | 0.71 | 0.001 | 0.070627 | 0.00208 | 0.60714 | chr19 | 57931873 | 57931873 | A | T | het | . | . | . | 213 | 3 | 1.39% | A | 65 | 9 | 12.16% | W | Somatic | 3.42E-04 |
| intronic | PRTFDC1 |  |  |  |  |  |  |  |  |  |  |  |  |  | chr10 | 25160899 | 25160899 | C | T | het | . | . | . | 297 | 0 | 0% | C | 377 | 6 | 1.57% | Y | Somatic | 0.0313801 |
| exonic | GPR158 | nonsynonymous SNV | NM_020752:c.C2870T:p.A957V |  |  |  |  |  |  | 0.66 | 0.002 | 0.191854 | 2.50E-05 | 0.573398 | chr10 | 25887425 | 25887425 | C | T | het | lung | carcinoma | adenocarcinoma | 518 | 1 | 0.19% | C | 269 | 5 | 1.82% | Y | Somatic | 0.0206401 |
| upstream | USP34 |  |  | 634;Name=lod=503 |  |  |  |  |  |  |  |  |  |  | chr2 | 61697858 | 61697858 | T | C | het | . | . | . | 75 | 0 | 0% | T | 66 | 9 | 12% | Y | Somatic | 0.0015142 |
| exonic | SYT15 | synonymous SNV | NM_031912:c.A132C:p.T44T |  |  |  |  |  |  |  |  |  |  |  | chr10 | 46969329 | 46969329 | T | G | het | . | . | . | 75 | 1 | 1.32% | T | 96 | 13 | 11.93% | K | Somatic | 0.0050843 |
| exonic | KIF5B | nonsynonymous SNV | NM_004521:c.G2237A:p.R746H | 648;Name=lod=573 |  |  |  |  |  | 0.02 | 0.33 | 0.998164 | 0.844249 | 1 | chr10 | 32308855 | 32308855 | C | T | het | . | . | . | 235 | 0 | 0% | C | 163 | 4 | 2.40% | Y | Somatic | 0.0291574 |
| exonic | PARD3 | nonsynonymous SNV | NM_001184790:c.G2819A:p.R940Q | 640;Name=lod=534 |  |  |  |  |  | 0.01 | 0.996 | 0.999135 | 0.997075 | 0.999998 | chr10 | 34573159 | 34573159 | C | T | het | . | . | . | 321 | 0 | 0% | C | 124 | 4 | 3.12% | Y | Somatic | 0.0063845 |
| exonic | ZNF586 | nonsynonymous SNV | NM_001077426:c.T413C:p.V138A | 366;Name=lod=41 |  |  |  |  |  | 0.11 |  |  |  |  | chr19 | 58290495 | 58290495 | T | C | het | . | . | . | 196 | 3 | 1.51% | T | 96 | 13 | 11.93% | Y | Somatic | 1.59E-04 |
| exonic | ZNF208 | nonsynonymous SNV | NM_007153:c.A946G:p.K316E |  | 0.92 |  |  |  | rs79937054 | 1 |  |  |  |  | chr19 | 22156890 | 22156890 | T | C | het | . | . | . | 166 | 2 | 1.19% | T | 52 | 7 | 11.86% | Y | Somatic | 0.0013767 |
| intronic | BICC1 |  |  |  |  |  |  |  |  |  |  |  |  |  | chr10 | 60566331 | 60566331 | G | T | het | . | . | . | 298 | 0 | 0% | G | 152 | 3 | 1.94% | K | Somatic | 0.0395486 |
| exonic | DNAJC12 | synonymous SNV | NM_021800:c.G483A:p.P161P |  |  |  |  |  |  |  |  |  |  |  | chr10 | 69565360 | 69565360 | C | T | het | kidney | carcinoma | clear_cell_renal_cell_carcinoma | 639 | 1 | 0.16% | C | 318 | 4 | 1.24% | Y | Somatic | 0.0455243 |
| exonic | KRI1 | nonsynonymous SNV | NM_023008:c.G1691A:p.C564Y | 583;Name=lod=311 |  |  |  |  |  | 0.01 | 0.998 | 0.998116 | 0.991164 | 1 | chr19 | 10665970 | 10665970 | C | T | het | . | . | . | 70 | 0 | 0% | C | 60 | 8 | 11.76% | Y | Somatic | 0.0027862 |
| exonic | RUFY2 | nonsynonymous SNV | NM_017987:c.C1826G:p.S609C | 649;Name=lod=578 |  |  |  |  |  | 0 | 0.987 | 0.999744 | 0.998634 | 0.999999 | chr10 | 70105625 | 70105625 | G | C | het | . | . | . | 280 | 0 | 0% | G | 187 | 4 | 2.09% | S | Somatic | 0.0265381 |
| exonic | KRI1 | nonsynonymous SNV | NM_023008:c.T1690C:p.C564R | 583;Name=lod=311 |  |  |  |  |  | 0.01 | 1 | 0.997842 | 0.98552 | 1 | chr19 | 10665971 | 10665971 | A | G | het | . | . | . | 70 | 0 | 0% | A | 60 | 8 | 11.76% | R | Somatic | 0.0027862 |
| exonic | TTC18 | synonymous SNV | NM_145170:c.C2766T:p.C922C | 629;Name=lod=479 |  |  |  |  |  | 0.12 |  |  |  |  | chr10 | 75035321 | 75035321 | G | A | het | . | . | . | 302 | 0 | 0% | G | 145 | 5 | 3.33% | R | Somatic | 0.0038473 |
| exonic | ZNF83 | nonsynonymous SNV | NM_018300:c.T818A:p.I273N |  |  |  |  |  |  | 0.54 | 0.080604 | 0.002349 | 0.023368 | 0.584283 | chr19 | 53117000 | 53117000 | A | T | het | . | . | . | 161 | 3 | 1.83% | A | 61 | 8 | 11.59% | W | Somatic | 0.0031625 |
| exonic | FAM190B | synonymous SNV | NM_018999:c.G1332A:p.G444G |  |  |  |  |  |  |  |  |  |  |  | chr10 | 86132140 | 86132140 | G | A | het | . | . | . | 409 | 2 | 0.49% | G | 204 | 5 | 2.39% | R | Somatic | 0.0465208 |
| exonic | LIPJ | synonymous SNV | NM_001010939:c.A585T:p.S195S | 243;Name=lod=13 |  | 0.02 | 0.02 | 0.02 | rs41299155 |  |  |  |  |  | chr10 | 90356555 | 90356555 | A | T | het | . | . | . | 436 | 0 | 0% | A | 170 | 4 | 2.30% | W | Somatic | 0.0064577 |
| exonic | PDE6B | synonymous SNV | NM_001145292:c.C414T:p.L138L | 429;Name=lod=74 |  |  |  |  |  |  |  |  |  |  | chr4 | 650806 | 650806 | C | T | het | . | . | . | 109 | 0 | 0% | C | 100 | 13 | 11.50% | Y | Somatic | 1.08E-04 |
| exonic | CACNB1 | nonsynonymous SNV | NM_000723:c.A1355C:p.D452A | 689;Name=lod=842 |  |  |  |  |  | 0.03 | 0.549411 | 0.998023 | 0.379433 | 0.989363 | chr17 | 37331888 | 37331888 | T | G | het | . | . | . | 117 | 2 | 1.68% | T | 54 | 7 | 11.48% | K | Somatic | 0.0076908 |
| exonic | LZTS2 | nonsynonymous SNV | NM_032429:c.C1279T:p.R427W | 522;Name=lod=177 |  |  |  |  |  | 0 | 0.997 | 0.972523 | 0.418202 | 0.999992 | chr10 | 102765425 | 102765425 | C | T | het | . | . | . | 63 | 0 | 0% | C | 53 | 4 | 7.02% | Y | Somatic | 0.0480865 |
| exonic | ADRA2A | synonymous SNV | NM_000681:c.G1263A:p.T421T | 674;Name=lod=729 |  |  |  |  |  |  |  |  |  |  | chr10 | 112839017 | 112839017 | G | A | het | . | . | . | 351 | 0 | 0% | G | 252 | 5 | 1.95% | R | Somatic | 0.013192 |
| exonic | PGAM1 | nonsynonymous SNV | NM_002629:c.T76G:p.Y26D | 630;Name=lod=484 |  |  |  |  |  | 0.04 | 0.972 | 0.997812 | 0.963661 | 0.998226 | chr10 | 99186140 | 99186140 | T | G | het | . | . | . | 78 | 1 | 1.27% | T | 85 | 11 | 11.46% | K | Somatic | 0.0064821 |
| exonic | DMBT1 | nonsynonymous SNV | NM_017579:c.C2132T:p.P711L |  |  |  |  |  |  | 0.01 |  |  |  |  | chr10 | 124351019 | 124351019 | C | T | het | . | . | . | 635 | 1 | 0.16% | C | 309 | 4 | 1.28% | Y | Somatic | 0.0431295 |
| intronic | TCERG1L |  |  |  |  |  |  |  |  |  |  |  |  |  | chr10 | 132944959 | 132944959 | G | A | het | . | . | . | 67 | 0 | 0% | G | 70 | 6 | 7.89% | R | Somatic | 0.0204717 |
| exonic | INPP5A | synonymous SNV | NM_005539:c.C84T:p.N28N | 450;Name=lod=90 |  |  |  |  |  |  |  |  |  |  | chr10 | 134421427 | 134421427 | C | T | het | . | . | . | 717 | 0 | 0% | C | 328 | 4 | 1.20% | Y | Somatic | 0.0099097 |
| exonic | OR51L1 | nonsynonymous SNV | NM_001004755:c.G466A:p.G156R | 346;Name=lod=34 |  |  |  |  |  | 0.29 | 0.988 | 0.999606 | 0.018263 | 0.992554 | chr11 | 5020678 | 5020678 | G | A | het | . | . | . | 283 | 0 | 0% | G | 146 | 4 | 2.67% | R | Somatic | 0.0140262 |
| exonic | OR51M1 | nonsynonymous SNV | NM_001004756:c.T306G:p.H102Q |  |  | 0.69 | 0.69 | 0.69 | rs1498467 | 0.11 |  |  |  |  | chr11 | 5410934 | 5410934 | T | G | het | . | . | . | 371 | 0 | 0% | T | 143 | 4 | 2.72% | K | Somatic | 0.0062969 |
| exonic | ABCC2 | synonymous SNV | NM_000392:c.C1992G:p.G664G | 336;Name=lod=31 |  |  |  |  |  |  |  |  |  |  | chr10 | 101572799 | 101572799 | C | G | het | . | . | . | 95 | 1 | 1.04% | C | 85 | 11 | 11.46% | S | Somatic | 0.0024642 |
| exonic | APBB1 | synonymous SNV | NM_001164:c.C252T:p.H84H | 663;Name=lod=659 |  |  |  |  |  |  |  |  |  |  | chr11 | 6432326 | 6432326 | G | A | het | . | . | . | 362 | 0 | 0% | G | 342 | 5 | 1.44% | R | Somatic | 0.0276684 |
| exonic | DENND5A | nonsynonymous SNV | NM_001243254:c.A1787T:p.D596V | 636;Name=lod=513 |  |  |  |  |  | 0.02 | 0.006 | 0.997667 | 0.97705 | 1 | chr11 | 9199798 | 9199798 | T | A | het | . | . | . | 267 | 0 | 0% | T | 116 | 2 | 1.69% | W | Somatic | 0.0933847 |
| exonic | DENND5A | stopgain SNV | NM_001243254:c.C238T:p.R80X | 690;Name=lod=846 |  |  |  |  |  | 0.23 | 0.73549 | 0.999576 | 1 | 0.999999 | chr11 | 9228273 | 9228273 | G | A | het | . | . | . | 461 | 0 | 0% | G | 177 | 3 | 1.67% | R | Somatic | 0.021878 |
| exonic | NCL | synonymous SNV | NM_005381:c.G720A:p.E240E | 295;Name=lod=21 |  |  |  |  |  |  |  |  |  |  | chr2 | 232325471 | 232325471 | C | T | het | . | . | . | 126 | 2 | 1.56% | C | 62 | 8 | 11.43% | Y | Somatic | 0.0042185 |
| exonic | USP47 | synonymous SNV | NM_017944:c.T3715C:p.L1239L | 638;Name=lod=523 |  | 0.12 | 0.12 | 0.12 | rs2307073 |  |  |  |  |  | chr11 | 11977573 | 11977573 | T | C | het | . | . | . | 304 | 0 | 0% | T | 150 | 6 | 3.85% | Y | Somatic | 0.0014261 |
| exonic | ZNF12 | stopgain SNV | NM_016265:c.T1071A:p.Y357X |  |  |  |  |  |  | 0 |  |  |  |  | chr7 | 6731502 | 6731502 | A | T | het | . | . | . | 201 | 2 | 0.99% | A | 70 | 9 | 11.39% | W | Somatic | 2.45E-04 |
| exonic | GTF2H1 | synonymous SNV | NM_005316:c.G57A:p.K19K | 501;Name=lod=145 |  |  |  |  |  |  |  |  |  |  | chr11 | 18354678 | 18354678 | G | A | het | . | . | . | 244 | 0 | 0% | G | 123 | 4 | 3.15% | R | Somatic | 0.0133063 |
| exonic | CCDC34 | nonsynonymous SNV | NM_030771:c.G470A:p.R157H | 559;Name=lod=249 |  |  |  |  |  | 0 | 0.999 | 0.998965 | 5.00E-06 | 1 | chr11 | 27378978 | 27378978 | C | T | het | . | . | . | 248 | 0 | 0% | C | 136 | 4 | 2.86% | Y | Somatic | 0.0164874 |
| ncRNA_exonic | RPSAP58 |  |  | 701;Name=lod=944 | 0.98 |  |  |  |  | 0 |  |  |  |  | chr19 | 24010193 | 24010193 | T | A | het | . | . | . | 87 | 0 | 0% | T | 86 | 11 | 11.34% | W | Somatic | 6.58E-04 |
| exonic | QSER1 | synonymous SNV | NM_001076786:c.G5193A:p.Q1731Q | 632;Name=lod=492 |  |  |  |  |  |  |  |  |  |  | chr11 | 32998005 | 32998005 | G | A | het | . | . | . | 301 | 1 | 0.33% | G | 133 | 4 | 2.92% | R | Somatic | 0.0347488 |
| intronic | MADD |  |  | 435;Name=lod=78 |  | 0.21 | 0.21 | 0.21 | rs58023804 |  |  |  |  |  | chr11 | 47312374 | 47312374 | A | G | het | . | . | . | 67 | 0 | 0% | A | 61 | 5 | 7.58% | R | Somatic | 0.0278061 |
| exonic | OR4C3 | synonymous SNV | NM_001004702:c.T873C:p.P291P |  |  |  |  |  |  |  |  |  |  |  | chr11 | 48347365 | 48347365 | T | C | het | . | . | . | 286 | 0 | 0% | T | 149 | 4 | 2.61% | Y | Somatic | 0.0143779 |
| intronic | TNKS1BP1 |  |  |  |  |  |  |  |  |  |  |  |  |  | chr11 | 57075849 | 57075849 | G | A | het | . | . | . | 570 | 0 | 0% | G | 509 | 4 | 0.78% | R | Somatic | 0.0500349 |
| exonic | OR5A1 | synonymous SNV | NM_001004728:c.C369T:p.Y123Y |  |  |  |  |  | rs139346783 |  |  |  |  |  | chr11 | 59211010 | 59211010 | C | T | het | . | . | . | 294 | 0 | 0% | C | 304 | 6 | 1.94% | Y | Somatic | 0.0178495 |
| exonic | AHNAK | synonymous SNV | NM_001620:c.T8103C:p.N2701N |  |  |  |  |  |  |  |  |  |  |  | chr11 | 62293786 | 62293786 | A | G | het | . | . | . | 512 | 1 | 0.19% | A | 208 | 8 | 3.70% | R | Somatic | 3.62E-04 |
| exonic | TIGD3 | nonsynonymous SNV | NM_145719:c.C535T:p.P179S | 417;Name=lod=66 |  |  |  |  |  | 0.45 | 0.251 | 0.996369 | 0.034826 | 0.997839 | chr11 | 65123814 | 65123814 | C | T | het | . | . | . | 573 | 0 | 0% | C | 432 | 4 | 0.92% | Y | Somatic | 0.0345919 |
| exonic | TNRC18 | nonsynonymous SNV | NM_001080495:c.C2104G:p.P702A | 391;Name=lod=52 |  |  |  |  |  | 0.25 |  |  |  |  | chr7 | 5427351 | 5427351 | G | C | het | . | . | . | 109 | 1 | 0.91% | G | 149 | 19 | 11.31% | S | Somatic | 4.13E-04 |
| exonic | ADRBK1 | synonymous SNV | NM_001619:c.C525T:p.C175C | 552;Name=lod=234 |  |  |  |  |  |  |  |  |  |  | chr11 | 67048224 | 67048224 | C | T | het | . | . | . | 475 | 0 | 0% | C | 427 | 4 | 0.93% | Y | Somatic | 0.050841 |
| exonic | DEFB108B | nonsynonymous SNV | NM_001002035:c.A79G:p.I27V |  | 0.97 | 0.45 | 0.45 | 0.45 | rs12285436 | 0 | 0.423 | 0.011694 | 7.51E-04 | 0.632167 | chr11 | 71548465 | 71548465 | A | G | het | . | . | . | 380 | 0 | 0% | A | 245 | 4 | 1.61% | R | Somatic | 0.0242009 |
| exonic | ARHGEF17 | synonymous SNV | NM_014786:c.C5277T:p.I1759I | 467;Name=lod=105 |  |  |  |  |  |  |  |  |  |  | chr11 | 73074822 | 73074822 | C | T | het | . | . | . | 157 | 0 | 0% | C | 285 | 12 | 4.04% | Y | Somatic | 0.0056794 |
| exonic | ZNF25 | synonymous SNV | NM_145011:c.C1000A:p.R334R | 493;Name=lod=135 |  |  |  |  |  |  |  |  |  |  | chr10 | 38241426 | 38241426 | G | T | het | . | . | . | 201 | 4 | 1.95% | G | 80 | 10 | 11.11% | K | Somatic | 0.0014944 |
| exonic | MRE11A | stopgain SNV | NM_005590:c.C1333T:p.Q445X | 478;Name=lod=117 |  |  |  |  |  | 0.03 | 0.735512 | 0.99971 | 1 | 1 | chr11 | 94192741 | 94192741 | G | A | het | . | . | . | 216 | 0 | 0% | G | 93 | 2 | 2.11% | R | Somatic | 0.0926252 |
| exonic | MMP13 | nonsynonymous SNV | NM_002427:c.G1384A:p.V462I | 393;Name=lod=53 |  |  |  |  |  | 0.33 | 0.001 | 0.985712 | 0.009265 | 0.842733 | chr11 | 102815027 | 102815027 | C | T | het | ovary | carcinoma | serous_carcinoma | 398 | 1 | 0.25% | C | 216 | 5 | 2.26% | Y | Somatic | 0.023714 |
| exonic | PLEKHG4 | nonsynonymous SNV | NM_001129731:c.A1763C:p.H588P |  |  |  |  |  |  | 0.13 | 0 | 0.079794 | 6.83E-04 | 0.582928 | chr16 | 67318929 | 67318929 | A | C | het | . | . | . | 69 | 1 | 1.43% | A | 64 | 8 | 11.11% | M | Somatic | 0.0184804 |
| intronic | PHLDB1 |  |  |  |  |  |  |  |  |  |  |  |  |  | chr11 | 118506068 | 118506068 | C | T | het | . | . | . | 310 | 0 | 0% | C | 301 | 5 | 1.63% | Y | Somatic | 0.0297515 |
| exonic | TECTA | synonymous SNV | NM_005422:c.C2256T:p.I752I | 492;Name=lod=133 |  | 0.31 | 0.31 | 0.31 | rs10502247 |  |  |  |  |  | chr11 | 120998942 | 120998942 | C | T | het | . | . | . | 305 | 0 | 0% | C | 268 | 5 | 1.83% | Y | Somatic | 0.0230521 |
| exonic | SORL1 | synonymous SNV | NM_003105:c.C4533T:p.T1511T |  |  |  |  |  |  |  |  |  |  |  | chr11 | 121474915 | 121474915 | C | T | het | . | . | . | 353 | 0 | 0% | C | 314 | 5 | 1.57% | Y | Somatic | 0.0237086 |
| exonic | UBASH3B | synonymous SNV | NM_032873:c.T1518A:p.V506V | 536;Name=lod=201 |  | 0.03 | 0.03 | 0.03 | rs3741015 |  |  |  |  |  | chr11 | 122671963 | 122671963 | T | A | het | . | . | . | 256 | 0 | 0% | T | 214 | 6 | 2.73% | W | Somatic | 0.0093923 |
| exonic | RAD51D | nonsynonymous SNV | NM_001142571:c.G135T:p.L45F | 560;Name=lod=251 |  |  |  |  |  | 0.01 | 0.780182 | 0.977251 | 0.997283 | 0.999804 | chr17 | 33446139 | 33446139 | C | A | het | . | . | . | 76 | 1 | 1.30% | C | 64 | 8 | 11.11% | M | Somatic | 0.0129117 |
| intronic | GLB1L2 |  |  |  |  | 0.31 | 0.31 | 0.31 | rs60460137 |  |  |  |  |  | chr11 | 134234186 | 134234186 | G | A | het | . | . | . | 394 | 0 | 0% | G | 306 | 4 | 1.29% | R | Somatic | 0.03719 |
| exonic | CHD4 | synonymous SNV | NM_001273:c.T3909A:p.I1303I | 808;Name=lod=2565 |  |  |  |  |  |  |  |  |  |  | chr12 | 6692515 | 6692515 | A | T | het | . | . | . | 232 | 0 | 0% | A | 172 | 6 | 3.37% | W | Somatic | 0.0063796 |
| exonic | A2ML1 | synonymous SNV | NM_144670:c.G2487A:p.S829S |  |  |  |  |  |  |  |  |  |  |  | chr12 | 9004829 | 9004829 | G | A | het | . | . | . | 325 | 1 | 0.31% | G | 222 | 5 | 2.20% | R | Somatic | 0.0451228 |
| exonic | ZNF17 | synonymous SNV | NM_006959:c.A993G:p.G331G |  |  |  |  |  |  |  |  |  |  |  | chr19 | 57931853 | 57931853 | A | G | het | . | . | . | 185 | 1 | 0.54% | A | 56 | 7 | 11.11% | R | Somatic | 3.26E-04 |
| intronic | PLEKHA5 |  |  |  |  | 0.75 | 0.75 | 0.75 | rs6486942 |  |  |  |  |  | chr12 | 19406863 | 19406863 | C | T | het | . | . | . | 230 | 1 | 0.43% | C | 129 | 6 | 4.44% | Y | Somatic | 0.0113716 |
| exonic | ZNF549 | synonymous SNV | NM_153263:c.T1050C:p.C350C | 251;Name=lod=14 |  |  |  |  |  |  |  |  |  |  | chr19 | 58049461 | 58049461 | T | C | het | . | . | . | 148 | 1 | 0.67% | T | 56 | 7 | 11.11% | Y | Somatic | 9.75E-04 |
| intronic | PLXNB2 |  |  |  |  |  |  |  |  |  |  |  |  |  | chr22 | 50721745 | 50721745 | T | G | het | . | . | . | 109 | 2 | 1.80% | T | 80 | 10 | 11.11% | K | Somatic | 0.0061299 |
| intergenic | OR11H1(dist=605654),CCT8L2(dist=16190) |  |  | 662;Name=lod=653 | 0.98 | 0.16 | 0.16 | 0.16 | rs35893428 |  |  |  |  |  | chr22 | 17055458 | 17055458 | G | A | het | . | . | . | 118 | 1 | 0.84% | G | 57 | 7 | 10.94% | R | Somatic | 0.0029331 |
| intronic | DUX4L3,DUX4L5,DUX4L6,DUX4L7 |  |  | 265;Name=lod=16 | 1 | 0.11 | 0.11 | 0.11 |  |  |  |  |  |  | chr10 | 135491150 | 135491150 | C | A | het | . | . | . | 142 | 2 | 1.39% | C | 57 | 7 | 10.94% | M | Somatic | 0.0041905 |
| exonic | ZNF23 | nonsynonymous SNV | NM_145911:c.G1031C:p.G344A | 439;Name=lod=81 |  |  |  |  |  | 1 | 0.683 | 0.918909 | 0.452971 | 0.786981 | chr16 | 71482897 | 71482897 | C | G | het | . | . | . | 165 | 2 | 1.20% | C | 57 | 7 | 10.94% | S | Somatic | 0.0021275 |
| exonic | LILRB4 | synonymous SNV | NM_001081438:c.C1125T:p.P375P |  |  | 0.009 | 0.009 | 0.009 | rs637515 |  |  |  |  |  | chr19 | 55179172 | 55179172 | C | T | het | . | . | . | 114 | 2 | 1.72% | C | 106 | 13 | 10.92% | Y | Somatic | 0.0033279 |
| exonic | PRKAG1 | synonymous SNV | NM_001206709:c.G786A:p.K262K | 691;Name=lod=859 |  |  |  |  |  |  |  |  |  |  | chr12 | 49397056 | 49397056 | C | T | het | . | . | . | 316 | 0 | 0% | C | 260 | 7 | 2.62% | Y | Somatic | 0.0040473 |
| exonic | DUX2 | nonsynonymous SNV | NM_012147:c.C1388T:p.S463L |  | 1 |  |  |  |  |  |  |  |  |  | chr4 | 190990294 | 190990294 | C | T | het | . | . | . | 87 | 0 | 0% | C | 90 | 11 | 10.89% | Y | Somatic | 8.24E-04 |
| exonic | KRT80 | synonymous SNV | NM_001081492:c.G765A:p.V255V | 506;Name=lod=152 |  |  |  |  |  |  |  |  |  |  | chr12 | 52567450 | 52567450 | C | T | het | . | . | . | 168 | 1 | 0.59% | C | 178 | 8 | 4.30% | Y | Somatic | 0.0258443 |
| exonic | CALCOCO1 | synonymous SNV | NM_001143682:c.G564A:p.R188R | 550;Name=lod=229 |  |  |  |  |  |  |  |  |  |  | chr12 | 54115346 | 54115346 | C | T | het | . | . | . | 430 | 1 | 0.23% | C | 471 | 7 | 1.46% | Y | Somatic | 0.047305 |
| intronic | PKP3 |  |  |  |  |  |  |  |  |  |  |  |  |  | chr11 | 403601 | 403601 | A | C | het | . | . | . | 88 | 1 | 1.12% | A | 74 | 9 | 10.84% | M | Somatic | 0.006624 |
| exonic | MDM1 | nonsynonymous SNV | NM_001205028:c.G1361A:p.R454H |  |  | 0.7 | 0.7 | 0.7 | rs2306393 | 0.16 | 0 | 0.247725 | 3.00E-06 | 0.090939 | chr12 | 68708761 | 68708761 | C | T | het | . | . | . | 669 | 1 | 0.15% | C | 308 | 9 | 2.84% | Y | Somatic | 2.41E-04 |
| exonic | ZNF485 | nonsynonymous SNV | NM_145312:c.T625A:p.S209T | 397;Name=lod=55 |  |  |  |  |  | 1 | 0.001 | 0.870291 | 0.002858 | 0.823448 | chr10 | 44112116 | 44112116 | T | A | het | . | . | . | 151 | 3 | 1.95% | T | 66 | 8 | 10.81% | W | Somatic | 0.0061357 |
| ncRNA_exonic | MIR618 |  |  |  |  | 0.76 | 0.76 | 0.76 | rs2682818 |  |  |  |  |  | chr12 | 81329536 | 81329536 | A | C | het | . | . | . | 107 | 0 | 0% | A | 46 | 6 | 11.54% | M | Somatic | 9.98E-04 |
| exonic | LRRIQ1 | synonymous SNV | NM_001079910:c.T894A:p.V298V |  |  | 0.59 | 0.59 | 0.59 | rs7974418 |  |  |  |  |  | chr12 | 85449465 | 85449465 | T | A | het | . | . | . | 215 | 0 | 0% | T | 98 | 4 | 3.92% | W | Somatic | 0.0102937 |
| exonic | ATP2B1 | synonymous SNV | NM_001001323:c.A306C:p.V102V | 747;Name=lod=1447 |  |  |  |  |  |  |  |  |  |  | chr12 | 90036035 | 90036035 | T | G | het | . | . | . | 588 | 1 | 0.17% | T | 282 | 4 | 1.40% | K | Somatic | 0.0416914 |
| exonic | C12orf12 | nonsynonymous SNV | NM_152638:c.G832A:p.E278K |  |  |  |  |  |  | 0 | 0.544421 | 0.900924 | 0.002435 | 0.967525 | chr12 | 91347688 | 91347688 | C | T | het | . | . | . | 904 | 0 | 0% | C | 417 | 2 | 0.48% | Y | Somatic | 0.100138 |
| exonic | C12orf12 | nonsynonymous SNV | NM_152638:c.G719A:p.S240N |  |  |  |  |  |  | 0.43 | 0.085239 | 0.149269 | 4.52E-04 | 0.271459 | chr12 | 91347801 | 91347801 | C | T | het | . | . | . | 876 | 0 | 0% | C | 690 | 4 | 0.58% | Y | Somatic | 0.0379961 |
| exonic | C12orf12 | synonymous SNV | NM_152638:c.T711C:p.P237P |  |  |  |  |  |  |  |  |  |  |  | chr12 | 91347809 | 91347809 | A | G | het | . | . | . | 845 | 1 | 0.12% | A | 657 | 6 | 0.90% | R | Somatic | 0.0310897 |
| exonic | UBE2N | synonymous SNV | NM_003348:c.T126C:p.P42P | 786;Name=lod=2085 | 0.95 |  |  |  |  |  |  |  |  |  | chr12 | 93804980 | 93804980 | A | G | het | . | . | . | 234 | 1 | 0.43% | A | 128 | 8 | 5.88% | R | Somatic | 0.0017625 |
| exonic | GATAD2A | nonsynonymous SNV | NM_017660:c.A1006C:p.T336P |  |  |  |  |  |  | 0.02 | 0.024 | 0.051401 | 0.001611 | 0.97832 | chr19 | 19609333 | 19609333 | A | C | het | . | . | . | 79 | 1 | 1.25% | A | 99 | 12 | 10.81% | M | Somatic | 0.0072558 |
| exonic | SCYL2 | nonsynonymous SNV | NM_017988:c.G2479A:p.A827T |  | 0.94 |  |  |  |  | 0.61 | 0 | 0.837456 | 0.001632 | 0.702788 | chr12 | 100732639 | 100732639 | G | A | het | . | . | . | 468 | 0 | 0% | G | 161 | 6 | 3.59% | R | Somatic | 3.09E-04 |
| exonic | MYBPC1 | nonsynonymous SNV | NM_206820:c.G1184A:p.G395D | 414;Name=lod=64 |  |  |  |  | rs149515650 | 0 | 0.997 | 0.999774 | 0.996427 | 0.999959 | chr12 | 102043100 | 102043100 | G | A | het | . | . | . | 219 | 0 | 0% | G | 117 | 5 | 4.10% | R | Somatic | 0.0055562 |
| intronic | USP30 |  |  |  |  |  |  |  |  |  |  |  |  |  | chr12 | 109522721 | 109522721 | G | A | het | . | . | . | 426 | 0 | 0% | G | 295 | 7 | 2.32% | R | Somatic | 0.002029 |
| exonic | ERP29 | nonsynonymous SNV | NM_006817:c.G699A:p.M233I | 513;Name=lod=162 |  |  |  |  |  | 0.53 | 0.998 | 0.999657 | 0.954195 | 1 | chr12 | 112460369 | 112460369 | G | A | het | cervix | carcinoma | squamous_cell_carcinoma | 207 | 0 | 0% | G | 208 | 6 | 2.80% | R | Somatic | 0.0166581 |
| exonic | C12orf51 | nonsynonymous SNV | NM_001109662:c.G1517A:p.G506D | 671;Name=lod=708 |  |  |  |  |  | 0 |  |  |  |  | chr12 | 112708143 | 112708143 | C | T | het | . | . | . | 342 | 0 | 0% | C | 176 | 4 | 2.22% | Y | Somatic | 0.0138306 |
| exonic | ZNF416 | synonymous SNV | NM_017879:c.C978T:p.Y326Y |  |  |  |  |  | rs138114602 |  |  |  |  |  | chr19 | 58084294 | 58084294 | G | A | het | . | . | . | 189 | 3 | 1.56% | G | 91 | 11 | 10.78% | R | Somatic | 7.73E-04 |
| exonic | REN | nonsynonymous SNV | NM_000537:c.A436C:p.T146P | 391;Name=lod=52 |  |  |  |  |  | 0.04 | 0.141 | 0.194971 | 0.77806 | 0.999779 | chr1 | 204129744 | 204129744 | T | G | het | . | . | . | 82 | 0 | 0% | T | 116 | 14 | 10.77% | K | Somatic | 7.98E-04 |
| exonic | PABPC3 | nonsynonymous SNV | NM_030979:c.G907T:p.D303Y | 857;Name=lod=4067 | 0.91 |  |  |  |  | 0 | 0.996 | 0.967117 | 0.999599 | 0.999905 | chr13 | 25671243 | 25671243 | G | T | het | lung | carcinoma | adenocarcinoma | 669 | 1 | 0.15% | G | 272 | 4 | 1.45% | K | Somatic | 0.0274313 |
| exonic | ZNF571 | synonymous SNV | NM_016536:c.A840G:p.E280E |  |  |  |  |  |  |  |  |  |  |  | chr19 | 38056490 | 38056490 | T | C | het | . | . | . | 165 | 0 | 0% | T | 58 | 7 | 10.77% | Y | Somatic | 1.13E-04 |
| exonic | EFS | synonymous SNV | NM_032459:c.A738C:p.P246P | 489;Name=lod=129 |  |  |  |  |  |  |  |  |  |  | chr14 | 23828670 | 23828670 | T | G | het | . | . | . | 103 | 2 | 1.90% | T | 83 | 10 | 10.75% | K | Somatic | 0.0094404 |
| exonic | NBEA | nonsynonymous SNV | NM_015678:c.C211T:p.R71W | 714;Name=lod=1064 |  |  |  |  |  | 0 |  |  |  |  | chr13 | 35517168 | 35517168 | C | T | het | . | . | . | 266 | 0 | 0% | C | 134 | 5 | 3.60% | Y | Somatic | 0.0045392 |
| exonic | ZC3H13 | nonsynonymous SNV | NM_015070:c.G2606A:p.R869Q | 556;Name=lod=243 |  |  |  |  |  | 0.03 | 0.7774 | 0.999392 | 0.997756 | 0.999957 | chr13 | 46544073 | 46544073 | C | T | het | . | . | . | 257 | 0 | 0% | C | 91 | 4 | 4.21% | Y | Somatic | 0.0050627 |
| exonic | CITED2 | nonsynonymous SNV | NM_001168388:c.A523C:p.T175P | 755;Name=lod=1561 |  |  |  |  |  | 0.21 | 0.508 | 0.95615 | 7.17E-04 | 0.851893 | chr6 | 139694559 | 139694559 | T | G | het | . | . | . | 83 | 0 | 0% | T | 50 | 6 | 10.71% | K | Somatic | 0.0036158 |
| intronic | KLF5 |  |  |  |  |  |  |  |  |  |  |  |  |  | chr13 | 73649808 | 73649808 | C | T | het | . | . | . | 538 | 0 | 0% | C | 335 | 4 | 1.18% | Y | Somatic | 0.0220832 |
| exonic | MYCBP2 | nonsynonymous SNV | NM_015057:c.G10481A:p.R3494Q | 676;Name=lod=745 |  |  |  |  |  | 0.1 | 0.994 | 0.998382 | 0.994677 | 0.999999 | chr13 | 77664285 | 77664285 | C | T | het | . | . | . | 445 | 2 | 0.45% | C | 128 | 6 | 4.48% | Y | Somatic | 0.0025301 |
| exonic | MYCBP2 | nonsynonymous SNV | NM_015057:c.G4465A:p.E1489K | 697;Name=lod=909 |  |  |  |  |  | 0.14 | 0.028 | 0.998933 | 0.96613 | 0.999948 | chr13 | 77759492 | 77759492 | C | T | het | . | . | . | 761 | 1 | 0.13% | C | 241 | 4 | 1.63% | Y | Somatic | 0.0138903 |
| exonic | ADAM12 | synonymous SNV | NM_003474:c.A2484C:p.A828A |  |  |  |  |  |  |  |  |  |  |  | chr10 | 127724769 | 127724769 | T | G | het | . | . | . | 68 | 1 | 1.45% | T | 126 | 15 | 10.64% | K | Somatic | 0.012362 |
| exonic | MUC17 | nonsynonymous SNV | NM_001040105:c.T11365A:p.S3789T |  |  |  |  |  |  |  | 0.651 | 0.095933 | 2.00E-06 | 0.623084 | chr7 | 100686062 | 100686062 | T | A | het | . | . | . | 144 | 2 | 1.37% | T | 59 | 7 | 10.61% | W | Somatic | 0.0045441 |
| exonic | GPR18 | nonsynonymous SNV | NM_001098200:c.C931T:p.R311C | 484;Name=lod=124 |  |  |  |  |  | 0.01 | 0.999 | 0.999772 | 0.988831 | 1 | chr13 | 99907196 | 99907196 | G | A | het | . | . | . | 308 | 0 | 0% | G | 133 | 5 | 3.62% | R | Somatic | 0.0026957 |
| exonic | NALCN | nonsynonymous SNV | NM_052867:c.G4072T:p.G1358C | 419;Name=lod=67 |  |  |  |  |  | 0 | 0.999 | 0.99897 | 0.999973 | 1 | chr13 | 101726896 | 101726896 | C | A | het | lung | carcinoma | adenocarcinoma | 332 | 0 | 0% | C | 146 | 5 | 3.31% | M | Somatic | 0.0028518 |
| exonic | ADAMTSL3 | nonsynonymous SNV | NM_207517:c.A3497C:p.N1166T |  |  |  |  |  |  | 0.6 | 0.01 | 0.029172 | 0.0438 | 0.155849 | chr15 | 84651877 | 84651877 | A | C | het | ovary | carcinoma | serous_carcinoma | 134 | 1 | 0.74% | A | 118 | 14 | 10.61% | M | Somatic | 3.06E-04 |
| exonic | MCF2L | nonsynonymous SNV | NM_001112732:c.C892A:p.Q298K | 492;Name=lod=133 |  |  |  |  |  | 0 | 0.051 | 0.998852 | 0.03949 | 1 | chr13 | 113724383 | 113724383 | C | A | het | . | . | . | 355 | 0 | 0% | C | 314 | 4 | 1.26% | M | Somatic | 0.0493517 |
| exonic;splicing | TTC5;TTC5 | synonymous SNV | NM_138376:c.G696A:p.T232T | 584;Name=lod=316 |  |  |  |  |  |  |  |  |  |  | chr14 | 20764549 | 20764549 | C | T | het | kidney | carcinoma | clear_cell_renal_cell_carcinoma | 354 | 0 | 0% | C | 169 | 5 | 2.87% | Y | Somatic | 0.0037381 |
| exonic | ZNF17 | nonsynonymous SNV | NM_006959:c.A998G:p.N333S |  |  |  |  |  |  | 0.86 | 0.98 | 0.13212 | 0.001903 | 0.67353 | chr19 | 57931858 | 57931858 | A | G | het | . | . | . | 196 | 2 | 1.01% | A | 59 | 7 | 10.61% | R | Somatic | 0.0010941 |
| exonic | DHRS4L2 | nonsynonymous SNV | NM_001193636:c.G28A:p.D10N | 461;Name=lod=100 | 0.97 |  |  |  | rs146629945 | 0.04 | 0.734467 | 0.996704 | 0.998143 | 1 | chr14 | 24464265 | 24464265 | G | A | het | . | . | . | 871 | 1 | 0.11% | G | 582 | 7 | 1.19% | R | Somatic | 0.0088066 |
| exonic | RIPK3 | nonsynonymous SNV | NM_006871:c.G98A:p.G33D |  |  |  |  |  |  | 0 | 0.999 | 0.998578 | 0.989647 | 0.99696 | chr14 | 24808726 | 24808726 | C | T | het | . | . | . | 278 | 1 | 0.36% | C | 246 | 7 | 2.77% | Y | Somatic | 0.024846 |
| exonic | STXBP6 | synonymous SNV | NM_014178:c.C273T:p.I91I |  |  |  |  |  |  |  |  |  |  |  | chr14 | 25326245 | 25326245 | G | A | het | . | . | . | 311 | 0 | 0% | G | 110 | 5 | 4.35% | R | Somatic | 0.001344 |
| exonic | SLC35G6 | nonsynonymous SNV | NM_001102614:c.A26C:p.N9T | 609;Name=lod=397 | 0.97 |  |  |  |  | 0 |  |  |  |  | chr17 | 7385329 | 7385329 | A | C | het | . | . | . | 70 | 1 | 1.41% | A | 76 | 9 | 10.59% | M | Somatic | 0.0184695 |
| exonic | PSMA6 | nonsynonymous SNV | NM_002791:c.C503T:p.A168V | 669;Name=lod=696 |  |  |  |  |  | 0.62 | 0.002 | 0.999034 | 0.999554 | 1 | chr14 | 35782180 | 35782180 | C | T | het | . | . | . | 353 | 0 | 0% | C | 192 | 4 | 2.04% | Y | Somatic | 0.0159264 |
| exonic | C1orf56 | synonymous SNV | NM_017860:c.A531C:p.T177T |  |  |  |  |  |  |  |  |  |  |  | chr1 | 151020854 | 151020854 | A | C | het | . | . | . | 116 | 2 | 1.69% | A | 68 | 8 | 10.53% | M | Somatic | 0.0089995 |
| exonic | SAMD4A | synonymous SNV | NM_015589:c.C843T:p.P281P | 786;Name=lod=2087 |  |  |  |  |  |  |  |  |  |  | chr14 | 55203869 | 55203869 | C | T | het | . | . | . | 439 | 0 | 0% | C | 285 | 8 | 2.73% | Y | Somatic | 6.22E-04 |
| intronic | SYNE2 |  |  |  |  |  |  |  |  | 0 |  |  |  |  | chr14 | 64427499 | 64427499 | C | T | het | . | . | . | 37 | 0 | 0% | C | 27 | 4 | 12.90% | Y | Somatic | 0.0386365 |
| exonic | MPP5 | nonsynonymous SNV | NM_022474:c.C503T:p.A168V | 706;Name=lod=991 |  |  |  |  |  | 0.45 | 0.004 | 0.998792 | 0.999977 | 1 | chr14 | 67759363 | 67759363 | C | T | het | . | . | . | 300 | 0 | 0% | C | 110 | 4 | 3.51% | Y | Somatic | 0.0055314 |
| intronic | ZFYVE26 |  |  |  |  | 0.08 | 0.08 | 0.08 | rs2295110 |  |  |  |  |  | chr14 | 68251013 | 68251013 | G | A | het | . | . | . | 284 | 0 | 0% | G | 124 | 5 | 3.88% | R | Somatic | 0.0028164 |
| exonic | SMOC1 | synonymous SNV | NM_001034852:c.G126A:p.Q42Q | 528;Name=lod=187 |  | 0.21 | 0.21 | 0.21 | rs3742909 |  |  |  |  |  | chr14 | 70418881 | 70418881 | G | A | het | . | . | . | 168 | 0 | 0% | G | 173 | 8 | 4.42% | R | Somatic | 0.0048505 |
| intergenic | SPTSSB(dist=57239),OTOL1(dist=67486) |  |  | 783;Name=lod=2025 |  |  |  |  | rs61733116 |  |  |  |  |  | chr3 | 161147110 | 161147110 | G | A | het | . | . | . | 186 | 3 | 1.59% | G | 153 | 18 | 10.53% | R | Somatic | 2.35E-04 |
| exonic | FAM161B | nonsynonymous SNV | NM_152445:c.A1649G:p.K550R | 444;Name=lod=85 |  | 0.4 | 0.4 | 0.4 | rs28927675 | 0.22 | 0.246 | 0.998419 | 0.587826 | 0.992014 | chr14 | 74404756 | 74404756 | T | C | het | . | . | . | 267 | 0 | 0% | T | 164 | 5 | 2.96% | Y | Somatic | 0.0084347 |
| exonic | YLPM1 | stopgain SNV | NM_019589:c.C6424T:p.R2142X | 728;Name=lod=1212 |  |  |  |  |  | 1 |  |  |  |  | chr14 | 75302097 | 75302097 | C | T | het | . | . | . | 241 | 0 | 0% | C | 143 | 4 | 2.72% | Y | Somatic | 0.0200821 |
| intronic | GPR123 |  |  |  |  |  |  |  |  |  |  |  |  |  | chr10 | 134912122 | 134912122 | A | C | het | . | . | . | 109 | 1 | 0.91% | A | 68 | 8 | 10.53% | M | Somatic | 0.0036745 |
| exonic | OR2T27 | synonymous SNV | NM_001001824:c.A453G:p.G151G |  | 0.98 |  |  |  |  |  |  |  |  |  | chr1 | 248813733 | 248813733 | T | C | het | . | . | . | 94 | 1 | 1.05% | T | 154 | 18 | 10.47% | Y | Somatic | 0.0020535 |
| exonic | C14orf49 | synonymous SNV | NM_152592:c.C2307T:p.F769F | 412;Name=lod=63 |  |  |  |  |  |  |  |  |  |  | chr14 | 95905439 | 95905439 | G | A | het | . | . | . | 408 | 0 | 0% | G | 277 | 4 | 1.42% | R | Somatic | 0.0273165 |
| exonic | ZNF490 | nonsynonymous SNV | NM_020714:c.T1004A:p.V335E |  |  |  |  |  |  | 1 | 0 | 9.25E-04 | 0.002496 | 0.595462 | chr19 | 12691885 | 12691885 | A | T | het | . | . | . | 211 | 4 | 1.86% | A | 60 | 7 | 10.45% | W | Somatic | 0.0047161 |
| exonic | AHNAK2 | nonsynonymous SNV | NM_138420:c.G11377A:p.D3793N |  | 0.91 | 0.5 | 0.5 | 0.5 | rs11160825 | 0.08 |  |  |  |  | chr14 | 105410411 | 105410411 | C | T | het | . | . | . | 666 | 0 | 0% | C | 528 | 5 | 0.94% | Y | Somatic | 0.0171789 |
| exonic | AHNAK2 | nonsynonymous SNV | NM_138420:c.C7910T:p.A2637V |  | 0.91 |  |  |  |  | 0.06 |  |  |  |  | chr14 | 105413878 | 105413878 | G | A | het | . | . | . | 630 | 0 | 0% | G | 610 | 5 | 0.81% | R | Somatic | 0.0291704 |
| exonic | AHNAK2 | synonymous SNV | NM_138420:c.G6978C:p.L2326L |  | 0.91 | 0.53 | 0.53 | 0.53 | rs10145032 |  |  |  |  |  | chr14 | 105414810 | 105414810 | C | G | het | . | . | . | 807 | 1 | 0.12% | C | 618 | 8 | 1.28% | S | Somatic | 0.0071146 |
| exonic | AHNAK2 | nonsynonymous SNV | NM_138420:c.C6481T:p.P2161S | 446;Name=lod=87 | 0.91 |  |  |  |  | 0.16 |  |  |  |  | chr14 | 105415307 | 105415307 | G | A | het | . | . | . | 387 | 0 | 0% | G | 326 | 4 | 1.21% | R | Somatic | 0.0444319 |
| intergenic | ADAM6(dist=183684),LINC00226(dist=122227) |  |  |  | 0.96 |  |  |  |  | 0 |  |  |  |  | chr14 | 106622042 | 106622042 | G | A | het | . | . | . | 421 | 0 | 0% | G | 415 | 5 | 1.19% | R | Somatic | 0.0306946 |
| exonic | ZFP106 | nonsynonymous SNV | NM_022473:c.G2152C:p.E718Q | 596;Name=lod=354 |  |  |  |  |  | 0.11 | 0.514 | 0.999111 | 0.004619 | 0.997328 | chr15 | 42742249 | 42742249 | C | G | het | . | . | . | 224 | 0 | 0% | C | 103 | 5 | 4.63% | S | Somatic | 0.0034181 |
| exonic | TMEM62 | nonsynonymous SNV | NM_024956:c.G1517A:p.G506D | 519;Name=lod=172 |  |  |  |  |  | 0.24 | 0.314 | 0.990516 | 0.998939 | 1 | chr15 | 43473409 | 43473409 | G | A | het | . | . | . | 385 | 0 | 0% | G | 225 | 5 | 2.17% | R | Somatic | 0.0071176 |
| exonic | ZNF345 | synonymous SNV | NM_001242472:c.C720T:p.S240S |  |  |  |  |  |  |  |  |  |  |  | chr19 | 37368452 | 37368452 | C | T | het | . | . | . | 180 | 1 | 0.55% | C | 69 | 8 | 10.39% | Y | Somatic | 3.28E-04 |
| exonic | AP4E1 | synonymous SNV | NM_007347:c.A2685G:p.S895S |  |  |  |  |  |  |  |  |  |  |  | chr15 | 51289861 | 51289861 | A | G | het | . | . | . | 223 | 0 | 0% | A | 139 | 4 | 2.80% | R | Somatic | 0.0227086 |
| exonic | MYO5A | stopgain SNV | NM_000259:c.C697T:p.R233X | 704;Name=lod=969 |  |  |  |  |  | 0.71 | 0.733622 | 0.988225 | 1 | 0.999992 | chr15 | 52702589 | 52702589 | G | A | het | large_intestine | carcinoma | adenocarcinoma | 251 | 0 | 0% | G | 142 | 4 | 2.74% | R | Somatic | 0.0178172 |
| exonic | NEDD4 | nonsynonymous SNV | NM_198400:c.G1820A:p.R607Q | 313;Name=lod=25 |  | 0.68 | 0.68 | 0.68 | rs2303580 | 0.34 | 0.550354 | 0.837456 | 0.978629 | 0.052875 | chr15 | 56152872 | 56152872 | C | T | het | . | . | . | 271 | 0 | 0% | C | 106 | 4 | 3.64% | Y | Somatic | 0.0066802 |
| exonic | VPS13C | stopgain SNV | NM_017684:c.G4915T:p.E1639X | 415;Name=lod=65 |  |  |  |  |  | 0.18 | 0.735306 | 0.998469 | 1 | 0.999987 | chr15 | 62238018 | 62238018 | C | A | het | . | . | . | 554 | 0 | 0% | C | 274 | 4 | 1.44% | M | Somatic | 0.012286 |
| exonic | ZNF699 | synonymous SNV | NM_198535:c.G1380C:p.S460S |  |  |  |  |  |  |  |  |  |  |  | chr19 | 9406700 | 9406700 | C | G | het | . | . | . | 139 | 1 | 0.71% | C | 52 | 6 | 10.34% | S | Somatic | 0.0028062 |
| exonic | ZNF609 | synonymous SNV | NM_015042:c.T99C:p.N33N | 840;Name=lod=3457 |  |  |  |  |  |  |  |  |  |  | chr15 | 64791717 | 64791717 | T | C | het | . | . | . | 210 | 0 | 0% | T | 194 | 5 | 2.51% | Y | Somatic | 0.0265654 |
| exonic | ZNF609 | synonymous SNV | NM_015042:c.G225C:p.V75V | 840;Name=lod=3457 |  |  |  |  |  |  |  |  |  |  | chr15 | 64791843 | 64791843 | G | C | het | . | . | . | 207 | 0 | 0% | G | 242 | 7 | 2.81% | S | Somatic | 0.0139248 |
| exonic | ZNF606 | nonsynonymous SNV | NM_025027:c.A1255C:p.K419Q | 488;Name=lod=128 |  |  |  |  |  | 0.96 | 0.683 | 0.898616 | 0.048716 | 0.997634 | chr19 | 58490793 | 58490793 | T | G | het | . | . | . | 155 | 3 | 1.90% | T | 61 | 7 | 10.29% | K | Somatic | 0.0092626 |
| exonic | TOX2 | synonymous SNV | NM_001098798:c.A1125C:p.P375P | 480;Name=lod=119 |  |  |  |  |  |  |  |  |  |  | chr20 | 42694570 | 42694570 | A | C | het | . | . | . | 102 | 1 | 0.97% | A | 61 | 7 | 10.29% | M | Somatic | 0.006984 |
| exonic | ACAN | synonymous SNV | NM_001135:c.G4350A:p.G1450G |  |  |  |  |  |  |  |  |  |  |  | chr15 | 89400166 | 89400166 | G | A | het | . | . | . | 423 | 1 | 0.24% | G | 203 | 5 | 2.40% | R | Somatic | 0.0163749 |
| intronic | WDR90 |  |  |  |  |  |  |  |  |  |  |  |  |  | chr16 | 700228 | 700228 | C | T | het | . | . | . | 965 | 0 | 0% | C | 620 | 4 | 0.64% | Y | Somatic | 0.0236429 |
| intronic | TBL3 |  |  |  |  |  |  |  |  |  |  |  |  |  | chr16 | 2024356 | 2024356 | G | A | het | . | . | . | 384 | 1 | 0.26% | G | 340 | 8 | 2.30% | R | Somatic | 0.0129798 |
| exonic | SRRM2 | synonymous SNV | NM_016333:c.G3633A:p.R1211R | 295;Name=lod=21 |  | 0.61 | 0.61 | 0.61 | rs3094775 |  |  |  |  |  | chr16 | 2814162 | 2814162 | G | A | het | . | . | . | 431 | 0 | 0% | G | 221 | 4 | 1.78% | R | Somatic | 0.0135973 |
| exonic | SRRM2 | nonsynonymous SNV | NM_016333:c.C6275T:p.T2092I | 492;Name=lod=133 |  |  |  |  |  |  | 0.022 | 0.979957 | 0.864006 | 0.999925 | chr16 | 2816804 | 2816804 | C | T | het | . | . | . | 359 | 0 | 0% | C | 240 | 4 | 1.64% | Y | Somatic | 0.0264173 |
| exonic | ACSM2B | nonsynonymous SNV | NM_001105069:c.C1651T:p.P551S | 381;Name=lod=47 | 0.98 |  |  |  |  | 0 | 0.998 | 0.994605 | 0.150026 | 0.999729 | chr16 | 20548663 | 20548663 | G | A | het | . | . | . | 463 | 1 | 0.22% | G | 212 | 4 | 1.85% | R | Somatic | 0.0374115 |
| exonic | ACSM3 | stopgain SNV | NM_005622:c.C1459T:p.R487X | 407;Name=lod=60 |  |  |  |  |  | 1 | 0.704866 | 0.813375 | 1 | 1 | chr16 | 20803562 | 20803562 | C | T | het | . | . | . | 618 | 1 | 0.16% | C | 229 | 4 | 1.72% | Y | Somatic | 0.0215156 |
| exonic | TAOK2 | nonsynonymous SNV | NM_004783:c.C1400T:p.A467V | 623;Name=lod=454 |  |  |  |  |  | 0 | 0.891 | 0.979395 | 0.999182 | 1 | chr16 | 29994963 | 29994963 | C | T | het | . | . | . | 580 | 0 | 0% | C | 875 | 7 | 0.79% | Y | Somatic | 0.0288091 |
| exonic | ZNF12 | nonsynonymous SNV | NM_016265:c.T1069G:p.Y357D |  |  |  |  |  |  | 0.18 |  |  |  |  | chr7 | 6731504 | 6731504 | A | C | het | . | . | . | 206 | 4 | 1.90% | A | 70 | 8 | 10.26% | M | Somatic | 0.0039952 |
| exonic | ACIN1 | synonymous SNV | NM_001164816:c.A1185C:p.P395P | 464;Name=lod=103 |  |  |  |  | rs139086496 |  |  |  |  |  | chr14 | 23530739 | 23530739 | T | G | het | . | . | . | 70 | 0 | 0% | T | 70 | 8 | 10.26% | K | Somatic | 0.0049785 |
| intronic | AMFR |  |  |  |  | 0.04 | 0.04 | 0.04 | rs77132693 |  |  |  |  |  | chr16 | 56401352 | 56401352 | T | C | het | breast | carcinoma | ductal_carcinoma | 513 | 1 | 0.19% | T | 349 | 6 | 1.69% | Y | Somatic | 0.0207288 |
| exonic | MT1E | nonsynonymous SNV | NM_175617:c.G22A:p.A8T |  |  |  |  |  |  | 0.22 | 0.003 | 0.020416 | 0.190503 | 0.992759 | chr16 | 56659785 | 56659785 | G | A | het | . | . | . | 296 | 0 | 0% | G | 251 | 5 | 1.95% | R | Somatic | 0.0210053 |
| exonic | CKLF-CMTM1,CMTM1 | synonymous SNV | NM_001202509:c.C213T:p.I71I |  |  |  |  |  |  |  |  |  |  |  | chr16 | 66612769 | 66612769 | C | T | het | . | . | . | 324 | 1 | 0.31% | C | 166 | 4 | 2.35% | Y | Somatic | 0.0495714 |
| UTR3 | EIF2AK1 |  |  |  |  |  |  |  |  |  |  |  |  |  | chr7 | 6062920 | 6062920 | T | G | het | . | . | . | 71 | 1 | 1.39% | T | 79 | 9 | 10.23% | K | Somatic | 0.0200747 |
| intronic | WDR59 |  |  |  |  | 0.62 | 0.62 | 0.62 | rs4887796 |  |  |  |  |  | chr16 | 74999625 | 74999625 | A | T | het | . | . | . | 141 | 0 | 0% | A | 184 | 10 | 5.15% | W | Somatic | 0.0038374 |
| exonic | MLYCD | synonymous SNV | NM_012213:c.C1029T:p.N343N |  |  |  |  |  |  |  |  |  |  |  | chr16 | 83948641 | 83948641 | C | T | het | . | . | . | 571 | 0 | 0% | C | 272 | 6 | 2.16% | Y | Somatic | 0.0011883 |
| exonic | BSN | nonsynonymous SNV | NM_003458:c.T10597C:p.S3533P | 596;Name=lod=354 |  |  |  |  |  | 0.01 | 0.734483 | 0.998081 | 0.99743 | 1 | chr3 | 49699875 | 49699875 | T | C | het | . | . | . | 110 | 1 | 0.90% | T | 88 | 10 | 10.20% | Y | Somatic | 0.0026161 |
| exonic | FLCN | nonsynonymous SNV | NM_144606:c.T884G:p.V295G |  |  |  |  |  |  | 0.27 | 0.321093 | 0.852779 | 1.10E-05 | 0.819012 | chr17 | 17124838 | 17124838 | A | C | het | . | . | . | 69 | 1 | 1.43% | A | 150 | 17 | 10.18% | M | Somatic | 0.0129279 |
| exonic | ANKRD11 | nonsynonymous SNV | NM_013275:c.G2752A:p.E918K | 399;Name=lod=56 |  |  |  |  |  | 0 | 0.937 | 0.998548 | 0 | 0.999815 | chr16 | 89350198 | 89350198 | C | T | het | . | . | . | 344 | 0 | 0% | C | 193 | 7 | 3.50% | Y | Somatic | 8.49E-04 |
| splicing | ANKFY1(NM_016376:exon10:c.1172+1G>A,NM_020740:exon10:c.1172+1G>A) |  |  | 529;Name=lod=189 |  |  |  |  |  |  |  |  |  |  | chr17 | 4098697 | 4098697 | C | T | het | . | . | . | 419 | 0 | 0% | C | 439 | 5 | 1.13% | Y | Somatic | 0.0356523 |
| exonic | KDM6B | synonymous SNV | NM_001080424:c.G2124A:p.K708K | 598;Name=lod=359 |  |  |  |  |  |  |  |  |  |  | chr17 | 7751730 | 7751730 | G | A | het | . | . | . | 335 | 0 | 0% | G | 316 | 5 | 1.56% | R | Somatic | 0.0276089 |
| exonic | PRPSAP2 | nonsynonymous SNV | NM_001243936:c.C322T:p.H108Y | 613;Name=lod=415 |  |  |  |  |  | 0.04 | 0.316 | 0.998193 | 0.976598 | 0.999998 | chr17 | 18785910 | 18785910 | C | T | het | . | . | . | 277 | 0 | 0% | C | 264 | 5 | 1.86% | Y | Somatic | 0.0284799 |
| exonic | PHF12 | nonsynonymous SNV | NM_001033561:c.C2588T:p.T863M | 720;Name=lod=1125 |  |  |  |  |  | 0.06 | 0.999 | 0.991311 | 0.998037 | 1 | chr17 | 27233966 | 27233966 | G | A | het | . | . | . | 228 | 0 | 0% | G | 172 | 5 | 2.82% | R | Somatic | 0.0154386 |
| exonic | ZNF121 | nonsynonymous SNV | NM_001008727:c.A607G:p.K203E |  |  |  |  |  |  | 0.01 | 0.093 | 0.941525 | 0.038719 | 0.831003 | chr19 | 9677182 | 9677182 | T | C | het | . | . | . | 199 | 2 | 1% | T | 53 | 6 | 10.17% | Y | Somatic | 0.0021019 |
| exonic | NF1 | nonsynonymous SNV | NM_000267:c.G3749A:p.R1250Q | 706;Name=lod=985 | 0.92 |  |  |  |  | 0.01 | 0.955 | 0.99966 | 0.999629 | 0.999998 | chr17 | 29562669 | 29562669 | G | A | het | . | . | . | 503 | 0 | 0% | G | 219 | 5 | 2.23% | R | Somatic | 0.0026918 |
| exonic | C17orf102 | nonsynonymous SNV | NM_207454:c.T350A:p.L117Q |  |  |  |  |  |  |  | 0.107167 | 0.031259 | 0.011856 | 3.35E-04 | chr17 | 32905950 | 32905950 | A | T | het | . | . | . | 608 | 0 | 0% | A | 376 | 4 | 1.05% | W | Somatic | 0.0216705 |
| exonic | TMEM99 | nonsynonymous SNV | NM_001195386:c.T235C:p.Y79H |  |  | 0.3 | 0.3 | 0.3 | rs10558 |  | 0.121 | 0.857777 | 5.28E-04 | 0.804014 | chr17 | 38991003 | 38991003 | T | C | het | . | . | . | 362 | 0 | 0% | T | 250 | 6 | 2.34% | Y | Somatic | 0.0048803 |
| exonic | TMEM99 | synonymous SNV | NM_001195386:c.T264A:p.G88G |  |  | 0.54 | 0.54 | 0.54 | rs6694 |  |  |  |  |  | chr17 | 38991032 | 38991032 | T | A | het | . | . | . | 367 | 0 | 0% | T | 259 | 4 | 1.52% | W | Somatic | 0.0299676 |
| exonic | TIE1 | nonsynonymous SNV | NM_005424:c.A1946C:p.H649P |  |  |  |  |  |  | 0.02 | 0.41 | 0.997797 | 0.493896 | 0.77661 | chr1 | 43778824 | 43778824 | A | C | het | . | . | . | 68 | 1 | 1.45% | A | 89 | 10 | 10.10% | M | Somatic | 0.0222609 |
| intronic | TTLL6 |  |  |  |  |  |  |  |  |  |  |  |  |  | chr17 | 46863701 | 46863701 | C | T | het | . | . | . | 387 | 0 | 0% | C | 305 | 3 | 0.97% | Y | Somatic | 0.0865631 |
| exonic | ZNF491 | nonsynonymous SNV | NM_152356:c.G1037A:p.G346E |  |  |  |  |  |  | 1 | 0.001 | 0.188786 | 3.49E-04 | 0.646635 | chr19 | 11917805 | 11917805 | G | A | het | . | . | . | 192 | 3 | 1.54% | G | 89 | 10 | 10.10% | R | Somatic | 0.0014311 |
| exonic | INTS2 | nonsynonymous SNV | NM_020748:c.C3052G:p.Q1018E | 547;Name=lod=222 |  |  |  |  |  | 0 |  |  |  |  | chr17 | 59947100 | 59947100 | G | C | het | . | . | . | 403 | 0 | 0% | G | 174 | 5 | 2.79% | S | Somatic | 0.0026464 |
| exonic | CD163 | synonymous SNV | NM_004244:c.T168G:p.G56G |  |  |  |  |  |  |  |  |  |  |  | chr12 | 7654024 | 7654024 | A | C | het | . | . | . | 150 | 2 | 1.32% | A | 98 | 11 | 10.09% | M | Somatic | 0.0015861 |
| exonic | PRKAR1A | nonsynonymous SNV | NM_002734:c.G478A:p.A160T | 622;Name=lod=449 |  |  |  |  |  | 0.28 | 0.001 | 0.999777 | 0.999999 | 1 | chr17 | 66520194 | 66520194 | G | A | het | . | . | . | 373 | 1 | 0.27% | G | 164 | 4 | 2.38% | R | Somatic | 0.0340414 |
| exonic | MFSD11 | nonsynonymous SNV | NM_001242532:c.C470T:p.A157V | 581;Name=lod=306 |  |  |  |  |  | 0.26 | 0.028 | 0.998803 | 0.999743 | 0.999972 | chr17 | 74739512 | 74739512 | C | T | het | . | . | . | 237 | 0 | 0% | C | 152 | 4 | 2.56% | Y | Somatic | 0.0242521 |
| intronic | ALPK1 |  |  | 446;Name=lod=87 |  |  |  |  |  |  |  |  |  |  | chr4 | 113347620 | 113347620 | C | T | het | . | . | . | 196 | 2 | 1.01% | C | 81 | 9 | 10% | Y | Somatic | 6.40E-04 |
| exonic | ANKRD20A1,ANKRD20A3 | nonsynonymous SNV | NM_001012419:c.G283A:p.V95I |  | 0.99 |  |  |  | rs10909479 | 0.62 | 0 | 0.003388 | 7.00E-06 | 0.565417 | chr9 | 67930466 | 67930466 | G | A | het | . | . | . | 211 | 3 | 1.40% | G | 108 | 12 | 10% | R | Somatic | 4.79E-04 |
| intronic | LAMA1 |  |  |  |  |  |  |  |  |  |  |  |  |  | chr18 | 6980462 | 6980462 | G | A | het | . | . | . | 81 | 0 | 0% | G | 26 | 4 | 13.33% | R | Somatic | 0.0045759 |
| exonic | ZNF763 | nonsynonymous SNV | NM_001012753:c.G944A:p.C315Y |  |  |  |  |  |  | 1 |  |  |  |  | chr19 | 12089674 | 12089674 | G | A | het | . | . | . | 150 | 3 | 1.96% | G | 54 | 6 | 10% | R | Somatic | 0.0163211 |
| exonic | TXNDC2 | nonsynonymous SNV | NM_001098529:c.G1070A:p.G357D |  |  | 0.47 | 0.47 | 0.47 | rs2240906 |  | 0.400427 | 0.249442 | 4.00E-06 | 0.645042 | chr18 | 9887546 | 9887546 | G | A | het | . | . | . | 285 | 1 | 0.35% | G | 341 | 13 | 3.67% | R | Somatic | 0.0028436 |
| ncRNA_exonic | ANKRD20A5P |  |  |  | 0.96 |  |  |  |  |  |  |  |  |  | chr18 | 14183706 | 14183706 | G | A | het | . | . | . | 496 | 1 | 0.20% | G | 240 | 4 | 1.64% | R | Somatic | 0.042754 |
| exonic | IMPACT | nonsynonymous SNV | NM_018439:c.C451G:p.L151V |  |  | 0.87 | 0.87 | 0.87 | rs677688 | 1 | 0 | 0.872071 | 0 | 0.773257 | chr18 | 22020543 | 22020543 | C | G | het | . | . | . | 278 | 0 | 0% | C | 197 | 11 | 5.29% | S | Somatic | 7.56E-05 |
| exonic | SERPINI2 | nonsynonymous SNV | NM_006217:c.A443G:p.E148G | 637;Name=lod=518 |  | 0.4 | 0.4 | 0.4 | rs9841174 | 0.34 | 0.956 | 0.904067 | 0.011214 | 0.981488 | chr3 | 167184878 | 167184878 | T | C | het | . | . | . | 205 | 0 | 0% | T | 84 | 8 | 8.70% | Y | Somatic | 6.82E-05 |
| intronic | MYO5B |  |  |  |  | 0.37 | 0.37 | 0.37 | rs488890 |  |  |  |  |  | chr18 | 47375932 | 47375932 | C | G | het | . | . | . | 681 | 0 | 0% | C | 556 | 4 | 0.71% | S | Somatic | 0.0412196 |
| exonic;splicing | ALPK2;ALPK2 | nonsynonymous SNV | NM_052947:c.G5353T:p.A1785S | 412;Name=lod=63 |  |  |  |  |  | 0.01 | 0.96 | 0.997677 | 0.055325 | 0.999731 | chr18 | 56202066 | 56202066 | C | A | het | . | . | . | 324 | 0 | 0% | C | 252 | 4 | 1.56% | M | Somatic | 0.0374562 |
| exonic | C18orf55 | synonymous SNV | NM_014177:c.G99T:p.A33A |  |  |  |  |  |  |  |  |  |  |  | chr18 | 71816142 | 71816142 | G | T | het | . | . | . | 548 | 1 | 0.18% | G | 221 | 4 | 1.78% | K | Somatic | 0.0269884 |
| exonic | EXOC2 | synonymous SNV | NM_018303:c.C666A:p.I222I | 634;Name=lod=505 |  |  |  |  |  |  |  |  |  |  | chr6 | 610174 | 610174 | G | T | het | . | . | . | 161 | 0 | 0% | G | 54 | 5 | 8.47% | K | Somatic | 0.0012203 |
| exonic | BCAS1 | nonsynonymous SNV | NM_003657:c.C70A:p.Q24K |  |  | 0.55 | 0.55 | 0.55 | rs394732 | 1 | 0 | 0.146882 | 0 | 0.885596 | chr20 | 52675188 | 52675188 | G | T | het | . | . | . | 206 | 0 | 0% | G | 77 | 7 | 8.33% | K | Somatic | 1.42E-04 |
| exonic | PNPLA6 | nonsynonymous SNV | NM_001166114:c.C1850T:p.A617V |  |  |  |  |  |  | 1 | 0 | 0.969453 | 0.920968 | 0.999428 | chr19 | 7615219 | 7615219 | C | T | het | . | . | . | 149 | 0 | 0% | C | 201 | 8 | 3.83% | Y | Somatic | 0.0127474 |
| intronic | SMURF1 |  |  |  |  | 0.31 | 0.31 | 0.31 | rs219826 |  |  |  |  |  | chr7 | 98647139 | 98647139 | C | T | het | . | . | . | 118 | 0 | 0% | C | 55 | 5 | 8.33% | Y | Somatic | 0.0038815 |
| intronic | CCT2 |  |  |  |  | 0.18 | 0.18 | 0.18 | rs11177742 |  |  |  |  |  | chr12 | 69995039 | 69995039 | C | A | het | . | . | . | 125 | 2 | 1.57% | C | 56 | 5 | 8.20% | M | Somatic | 0.0374504 |
| exonic | MUC16 | synonymous SNV | NM_024690:c.A10182C:p.V3394V |  |  | 0.24 | 0.24 | 0.24 | rs1862461 |  |  |  |  |  | chr19 | 9077264 | 9077264 | T | G | het | stomach | carcinoma | adenocarcinoma | 374 | 0 | 0% | T | 150 | 5 | 3.23% | K | Somatic | 0.0020621 |
| exonic | LSR | nonsynonymous SNV | NM_205835:c.C1718T:p.A573V | 457;Name=lod=96 |  |  |  |  |  | 0 | 0.834 | 0.966118 | 0.983681 | 1 | chr19 | 35758734 | 35758734 | C | T | het | . | . | . | 542 | 0 | 0% | C | 374 | 5 | 1.32% | Y | Somatic | 0.0116176 |
| UTR3 | ZNF573 |  |  |  |  | 0.58 | 0.58 | 0.58 | rs1291 |  |  |  |  |  | chr19 | 38229378 | 38229378 | G | A | het | . | . | . | 743 | 0 | 0% | G | 311 | 4 | 1.27% | R | Somatic | 0.0077529 |
| exonic | XRCC1 | nonsynonymous SNV | NM_006297:c.G1136A:p.R379H | 449;Name=lod=89 |  |  |  |  |  | 0.08 | 0.995 | 0.985646 | 0.747518 | 0.999864 | chr19 | 44055786 | 44055786 | C | T | het | . | . | . | 146 | 0 | 0% | C | 200 | 5 | 2.44% | Y | Somatic | 0.0665761 |
| exonic | KRIT1 | synonymous SNV | NM_001013406:c.A1836G:p.V612V | 718;Name=lod=1101 |  | 0.17 | 0.17 | 0.17 | rs11542682 |  |  |  |  |  | chr7 | 91842554 | 91842554 | T | C | het | stomach | carcinoma | adenocarcinoma | 212 | 0 | 0% | T | 91 | 8 | 8.08% | Y | Somatic | 8.64E-05 |
| intronic | TRPM4 |  |  |  |  |  |  |  |  |  |  |  |  |  | chr19 | 49714733 | 49714733 | T | C | het | . | . | . | 287 | 0 | 0% | T | 244 | 6 | 2.40% | Y | Somatic | 0.0098564 |
| exonic | ZNF677 | synonymous SNV | NM_182609:c.C78T:p.D26D |  |  |  |  |  |  |  |  |  |  |  | chr19 | 53747088 | 53747088 | G | A | het | . | . | . | 237 | 0 | 0% | G | 134 | 4 | 2.90% | R | Somatic | 0.0178368 |
| exonic | NOM1 | nonsynonymous SNV | NM_138400:c.G2336A:p.R779H | 522;Name=lod=176 |  | 0.47 | 0.47 | 0.47 | rs2302445 |  | 0.063 | 0.782244 | 0.004173 | 0.986354 | chr7 | 156761818 | 156761818 | G | A | het | . | . | . | 136 | 0 | 0% | G | 94 | 8 | 7.84% | R | Somatic | 9.67E-04 |
| upstream | MIR517B,MIR520D |  |  |  |  |  |  |  |  |  |  |  |  |  | chr19 | 54223336 | 54223336 | C | T | het | . | . | . | 326 | 0 | 0% | C | 232 | 5 | 2.11% | Y | Somatic | 0.0128971 |
| exonic | NLRP7 | stopgain SNV | NM_001127255:c.A2971T:p.K991X |  |  |  |  |  |  | 0.02 | 0.457561 | 0.188352 | 0.999998 | 0.179946 | chr19 | 55438983 | 55438983 | T | A | het | . | . | . | 145 | 0 | 0% | T | 200 | 2 | 0.99% | W | Somatic | 0.3381753 |
| exonic | PEG3 | synonymous SNV | NM_001146186:c.T2307C:p.Y769Y |  |  | 0.2 | 0.2 | 0.2 | rs33931963 |  |  |  |  |  | chr19 | 57327503 | 57327503 | A | G | het | . | . | . | 479 | 0 | 0% | A | 158 | 4 | 2.47% | R | Somatic | 0.0039673 |
| intronic | ITGB8 |  |  |  |  | 0.39 | 0.39 | 0.39 | rs1042277 |  |  |  |  |  | chr7 | 20418926 | 20418926 | T | C | het | stomach | carcinoma | adenocarcinoma | 167 | 0 | 0% | T | 59 | 5 | 7.81% | Y | Somatic | 0.001453 |
| intronic | WT1 |  |  |  |  | 0.46 | 0.46 | 0.46 | rs1799937 |  |  |  |  |  | chr11 | 32410774 | 32410774 | A | G | het | . | . | . | 89 | 0 | 0% | A | 71 | 6 | 7.79% | R | Somatic | 0.008939 |
| intronic | LRP2 |  |  |  |  | 0.32 | 0.32 | 0.32 | rs16823023 |  |  |  |  |  | chr2 | 170055255 | 170055255 | T | C | het | . | . | . | 83 | 0 | 0% | T | 71 | 6 | 7.79% | Y | Somatic | 0.0111872 |
| intronic | NUDT13 |  |  |  |  | 0.16 | 0.16 | 0.16 | rs7083730 |  |  |  |  |  | chr10 | 74890449 | 74890449 | T | A | het | . | . | . | 122 | 0 | 0% | T | 61 | 5 | 7.58% | W | Somatic | 0.004818 |
| exonic | MOAP1 | nonsynonymous SNV | NM_022151:c.G1003A:p.A335T |  |  |  |  |  |  | 0.19 | 0.036 | 0.997557 | 0.107585 | 0.855669 | chr14 | 93649585 | 93649585 | C | T | het | . | . | . | 103 | 0 | 0% | C | 61 | 5 | 7.58% | Y | Somatic | 0.0082578 |
| intronic | GREB1 |  |  |  |  |  |  |  |  |  |  |  |  |  | chr2 | 11778748 | 11778748 | G | A | het | . | . | . | 50 | 0 | 0% | G | 28 | 5 | 15.15% | R | Somatic | 0.0081743 |
| exonic;splicing | PICALM;PICALM | nonsynonymous SNV | NM_001008660:c.G350T:p.G117V | 681;Name=lod=778 |  |  |  |  |  | 0 | 1 | 0.978073 | 0.999993 | 1 | chr11 | 85733512 | 85733512 | C | A | het | . | . | . | 212 | 0 | 0% | C | 110 | 9 | 7.56% | M | Somatic | 8.21E-05 |
| exonic | NRBP1 | synonymous SNV | NM_013392:c.G1530T:p.L510L | 642;Name=lod=542 |  |  |  |  |  |  |  |  |  |  | chr2 | 27664601 | 27664601 | G | T | het | . | . | . | 412 | 0 | 0% | G | 432 | 9 | 2.04% | K | Somatic | 0.0025358 |
| exonic | IFT172 | nonsynonymous SNV | NM_015662:c.G1267T:p.G423W | 619;Name=lod=439 |  |  |  |  |  | 0 | 0.991 | 0.999115 | 0.999993 | 0.999999 | chr2 | 27700142 | 27700142 | C | A | het | . | . | . | 245 | 0 | 0% | C | 118 | 4 | 3.28% | M | Somatic | 0.0118122 |
| exonic | PTGR2 | nonsynonymous SNV | NM_001146154:c.G889C:p.E297Q | 603;Name=lod=377 |  |  |  |  |  | 0.29 | 0 | 0.989978 | 0.273895 | 0.995145 | chr14 | 74347945 | 74347945 | G | C | het | . | . | . | 176 | 0 | 0% | G | 86 | 7 | 7.53% | S | Somatic | 5.07E-04 |
| exonic | BIRC6 | stopgain SNV | NM_016252:c.C8383T:p.R2795X | 681;Name=lod=781 |  |  |  |  |  | 1 |  |  |  |  | chr2 | 32718649 | 32718649 | C | T | het | . | . | . | 226 | 0 | 0% | C | 105 | 5 | 4.55% | Y | Somatic | 0.0035336 |
| exonic | FEZ2 | nonsynonymous SNV | NM_001042548:c.G623A:p.S208N | 502;Name=lod=146 |  |  |  |  |  | 0.35 |  |  |  |  | chr2 | 36808444 | 36808444 | C | T | het | . | . | . | 410 | 0 | 0% | C | 227 | 4 | 1.73% | Y | Somatic | 0.0165863 |
| exonic | CAMKMT | synonymous SNV | NM_024766:c.C726T:p.S242S | 816;Name=lod=2762 |  |  |  |  |  |  |  |  |  |  | chr2 | 44981221 | 44981221 | C | T | het | . | . | . | 417 | 0 | 0% | C | 235 | 6 | 2.49% | Y | Somatic | 0.0023197 |
| exonic | CUBN | synonymous SNV | NM_001081:c.A5856G:p.S1952S |  |  | 0.53 | 0.53 | 0.53 | rs1801234 |  |  |  |  |  | chr10 | 16979661 | 16979661 | T | C | het | . | . | . | 110 | 0 | 0% | T | 63 | 5 | 7.35% | Y | Somatic | 0.0074085 |
| exonic | PSME4 | nonsynonymous SNV | NM_014614:c.A2614G:p.I872V | 583;Name=lod=312 |  | 0.1 | 0.1 | 0.1 | rs2302878 | 0.17 | 0.440943 | 0.999077 | 0.006604 | 0.999998 | chr2 | 54135936 | 54135936 | T | C | het | . | . | . | 332 | 0 | 0% | T | 161 | 4 | 2.42% | Y | Somatic | 0.0118539 |
| exonic | PUS7L | stoploss SNV | NM_001098614:c.T2104A:p.X702K | 265;Name=lod=16 |  |  |  |  |  | 0.92 | 0.414116 | 0.998924 | 0 | 0.859013 | chr12 | 44124181 | 44124181 | A | T | het | . | . | . | 117 | 0 | 0% | A | 52 | 4 | 7.14% | W | Somatic | 0.0101906 |
| exonic | MEIS1 | synonymous SNV | NM_002398:c.C564T:p.D188D | 755;Name=lod=1565 |  |  |  |  |  |  |  |  |  |  | chr2 | 66670114 | 66670114 | C | T | het | . | . | . | 288 | 1 | 0.35% | C | 159 | 8 | 4.79% | Y | Somatic | 0.0017887 |
| exonic | TMC2 | synonymous SNV | NM_080751:c.G387A:p.R129R |  |  |  |  |  |  |  |  |  |  |  | chr20 | 2539406 | 2539406 | G | A | het | . | . | . | 71 | 0 | 0% | G | 52 | 4 | 7.14% | R | Somatic | 0.0355398 |
| exonic | ARHGAP25 | nonsynonymous SNV | NM_001166276:c.T1649C:p.M550T | 325;Name=lod=28 |  | 0.38 | 0.38 | 0.38 | rs10177248 | 1 | 0.001 | 0.088914 | 7.70E-05 | 0.390649 | chr2 | 69049941 | 69049941 | T | C | het | . | . | . | 255 | 0 | 0% | T | 209 | 5 | 2.34% | Y | Somatic | 0.0192778 |
| exonic | ALMS1 | nonsynonymous SNV | NM_015120:c.G7721A:p.S2574N | 482;Name=lod=121 |  | 0.15 | 0.15 | 0.15 | rs3820700 | 0.28 | 0.677 | 0.999728 | 0.020463 | 0.946566 | chr2 | 73716810 | 73716810 | G | A | het | . | . | . | 220 | 0 | 0% | G | 122 | 6 | 4.69% | R | Somatic | 0.002296 |
| intronic | C2orf55 |  |  |  |  |  |  |  |  |  |  |  |  |  | chr2 | 99449302 | 99449302 | G | A | het | . | . | . | 648 | 0 | 0% | G | 546 | 4 | 0.73% | R | Somatic | 0.0441624 |
| exonic | RANBP2 | nonsynonymous SNV | NM_006267:c.C2518T:p.R840C | 433;Name=lod=77 | 0.97 |  |  |  |  | 0 | 0.854 | 0.997471 | 0.252491 | 0.908889 | chr2 | 109374920 | 109374920 | C | T | het | . | . | . | 438 | 0 | 0% | C | 197 | 4 | 1.99% | Y | Somatic | 0.0095902 |
| exonic;splicing | CCDC93;CCDC93 | nonsynonymous SNV | NM_019044:c.G1223A:p.R408Q | 614;Name=lod=418 |  |  |  |  |  | 0.01 | 0.952 | 0.999025 | 0.955332 | 1 | chr2 | 118705682 | 118705682 | C | T | het | lung | carcinoma | adenocarcinoma | 439 | 0 | 0% | C | 313 | 4 | 1.26% | Y | Somatic | 0.0305739 |
| exonic | DNMBP | synonymous SNV | NM_015221:c.C2883T:p.N961N | 584;Name=lod=314 |  | 0.25 | 0.25 | 0.25 | rs7919323 |  |  |  |  |  | chr10 | 101657880 | 101657880 | G | A | het | . | . | . | 118 | 0 | 0% | G | 66 | 5 | 7.04% | R | Somatic | 0.0068335 |
| exonic | UBXN4 | nonsynonymous SNV | NM_014607:c.G1480A:p.E494K | 648;Name=lod=575 |  |  |  |  |  | 0.01 | 0.98 | 0.999564 | 0.995543 | 1 | chr2 | 136540410 | 136540410 | G | A | het | . | . | . | 314 | 1 | 0.32% | G | 177 | 5 | 2.75% | R | Somatic | 0.0267088 |
| exonic | KLHL24 | synonymous SNV | NM_017644:c.A72T:p.R24R | 699;Name=lod=927 |  | 0.37 | 0.37 | 0.37 | rs3755648 |  |  |  |  |  | chr3 | 183368216 | 183368216 | A | T | het | stomach | carcinoma | adenocarcinoma | 152 | 0 | 0% | A | 66 | 5 | 7.04% | W | Somatic | 0.002964 |
| exonic | NR4A2 | nonsynonymous SNV | NM_006186:c.G1349A:p.R450Q | 544;Name=lod=217 |  |  |  |  |  | 0 | 1 | 0.999202 | 0.999993 | 1 | chr2 | 157183242 | 157183242 | C | T | het | . | . | . | 517 | 0 | 0% | C | 428 | 4 | 0.93% | Y | Somatic | 0.0426158 |
| exonic | OR52N5 | nonsynonymous SNV | NM_001001922:c.G397A:p.V133I | 519;Name=lod=172 |  | 0.14 | 0.14 | 0.14 | rs12360738 | 0.06 | 0.011 | 0.217756 | 0.005906 | 0.722768 | chr11 | 5799468 | 5799468 | C | T | het | . | . | . | 108 | 0 | 0% | C | 53 | 4 | 7.02% | Y | Somatic | 0.0132675 |
| ncRNA_exonic | SNORD9 |  |  |  |  | 0.81 | 0.81 | 0.81 | rs1998332 |  |  |  |  |  | chr14 | 21860360 | 21860360 | G | A | het | . | . | . | 190 | 0 | 0% | G | 67 | 5 | 6.94% | R | Somatic | 0.0014132 |
| exonic | DNAH7 | synonymous SNV | NM_018897:c.G1152A:p.T384T | 491;Name=lod=132 |  | 0.65 | 0.65 | 0.65 | rs6434811 |  |  |  |  |  | chr2 | 196866420 | 196866420 | C | T | het | . | . | . | 148 | 0 | 0% | C | 67 | 5 | 6.94% | Y | Somatic | 0.0034104 |
| exonic | DPP4 | nonsynonymous SNV | NM_001935:c.A2080C:p.N694H | 786;Name=lod=2089 |  |  |  |  |  | 0.09 | 0.009 | 0.970354 | 0.816975 | 0.999998 | chr2 | 162851855 | 162851855 | T | G | het | . | . | . | 225 | 0 | 0% | T | 118 | 5 | 4.07% | K | Somatic | 0.005229 |
| exonic | XIRP2 | stopgain SNV | NM_001199145:c.C1147T:p.Q383X |  |  |  |  |  |  | 0.12 | 0.722092 | 0.924843 | 1 | 0.993714 | chr2 | 168114869 | 168114869 | C | T | het | . | . | . | 318 | 0 | 0% | C | 183 | 4 | 2.14% | Y | Somatic | 0.0184226 |
| exonic | STK31 | synonymous SNV | NM_031414:c.A69G:p.Q23Q | 439;Name=lod=81 |  | 0.39 | 0.39 | 0.39 | rs7779633 |  |  |  |  |  | chr7 | 23751736 | 23751736 | A | G | het | . | . | . | 130 | 1 | 0.76% | A | 67 | 5 | 6.94% | R | Somatic | 0.0220498 |
| exonic | TTN | nonsynonymous SNV | NM_003319:c.G33148A:p.D11050N | 555;Name=lod=241 |  |  |  |  |  |  |  |  |  |  | chr2 | 179456109 | 179456109 | C | T | het | . | . | . | 648 | 0 | 0% | C | 261 | 4 | 1.51% | Y | Somatic | 0.0069836 |
| exonic | TTN | nonsynonymous SNV | NM_133378:c.C23855T:p.S7952L | 469;Name=lod=107 |  |  |  |  |  |  |  |  |  |  | chr2 | 179577062 | 179577062 | G | A | het | . | . | . | 470 | 1 | 0.21% | G | 184 | 4 | 2.13% | R | Somatic | 0.0250914 |
| intronic | TTN |  |  |  |  | 0.08 | 0.08 | 0.08 | rs2562837 |  |  |  |  |  | chr2 | 179579694 | 179579694 | T | A | het | . | . | . | 301 | 0 | 0% | T | 107 | 8 | 6.96% | W | Somatic | 2.85E-05 |
| exonic | SESTD1 | nonsynonymous SNV | NM_178123:c.G803A:p.R268H | 506;Name=lod=152 |  |  |  |  |  | 0 | 0.958 | 0.999259 | 0.780159 | 1 | chr2 | 180008365 | 180008365 | C | T | het | . | . | . | 260 | 0 | 0% | C | 105 | 6 | 5.41% | Y | Somatic | 6.51E-04 |
| exonic | SSFA2 | synonymous SNV | NM_001130445:c.G312A:p.V104V | 611;Name=lod=406 |  |  |  |  |  |  |  |  |  |  | chr2 | 182761639 | 182761639 | G | A | het | . | . | . | 711 | 0 | 0% | G | 349 | 4 | 1.13% | R | Somatic | 0.0119778 |
| exonic | YTHDF2 | synonymous SNV | NM_001172828:c.T321C:p.P107P | 861;Name=lod=4191 | 0.98 |  |  |  |  |  |  |  |  |  | chr1 | 29069253 | 29069253 | T | C | het | . | . | . | 88 | 0 | 0% | T | 54 | 4 | 6.90% | Y | Somatic | 0.0233579 |
| exonic | KIAA0284 | nonsynonymous SNV | NM_015005:c.C2728T:p.R910W |  |  |  |  |  |  | 0.03 |  |  |  |  | chr14 | 105353514 | 105353514 | C | T | het | . | . | . | 73 | 0 | 0% | C | 54 | 4 | 6.90% | Y | Somatic | 0.0362109 |
| exonic | CASP8 | nonsynonymous SNV | NM_033356:c.G820A:p.V274I |  |  |  |  |  |  | 0.01 | 0.04 | 0.992723 | 1.51E-04 | 1 | chr2 | 202149601 | 202149601 | G | A | het | . | . | . | 122 | 0 | 0% | G | 45 | 4 | 8.16% | R | Somatic | 0.006161 |
| exonic | GOLGB1 | stopgain SNV | NM_004487:c.C6817T:p.Q2273X | 425;Name=lod=71 |  |  |  |  |  | 0.41 | 0.735463 | 0.999752 | 1 | 0.999682 | chr3 | 121411379 | 121411379 | G | A | het | . | . | . | 186 | 0 | 0% | G | 54 | 4 | 6.90% | R | Somatic | 0.0029446 |
| intronic | CD28 |  |  |  |  |  |  |  |  |  |  |  |  |  | chr2 | 204599504 | 204599504 | C | T | het | . | . | . | 248 | 0 | 0% | C | 169 | 4 | 2.31% | Y | Somatic | 0.0279317 |
| UTR3 | ZPBP |  |  |  |  |  |  |  |  |  |  |  |  |  | chr7 | 49977112 | 49977112 | G | A | het | . | . | . | 182 | 0 | 0% | G | 68 | 5 | 6.85% | R | Somatic | 0.001739 |
| exonic | RNF17 | synonymous SNV | NM_001184993:c.T582C:p.F194F | 456;Name=lod=95 |  | 0.46 | 0.46 | 0.46 | rs9707144 |  |  |  |  |  | chr13 | 25356053 | 25356053 | T | C | het | . | . | . | 115 | 0 | 0% | T | 69 | 5 | 6.76% | Y | Somatic | 0.0084547 |
| exonic | GIGYF2 | nonsynonymous SNV | NM_001103148:c.C1360A:p.P454T | 376;Name=lod=45 |  | 0.09 | 0.09 | 0.09 | rs2289912 | 0.17 | 0.855 | 0.886507 | 0.912796 | 0.996555 | chr2 | 233659553 | 233659553 | C | A | het | stomach | carcinoma | adenocarcinoma | 480 | 1 | 0.21% | C | 260 | 6 | 2.26% | M | Somatic | 0.00962 |
| splicing | GIGYF2(NM_001103148:exon26:c.3443-1G>A,NM_001103146:exon27:c.3461-1G>A,NM_001103147:exon29:c.3524-1G>A,NM_015575:exon29:c.3461-1G>A) |  |  | 712;Name=lod=1039 |  |  |  |  |  |  |  |  |  |  | chr2 | 233712057 | 233712057 | G | A | het | . | . | . | 308 | 0 | 0% | G | 155 | 4 | 2.52% | R | Somatic | 0.013104 |
| intronic | ATG16L1 |  |  |  |  |  |  |  |  |  |  |  |  |  | chr2 | 234191292 | 234191292 | G | A | het | . | . | . | 214 | 0 | 0% | G | 104 | 5 | 4.59% | R | Somatic | 0.0041136 |
| exonic | UGT1A7 | synonymous SNV | NM_019077:c.G756A:p.L252L | 371;Name=lod=43 |  | 0.16 | 0.16 | 0.16 | rs17864686 |  |  |  |  |  | chr2 | 234591339 | 234591339 | G | A | het | . | . | . | 570 | 0 | 0% | G | 276 | 4 | 1.43% | R | Somatic | 0.011606 |
| exonic | HDLBP | nonsynonymous SNV | NM_001243900:c.C1723T:p.R575C | 295;Name=lod=21 |  |  |  |  |  | 0 | 1 | 0.965329 | 0.999997 | 1 | chr2 | 242186295 | 242186295 | G | A | het | . | . | . | 243 | 0 | 0% | G | 133 | 4 | 2.92% | R | Somatic | 0.0164226 |
| exonic | 2-Sep | synonymous SNV | NM_004404:c.C411T:p.N137N | 410;Name=lod=62 |  |  |  |  |  |  |  |  |  |  | chr2 | 242276866 | 242276866 | C | T | het | . | . | . | 267 | 1 | 0.37% | C | 175 | 5 | 2.78% | Y | Somatic | 0.0407489 |
| exonic | THAP4 | synonymous SNV | NM_015963:c.G684A:p.A228A |  |  |  |  |  | rs143045643 |  |  |  |  |  | chr2 | 242572888 | 242572888 | C | T | het | . | . | . | 245 | 1 | 0.41% | C | 183 | 6 | 3.17% | Y | Somatic | 0.0285434 |
| intronic | LAMA1 |  |  |  |  | 0.52 | 0.52 | 0.52 | rs523711 |  |  |  |  |  | chr18 | 6985507 | 6985507 | G | A | het | . | . | . | 172 | 0 | 0% | G | 69 | 5 | 6.76% | R | Somatic | 0.0022353 |
| exonic;splicing | PLCB4;PLCB4 | nonsynonymous SNV | NM_000933:c.G2488A:p.D830N | 536;Name=lod=201 |  |  |  |  |  | 0.02 | 0.07 | 0.999636 | 0.565275 | 1 | chr20 | 9404599 | 9404599 | G | A | het | . | . | . | 117 | 0 | 0% | G | 42 | 4 | 8.70% | R | Somatic | 0.0057576 |
| exonic | PAK7 | nonsynonymous SNV | NM_177990:c.G1532A:p.S511N | 529;Name=lod=189 |  | 0.54 | 0.54 | 0.54 | rs2297345 | 1 | 0 | 0.980503 | 2.36E-04 | 1 | chr20 | 9543622 | 9543622 | C | T | het | . | . | . | 306 | 0 | 0% | C | 140 | 4 | 2.78% | Y | Somatic | 0.0101897 |
| exonic | SPTLC3 | nonsynonymous SNV | NM_018327:c.G392A:p.C131Y | 579;Name=lod=302 |  |  |  |  |  | 0 | 0.66 | 0.999567 | 0.712069 | 1 | chr20 | 13052992 | 13052992 | G | A | het | . | . | . | 640 | 1 | 0.16% | G | 258 | 4 | 1.53% | R | Somatic | 0.0268563 |
| exonic | ESF1 | nonsynonymous SNV | NM_016649:c.C1328T:p.P443L | 681;Name=lod=784 |  |  |  |  |  | 0.12 | 0.011 | 0.963778 | 0.00765 | 0.999877 | chr20 | 13752052 | 13752052 | G | A | het | . | . | . | 460 | 0 | 0% | G | 210 | 4 | 1.87% | R | Somatic | 0.0099688 |
| exonic | BFSP1 | nonsynonymous SNV | NM_001161705:c.C1400T:p.A467V |  |  |  |  |  | rs145703098 | 0.39 | 0 | 0.001884 | 7.80E-05 | 0.187395 | chr20 | 17474942 | 17474942 | G | A | het | . | . | . | 217 | 0 | 0% | G | 289 | 6 | 2.03% | R | Somatic | 0.0357981 |
| exonic | CCL20 | synonymous SNV | NM_001130046:c.C219T:p.C73C | 358;Name=lod=38 |  |  |  |  | rs150823624 |  |  |  |  |  | chr2 | 228681053 | 228681053 | C | T | het | . | . | . | 200 | 0 | 0% | C | 97 | 7 | 6.73% | Y | Somatic | 4.78E-04 |
| exonic | SLC35B3 | synonymous SNV | NM_001142540:c.G501T:p.L167L | 730;Name=lod=1237 |  |  |  |  |  |  |  |  |  |  | chr6 | 8422776 | 8422776 | C | A | het | . | . | . | 168 | 0 | 0% | C | 56 | 4 | 6.67% | M | Somatic | 0.0044469 |
| exonic | KCNH1 | nonsynonymous SNV | NM_002238:c.C2587T:p.R863C | 540;Name=lod=208 |  |  |  |  |  |  | 1 | 0.999109 | 0.999601 | 1 | chr1 | 210856925 | 210856925 | G | A | het | . | . | . | 108 | 0 | 0% | G | 87 | 6 | 6.45% | R | Somatic | 0.0089735 |
| exonic | ADNP | synonymous SNV | NM_181442:c.G1956A:p.T652T |  |  |  |  |  |  |  |  |  |  |  | chr20 | 49509295 | 49509295 | C | T | het | . | . | . | 305 | 1 | 0.33% | C | 103 | 5 | 4.63% | Y | Somatic | 0.0053422 |
| exonic | PSMA1 | nonsynonymous SNV | NM_002786:c.G365A:p.R122Q | 620;Name=lod=442 |  |  |  |  |  | 0.04 | 0.999 | 0.998703 | 0.984124 | 1 | chr11 | 14535412 | 14535412 | C | T | het | . | . | . | 127 | 0 | 0% | C | 58 | 4 | 6.45% | Y | Somatic | 0.010833 |
| exonic | ERGIC2 | synonymous SNV | NM_016570:c.C192T:p.Y64Y | 660;Name=lod=643 |  |  |  |  |  |  |  |  |  |  | chr12 | 29523070 | 29523070 | G | A | het | . | . | . | 147 | 0 | 0% | G | 58 | 4 | 6.45% | R | Somatic | 0.0072223 |
| exonic | DIDO1 | synonymous SNV | NM_001193369:c.G5340A:p.P1780P |  |  |  |  |  |  |  |  |  |  |  | chr20 | 61511968 | 61511968 | C | T | het | . | . | . | 338 | 0 | 0% | C | 296 | 4 | 1.33% | Y | Somatic | 0.0483698 |
| exonic | SLC17A9 | synonymous SNV | NM_022082:c.C984T:p.C328C | 419;Name=lod=67 |  |  |  |  |  |  |  |  |  |  | chr20 | 61597000 | 61597000 | C | T | het | . | . | . | 490 | 0 | 0% | C | 421 | 5 | 1.17% | Y | Somatic | 0.0214827 |
| exonic;splicing | PRPF6;PRPF6 | nonsynonymous SNV | NM_012469:c.C1910T:p.A637V | 505;Name=lod=151 |  |  |  |  |  | 0.05 | 0.012 | 0.998833 | 0.999997 | 1 | chr20 | 62657293 | 62657293 | C | T | het | . | . | . | 683 | 0 | 0% | C | 571 | 5 | 0.87% | Y | Somatic | 0.0198554 |
| exonic | VPS13B | stopgain SNV | NM_181661:c.T1239G:p.Y413X |  |  | 0.68 | 0.68 | 0.68 | rs7460625 |  |  |  |  |  | chr8 | 100133706 | 100133706 | T | G | het | . | . | . | 210 | 0 | 0% | T | 118 | 8 | 6.35% | K | Somatic | 3.39E-04 |
| exonic | ZNF211 | synonymous SNV | NM_198855:c.G963C:p.S321S |  |  | 0.23 | 0.23 | 0.23 | rs11880050 |  |  |  |  |  | chr19 | 58152817 | 58152817 | G | C | het | . | . | . | 208 | 1 | 0.48% | G | 89 | 6 | 6.32% | S | Somatic | 0.0043391 |
| UTR5 | LIMA1 |  |  | 383;Name=lod=48 |  |  |  |  |  |  |  |  |  |  | chr12 | 50594671 | 50594671 | C | A | het | . | . | . | 130 | 0 | 0% | C | 60 | 4 | 6.25% | M | Somatic | 0.0111058 |
| exonic | KCNJ15 | nonsynonymous SNV | NM_170736:c.G293A:p.G98D |  |  | 0.31 | 0.31 | 0.31 | rs2230033 | 0.36 | 0.004 | 0.987148 | 0.196507 | 0.863858 | chr21 | 39671476 | 39671476 | G | A | het | . | . | . | 180 | 0 | 0% | G | 91 | 6 | 6.19% | R | Somatic | 0.0016633 |
| intronic | TXNRD2 |  |  |  |  |  |  |  |  |  |  |  |  |  | chr22 | 19870987 | 19870987 | G | A | het | . | . | . | 307 | 0 | 0% | G | 229 | 3 | 1.29% | R | Somatic | 0.0791558 |
| exonic | SEZ6L | synonymous SNV | NM_001184773:c.G576A:p.A192A |  |  |  |  |  |  |  |  |  |  |  | chr22 | 26688853 | 26688853 | G | A | het | large_intestine | carcinoma | adenocarcinoma | 212 | 0 | 0% | G | 275 | 6 | 2.14% | R | Somatic | 0.033503 |
| exonic | ZC3H7B | synonymous SNV | NM_017590:c.C1530T:p.D510D | 681;Name=lod=782 |  |  |  |  |  |  |  |  |  |  | chr22 | 41742077 | 41742077 | C | T | het | . | . | . | 381 | 0 | 0% | C | 529 | 6 | 1.12% | Y | Somatic | 0.0392335 |
| exonic | APBB1IP | nonsynonymous SNV | NM_019043:c.G835A:p.G279R | 499;Name=lod=142 |  |  |  |  |  | 0 | 0.754 | 0.999534 | 0.947537 | 0.999994 | chr10 | 26822389 | 26822389 | G | A | het | . | . | . | 70 | 0 | 0% | G | 61 | 4 | 6.15% | R | Somatic | 0.0511636 |
| exonic | APPL1 | nonsynonymous SNV | NM_012096:c.G1399A:p.G467S | 550;Name=lod=229 |  |  |  |  |  | 0.45 | 0 | 0.999664 | 0.989089 | 1 | chr3 | 57291425 | 57291425 | G | A | het | . | . | . | 180 | 0 | 0% | G | 61 | 4 | 6.15% | R | Somatic | 0.0046222 |
| intronic | DAZL |  |  |  |  |  |  |  |  |  |  |  |  |  | chr3 | 16646693 | 16646693 | C | T | het | . | . | . | 524 | 0 | 0% | C | 455 | 4 | 0.87% | Y | Somatic | 0.0472062 |
| ncRNA_exonic | SNORA23 |  |  | 489;Name=lod=130 |  | 0.44 | 0.44 | 0.44 | rs2290423 |  |  |  |  |  | chr11 | 9450377 | 9450377 | T | G | het | . | . | . | 147 | 0 | 0% | T | 62 | 4 | 6.06% | K | Somatic | 0.0086449 |
| exonic | ANKRD30A | nonsynonymous SNV | NM_052997:c.G2583C:p.K861N |  | 0.91 | 0.52 | 0.52 | 0.52 | rs1209750 | 0.44 | 0.017 | 2.00E-05 | 6.70E-05 | 0.570042 | chr10 | 37488689 | 37488689 | G | C | het | . | . | . | 192 | 1 | 0.52% | G | 78 | 5 | 6.02% | S | Somatic | 0.0102901 |
| ncRNA_exonic | ENTPD3-AS1 |  |  |  |  |  |  |  |  |  |  |  |  |  | chr3 | 40433476 | 40433476 | C | T | het | . | . | . | 138 | 0 | 0% | C | 45 | 4 | 8.16% | Y | Somatic | 0.0042949 |
| exonic | SETD2 | synonymous SNV | NM_014159:c.G3168A:p.S1056S | 664;Name=lod=665 |  |  |  |  |  |  |  |  |  |  | chr3 | 47162958 | 47162958 | C | T | het | . | . | . | 264 | 0 | 0% | C | 100 | 4 | 3.85% | Y | Somatic | 0.0061165 |
| intronic | RHOA |  |  |  |  |  |  |  |  |  |  |  |  |  | chr3 | 49405796 | 49405796 | C | T | het | . | . | . | 249 | 0 | 0% | C | 206 | 5 | 2.37% | Y | Somatic | 0.0197863 |
| exonic | C3orf54 | nonsynonymous SNV | NM_203370:c.C832T:p.P278S | 592;Name=lod=341 |  |  |  |  |  | 0 | 0.788836 | 0.998995 | 0.999313 | 0.999864 | chr3 | 49842388 | 49842388 | C | T | het | . | . | . | 340 | 0 | 0% | C | 291 | 6 | 2.02% | Y | Somatic | 0.0099974 |
| exonic | CACNA2D3 | synonymous SNV | NM_018398:c.G1857A:p.A619A | 644;Name=lod=553 |  |  |  |  |  | 0.03 |  |  |  |  | chr3 | 54914835 | 54914835 | G | A | het | . | . | . | 470 | 0 | 0% | G | 182 | 5 | 2.67% | R | Somatic | 0.0017972 |
| intronic | CCDC66 |  |  |  |  | 0.28 | 0.28 | 0.28 | rs17216685 |  |  |  |  |  | chr3 | 56647799 | 56647799 | T | C | het | . | . | . | 247 | 0 | 0% | T | 154 | 4 | 2.53% | Y | Somatic | 0.022628 |
| intronic | SCFD1 |  |  |  |  |  |  |  |  |  |  |  |  |  | chr14 | 31118801 | 31118801 | C | T | het | . | . | . | 116 | 0 | 0% | C | 63 | 4 | 5.97% | Y | Somatic | 0.0169527 |
| exonic | SOX17 | synonymous SNV | NM_022454:c.G1122A:p.E374E | 340;Name=lod=32 |  |  |  |  |  |  |  |  |  |  | chr8 | 55372432 | 55372432 | G | A | het | . | . | . | 76 | 0 | 0% | G | 63 | 4 | 5.97% | R | Somatic | 0.0458924 |
| intronic | SFPQ |  |  |  |  |  |  |  |  |  |  |  |  |  | chr1 | 35654779 | 35654779 | C | G | het | . | . | . | 98 | 0 | 0% | C | 80 | 5 | 5.88% | S | Somatic | 0.0202651 |
| exonic | CHMP2B | nonsynonymous SNV | NM_001244644:c.G346A:p.E116K | 671;Name=lod=714 |  |  |  |  |  | 0.01 | 0.993 | 0.999606 | 0.999969 | 1 | chr3 | 87302598 | 87302598 | G | A | het | lung | carcinoma | adenocarcinoma | 242 | 0 | 0% | G | 93 | 4 | 4.12% | R | Somatic | 0.0064093 |
| intronic | ARL6 |  |  | 503;Name=lod=148 |  |  |  |  |  |  |  |  |  |  | chr3 | 97516865 | 97516865 | C | A | het | . | . | . | 455 | 0 | 0% | C | 179 | 4 | 2.19% | M | Somatic | 0.0066112 |
| exonic | GCET2 | synonymous SNV | NM_001190259:c.G39A:p.R13R | 329;Name=lod=29 |  |  |  |  |  |  |  |  |  |  | chr3 | 111849357 | 111849357 | C | T | het | . | . | . | 291 | 0 | 0% | C | 137 | 6 | 4.20% | Y | Somatic | 0.0011912 |
| intronic | SLC9A10 |  |  |  |  | 0.33 | 0.33 | 0.33 | rs6781137 |  |  |  |  |  | chr3 | 111870228 | 111870228 | C | T | het | . | . | . | 465 | 1 | 0.21% | C | 180 | 4 | 2.17% | Y | Somatic | 0.0243682 |
| exonic | KIAA1407 | stopgain SNV | NM_020817:c.C1207T:p.R403X |  |  |  |  |  | rs138387426 | 1 | 0.726184 | 0.995214 | 1 | 0.951263 | chr3 | 113729825 | 113729825 | G | A | het | lung | carcinoma | adenocarcinoma | 484 | 0 | 0% | G | 217 | 5 | 2.25% | R | Somatic | 0.00298 |
| exonic | KIAA1407 | stopgain SNV | NM_020817:c.G999A:p.W333X | 574;Name=lod=287 |  |  |  |  |  | 0.12 | 0.735384 | 0.998931 | 1 | 1 | chr3 | 113737689 | 113737689 | C | T | het | . | . | . | 486 | 0 | 0% | C | 230 | 5 | 2.13% | Y | Somatic | 0.0035735 |
| exonic | FAM168B | synonymous SNV | NM_001009993:c.G135A:p.A45A | 604;Name=lod=381 |  |  |  |  |  |  |  |  |  |  | chr2 | 131829447 | 131829447 | C | T | het | . | . | . | 101 | 0 | 0% | C | 80 | 5 | 5.88% | Y | Somatic | 0.018666 |
| exonic | GTF2E1 | synonymous SNV | NM_005513:c.G591A:p.V197V | 737;Name=lod=1318 |  |  |  |  |  |  |  |  |  |  | chr3 | 120489717 | 120489717 | G | A | het | . | . | . | 788 | 1 | 0.13% | G | 309 | 6 | 1.90% | R | Somatic | 0.0027693 |
| intronic | SLC13A1 |  |  |  |  | 0.29 | 0.29 | 0.29 | rs2470983 |  |  |  |  |  | chr7 | 122774603 | 122774603 | C | A | het | . | . | . | 104 | 0 | 0% | C | 64 | 4 | 5.88% | M | Somatic | 0.0231303 |
| exonic | GOLGB1 | nonsynonymous SNV | NM_004487:c.G3412A:p.V1138I | 259;Name=lod=15 |  |  |  |  |  | 0.03 | 0.003 | 0.862289 | 2.27E-04 | 0.951808 | chr3 | 121415943 | 121415943 | C | T | het | . | . | . | 426 | 0 | 0% | C | 129 | 4 | 3.01% | Y | Somatic | 0.003095 |
| exonic | TMEM176B | nonsynonymous SNV | NM_001101314:c.C574G:p.L192V |  |  |  |  |  |  | 0.01 | 0.97 | 0.974179 | 0.24121 | 0.458127 | chr7 | 150489179 | 150489179 | G | C | het | . | . | . | 99 | 0 | 0% | G | 148 | 9 | 5.73% | S | Somatic | 0.0112012 |
| exonic | TOPBP1 | nonsynonymous SNV | NM_007027:c.A1369C:p.K457Q | 420;Name=lod=68 |  | 0.73 | 0.73 | 0.73 | rs3192149 | 0.85 |  |  |  |  | chr3 | 133368362 | 133368362 | T | G | het | . | . | . | 296 | 1 | 0.34% | T | 153 | 6 | 3.77% | K | Somatic | 0.0083747 |
| intronic | TF |  |  |  |  | 0.49 | 0.49 | 0.49 | rs1880669 |  |  |  |  |  | chr3 | 133483696 | 133483696 | T | C | het | . | . | . | 356 | 0 | 0% | T | 162 | 6 | 3.57% | Y | Somatic | 0.0010213 |
| splicing | CEP70(NM_024491:exon16:c.1369-1G>A) |  |  | 295;Name=lod=21 |  |  |  |  |  |  |  |  |  |  | chr3 | 138219410 | 138219410 | C | T | het | lung | carcinoma | adenocarcinoma | 497 | 0 | 0% | C | 192 | 4 | 2.04% | Y | Somatic | 0.0062587 |
| exonic | ZBTB38 | stopgain SNV | NM_001080412:c.C1084T:p.Q362X | 415;Name=lod=65 |  |  |  |  |  | 0.13 | 0.734978 | 0.999012 | 1 | 0.997618 | chr3 | 141162314 | 141162314 | C | T | het | . | . | . | 395 | 0 | 0% | C | 331 | 4 | 1.19% | Y | Somatic | 0.0439195 |
| exonic;splicing | CPA3;CPA3 | synonymous SNV | NM_001870:c.G474A:p.K158K | 417;Name=lod=66 |  |  |  |  |  |  |  |  |  |  | chr3 | 148596535 | 148596535 | G | A | het | . | . | . | 220 | 1 | 0.45% | G | 89 | 4 | 4.30% | R | Somatic | 0.0283014 |
| ncRNA_UTR3 | AADACL2 |  |  |  |  |  |  |  |  |  |  |  |  |  | chr3 | 151475389 | 151475389 | G | T | het | . | . | . | 319 | 0 | 0% | G | 131 | 4 | 2.96% | K | Somatic | 0.0075752 |
| splicing | SERPINI2(NM_006217:exon6:c.674-1G>T) |  |  | 374;Name=lod=44 |  |  |  |  |  |  |  |  |  |  | chr3 | 167183187 | 167183187 | C | A | het | . | . | . | 285 | 1 | 0.35% | C | 88 | 4 | 4.35% | M | Somatic | 0.013546 |
| intronic | HEATR1 |  |  |  |  |  |  |  |  |  |  |  |  |  | chr1 | 236724602 | 236724602 | G | A | het | . | . | . | 190 | 1 | 0.52% | G | 99 | 6 | 5.71% | R | Somatic | 0.008977 |
| intronic | AP4E1 |  |  |  |  |  |  |  | rs191927941 |  |  |  |  |  | chr15 | 51240375 | 51240375 | C | G | het | . | . | . | 83 | 0 | 0% | C | 66 | 4 | 5.71% | S | Somatic | 0.0417761 |
| splicing | FNDC3B(NM_022763:exon25:c.3176-2A>G,NM_001135095:exon25:c.3176-2A>G) |  |  | 623;Name=lod=454 |  |  |  |  |  |  |  |  |  |  | chr3 | 172098754 | 172098754 | A | G | het | . | . | . | 260 | 0 | 0% | A | 131 | 2 | 1.50% | R | Somatic | 0.1139586 |
| exonic | EFCAB5 | nonsynonymous SNV | NM_198529:c.C4154T:p.A1385V |  |  |  |  |  |  | 0 |  |  |  |  | chr17 | 28419105 | 28419105 | C | T | het | . | . | . | 89 | 0 | 0% | C | 66 | 4 | 5.71% | Y | Somatic | 0.0357645 |
| exonic | HRASLS | nonsynonymous SNV | NM_020386:c.G143A:p.G48D | 304;Name=lod=23 |  |  |  |  |  | 1 | 0 | 0.996137 | 0.996572 | 0.999973 | chr3 | 192980762 | 192980762 | G | A | het | . | . | . | 618 | 1 | 0.16% | G | 231 | 5 | 2.12% | R | Somatic | 0.0072098 |
| exonic | VPS13A | nonsynonymous SNV | NM_001018037:c.G2059A:p.E687K |  |  |  |  |  |  | 0.22 | 0.011 | 0.173768 | 0.04009 | 0.815368 | chr9 | 79865034 | 79865034 | G | A | het | . | . | . | 168 | 0 | 0% | G | 66 | 4 | 5.71% | R | Somatic | 0.0070344 |
| exonic | TNFSF15 | synonymous SNV | NM_001204344:c.A426G:p.V142V | 528;Name=lod=186 |  | 0.69 | 0.69 | 0.69 | rs3810936 |  |  |  |  |  | chr9 | 117552885 | 117552885 | T | C | het | . | . | . | 96 | 0 | 0% | T | 66 | 4 | 5.71% | Y | Somatic | 0.0300544 |
| intronic | TNIP2 |  |  |  |  |  |  |  |  |  |  |  |  |  | chr4 | 2746248 | 2746248 | G | T | het | . | . | . | 228 | 0 | 0% | G | 179 | 4 | 2.19% | K | Somatic | 0.0385891 |
| exonic | MEIS1 | nonsynonymous SNV | NM_002398:c.A601G:p.I201V | 755;Name=lod=1565 |  |  |  |  |  | 0.32 |  |  |  |  | chr2 | 66670151 | 66670151 | A | G | het | . | . | . | 178 | 0 | 0% | A | 83 | 5 | 5.68% | R | Somatic | 0.0036661 |
| exonic | TBC1D19 | nonsynonymous SNV | NM_018317:c.C865T:p.L289F | 594;Name=lod=345 |  |  |  |  |  | 0.02 | 0.742 | 0.999109 | 0.999827 | 1 | chr4 | 26685345 | 26685345 | C | T | het | . | . | . | 236 | 0 | 0% | C | 120 | 2 | 1.64% | Y | Somatic | 0.1155032 |
| exonic | APBB2 | synonymous SNV | NM_001166050:c.C612T:p.G204G |  |  | 0.33 | 0.33 | 0.33 | rs2292234 |  |  |  |  |  | chr4 | 41015823 | 41015823 | G | A | het | . | . | . | 537 | 0 | 0% | G | 670 | 9 | 1.33% | R | Somatic | 0.0051545 |
| exonic | KCTD8 | nonsynonymous SNV | NM_198353:c.G1253A:p.S418N | 543;Name=lod=215 |  |  |  |  |  | 0.02 | 0.469 | 0.99886 | 0.090577 | 0.999996 | chr4 | 44176976 | 44176976 | C | T | het | lung | carcinoma | adenocarcinoma | 677 | 1 | 0.15% | C | 284 | 7 | 2.41% | Y | Somatic | 0.0012421 |
| exonic | ATP10D | nonsynonymous SNV | NM_020453:c.G3620A:p.S1207N | 615;Name=lod=420 |  |  |  |  |  | 0 | 0.995 | 0.999704 | 0.859457 | 1 | chr4 | 47582467 | 47582467 | G | A | het | . | . | . | 442 | 0 | 0% | G | 205 | 4 | 1.91% | R | Somatic | 0.0104168 |
| exonic | REST | nonsynonymous SNV | NM_001193508:c.C2390T:p.P797L |  |  | 0.25 | 0.25 | 0.25 | rs3796529 | 0.2 | 0.991 | 0.808588 | 0.002267 | 0.413791 | chr4 | 57797414 | 57797414 | C | T | het | prostate | carcinoma | adenocarcinoma | 387 | 1 | 0.26% | C | 287 | 6 | 2.05% | Y | Somatic | 0.0274026 |
| exonic | AMTN | nonsynonymous SNV | NM_212557:c.A134G:p.N45S | 385;Name=lod=49 |  | 0.1 | 0.1 | 0.1 | rs7660807 | 0 | 0.302 | 0.896731 | 0.114324 | 0.958675 | chr4 | 71388551 | 71388551 | A | G | het | . | . | . | 528 | 0 | 0% | A | 253 | 4 | 1.56% | R | Somatic | 0.0113082 |
| exonic | ENAM | synonymous SNV | NM_031889:c.G1893A:p.G631G |  |  |  |  |  | rs143023982 |  |  |  |  |  | chr4 | 71509036 | 71509036 | G | A | het | . | . | . | 410 | 1 | 0.24% | G | 147 | 5 | 3.29% | R | Somatic | 0.0063982 |
| exonic | ZNF548 | nonsynonymous SNV | NM_152909:c.G217A:p.A73T |  |  | 0.91 | 0.91 | 0.91 | rs4801478 | 1 |  |  |  |  | chr19 | 57909872 | 57909872 | G | A | het | . | . | . | 197 | 0 | 0% | G | 100 | 6 | 5.66% | R | Somatic | 0.0016683 |
| exonic | SLC4A4 | nonsynonymous SNV | NM_003759:c.G2834A:p.G945D | 720;Name=lod=1127 |  |  |  |  |  | 0.01 | 0.038 | 0.999448 | 0.999998 | 0.999999 | chr4 | 72425838 | 72425838 | G | A | het | . | . | . | 395 | 1 | 0.25% | G | 196 | 4 | 2% | R | Somatic | 0.0456822 |
| exonic | ANKRD17 | synonymous SNV | NM_198889:c.G6369A:p.P2123P | 618;Name=lod=432 |  |  |  |  |  |  |  |  |  |  | chr4 | 73951003 | 73951003 | C | T | het | . | . | . | 445 | 1 | 0.22% | C | 287 | 6 | 2.05% | Y | Somatic | 0.0175116 |
| exonic | CXCL11 | stopgain SNV | NM_005409:c.C217T:p.R73X | 349;Name=lod=35 |  | 0.001 | 0.001 | 0.001 | rs149207110 | 1 | 0.724772 | 0.934541 | 0.99997 | 0.999925 | chr4 | 76956244 | 76956244 | G | A | het | . | . | . | 234 | 0 | 0% | G | 124 | 4 | 3.12% | R | Somatic | 0.0151594 |
| exonic | WDFY3 | nonsynonymous SNV | NM_014991:c.G8150A:p.G2717D | 450;Name=lod=90 |  |  |  |  |  | 0 | 0.744 | 0.999224 | 1 | 1 | chr4 | 85630129 | 85630129 | C | T | het | . | . | . | 220 | 0 | 0% | C | 129 | 6 | 4.44% | Y | Somatic | 0.0028194 |
| exonic | PTPN13 | nonsynonymous SNV | NM_080684:c.C3379T:p.R1127C | 533;Name=lod=195 |  |  |  |  |  | 0.17 |  |  |  |  | chr4 | 87684278 | 87684278 | C | T | het | . | . | . | 345 | 1 | 0.29% | C | 213 | 5 | 2.29% | Y | Somatic | 0.0343385 |
| exonic | SPP1 | synonymous SNV | NM_000582:c.C201T:p.S67S | 304;Name=lod=23 |  |  |  |  | rs11544547 |  |  |  |  |  | chr4 | 88902653 | 88902653 | C | T | het | . | . | . | 452 | 1 | 0.22% | C | 163 | 4 | 2.40% | Y | Somatic | 0.0202053 |
| exonic | UNC5C | synonymous SNV | NM_003728:c.A1455G:p.S485S |  |  |  |  |  |  |  |  |  |  |  | chr4 | 96140310 | 96140310 | T | C | het | . | . | . | 431 | 0 | 0% | T | 233 | 4 | 1.69% | Y | Somatic | 0.0155864 |
| splicing | NFKB1(NM_001165412:exon12:c.1064-1G>A,NM_003998:exon12:c.1067-1G>A) |  |  | 552;Name=lod=234 |  |  |  |  |  |  |  |  |  |  | chr4 | 103514581 | 103514581 | G | A | het | . | . | . | 475 | 0 | 0% | G | 377 | 3 | 0.79% | R | Somatic | 0.0874061 |
| exonic;splicing | EGF;EGF | nonsynonymous SNV | NM_001178131:c.G1186A:p.G396S | 415;Name=lod=65 |  |  |  |  |  | 0.02 | 0.998 | 0.999397 | 0.769105 | 1 | chr4 | 110883141 | 110883141 | G | A | het | . | . | . | 295 | 0 | 0% | G | 172 | 4 | 2.27% | R | Somatic | 0.0190813 |
| exonic | PITX2 | stopgain SNV | NM_001204398:c.C52T:p.Q18X | 574;Name=lod=287 |  |  |  |  |  | 0 | 0.738075 | 0.999492 | 1 | 1 | chr4 | 111553631 | 111553631 | G | A | het | . | . | . | 350 | 0 | 0% | G | 294 | 4 | 1.34% | R | Somatic | 0.04424 |
| exonic | CAMK2D | nonsynonymous SNV | NM_001221:c.G1255T:p.V419L | 358;Name=lod=38 |  |  |  |  |  | 0 | 0.035 | 0.999101 | 0.999751 | 1 | chr4 | 114378669 | 114378669 | C | A | het | . | . | . | 309 | 1 | 0.32% | C | 159 | 6 | 3.64% | M | Somatic | 0.0082145 |
| exonic | PRDM5 | synonymous SNV | NM_018699:c.A681G:p.L227L |  |  | 0.3 | 0.3 | 0.3 | rs343192 |  |  |  |  |  | chr4 | 121738049 | 121738049 | T | C | het | . | . | . | 756 | 0 | 0% | T | 305 | 6 | 1.93% | Y | Somatic | 5.92E-04 |
| exonic | ANAPC10 | nonsynonymous SNV | NM_014885:c.C419T:p.A140V | 753;Name=lod=1528 |  |  |  |  |  | 0.05 | 0.038 | 0.995103 | 0.99999 | 1 | chr4 | 145916664 | 145916664 | G | A | het | . | . | . | 332 | 0 | 0% | G | 127 | 4 | 3.05% | R | Somatic | 0.0061992 |
| exonic | LAX1 | nonsynonymous SNV | NM_001136190:c.C1129A:p.Q377K |  |  |  |  |  |  | 0.72 | 0.004 | 0.049087 | 0.066039 | 0.013983 | chr1 | 203743789 | 203743789 | C | A | het | . | . | . | 87 | 0 | 0% | C | 67 | 4 | 5.63% | M | Somatic | 0.0388778 |
| splicing | ITGB6 |  |  | 621;Name=lod=445 |  |  |  |  |  |  |  |  |  |  | chr2 | 161056590 | 161056590 | C | T | het | . | . | . | 147 | 0 | 0% | C | 67 | 4 | 5.63% | Y | Somatic | 0.0106147 |
| exonic | C20orf177 | nonsynonymous SNV | NM_022106:c.C299T:p.S100L | 440;Name=lod=82 |  |  |  |  |  | 0 | 0.799 | 0.998951 | 0.063834 | 0.999997 | chr20 | 58519297 | 58519297 | C | T | het | . | . | . | 154 | 0 | 0% | C | 67 | 4 | 5.63% | Y | Somatic | 0.009346 |
| intronic | DPP4 |  |  | 786;Name=lod=2089 |  |  |  |  |  |  |  |  |  |  | chr2 | 162851804 | 162851804 | A | G | het | . | . | . | 174 | 0 | 0% | A | 84 | 5 | 5.62% | R | Somatic | 0.0041128 |
| intergenic | NBLA00301(dist=74490),FBXO8(dist=620339) |  |  | 775;Name=lod=1887 | 0.96 | 0.21 | 0.21 | 0.21 | rs13103221 |  |  |  |  |  | chr4 | 174537471 | 174537471 | G | A | het | . | . | . | 429 | 1 | 0.23% | G | 178 | 6 | 3.26% | R | Somatic | 0.0035842 |
| intronic | AGA |  |  |  |  | 0.14 | 0.14 | 0.14 | rs34241758 |  |  |  |  |  | chr4 | 178361414 | 178361414 | A | C | het | . | . | . | 294 | 0 | 0% | A | 213 | 5 | 2.29% | M | Somatic | 0.0136264 |
| ncRNA_intronic | LOC285501 |  |  |  |  |  |  |  |  |  |  |  |  |  | chr4 | 178897107 | 178897107 | C | T | het | . | . | . | 413 | 1 | 0.24% | C | 145 | 4 | 2.68% | Y | Somatic | 0.0188662 |
| exonic | CDKN2AIP | synonymous SNV | NM_017632:c.G1692A:p.S564S | 664;Name=lod=663 |  |  |  |  |  |  |  |  |  |  | chr4 | 184368529 | 184368529 | G | A | het | . | . | . | 519 | 0 | 0% | G | 229 | 4 | 1.72% | R | Somatic | 0.0090528 |
| exonic | CASP3 | nonsynonymous SNV | NM_032991:c.G67A:p.G23R | 346;Name=lod=34 |  |  |  |  |  | 0.01 | 0.002 | 0.998924 | 0.0484 | 0.964513 | chr4 | 185556559 | 185556559 | C | T | het | . | . | . | 234 | 0 | 0% | C | 91 | 4 | 4.21% | Y | Somatic | 0.0066418 |
| exonic | KLKB1 | nonsynonymous SNV | NM_000892:c.G1709A:p.G570E |  |  |  |  |  |  | 0 | 0.999 | 0.995159 | 0.861511 | 0.999236 | chr4 | 187178503 | 187178503 | G | A | het | . | . | . | 259 | 0 | 0% | G | 165 | 4 | 2.37% | R | Somatic | 0.0237876 |
| exonic | FAT1 | nonsynonymous SNV | NM_005245:c.G6290A:p.G2097D | 395;Name=lod=54 |  |  |  |  |  | 0.08 |  |  |  |  | chr4 | 187541450 | 187541450 | C | T | het | . | . | . | 404 | 1 | 0.25% | C | 205 | 5 | 2.38% | Y | Somatic | 0.0194277 |
| exonic | FAT1 | nonsynonymous SNV | NM_005245:c.A3818G:p.H1273R | 309;Name=lod=24 |  | 0.68 | 0.68 | 0.68 | rs328418 | 1 |  |  |  |  | chr4 | 187557893 | 187557893 | T | C | het | . | . | . | 587 | 1 | 0.17% | T | 399 | 15 | 3.62% | Y | Somatic | 1.49E-05 |
| exonic | CCDC127 | synonymous SNV | NM_145265:c.C729T:p.L243L |  |  |  |  |  |  |  |  |  |  |  | chr5 | 205466 | 205466 | G | A | het | . | . | . | 344 | 0 | 0% | G | 277 | 5 | 1.77% | R | Somatic | 0.0181905 |
| exonic | LRMP | synonymous SNV | NM_001204126:c.G231A:p.T77T |  |  |  |  |  |  |  |  |  |  |  | chr12 | 25232684 | 25232684 | G | A | het | . | . | . | 93 | 0 | 0% | G | 68 | 4 | 5.56% | R | Somatic | 0.0345547 |
| exonic | CLPTM1L | synonymous SNV | NM_030782:c.C714T:p.T238T | 513;Name=lod=162 |  |  |  |  |  |  |  |  |  |  | chr5 | 1335254 | 1335254 | G | A | het | . | . | . | 268 | 0 | 0% | G | 223 | 5 | 2.19% | R | Somatic | 0.0200389 |
| exonic | LPCAT1 | nonsynonymous SNV | NM_024830:c.C511T:p.R171W | 469;Name=lod=107 |  |  |  |  |  | 0 | 0.933 | 0.969241 | 0.99995 | 1 | chr5 | 1489956 | 1489956 | G | A | het | . | . | . | 595 | 1 | 0.17% | G | 539 | 6 | 1.10% | R | Somatic | 0.0485856 |
| exonic | MTRR | nonsynonymous SNV | NM_002454:c.T769A:p.S257T |  |  | 0.03 | 0.03 | 0.03 | rs2303080 | 0.75 | 0 | 2.30E-05 | 1.45E-04 | 0.243768 | chr5 | 7878424 | 7878424 | T | A | het | . | . | . | 569 | 1 | 0.18% | T | 235 | 5 | 2.08% | W | Somatic | 0.0100666 |
| intronic | MTRR |  |  |  |  |  |  |  |  |  |  |  |  |  | chr5 | 7895816 | 7895816 | C | A | het | . | . | . | 499 | 0 | 0% | C | 141 | 8 | 5.37% | M | Somatic | 6.74E-06 |
| exonic | CTNND2 | synonymous SNV | NM_001332:c.G2256A:p.G752G | 558;Name=lod=246 |  | 0.18 | 0.18 | 0.18 | rs2285975 |  |  |  |  |  | chr5 | 11117583 | 11117583 | C | T | het | . | . | . | 327 | 0 | 0% | C | 397 | 10 | 2.46% | Y | Somatic | 0.0026141 |
| exonic | DNAH5 | nonsynonymous SNV | NM_001369:c.G71A:p.G24E | 565;Name=lod=264 |  | 0.51 | 0.51 | 0.51 | rs1530496 | 1 | 0 | 0.975847 | 9.00E-06 | 1 | chr5 | 13931340 | 13931340 | C | T | het | . | . | . | 274 | 1 | 0.36% | C | 132 | 7 | 5.04% | Y | Somatic | 0.0024873 |
| intronic | FAM134B |  |  |  |  | 0.72 | 0.72 | 0.72 | rs162850 |  |  |  |  |  | chr5 | 16477751 | 16477751 | C | A | het | . | . | . | 269 | 0 | 0% | C | 83 | 4 | 4.60% | M | Somatic | 0.0033827 |
| exonic | NIPBL | nonsynonymous SNV | NM_015384:c.G3434A:p.R1145H | 734;Name=lod=1278 |  |  |  |  |  | 0.04 | 0.996 | 0.999718 | 0.95636 | 1 | chr5 | 37000604 | 37000604 | G | A | het | . | . | . | 291 | 0 | 0% | G | 147 | 4 | 2.65% | R | Somatic | 0.0132659 |
| exonic | PTGER4 | synonymous SNV | NM_000958:c.C276T:p.C92C | 713;Name=lod=1050 |  |  |  |  |  |  |  |  |  |  | chr5 | 40681371 | 40681371 | C | T | het | . | . | . | 461 | 0 | 0% | C | 387 | 4 | 1.02% | Y | Somatic | 0.0439873 |
| exonic | ITGA1 | nonsynonymous SNV | NM_181501:c.C1439T:p.T480M | 391;Name=lod=52 |  | 0.08 | 0.08 | 0.08 | rs4145748 | 0.01 | 0.97 | 0.999318 | 0.947779 | 0.999989 | chr5 | 52201722 | 52201722 | C | T | het | stomach | carcinoma | adenocarcinoma | 222 | 1 | 0.45% | C | 98 | 6 | 5.77% | Y | Somatic | 0.0048363 |
| intronic | FAM135B |  |  |  |  |  |  |  |  |  |  |  |  |  | chr8 | 139190770 | 139190770 | C | T | het | . | . | . | 95 | 0 | 0% | C | 68 | 4 | 5.56% | Y | Somatic | 0.0329144 |
| exonic | DHX29 | nonsynonymous SNV | NM_019030:c.C1586T:p.S529L | 558;Name=lod=246 |  |  |  |  |  | 0.02 | 0.025 | 0.999432 | 0.805163 | 0.992221 | chr5 | 54579410 | 54579410 | G | A | het | ovary | carcinoma | serous_carcinoma | 384 | 0 | 0% | G | 166 | 4 | 2.35% | R | Somatic | 0.0086504 |
| UTR3 | AKR1D1 |  |  |  |  | 0.77 | 0.77 | 0.77 | rs1872929 |  |  |  |  |  | chr7 | 137801413 | 137801413 | A | C | het | . | . | . | 128 | 0 | 0% | A | 86 | 5 | 5.49% | M | Somatic | 0.0115991 |
| exonic | IL6ST | nonsynonymous SNV | NM_001190981:c.G1874A:p.G625D | 588;Name=lod=327 | 0.96 |  |  |  |  | 0 | 0.063 | 0.937487 | 9.33E-04 | 0.981073 | chr5 | 55237610 | 55237610 | C | T | het | . | . | . | 293 | 1 | 0.34% | C | 98 | 4 | 3.92% | Y | Somatic | 0.0168436 |
| exonic | HTR1A | synonymous SNV | NM_000524:c.C634T:p.L212L | 588;Name=lod=328 |  |  |  |  |  |  |  |  |  |  | chr5 | 63256913 | 63256913 | G | A | het | . | . | . | 416 | 0 | 0% | G | 326 | 4 | 1.21% | R | Somatic | 0.0379031 |
| exonic | ADAMTS6 | synonymous SNV | NM_197941:c.C501T:p.I167I | 678;Name=lod=761 |  |  |  |  |  |  |  |  |  |  | chr5 | 64756127 | 64756127 | G | A | het | NS | NS | NS | 323 | 0 | 0% | G | 152 | 5 | 3.18% | R | Somatic | 0.0035846 |
| exonic | SLC30A5 | nonsynonymous SNV | NM_022902:c.C2047T:p.R683C | 601;Name=lod=369 |  |  |  |  |  | 0.09 | 0.984 | 0.998991 | 0.999138 | 1 | chr5 | 68423879 | 68423879 | C | T | het | large_intestine | carcinoma | adenocarcinoma | 231 | 0 | 0% | C | 163 | 4 | 2.40% | Y | Somatic | 0.0303519 |
| exonic | CENPH | synonymous SNV | NM_022909:c.C406T:p.L136L |  |  |  |  |  |  |  |  |  |  |  | chr5 | 68498367 | 68498367 | C | T | het | . | . | . | 231 | 0 | 0% | C | 123 | 4 | 3.15% | Y | Somatic | 0.0153559 |
| exonic | HMGCR | nonsynonymous SNV | NM_000859:c.C508T:p.L170F | 395;Name=lod=54 |  |  |  |  |  | 0 | 0.997 | 0.998286 | 0.99999 | 0.999999 | chr5 | 74643086 | 74643086 | C | T | het | . | . | . | 451 | 1 | 0.22% | C | 232 | 5 | 2.11% | Y | Somatic | 0.0201559 |
| exonic | IQGAP2 | synonymous SNV | NM_006633:c.G3741A:p.V1247V |  |  |  |  |  |  |  |  |  |  |  | chr5 | 75979020 | 75979020 | G | A | het | . | . | . | 246 | 1 | 0.40% | G | 123 | 4 | 3.15% | R | Somatic | 0.0472832 |
| exonic | OTP | nonsynonymous SNV | NM_032109:c.G329A:p.R110H | 671;Name=lod=710 |  |  |  |  |  | 0.01 | 1 | 0.999014 | 0.999987 | 1 | chr5 | 76932764 | 76932764 | C | T | het | . | . | . | 413 | 1 | 0.24% | C | 282 | 5 | 1.74% | Y | Somatic | 0.0447774 |
| exonic | MEF2C | nonsynonymous SNV | NM_001193348:c.G8A:p.R3K | 741;Name=lod=1366 |  |  |  |  |  | 0 |  |  |  |  | chr5 | 88119598 | 88119598 | C | T | het | . | . | . | 815 | 1 | 0.12% | C | 290 | 4 | 1.36% | Y | Somatic | 0.0191547 |
| exonic | ANKK1 | synonymous SNV | NM_178510:c.T255C:p.S85S | 313;Name=lod=25 |  | 0.55 | 0.55 | 0.55 | rs17115439 |  |  |  |  |  | chr11 | 113264272 | 113264272 | T | C | het | . | . | . | 102 | 0 | 0% | T | 87 | 5 | 5.43% | Y | Somatic | 0.0226202 |
| exonic | WDR36 | synonymous SNV | NM_139281:c.G2109A:p.R703R | 585;Name=lod=319 |  |  |  |  |  |  |  |  |  |  | chr5 | 110456230 | 110456230 | G | A | het | . | . | . | 371 | 1 | 0.27% | G | 142 | 6 | 4.05% | R | Somatic | 0.0026381 |
| exonic | YTHDC2 | nonsynonymous SNV | NM_022828:c.G3251A:p.G1084D | 614;Name=lod=418 |  |  |  |  |  | 0.49 | 0.001 | 0.999551 | 0.89847 | 0.999997 | chr5 | 112915289 | 112915289 | G | A | het | . | . | . | 384 | 0 | 0% | G | 164 | 4 | 2.38% | R | Somatic | 0.0083674 |
| exonic | CDO1 | nonsynonymous SNV | NM_001801:c.C32T:p.T11I | 374;Name=lod=44 |  |  |  |  |  | 0 | 0.534 | 0.985843 | 0.778854 | 0.999961 | chr5 | 115152063 | 115152063 | G | A | het | . | . | . | 593 | 0 | 0% | G | 482 | 5 | 1.03% | R | Somatic | 0.0184335 |
| exonic | GRAMD3 | nonsynonymous SNV | NM_001146319:c.G331A:p.E111K | 381;Name=lod=47 |  |  |  |  |  | 0.01 | 0.293 | 0.994429 | 0.974023 | 1 | chr5 | 125805451 | 125805451 | G | A | het | . | . | . | 408 | 1 | 0.24% | G | 264 | 5 | 1.86% | R | Somatic | 0.0388179 |
| exonic | LRRC31 | nonsynonymous SNV | NM_024727:c.C971A:p.A324E |  |  | 0.02 | 0.02 | 0.02 | rs3732452 | 0.56 | 0 | 2.00E-06 | 0.165515 | 0.83114 | chr3 | 169572621 | 169572621 | G | T | het | stomach | carcinoma | adenocarcinoma | 160 | 0 | 0% | G | 87 | 5 | 5.43% | K | Somatic | 0.0060434 |
| exonic | ASXL3 | nonsynonymous SNV | NM_030632:c.A5122G:p.M1708V |  |  | 0.66 | 0.66 | 0.66 | rs7232237 | 0 |  |  |  |  | chr18 | 31324934 | 31324934 | A | G | het | . | . | . | 130 | 0 | 0% | A | 140 | 8 | 5.41% | R | Somatic | 0.0058923 |
| exonic | PCDHA1 | nonsynonymous SNV | NM_018900:c.G1327A:p.V443M | 457;Name=lod=96 |  |  |  |  |  | 0 | 0.561449 | 0.996867 | 0.001134 | 0.998929 | chr5 | 140167202 | 140167202 | G | A | het | . | . | . | 533 | 0 | 0% | G | 352 | 4 | 1.12% | R | Somatic | 0.0254557 |
| exonic | PCDHA2 | synonymous SNV | NM_018905:c.C2232T:p.A744A | 468;Name=lod=106 |  |  |  |  |  |  |  |  |  |  | chr5 | 140176781 | 140176781 | C | T | het | . | . | . | 296 | 0 | 0% | C | 202 | 4 | 1.94% | Y | Somatic | 0.0278699 |
| exonic | PCDHA12 | nonsynonymous SNV | NM_018903:c.G1987A:p.A663T | 278;Name=lod=18 |  |  |  |  |  | 0 |  |  |  |  | chr5 | 140257044 | 140257044 | G | A | het | . | . | . | 290 | 0 | 0% | G | 153 | 4 | 2.55% | R | Somatic | 0.014842 |
| exonic | PCDHB10 | synonymous SNV | NM_018930:c.C1269T:p.V423V | 251;Name=lod=14 | 0.95 |  |  |  |  |  |  |  |  |  | chr5 | 140573394 | 140573394 | C | T | het | . | . | . | 491 | 0 | 0% | C | 318 | 4 | 1.24% | Y | Somatic | 0.0243304 |
| exonic | PCDHB13 | synonymous SNV | NM_018933:c.C1500T:p.L500L |  | 0.95 |  |  |  |  |  |  |  |  |  | chr5 | 140595195 | 140595195 | C | T | het | . | . | . | 521 | 0 | 0% | C | 460 | 5 | 1.08% | Y | Somatic | 0.0230633 |
| exonic | PCDHB13 | nonsynonymous SNV | NM_018933:c.G2266A:p.A756T |  | 0.95 |  |  |  |  | 1 | 0 | 0.164426 | 0.006561 | 0.640808 | chr5 | 140595961 | 140595961 | G | A | het | . | . | . | 543 | 1 | 0.18% | G | 274 | 4 | 1.44% | R | Somatic | 0.0472011 |
| exonic | PCDHB15 | synonymous SNV | NM_018935:c.G2202A:p.G734G |  | 0.95 |  |  |  |  |  |  |  |  |  | chr5 | 140627348 | 140627348 | G | A | het | . | . | . | 681 | 0 | 0% | G | 530 | 5 | 0.93% | R | Somatic | 0.0163131 |
| exonic | PCDHGB1 | nonsynonymous SNV | NM_018922:c.C536T:p.T179M |  |  |  |  |  |  |  |  |  |  |  | chr5 | 140730363 | 140730363 | C | T | het | endometrium | carcinoma | endometrioid_carcinoma | 362 | 0 | 0% | C | 207 | 4 | 1.90% | Y | Somatic | 0.0180572 |
| exonic | HMHB1 | nonsynonymous SNV | NM_021182:c.C46T:p.H16Y |  |  | 0.28 | 0.28 | 0.28 | rs161557 |  | 0.22695 | 0.110146 | 0.009686 | 0.610095 | chr5 | 143200053 | 143200053 | C | T | het | . | . | . | 272 | 0 | 0% | C | 138 | 6 | 4.17% | Y | Somatic | 0.0016052 |
| exonic | ADRB2 | nonsynonymous SNV | NM_000024:c.G46A:p.G16R |  |  | 0.44 | 0.44 | 0.44 | rs1042713 | 0.16 | 0 | 0.999523 | 0.284588 | 0.625517 | chr5 | 148206440 | 148206440 | G | A | het | . | . | . | 168 | 0 | 0% | G | 225 | 7 | 3.02% | R | Somatic | 0.0212441 |
| exonic | SH3TC2 | nonsynonymous SNV | NM_024577:c.C86T:p.S29L |  |  |  |  |  |  | 0.51 | 0.002 | 0.975958 | 0.015116 | 0.997732 | chr5 | 148431770 | 148431770 | G | A | het | . | . | . | 304 | 1 | 0.33% | G | 154 | 4 | 2.53% | R | Somatic | 0.0483692 |
| splicing | PDE6A(NM_000440:exon8:c.1065+1G>T) |  |  | 509;Name=lod=156 |  |  |  |  |  |  |  |  |  |  | chr5 | 149286874 | 149286874 | C | A | het | . | . | . | 301 | 0 | 0% | C | 215 | 2 | 0.92% | M | Somatic | 0.1750222 |
| exonic | FAT2 | nonsynonymous SNV | NM_001447:c.T2057C:p.F686S | 251;Name=lod=14 |  | 0.42 | 0.42 | 0.42 | rs9324700 | 0.81 | 0 | 0.972332 | 3.00E-06 | 0.999107 | chr5 | 150946436 | 150946436 | A | G | het | . | . | . | 251 | 0 | 0% | A | 80 | 4 | 4.76% | R | Somatic | 0.0037435 |
| exonic | BIRC8 | nonsynonymous SNV | NM_033341:c.G545A:p.R182H | 440;Name=lod=82 |  |  |  |  | rs138004934 | 0.02 | 0.983 | 0.082408 | 0.463185 | 0.697198 | chr19 | 53793083 | 53793083 | C | T | het | lung | carcinoma | adenocarcinoma | 166 | 0 | 0% | C | 70 | 4 | 5.41% | Y | Somatic | 0.0085351 |
| exonic | CCDC99 | synonymous SNV | NM_017785:c.C963A:p.A321A | 612;Name=lod=410 |  |  |  |  |  |  |  |  |  |  | chr5 | 169023636 | 169023636 | C | A | het | . | . | . | 263 | 0 | 0% | C | 139 | 4 | 2.80% | M | Somatic | 0.0149728 |
| intronic | WDR59 |  |  |  |  | 0.63 | 0.63 | 0.63 | rs4888324 |  |  |  |  |  | chr16 | 74999617 | 74999617 | A | C | het | . | . | . | 132 | 0 | 0% | A | 160 | 9 | 5.33% | M | Somatic | 0.0050375 |
| exonic;splicing | NSD1;NSD1 | nonsynonymous SNV | NM_022455:c.G5894A:p.G1965D | 664;Name=lod=664 |  |  |  |  |  | 0.02 | 0.047 | 0.999763 | 0.999208 | 0.999812 | chr5 | 176709467 | 176709467 | G | A | het | . | . | . | 327 | 0 | 0% | G | 167 | 5 | 2.91% | R | Somatic | 0.0046816 |
| intronic | TRIP12 |  |  |  |  |  |  |  |  |  |  |  |  |  | chr2 | 230638980 | 230638980 | A | T | het | . | . | . | 101 | 0 | 0% | A | 71 | 4 | 5.33% | W | Somatic | 0.0314632 |
| exonic;splicing | PSD3;PSD3 | nonsynonymous SNV | NM_015310:c.G1238A:p.R413K | 289;Name=lod=20 |  |  |  |  |  | 0 | 0.98 | 0.998758 | 0.11887 | 0.99677 | chr8 | 18729136 | 18729136 | C | T | het | . | . | . | 132 | 0 | 0% | C | 71 | 4 | 5.33% | Y | Somatic | 0.0163579 |
| exonic | KIF13A | synonymous SNV | NM_001105566:c.G1677A:p.T559T |  |  | 0.001 | 0.001 | 0.001 | rs79884092 |  |  |  |  |  | chr6 | 17826108 | 17826108 | C | T | het | . | . | . | 291 | 0 | 0% | C | 154 | 5 | 3.14% | Y | Somatic | 0.0052848 |
| exonic | ZSCAN16 | synonymous SNV | NM_025231:c.C96T:p.C32C |  |  |  |  |  |  |  |  |  |  |  | chr6 | 28093317 | 28093317 | C | T | het | . | . | . | 556 | 1 | 0.18% | C | 333 | 6 | 1.77% | Y | Somatic | 0.0135666 |
| exonic | MLNR | nonsynonymous SNV | NM_001507:c.G760A:p.A254T | 584;Name=lod=315 |  |  |  |  |  | 0.15 | 0.017 | 0.997551 | 0.003194 | 0.998228 | chr13 | 49795233 | 49795233 | G | A | het | . | . | . | 167 | 1 | 0.60% | G | 89 | 5 | 5.32% | R | Somatic | 0.0236658 |
| exonic | ITGAX | nonsynonymous SNV | NM_000887:c.G2446A:p.G816R | 387;Name=lod=50 |  |  |  |  |  | 0.75 | 0.925 | 0.825027 | 0.147645 | 0.883317 | chr16 | 31384649 | 31384649 | G | A | het | lung | carcinoma | adenocarcinoma | 81 | 0 | 0% | G | 161 | 9 | 5.29% | R | Somatic | 0.0279538 |
| exonic | SRSF3 | nonsynonymous SNV | NM_003017:c.C413T:p.S138L | 651;Name=lod=589 | 0.96 |  |  |  |  |  | 0.022 | 0.999021 | 0.999706 | 0.999205 | chr6 | 36569517 | 36569517 | C | T | het | . | . | . | 494 | 0 | 0% | C | 199 | 4 | 1.97% | Y | Somatic | 0.0070451 |
| exonic | KIAA0240 | nonsynonymous SNV | NM_015349:c.C2290A:p.L764I | 600;Name=lod=367 |  |  |  |  |  | 0.01 | 0.85 | 0.998732 | 0.416095 | 0.999937 | chr6 | 42828035 | 42828035 | C | A | het | . | . | . | 306 | 0 | 0% | C | 188 | 4 | 2.08% | M | Somatic | 0.0216709 |
| exonic | ZNRF4 | nonsynonymous SNV | NM_181710:c.G436A:p.E146K |  |  |  |  |  | rs182381348 | 0.66 | 0.307 | 0.903179 | 0.324953 | 0.950711 | chr19 | 5455938 | 5455938 | G | A | het | . | . | . | 154 | 0 | 0% | G | 90 | 5 | 5.26% | R | Somatic | 0.0075634 |
| exonic | ENPP4 | nonsynonymous SNV | NM_014936:c.G766A:p.D256N |  |  |  |  |  |  | 0.23 | 0 | 0.032283 | 0.085019 | 0.832504 | chr6 | 46108086 | 46108086 | G | A | het | . | . | . | 225 | 1 | 0.44% | G | 93 | 5 | 5.10% | R | Somatic | 0.010694 |
| exonic | TNFRSF21 | stopgain SNV | NM_014452:c.G1912T:p.E638X | 644;Name=lod=553 |  |  |  |  |  | 0 | 0.735426 | 0.999231 | 0.999976 | 0.999999 | chr6 | 47200557 | 47200557 | C | A | het | . | . | . | 352 | 0 | 0% | C | 195 | 4 | 2.01% | M | Somatic | 0.0166868 |
| exonic | GSTA5 | nonsynonymous SNV | NM_153699:c.A559G:p.R187G | 472;Name=lod=111 |  |  |  |  |  | 0 | 1 | 0.804129 | 0.99304 | 0.999982 | chr6 | 52696756 | 52696756 | T | C | het | . | . | . | 224 | 0 | 0% | T | 175 | 5 | 2.78% | Y | Somatic | 0.0170182 |
| intronic | GSTA3 |  |  |  |  |  |  |  |  |  |  |  |  |  | chr6 | 52767140 | 52767140 | G | A | het | . | . | . | 509 | 1 | 0.20% | G | 218 | 5 | 2.24% | R | Somatic | 0.0113713 |
| intronic | DST |  |  |  |  |  |  |  |  | 0.08 |  |  |  |  | chr6 | 56473886 | 56473886 | G | A | het | . | . | . | 621 | 0 | 0% | G | 188 | 5 | 2.59% | R | Somatic | 7.20E-04 |
| exonic | DST | synonymous SNV | NM_001723:c.G6108A:p.K2036K |  |  |  |  |  |  |  |  |  |  |  | chr6 | 56482157 | 56482157 | C | T | het | . | . | . | 668 | 0 | 0% | C | 233 | 4 | 1.69% | Y | Somatic | 0.0046156 |
| intronic | FGFR3 |  |  | 533;Name=lod=195 |  |  |  |  |  |  |  |  |  |  | chr4 | 1808058 | 1808058 | C | T | het | . | . | . | 97 | 0 | 0% | C | 72 | 4 | 5.26% | Y | Somatic | 0.0355967 |
| exonic | IMPG1 | nonsynonymous SNV | NM_001563:c.G214A:p.A72T |  |  |  |  |  |  | 0.53 | 0 | 0.170596 | 0.035272 | 0.621806 | chr6 | 76751697 | 76751697 | C | T | het | . | . | . | 380 | 2 | 0.52% | C | 110 | 4 | 3.51% | Y | Somatic | 0.0271327 |
| intronic | TBX18 |  |  |  |  |  |  |  |  |  |  |  |  |  | chr6 | 85472245 | 85472245 | G | A | het | . | . | . | 467 | 1 | 0.21% | G | 221 | 5 | 2.21% | R | Somatic | 0.0156225 |
| intronic | ORC3 |  |  | 385;Name=lod=49 |  | 0.33 | 0.33 | 0.33 | rs1075665 |  |  |  |  |  | chr6 | 88366602 | 88366602 | A | G | het | . | . | . | 217 | 0 | 0% | A | 123 | 5 | 3.91% | R | Somatic | 0.0066875 |
| exonic | MDN1 | nonsynonymous SNV | NM_014611:c.G15694A:p.V5232I |  |  |  |  |  |  | 0.22 | 0.354064 | 0.780229 | 3.77E-04 | 0.742643 | chr6 | 90362842 | 90362842 | C | T | het | . | . | . | 421 | 1 | 0.24% | C | 162 | 4 | 2.41% | Y | Somatic | 0.0240741 |
| exonic | MANEA | nonsynonymous SNV | NM_024641:c.G688A:p.D230N | 589;Name=lod=331 |  |  |  |  |  | 0.39 | 0 | 0.993632 | 0.945762 | 1 | chr6 | 96052736 | 96052736 | G | A | het | . | . | . | 125 | 0 | 0% | G | 48 | 4 | 7.69% | R | Somatic | 0.0068496 |
| exonic | UFL1 | nonsynonymous SNV | NM_015323:c.C685T:p.R229C | 539;Name=lod=207 |  |  |  |  |  | 0 | 1 | 0.99877 | 0.999698 | 1 | chr6 | 96984149 | 96984149 | C | T | het | . | . | . | 471 | 0 | 0% | C | 164 | 5 | 2.96% | Y | Somatic | 0.0012286 |
| exonic | FHL5 | nonsynonymous SNV | NM_001170807:c.A610G:p.R204G | 439;Name=lod=81 |  | 0.42 | 0.42 | 0.42 | rs2273621 | 0 | 0.954 | 0.981846 | 0.119968 | 0.996649 | chr6 | 97058553 | 97058553 | A | G | het | . | . | . | 426 | 0 | 0% | A | 113 | 4 | 3.42% | R | Somatic | 0.0020695 |
| exonic | CEP57L1 | nonsynonymous SNV | NM_173830:c.C1311A:p.S437R |  |  |  |  |  |  | 0.61 | 0.001 | 0.802771 | 0.351197 | 0.883572 | chr6 | 109484101 | 109484101 | C | A | het | . | . | . | 301 | 0 | 0% | C | 114 | 4 | 3.39% | M | Somatic | 0.0060618 |
| exonic | REV3L | synonymous SNV | NM_002912:c.G4290A:p.V1430V | 541;Name=lod=210 |  | 0.35 | 0.35 | 0.35 | rs455732 |  |  |  |  |  | chr6 | 111695268 | 111695268 | C | T | het | . | . | . | 557 | 1 | 0.18% | C | 181 | 6 | 3.21% | Y | Somatic | 0.0013015 |
| exonic | REV3L | nonsynonymous SNV | NM_002912:c.G3503T:p.S1168I | 371;Name=lod=43 |  |  |  |  |  | 0 | 0.293 | 0.985004 | 0.97313 | 0.99953 | chr6 | 111696055 | 111696055 | C | A | het | . | . | . | 404 | 1 | 0.25% | C | 153 | 4 | 2.55% | M | Somatic | 0.0231207 |
| exonic | REV3L | synonymous SNV | NM_002912:c.A2706G:p.G902G | 557;Name=lod=245 |  | 0.36 | 0.36 | 0.36 | rs458486 |  |  |  |  |  | chr6 | 111696852 | 111696852 | T | C | het | . | . | . | 327 | 0 | 0% | T | 107 | 4 | 3.60% | Y | Somatic | 0.0039594 |
| exonic | MET | synonymous SNV | NM_000245:c.C2214T:p.Y738Y | 376;Name=lod=45 |  |  |  |  |  |  |  |  |  |  | chr7 | 116398624 | 116398624 | C | T | het | . | . | . | 204 | 0 | 0% | C | 90 | 5 | 5.26% | Y | Somatic | 0.0030089 |
| exonic;splicing | C6orf170;C6orf170 | nonsynonymous SNV | NM_152730:c.A3464G:p.Q1155R | 601;Name=lod=369 |  |  |  |  |  | 0 | 0.497 | 0.998046 | 0.841869 | 1 | chr6 | 121427170 | 121427170 | T | C | het | . | . | . | 545 | 1 | 0.18% | T | 251 | 4 | 1.57% | Y | Somatic | 0.037803 |
| exonic | MOXD1 | nonsynonymous SNV | NM_015529:c.G1177A:p.A393T | 615;Name=lod=420 |  |  |  |  |  | 0.27 | 0.015 | 0.98329 | 0.949378 | 0.999999 | chr6 | 132643946 | 132643946 | C | T | het | . | . | . | 104 | 0 | 0% | C | 47 | 4 | 7.84% | Y | Somatic | 0.0108041 |
| intronic | EYA4 |  |  | 564;Name=lod=262 |  |  |  |  |  |  |  |  |  |  | chr6 | 133703523 | 133703523 | C | T | het | . | . | . | 281 | 0 | 0% | C | 172 | 4 | 2.27% | Y | Somatic | 0.0215376 |
| exonic | SGK1 | nonsynonymous SNV | NM_001143677:c.T856C:p.C286R | 454;Name=lod=93 |  |  |  |  |  | 0 | 0.999 | 0.99917 | 1 | 1 | chr6 | 134493345 | 134493345 | A | G | het | lung | carcinoma | small_cell_carcinoma | 275 | 0 | 0% | A | 239 | 4 | 1.65% | R | Somatic | 0.0477939 |
| exonic | HECA | nonsynonymous SNV | NM_016217:c.C284T:p.A95V | 537;Name=lod=203 |  |  |  |  |  | 0.04 | 0.843 | 0.999016 | 0.99924 | 1 | chr6 | 139487433 | 139487433 | C | T | het | . | . | . | 228 | 0 | 0% | C | 246 | 7 | 2.77% | Y | Somatic | 0.0107032 |
| intronic | LTV1 |  |  |  |  | 0.42 | 0.42 | 0.42 | rs3818089 |  |  |  |  |  | chr6 | 144178419 | 144178419 | T | C | het | . | . | . | 343 | 0 | 0% | T | 181 | 4 | 2.16% | Y | Somatic | 0.0147544 |
| intronic | NFE2L1 |  |  |  |  |  |  |  |  |  |  |  |  |  | chr17 | 46134672 | 46134672 | T | C | het | . | . | . | 162 | 0 | 0% | T | 91 | 5 | 5.21% | Y | Somatic | 0.0066716 |
| ncRNA_exonic | SNORA29 |  |  | 478;Name=lod=117 |  | 0.22 | 0.22 | 0.22 | rs3818298 |  |  |  |  |  | chr6 | 160206716 | 160206716 | T | C | het | . | . | . | 419 | 1 | 0.24% | T | 207 | 5 | 2.36% | Y | Somatic | 0.0178965 |
| exonic | TMEM106B | nonsynonymous SNV | NM_001134232:c.G151T:p.V51L | 619;Name=lod=439 |  |  |  |  |  | 0 | 0.984 | 0.99969 | 0.978676 | 1 | chr7 | 12254587 | 12254587 | G | T | het | . | . | . | 280 | 0 | 0% | G | 124 | 4 | 3.12% | K | Somatic | 0.0093769 |
| exonic | SCEL | nonsynonymous SNV | NM_003843:c.C518T:p.A173V |  |  |  |  |  | rs143177767 | 0.01 | 0.061 | 0.226449 | 0.129644 | 0.778303 | chr13 | 78146297 | 78146297 | C | T | het | . | . | . | 133 | 0 | 0% | C | 73 | 4 | 5.19% | Y | Somatic | 0.0171869 |
| exonic | CDCA7L | synonymous SNV | NM_018719:c.G264A:p.T88T |  |  |  |  |  |  |  |  |  |  |  | chr7 | 21951274 | 21951274 | C | T | het | . | . | . | 230 | 0 | 0% | C | 129 | 4 | 3.01% | Y | Somatic | 0.0175071 |
| exonic | MIA2 | synonymous SNV | NM_054024:c.G261A:p.E87E | 574;Name=lod=288 |  |  |  |  |  |  |  |  |  |  | chr14 | 39709776 | 39709776 | G | A | het | . | . | . | 151 | 0 | 0% | G | 73 | 4 | 5.19% | R | Somatic | 0.0123409 |
| intronic | TARP |  |  |  | 0.95 | 0.47 | 0.47 | 0.47 | rs2392542 |  |  |  |  |  | chr7 | 38301741 | 38301741 | T | A | het | . | . | . | 291 | 0 | 0% | T | 140 | 4 | 2.78% | W | Somatic | 0.011675 |
| exonic | MATR3 | synonymous SNV | NM_001194955:c.C330T:p.N110N | 909;Name=lod=6572 | 0.95 |  |  |  |  |  |  |  |  |  | chr5 | 138643434 | 138643434 | C | T | het | . | . | . | 154 | 0 | 0% | C | 73 | 4 | 5.19% | Y | Somatic | 0.0117081 |
| exonic | NGLY1 | synonymous SNV | NM_001145293:c.G597A:p.P199P | 470;Name=lod=108 |  |  |  |  |  |  |  |  |  |  | chr3 | 25792650 | 25792650 | C | T | het | . | . | . | 163 | 0 | 0% | C | 74 | 4 | 5.13% | Y | Somatic | 0.0104054 |
| intronic | CDK14 |  |  |  |  |  |  |  |  |  |  |  |  |  | chr7 | 90741844 | 90741844 | C | T | het | . | . | . | 456 | 0 | 0% | C | 303 | 5 | 1.62% | Y | Somatic | 0.0104427 |
| exonic | FZD1 | synonymous SNV | NM_003505:c.G1593A:p.E531E | 610;Name=lod=402 |  |  |  |  |  |  |  |  |  |  | chr7 | 90895788 | 90895788 | G | A | het | . | . | . | 364 | 0 | 0% | G | 305 | 6 | 1.93% | R | Somatic | 0.0093185 |
| exonic | WFDC3 | synonymous SNV | NM_080614:c.T51C:p.S17S | 374;Name=lod=44 |  | 0.63 | 0.63 | 0.63 | rs3746493 |  |  |  |  |  | chr20 | 44418564 | 44418564 | A | G | het | . | . | . | 99 | 0 | 0% | A | 75 | 4 | 5.06% | R | Somatic | 0.0371605 |
| exonic | SAMD9 | nonsynonymous SNV | NM_001193307:c.A308G:p.Q103R |  |  |  |  |  |  | 0.69 | 0.08 | 0.994543 | 0.001669 | 0.920937 | chr7 | 92735103 | 92735103 | T | C | het | . | . | . | 410 | 0 | 0% | T | 151 | 4 | 2.58% | Y | Somatic | 0.0055057 |
| exonic | SWT1 | synonymous SNV | NM_001105518:c.A126G:p.S42S |  |  | 0.34 | 0.34 | 0.34 | rs950327 |  |  |  |  |  | chr1 | 185135745 | 185135745 | A | G | het | . | . | . | 204 | 0 | 0% | A | 113 | 6 | 5.04% | R | Somatic | 0.0023054 |
| exonic | TRIM4 | synonymous SNV | NM_033017:c.G465A:p.K155K |  |  |  |  |  |  |  |  |  |  |  | chr7 | 99514331 | 99514331 | C | T | het | . | . | . | 579 | 0 | 0% | C | 319 | 4 | 1.24% | Y | Somatic | 0.0162473 |
| UTR5 | ZSCAN21 |  |  |  |  | 0.28 | 0.28 | 0.28 | rs11558475 |  |  |  |  |  | chr7 | 99654600 | 99654600 | A | G | het | . | . | . | 384 | 0 | 0% | A | 301 | 5 | 1.63% | R | Somatic | 0.0168425 |
| exonic | MEPCE | nonsynonymous SNV | NM_019606:c.G661A:p.A221T | 677;Name=lod=749 |  |  |  |  |  | 0.58 | 0.834 | 0.999438 | 0.949507 | 0.999998 | chr7 | 100028302 | 100028302 | G | A | het | . | . | . | 410 | 1 | 0.24% | G | 258 | 5 | 1.90% | R | Somatic | 0.0359841 |
| intergenic | ACHE(dist=56618),MUC12(dist=62745) |  |  |  |  |  |  |  |  | 0.41 |  |  |  |  | chr7 | 100550159 | 100550159 | C | T | het | . | . | . | 347 | 0 | 0% | C | 218 | 3 | 1.36% | Y | Somatic | 0.0584132 |
| exonic | MUC17 | nonsynonymous SNV | NM_001040105:c.C2920T:p.P974S |  | 0.9 |  |  |  |  |  | 0.209027 | 0.037151 | 0.002605 | 0.588427 | chr7 | 100677617 | 100677617 | C | T | het | . | . | . | 749 | 1 | 0.13% | C | 382 | 5 | 1.29% | Y | Somatic | 0.019367 |
| exonic | MUC17 | nonsynonymous SNV | NM_001040105:c.C9155T:p.P3052L |  |  |  |  |  |  |  | 0.995 | 0.039716 | 1.77E-04 | 0.622578 | chr7 | 100683852 | 100683852 | C | T | het | . | . | . | 777 | 0 | 0% | C | 336 | 7 | 2.04% | Y | Somatic | 2.42E-04 |
| exonic | DNAJC2 | stopgain SNV | NM_001129887:c.C1069T:p.R357X | 627;Name=lod=472 |  |  |  |  |  | 1 | 0.735491 | 0.999583 | 1 | 0.999999 | chr7 | 102962393 | 102962393 | G | A | het | . | . | . | 308 | 0 | 0% | G | 248 | 3 | 1.20% | R | Somatic | 0.0899315 |
| exonic | RELN | synonymous SNV | NM_005045:c.G492A:p.R164R | 628;Name=lod=474 |  |  |  |  |  |  |  |  |  |  | chr7 | 103417056 | 103417056 | C | T | het | . | . | . | 236 | 0 | 0% | C | 89 | 4 | 4.30% | Y | Somatic | 0.0060914 |
| exonic | CDHR3 | nonsynonymous SNV | NM_152750:c.G183C:p.Q61H |  |  | 0.16 | 0.16 | 0.16 | rs34426483 | 0.02 |  |  |  |  | chr7 | 105615426 | 105615426 | G | C | het | . | . | . | 267 | 0 | 0% | G | 120 | 5 | 4% | S | Somatic | 0.0031194 |
| exonic | CBLL1 | synonymous SNV | NM_024814:c.T1071C:p.H357H | 695;Name=lod=889 |  |  |  |  |  |  |  |  |  |  | chr7 | 107399218 | 107399218 | T | C | het | . | . | . | 353 | 1 | 0.28% | T | 190 | 7 | 3.55% | Y | Somatic | 0.0038699 |
| exonic | C7orf53 | nonsynonymous SNV | NM_001134468:c.G356A:p.R119Q | 300;Name=lod=22 |  |  |  |  |  | 0.14 | 0.922 | 0.901152 | 0.038786 | 0.929043 | chr7 | 112129964 | 112129964 | G | A | het | . | . | . | 337 | 1 | 0.30% | G | 159 | 4 | 2.45% | R | Somatic | 0.0406529 |
| exonic | PABPC4 | nonsynonymous SNV | NM_001135653:c.C1258T:p.P420S | 489;Name=lod=130 |  |  |  |  |  | 0.07 | 0 | 0.973699 | 0.790303 | 1 | chr1 | 40030433 | 40030433 | G | A | het | . | . | . | 107 | 0 | 0% | G | 76 | 4 | 5% | R | Somatic | 0.0320596 |
| exonic | FAM208A | nonsynonymous SNV | NM_015224:c.A2992G:p.I998V | 577;Name=lod=294 |  | 0.2 | 0.2 | 0.2 | rs2291498 | 0.52 | 0.434556 | 0.970962 | 8.63E-04 | 0.999649 | chr3 | 56658871 | 56658871 | T | C | het | . | . | . | 156 | 0 | 0% | T | 95 | 5 | 5% | Y | Somatic | 0.0085461 |
| exonic | HLA-DQA1 | synonymous SNV | NM_002122:c.C90T:p.H30H |  |  | 0.28 | 0.28 | 0.28 | rs1129737 |  |  |  |  |  | chr6 | 32609094 | 32609094 | C | T | het | . | . | . | 149 | 0 | 0% | C | 76 | 4 | 5% | Y | Somatic | 0.0141709 |
| intronic | CHCHD3 |  |  |  |  | 0.35 | 0.35 | 0.35 | rs13235276 |  |  |  |  |  | chr7 | 132709293 | 132709293 | C | T | het | . | . | . | 517 | 0 | 0% | C | 230 | 7 | 2.95% | Y | Somatic | 2.85E-04 |
| exonic | DGKI | nonsynonymous SNV | NM_004717:c.C632T:p.A211V | 578;Name=lod=298 |  |  |  |  |  | 0.01 | 0.47 | 0.987726 | 0.985629 | 1 | chr7 | 137341261 | 137341261 | G | A | het | . | . | . | 316 | 0 | 0% | G | 175 | 4 | 2.23% | R | Somatic | 0.0167346 |
| splicing | DYNLT3(NM_006520:exon5:c.274+1G>A) |  |  | 605;Name=lod=384 |  |  |  |  |  |  |  |  |  |  | chrX | 37700280 | 37700280 | C | T | het | . | . | . | 146 | 0 | 0% | C | 77 | 4 | 4.94% | Y | Somatic | 0.015443 |
| intronic | HIPK2 |  |  |  |  |  |  |  |  |  |  |  |  |  | chr7 | 139305318 | 139305318 | G | A | het | . | . | . | 305 | 0 | 0% | G | 323 | 4 | 1.22% | R | Somatic | 0.0710323 |
| exonic | ZNF778 | synonymous SNV | NM_182531:c.C1635T:p.S545S |  |  |  |  |  |  |  |  |  |  |  | chr16 | 89294415 | 89294415 | C | T | het | . | . | . | 170 | 0 | 0% | C | 97 | 5 | 4.90% | Y | Somatic | 0.0069662 |
| exonic | NOS3 | nonsynonymous SNV | NM_001160109:c.G1453A:p.A485T |  |  |  |  |  |  | 0.63 | 0 | 0.15921 | 0.13104 | 0.501626 | chr7 | 150698656 | 150698656 | G | A | het | . | . | . | 433 | 0 | 0% | G | 572 | 6 | 1.04% | R | Somatic | 0.034531 |
| exonic | ABCB8 | nonsynonymous SNV | NM_007188:c.G1358A:p.G453D | 289;Name=lod=20 |  |  |  |  |  | 0.02 | 0.992 | 0.999252 | 0.99988 | 1 | chr7 | 150737691 | 150737691 | G | A | het | . | . | . | 233 | 0 | 0% | G | 356 | 7 | 1.93% | R | Somatic | 0.0303882 |
| exonic | HEATR3 | nonsynonymous SNV | NM_182922:c.C1082T:p.T361I | 340;Name=lod=32 |  |  |  |  |  | 0.05 | 0.028 | 0.999227 | 0.163031 | 0.99784 | chr16 | 50117888 | 50117888 | C | T | het | . | . | . | 159 | 1 | 0.62% | C | 78 | 4 | 4.88% | Y | Somatic | 0.0462905 |
| exonic | GATA4 | synonymous SNV | NM_002052:c.C633T:p.F211F | 366;Name=lod=41 |  |  |  |  |  |  |  |  |  |  | chr8 | 11606444 | 11606444 | C | T | het | . | . | . | 277 | 0 | 0% | C | 267 | 4 | 1.48% | Y | Somatic | 0.0591375 |
| exonic | PTPDC1 | synonymous SNV | NM_152422:c.G783A:p.T261T | 625;Name=lod=461 |  |  |  |  | rs137947987 |  |  |  |  |  | chr9 | 96859637 | 96859637 | G | A | het | . | . | . | 207 | 0 | 0% | G | 98 | 5 | 4.85% | R | Somatic | 0.0037902 |
| exonic | CSGALNACT1 | synonymous SNV | NM_001130518:c.C963T:p.N321N | 558;Name=lod=246 |  |  |  |  |  |  |  |  |  |  | chr8 | 19278020 | 19278020 | G | A | het | . | . | . | 217 | 0 | 0% | G | 137 | 4 | 2.84% | R | Somatic | 0.0234429 |
| exonic | PROSC | synonymous SNV | NM_007198:c.C267A:p.I89I | 589;Name=lod=329 |  |  |  |  |  |  |  |  |  |  | chr8 | 37623821 | 37623821 | C | A | het | . | . | . | 303 | 0 | 0% | C | 203 | 5 | 2.40% | M | Somatic | 0.0108569 |
| exonic | TACC1 | synonymous SNV | NM_001122824:c.G666A:p.R222R | 586;Name=lod=322 |  |  |  |  |  |  |  |  |  |  | chr8 | 38696044 | 38696044 | G | A | het | . | . | . | 315 | 0 | 0% | G | 245 | 6 | 2.39% | R | Somatic | 0.0073544 |
| exonic | POTEA | nonsynonymous SNV | NM_001002920:c.C614T:p.A205V |  |  |  |  |  |  | 0 | 0.508 | 0.844056 | 0.014332 | 0.99837 | chr8 | 43155686 | 43155686 | C | T | het | . | . | . | 219 | 0 | 0% | C | 135 | 4 | 2.88% | Y | Somatic | 0.0221271 |
| exonic | ITGA1 | nonsynonymous SNV | NM_181501:c.C3452T:p.A1151V | 451;Name=lod=91 |  |  |  |  |  | 0.05 | 0.092 | 0.998986 | 0.480289 | 0.999987 | chr5 | 52243248 | 52243248 | C | T | het | . | . | . | 212 | 0 | 0% | C | 79 | 4 | 4.82% | Y | Somatic | 0.0059436 |
| exonic | DPP4 | synonymous SNV | NM_001935:c.A2118G:p.T706T | 786;Name=lod=2089 |  |  |  |  |  |  |  |  |  |  | chr2 | 162851817 | 162851817 | T | C | het | . | . | . | 190 | 0 | 0% | T | 99 | 5 | 4.81% | Y | Somatic | 0.0051988 |
| intronic | GGH |  |  |  |  | 0.25 | 0.25 | 0.25 | rs4617146 |  |  |  |  |  | chr8 | 63942717 | 63942717 | C | T | het | . | . | . | 270 | 0 | 0% | C | 139 | 5 | 3.47% | Y | Somatic | 0.0048625 |
| intronic | CAST |  |  |  |  | 0.4 | 0.4 | 0.4 | rs27654 |  |  |  |  |  | chr5 | 96093222 | 96093222 | G | A | het | . | . | . | 211 | 0 | 0% | G | 100 | 5 | 4.76% | R | Somatic | 0.0037963 |
| exonic | INTS8 | synonymous SNV | NM_017864:c.G1794A:p.E598E | 640;Name=lod=532 |  |  |  |  | rs150183548 |  |  |  |  |  | chr8 | 95869046 | 95869046 | G | A | het | . | . | . | 315 | 0 | 0% | G | 216 | 4 | 1.82% | R | Somatic | 0.0281351 |
| exonic | PGCP | nonsynonymous SNV | NM_016134:c.C716T:p.A239V | 510;Name=lod=158 |  |  |  |  |  | 0 | 0.955 | 0.999155 | 0.702288 | 1 | chr8 | 97892100 | 97892100 | C | T | het | . | . | . | 517 | 1 | 0.19% | C | 218 | 5 | 2.24% | Y | Somatic | 0.0108079 |
| exonic | GULP1 | nonsynonymous SNV | NM_016315:c.C485A:p.T162K | 694;Name=lod=884 |  |  |  |  |  | 0.03 | 0.977 | 0.999287 | 0.999966 | 1 | chr2 | 189434051 | 189434051 | C | A | het | . | . | . | 193 | 0 | 0% | C | 101 | 5 | 4.72% | M | Somatic | 0.0052627 |
| exonic | KLF10 | synonymous SNV | NM_001032282:c.G1143A:p.T381T | 586;Name=lod=322 |  |  |  |  |  |  |  |  |  |  | chr8 | 103663384 | 103663384 | C | T | het | large_intestine | carcinoma | adenocarcinoma | 254 | 1 | 0.39% | C | 164 | 5 | 2.96% | Y | Somatic | 0.0392332 |
| exonic | CTHRC1 | synonymous SNV | NM_138455:c.T648C:p.G216G |  |  | 0.72 | 0.72 | 0.72 | rs3098233 |  |  |  |  |  | chr8 | 104394744 | 104394744 | T | C | het | . | . | . | 512 | 1 | 0.19% | T | 186 | 8 | 4.12% | Y | Somatic | 1.99E-04 |
| exonic | RIMS2 | nonsynonymous SNV | NM_014677:c.G2336A:p.R779H | 485;Name=lod=125 |  |  |  |  |  | 0 | 0.192 | 0.991006 | 0 | 0.837418 | chr8 | 105001565 | 105001565 | G | A | het | . | . | . | 329 | 1 | 0.30% | G | 139 | 5 | 3.47% | R | Somatic | 0.0111325 |
| exonic | EIF3E | nonsynonymous SNV | NM_001568:c.T649G:p.W217G | 695;Name=lod=893 |  |  |  |  |  | 0 | 0.998 | 0.998955 | 0.999999 | 1 | chr8 | 109240569 | 109240569 | A | C | het | . | . | . | 366 | 0 | 0% | A | 188 | 4 | 2.08% | M | Somatic | 0.0137307 |
| exonic | CSMD3 | nonsynonymous SNV | NM_052900:c.G8819T:p.C2940F | 606;Name=lod=386 |  |  |  |  |  | 0 | 1 | 0.998758 | 0.999981 | 1 | chr8 | 113299298 | 113299298 | C | A | het | . | . | . | 269 | 1 | 0.37% | C | 77 | 4 | 4.94% | M | Somatic | 0.0110055 |
| intronic | FER1L6 |  |  |  |  |  |  |  |  |  |  |  |  |  | chr8 | 125078653 | 125078653 | C | T | het | . | . | . | 770 | 1 | 0.13% | C | 295 | 4 | 1.34% | Y | Somatic | 0.0233953 |
| exonic | NDUFB9 | nonsynonymous SNV | NM_005005:c.G404A:p.R135Q |  |  |  |  |  |  | 0.27 | 0.01 | 0.999159 | 0.696141 | 0.999185 | chr8 | 125559350 | 125559350 | G | A | het | . | . | . | 278 | 1 | 0.36% | G | 132 | 4 | 2.94% | R | Somatic | 0.0415844 |
| exonic | KIAA0196 | nonsynonymous SNV | NM_014846:c.G572A:p.R191Q | 709;Name=lod=1017 |  |  |  |  |  | 0.03 | 0.998 | 0.978424 | 0.999883 | 1 | chr8 | 126091119 | 126091119 | C | T | het | ovary | carcinoma | serous_carcinoma | 289 | 1 | 0.34% | C | 139 | 5 | 3.47% | Y | Somatic | 0.0168034 |
| exonic | TG | synonymous SNV | NM_003235:c.C6228T:p.C2076C | 329;Name=lod=29 |  |  |  |  |  |  |  |  |  |  | chr8 | 133995623 | 133995623 | C | T | het | . | . | . | 488 | 1 | 0.20% | C | 226 | 4 | 1.74% | Y | Somatic | 0.0384243 |
| exonic | KHDRBS3 | nonsynonymous SNV | NM_006558:c.G838A:p.D280N | 615;Name=lod=423 |  |  |  |  |  | 0.09 | 0.993 | 0.999783 | 0.866577 | 1 | chr8 | 136619228 | 136619228 | G | A | het | . | . | . | 401 | 0 | 0% | G | 169 | 2 | 1.17% | R | Somatic | 0.0890047 |
| exonic | NDOR1 | nonsynonymous SNV | NM_001144027:c.C715T:p.L239F |  |  |  |  |  |  | 0.7 | 0 | 0.004666 | 0.002693 | 0.027146 | chr9 | 140109116 | 140109116 | C | T | het | . | . | . | 102 | 0 | 0% | C | 81 | 4 | 4.71% | Y | Somatic | 0.0410437 |
| exonic | COL22A1 | nonsynonymous SNV | NM_152888:c.A1586G:p.K529R | 397;Name=lod=55 |  |  |  |  |  | 0.09 | 0.215868 | 0.996809 | 0.196383 | 0.999855 | chr8 | 139809072 | 139809072 | T | C | het | . | . | . | 563 | 1 | 0.18% | T | 249 | 4 | 1.58% | Y | Somatic | 0.0341466 |
| intronic | SCRIB |  |  | 381;Name=lod=47 |  |  |  |  |  |  |  |  |  |  | chr8 | 144891716 | 144891716 | C | T | het | . | . | . | 226 | 0 | 0% | C | 181 | 4 | 2.16% | Y | Somatic | 0.0403184 |
| exonic | EPPK1 | nonsynonymous SNV | NM_031308:c.G7096A:p.G2366S | 711;Name=lod=1036 |  |  |  |  |  | 0 |  |  |  |  | chr8 | 144940326 | 144940326 | C | T | het | . | . | . | 1565 | 1 | 0.06% | C | 1132 | 8 | 0.70% | Y | Somatic | 0.0055204 |
| exonic | MCOLN3 | nonsynonymous SNV | NM_018298:c.G1370A:p.G457E | 716;Name=lod=1086 |  |  |  |  |  | 0 | 0.995 | 0.999157 | 0.994769 | 1 | chr1 | 85486910 | 85486910 | C | T | het | . | . | . | 174 | 0 | 0% | C | 102 | 5 | 4.67% | Y | Somatic | 0.0075467 |
| exonic | CPSF1 | synonymous SNV | NM_013291:c.C1734T:p.S578S | 511;Name=lod=159 |  |  |  |  |  |  |  |  |  |  | chr8 | 145623933 | 145623933 | G | A | het | . | . | . | 418 | 0 | 0% | G | 316 | 4 | 1.25% | R | Somatic | 0.0349734 |
| intronic | AK3 |  |  |  |  | 0.47 | 0.47 | 0.47 | rs10758645 |  |  |  |  |  | chr9 | 4722685 | 4722685 | C | G | het | . | . | . | 69 | 2 | 2.82% | C | 35 | 8 | 18.60% | S | Somatic | 0.0059111 |
| exonic | CNTLN | synonymous SNV | NM_017738:c.G3873A:p.L1291L | 420;Name=lod=68 |  |  |  |  |  |  |  |  |  |  | chr9 | 17484310 | 17484310 | G | A | het | . | . | . | 481 | 1 | 0.21% | G | 201 | 5 | 2.43% | R | Somatic | 0.0105303 |
| exonic | MLLT3 | synonymous SNV | NM_004529:c.G735A:p.E245E | 771;Name=lod=1817 |  |  |  |  |  |  |  |  |  |  | chr9 | 20414109 | 20414109 | C | T | het | . | . | . | 826 | 1 | 0.12% | C | 272 | 8 | 2.86% | Y | Somatic | 1.09E-04 |
| exonic | MLLT3 | synonymous SNV | NM_004529:c.T717C:p.N239N | 771;Name=lod=1817 |  |  |  |  |  |  |  |  |  |  | chr9 | 20414127 | 20414127 | A | G | het | . | . | . | 832 | 3 | 0.36% | A | 290 | 9 | 3.01% | R | Somatic | 5.62E-04 |
| exonic | MLLT3 | nonsynonymous SNV | NM_004529:c.A693C:p.E231D | 771;Name=lod=1817 |  |  |  |  |  | 0.86 | 0.009 | 0.963611 | 0.757945 | 1 | chr9 | 20414151 | 20414151 | T | G | het | . | . | . | 872 | 0 | 0% | T | 319 | 7 | 2.15% | K | Somatic | 1.05E-04 |
| exonic | NBAS | synonymous SNV | NM_015909:c.G2223A:p.L741L | 523;Name=lod=178 |  | 0.52 | 0.52 | 0.52 | rs7590340 |  |  |  |  |  | chr2 | 15601441 | 15601441 | C | T | het | . | . | . | 210 | 0 | 0% | C | 102 | 5 | 4.67% | Y | Somatic | 0.0041136 |
| exonic | TRPM3 | synonymous SNV | NM_206944:c.C2373T:p.V791V |  |  |  |  |  | rs145029936 |  |  |  |  |  | chr9 | 73213485 | 73213485 | G | A | het | large_intestine | carcinoma | adenocarcinoma | 247 | 0 | 0% | G | 131 | 4 | 2.96% | R | Somatic | 0.0151514 |
| exonic;splicing | ALDH1A1;ALDH1A1 | nonsynonymous SNV | NM_000689:c.C311T:p.A104V | 329;Name=lod=29 |  |  |  |  |  | 0 | 0.765 | 0.999606 | 0.99286 | 0.99971 | chr9 | 75545796 | 75545796 | G | A | het | . | . | . | 282 | 1 | 0.35% | G | 110 | 5 | 4.35% | R | Somatic | 0.0087009 |
| exonic | ANXA1 | synonymous SNV | NM_000700:c.A327G:p.L109L | 508;Name=lod=155 |  | 0.14 | 0.14 | 0.14 | rs1050305 |  |  |  |  |  | chr9 | 75775235 | 75775235 | A | G | het | . | . | . | 346 | 1 | 0.29% | A | 118 | 4 | 3.28% | R | Somatic | 0.0175863 |
| exonic | CCDC104 | nonsynonymous SNV | NM_080667:c.C820T:p.H274Y | 439;Name=lod=81 |  |  |  |  |  | 0.04 | 0.949 | 0.21329 | 0.01509 | 0.999257 | chr2 | 55771398 | 55771398 | C | T | het | . | . | . | 158 | 0 | 0% | C | 102 | 5 | 4.67% | Y | Somatic | 0.0101392 |
| intronic | FMN2 |  |  |  |  |  |  |  |  |  |  |  |  |  | chr1 | 240341373 | 240341373 | C | T | het | . | . | . | 114 | 0 | 0% | C | 82 | 4 | 4.65% | Y | Somatic | 0.0328292 |
| intronic | NOL8 |  |  |  |  |  |  |  |  |  |  |  |  |  | chr9 | 95069139 | 95069139 | G | T | het | . | . | . | 349 | 0 | 0% | G | 151 | 3 | 1.95% | K | Somatic | 0.0283104 |
| exonic | ECM2 | nonsynonymous SNV | NM_001197295:c.G580A:p.D194N | 295;Name=lod=21 |  |  |  |  |  | 0.58 | 0.002 | 0.870291 | 0.054068 | 0.292739 | chr9 | 95277321 | 95277321 | C | T | het | . | . | . | 360 | 0 | 0% | C | 252 | 6 | 2.33% | Y | Somatic | 0.005116 |
| exonic | DDX4 | synonymous SNV | NM_001166534:c.G72A:p.R24R | 520;Name=lod=173 |  |  |  |  |  |  |  |  |  |  | chr5 | 55063743 | 55063743 | G | A | het | . | . | . | 149 | 0 | 0% | G | 82 | 4 | 4.65% | R | Somatic | 0.0171454 |
| ncRNA_exonic | LOC100507346 |  |  |  |  | 0.1 | 0.1 | 0.1 | rs16909898 |  |  |  |  |  | chr9 | 98231008 | 98231008 | A | G | het | skin | carcinoma | basal_cell_carcinoma | 379 | 0 | 0% | A | 320 | 4 | 1.23% | R | Somatic | 0.0446684 |
| intronic | HSD17B3 |  |  |  |  |  |  |  |  |  |  |  |  |  | chr9 | 99060640 | 99060640 | C | A | het | . | . | . | 419 | 0 | 0% | C | 207 | 3 | 1.43% | M | Somatic | 0.0368596 |
| exonic | C9orf156 | synonymous SNV | NM_016481:c.C1293T:p.G431G |  |  |  |  |  |  |  |  |  |  |  | chr9 | 100667048 | 100667048 | G | A | het | . | . | . | 174 | 0 | 0% | G | 187 | 5 | 2.60% | R | Somatic | 0.0387451 |
| exonic | OR13C5 | nonsynonymous SNV | NM_001004482:c.G566A:p.C189Y | 265;Name=lod=16 | 0.93 | 0.33 | 0.33 | 0.33 | rs4117966 | 0 | 0.998 | 0.995665 | 0.603083 | 0.999455 | chr9 | 107361129 | 107361129 | C | T | het | . | . | . | 406 | 0 | 0% | C | 175 | 5 | 2.78% | Y | Somatic | 0.0026301 |
| exonic | FKBP15 | synonymous SNV | NM_015258:c.A186C:p.P62P | 423;Name=lod=70 |  |  |  |  |  |  |  |  |  |  | chr9 | 115969560 | 115969560 | T | G | het | . | . | . | 318 | 0 | 0% | T | 212 | 4 | 1.85% | K | Somatic | 0.0263276 |
| exonic | HNRNPH3 | synonymous SNV | NM_012207:c.A270G:p.R90R | 678;Name=lod=760 |  |  |  |  |  |  |  |  |  |  | chr10 | 70098278 | 70098278 | A | G | het | . | . | . | 166 | 1 | 0.60% | A | 105 | 5 | 4.55% | R | Somatic | 0.0380081 |
| exonic | TNC | nonsynonymous SNV | NM_002160:c.G1316A:p.C439Y | 378;Name=lod=46 |  |  |  |  |  | 0 | 1 | 0.999227 | 0.995011 | 1 | chr9 | 117848694 | 117848694 | C | T | het | . | . | . | 191 | 0 | 0% | C | 258 | 6 | 2.27% | Y | Somatic | 0.0372462 |
| exonic | PSMD5 | nonsynonymous SNV | NM_005047:c.C1058T:p.A353V | 530;Name=lod=190 |  |  |  |  |  | 0.06 | 0.016 | 0.999727 | 0.173201 | 0.999981 | chr9 | 123583691 | 123583691 | G | A | het | . | . | . | 243 | 1 | 0.41% | G | 106 | 6 | 5.36% | R | Somatic | 0.0045754 |
| exonic | GCKR | nonsynonymous SNV | NM_001486:c.A230G:p.E77G |  |  | 0.003 | 0.003 | 0.003 | rs8179206 | 0.02 | 0.021 | 0.966506 | 0.156956 | 0.975026 | chr2 | 27720442 | 27720442 | A | G | het | . | . | . | 103 | 0 | 0% | A | 84 | 4 | 4.55% | R | Somatic | 0.0434023 |
| intronic | GOLGA2 |  |  |  |  | 0.1 | 0.1 | 0.1 | rs12345218 |  |  |  |  |  | chr9 | 131028226 | 131028226 | G | A | het | . | . | . | 256 | 0 | 0% | G | 208 | 4 | 1.89% | R | Somatic | 0.0414555 |
| exonic | SETX | synonymous SNV | NM_015046:c.C3147T:p.H1049H |  |  | 0.1 | 0.1 | 0.1 | rs3739921 |  |  |  |  |  | chr9 | 135203838 | 135203838 | G | A | het | . | . | . | 478 | 1 | 0.21% | G | 159 | 7 | 4.22% | R | Somatic | 4.25E-04 |
| exonic | TTF1 | nonsynonymous SNV | NM_007344:c.G868T:p.A290S |  |  | 0.14 | 0.14 | 0.14 | rs8999 | 0.28 | 0.036 | 0.774592 | 0.001024 | 0.769966 | chr9 | 135277341 | 135277341 | C | A | het | . | . | . | 309 | 0 | 0% | C | 287 | 3 | 1.03% | M | Somatic | 0.1128719 |
| exonic | NOTCH1 | nonsynonymous SNV | NM_017617:c.G5830T:p.A1944S | 542;Name=lod=213 |  |  |  |  |  | 0.01 | 0.421 | 0.970285 | 0.999605 | 1 | chr9 | 139395108 | 139395108 | C | A | het | . | . | . | 362 | 0 | 0% | C | 307 | 4 | 1.29% | M | Somatic | 0.0451284 |
| exonic | TCF20 | nonsynonymous SNV | NM_005650:c.C3997A:p.L1333I | 558;Name=lod=247 |  |  |  |  |  | 0.04 | 0.788 | 0.999341 | 1 | 0.999995 | chr22 | 42607315 | 42607315 | G | T | het | . | . | . | 137 | 0 | 0% | G | 84 | 4 | 4.55% | K | Somatic | 0.0224301 |
| exonic | SLC34A3 | synonymous SNV | NM_001177316:c.C295T:p.L99L | 557;Name=lod=244 |  |  |  |  |  |  |  |  |  |  | chr9 | 140127146 | 140127146 | C | T | het | . | . | . | 267 | 0 | 0% | C | 163 | 4 | 2.40% | Y | Somatic | 0.0214395 |
| exonic | TLR7 | nonsynonymous SNV | NM_016562:c.G290A:p.R97K | 492;Name=lod=133 |  |  |  |  |  | 0.07 | 0.994 | 0.999707 | 0.155961 | 1 | chrX | 12903917 | 12903917 | G | A | het | . | . | . | 293 | 0 | 0% | G | 128 | 5 | 3.76% | R | Somatic | 0.0028146 |
| exonic | PIGA | synonymous SNV | NM_020473:c.C687T:p.A229A | 336;Name=lod=31 | 0.91 |  |  |  |  |  |  |  |  |  | chrX | 15339694 | 15339694 | G | A | het | . | . | . | 368 | 0 | 0% | G | 176 | 4 | 2.22% | R | Somatic | 0.0113805 |
| exonic | CXorf23 | nonsynonymous SNV | NM_198279:c.G1964A:p.G655D | 522;Name=lod=176 |  |  |  |  |  | 0 | 0.995 | 0.998487 | 0.718808 | 0.983407 | chrX | 19947958 | 19947958 | C | T | het | . | . | . | 361 | 0 | 0% | C | 231 | 2 | 0.86% | Y | Somatic | 0.1534627 |
| intronic | SAFB |  |  |  | 0.93 |  |  |  |  |  |  |  |  |  | chr19 | 5668128 | 5668128 | G | A | het | . | . | . | 124 | 0 | 0% | G | 148 | 7 | 4.52% | R | Somatic | 0.0153588 |
| exonic | TARDBP | nonsynonymous SNV | NM_007375:c.C779T:p.A260V | 746;Name=lod=1430 | 0.96 |  |  |  |  | 0 | 0.684 | 0.999105 | 0.999999 | 1 | chr1 | 11082245 | 11082245 | C | T | het | . | . | . | 157 | 0 | 0% | C | 106 | 5 | 4.50% | Y | Somatic | 0.0115497 |
| exonic | KIF4A | synonymous SNV | NM_012310:c.A969G:p.T323T | 621;Name=lod=444 |  | 0.1 | 0.1 | 0.1 | rs5936873 |  |  |  |  |  | chrX | 69550080 | 69550080 | A | G | het | . | . | . | 351 | 1 | 0.28% | A | 198 | 5 | 2.46% | R | Somatic | 0.0266414 |
| intronic | DRP2 |  |  |  |  | 0.47 | 0.47 | 0.47 | rs5967281 |  |  |  |  |  | chrX | 100514986 | 100514986 | G | A | het | . | . | . | 282 | 0 | 0% | G | 247 | 9 | 3.52% | R | Somatic | 0.0011603 |
| exonic | VSIG1 | synonymous SNV | NM_182607:c.C768T:p.F256F |  |  |  |  |  |  |  |  |  |  |  | chrX | 107319386 | 107319386 | C | T | het | . | . | . | 267 | 0 | 0% | C | 108 | 12 | 10% | Y | Somatic | 5.32E-07 |
| exonic | PSMD10 | nonsynonymous SNV | NM_002814:c.C193T:p.P65S | 562;Name=lod=257 |  |  |  |  |  | 0.01 | 0.897 | 0.984116 | 0.916479 | 0.9999 | chrX | 107332002 | 107332002 | G | A | het | . | . | . | 383 | 1 | 0.26% | G | 184 | 5 | 2.65% | R | Somatic | 0.0164996 |
| exonic | SLC25A3 | nonsynonymous SNV | NM_005888:c.G265C:p.V89L | 705;Name=lod=975 |  |  |  |  |  | 0.01 | 0.41 | 0.950115 | 0.999927 | 1 | chr12 | 98989318 | 98989318 | G | C | het | . | . | . | 206 | 0 | 0% | G | 107 | 5 | 4.46% | S | Somatic | 0.0051094 |
| exonic | GUCY2F | nonsynonymous SNV | NM_001522:c.T479A:p.I160N |  |  | 0.02 | 0.02 | 0.02 | rs33971675 | 0.51 | 0 | 0.97382 | 3.00E-06 | 0.997694 | chrX | 108718687 | 108718687 | A | T | het | . | . | . | 309 | 0 | 0% | A | 194 | 5 | 2.51% | W | Somatic | 0.008944 |
| exonic | DOCK11 | nonsynonymous SNV | NM_144658:c.G1237A:p.A413T | 584;Name=lod=315 |  |  |  |  |  | 0.03 | 0.96 | 0.999748 | 0.968437 | 1 | chrX | 117707829 | 117707829 | G | A | het | . | . | . | 269 | 0 | 0% | G | 158 | 4 | 2.47% | R | Somatic | 0.0194989 |
| exonic | GLUD2 | nonsynonymous SNV | NM_012084:c.G463A:p.D155N | 776;Name=lod=1894 |  |  |  |  |  | 0.04 | 0.003 | 0.876129 | 0.998139 | 0.999991 | chrX | 120182001 | 120182001 | G | A | het | . | . | . | 298 | 0 | 0% | G | 259 | 4 | 1.52% | R | Somatic | 0.0477171 |
| exonic | FSTL5 | nonsynonymous SNV | NM_001128427:c.G967T:p.D323Y | 625;Name=lod=464 |  |  |  |  |  | 1 | 0.727 | 0.812513 | 0.999978 | 0.999787 | chr4 | 162508652 | 162508652 | C | A | het | . | . | . | 199 | 0 | 0% | C | 86 | 4 | 4.44% | M | Somatic | 0.0089763 |
| exonic | MST4 | nonsynonymous SNV | NM_001042452:c.G973A:p.V325M | 432;Name=lod=76 |  |  |  |  |  | 0.03 | 0 | 0.920206 | 0.003895 | 0.999575 | chrX | 131207054 | 131207054 | G | A | het | . | . | . | 364 | 0 | 0% | G | 220 | 6 | 2.65% | R | Somatic | 0.0030308 |
| exonic | ATAD3B | nonsynonymous SNV | NM_031921:c.T1810C:p.Y604H |  | 0.91 | 0.01 | 0.01 | 0.01 | rs9792997 | 0.24 | 0.291125 | 0.001227 | 2.20E-05 | 0.185515 | chr1 | 1431060 | 1431060 | T | C | het | . | . | . | 65 | 1 | 1.52% | T | 64 | 8 | 11.11% | Y | Somatic | 0.0228343 |
| intronic | CHD5 |  |  |  |  |  |  |  |  |  |  |  |  |  | chr1 | 6194170 | 6194170 | T | G | het | . | . | . | 54 | 1 | 1.82% | T | 66 | 8 | 10.81% | K | Somatic | 0.0458441 |
| exonic | H6PD | nonsynonymous SNV | NM_004285:c.A1720C:p.T574P | 313;Name=lod=25 |  |  |  |  |  |  | 0.125 | 0.120218 | 0.012315 | 0.993612 | chr1 | 9324272 | 9324272 | A | C | het | . | . | . | 45 | 0 | 0% | A | 20 | 3 | 13.04% | M | Somatic | 0.035338 |
| exonic | EPHA2 | nonsynonymous SNV | NM_004431:c.A1348C:p.T450P | 374;Name=lod=44 |  |  |  |  |  | 0.02 | 0.113 | 0.959171 | 0.911119 | 0.999977 | chr1 | 16462230 | 16462230 | T | G | het | . | . | . | 48 | 0 | 0% | T | 122 | 14 | 10.29% | K | Somatic | 0.0120676 |
| splicing | CROCC(NM_014675:exon35:c.5784+2T>G) |  |  | 549;Name=lod=227 |  |  |  |  |  |  |  |  |  |  | chr1 | 17297264 | 17297264 | T | G | het | . | . | . | 51 | 0 | 0% | T | 35 | 5 | 12.50% | K | Somatic | 0.0141494 |
| UTR3 | C1QA |  |  |  |  |  |  |  |  | 0 |  |  |  |  | chr1 | 22965912 | 22965912 | A | C | het | . | . | . | 34 | 0 | 0% | A | 46 | 6 | 11.54% | M | Somatic | 0.0433017 |
| exonic | FAM46B | nonsynonymous SNV | NM_052943:c.C195G:p.S65R | 598;Name=lod=358 |  |  |  |  |  | 0.18 | 0.002 | 0.972775 | 0.006623 | 0.955119 | chr1 | 27338967 | 27338967 | G | C | het | . | . | . | 27 | 0 | 0% | G | 39 | 7 | 15.22% | S | Somatic | 0.0328504 |
| intronic | ZMYM1 |  |  |  |  |  |  |  |  |  |  |  |  |  | chr1 | 35577561 | 35577561 | C | A | het | . | . | . | 62 | 0 | 0% | C | 14 | 2 | 12.50% | M | Somatic | 0.03996 |
| exonic | MELK | synonymous SNV | NM_014791:c.A1443G:p.K481K | 423;Name=lod=70 |  | 0 | 0 | 0 | rs56176668 |  |  |  |  |  | chr9 | 36669341 | 36669341 | A | G | het | . | . | . | 185 | 0 | 0% | A | 86 | 4 | 4.44% | R | Somatic | 0.0109602 |
| intronic | SPATA6 |  |  |  |  |  |  |  |  |  |  |  |  |  | chr1 | 48937573 | 48937573 | T | G | het | . | . | . | 37 | 0 | 0% | T | 35 | 5 | 12.50% | K | Somatic | 0.0333037 |
| intronic | ELAVL4 |  |  |  |  |  |  |  |  |  |  |  |  |  | chr1 | 50610580 | 50610580 | G | T | het | . | . | . | 94 | 0 | 0% | G | 17 | 2 | 10.53% | K | Somatic | 0.0270228 |
| exonic | NRD1 | stopgain SNV | NM_001101662:c.T1223A:p.L408X | 627;Name=lod=470 |  |  |  |  |  | 0.02 | 0.735008 | 0.99827 | 1 | 0.998481 | chr1 | 52285540 | 52285540 | A | T | het | . | . | . | 71 | 0 | 0% | A | 27 | 3 | 10% | W | Somatic | 0.0243624 |
| intergenic | ST6GALNAC3(dist=69573),ST6GALNAC5(dist=166944) |  |  |  | 0.92 |  |  |  |  |  |  |  |  |  | chr1 | 77166242 | 77166242 | A | C | het | . | . | . | 51 | 0 | 0% | A | 73 | 11 | 13.10% | M | Somatic | 0.0041505 |
| intronic | ELTD1 |  |  |  |  |  |  |  |  |  |  |  |  |  | chr1 | 79358762 | 79358762 | T | A | het | . | . | . | 50 | 1 | 1.96% | T | 31 | 5 | 13.89% | W | Somatic | 0.041931 |
| intronic | ELTD1 |  |  |  |  |  |  |  |  |  |  |  |  |  | chr1 | 79358763 | 79358763 | G | A | het | . | . | . | 55 | 0 | 0% | G | 33 | 4 | 10.81% | R | Somatic | 0.0236368 |
| exonic | DDAH1 | nonsynonymous SNV | NM_012137:c.A271C:p.T91P | 679;Name=lod=766 |  |  |  |  |  | 0 | 0.999 | 0.854799 | 0.488112 | 0.999852 | chr1 | 85930458 | 85930458 | T | G | het | . | . | . | 36 | 0 | 0% | T | 20 | 4 | 16.67% | K | Somatic | 0.0217909 |
| intronic | FAM40A |  |  |  |  |  |  |  |  |  |  |  |  |  | chr1 | 110592049 | 110592049 | T | C | het | . | . | . | 33 | 0 | 0% | T | 31 | 7 | 18.42% | Y | Somatic | 0.0094897 |
| intergenic | SRGAP2P2(dist=65497),LOC728855(dist=140538) |  |  |  | 0.99 |  |  |  |  |  |  |  |  |  | chr1 | 144159974 | 144159974 | C | T | het | . | . | . | 86 | 0 | 0% | C | 16 | 2 | 11.11% | Y | Somatic | 0.0285661 |
| intergenic | SRGAP2P2(dist=73464),LOC728855(dist=132571) |  |  |  | 0.99 |  |  |  |  |  |  |  |  |  | chr1 | 144167941 | 144167941 | C | G | het | . | . | . | 97 | 0 | 0% | C | 20 | 4 | 16.67% | S | Somatic | 0.0012508 |
| intergenic | SRGAP2P2(dist=94068),LOC728855(dist=111967) |  |  |  | 0.99 |  |  |  |  |  |  |  |  |  | chr1 | 144188545 | 144188545 | C | T | het | . | . | . | 74 | 0 | 0% | C | 14 | 2 | 12.50% | Y | Somatic | 0.0299625 |
| intergenic | SRGAP2P2(dist=94076),LOC728855(dist=111959) |  |  |  | 0.99 |  |  |  |  |  |  |  |  |  | chr1 | 144188553 | 144188553 | G | T | het | . | . | . | 75 | 0 | 0% | G | 11 | 2 | 15.38% | K | Somatic | 0.0203762 |
| intergenic | SRGAP2P2(dist=114757),LOC728855(dist=91278) |  |  |  | 0.99 |  |  |  |  |  |  |  |  |  | chr1 | 144209234 | 144209234 | T | A | het | . | . | . | 101 | 0 | 0% | T | 25 | 3 | 10.71% | W | Somatic | 0.0093733 |
| intronic | NBPF11 |  |  | 740;Name=lod=1360 | 1 |  |  |  |  | 0 |  |  |  |  | chr1 | 147621810 | 147621810 | T | C | het | . | . | . | 49 | 1 | 2% | T | 41 | 9 | 18% | Y | Somatic | 0.0078302 |
| exonic | PPIAL4A,PPIAL4B | synonymous SNV | NM_001143883:c.T345C:p.C115C | 687;Name=lod=823 | 1 |  |  |  | rs4105356 |  |  |  |  |  | chr1 | 147955000 | 147955000 | A | G | het | . | . | . | 105 | 2 | 1.87% | A | 43 | 5 | 10.42% | R | Somatic | 0.0298539 |
| intergenic | PPIAL4D(dist=76775),NBPF15(dist=278877) |  |  |  | 0.99 |  |  |  |  |  |  |  |  |  | chr1 | 148279311 | 148279311 | A | G | het | . | . | . | 57 | 0 | 0% | A | 12 | 2 | 14.29% | R | Somatic | 0.0366197 |
| intergenic | PPIAL4D(dist=110451),NBPF15(dist=245201) |  |  |  | 0.99 |  |  |  |  |  |  |  |  |  | chr1 | 148312987 | 148312987 | G | A | het | . | . | . | 67 | 0 | 0% | G | 15 | 2 | 11.76% | R | Somatic | 0.0390132 |
| exonic | IQCH | nonsynonymous SNV | NM_001031715:c.C2459T:p.S820L | 383;Name=lod=48 |  |  |  |  |  | 0 | 0.983 | 0.999135 | 0.784293 | 1 | chr15 | 67713869 | 67713869 | C | T | het | . | . | . | 143 | 0 | 0% | C | 108 | 5 | 4.42% | Y | Somatic | 0.0159332 |
| intronic | MRPL9 |  |  |  |  |  |  |  |  |  |  |  |  |  | chr1 | 151733379 | 151733379 | C | A | het | . | . | . | 16 | 0 | 0% | C | 6 | 4 | 40% | M | Somatic | 0.0140468 |
| exonic | ZEB2 | synonymous SNV | NM_001171653:c.C1941T:p.S647S | 785;Name=lod=2069 |  |  |  |  |  |  |  |  |  |  | chr2 | 145156741 | 145156741 | G | A | het | . | . | . | 207 | 0 | 0% | G | 87 | 4 | 4.40% | R | Somatic | 0.0082998 |
| exonic | HRNR | nonsynonymous SNV | NM_001009931:c.G8226C:p.Q2742H |  | 0.98 |  |  |  |  | 0.12 | 0.369497 | 0.008203 | 0.279705 | 0.621915 | chr1 | 152185879 | 152185879 | C | G | het | . | . | . | 29 | 0 | 0% | C | 13 | 13 | 50% | S | Somatic | 7.17E-06 |
| ncRNA_intronic | LOC100505666 |  |  | 272;Name=lod=17 |  |  |  |  |  |  |  |  |  |  | chr1 | 155025869 | 155025869 | T | G | het | . | . | . | 64 | 1 | 1.54% | T | 118 | 20 | 14.49% | K | Somatic | 0.0022296 |
| exonic | SH2D2A | nonsynonymous SNV | NM_001161443:c.A983C:p.H328P |  |  |  |  |  |  | 0.02 | 0.969 | 0.948835 | 0.296847 | 0.016398 | chr1 | 156777073 | 156777073 | T | G | het | . | . | . | 25 | 0 | 0% | T | 60 | 16 | 21.05% | K | Somatic | 0.0067722 |
| exonic | PVRL4 | nonsynonymous SNV | NM_030916:c.A391C:p.T131P | 433;Name=lod=77 |  |  |  |  |  | 0 | 0.997 | 0.997842 | 0.931383 | 0.999874 | chr1 | 161049428 | 161049428 | T | G | het | . | . | . | 53 | 0 | 0% | T | 55 | 7 | 11.29% | K | Somatic | 0.0112247 |
| exonic | SELL | synonymous SNV | NM_000655:c.C534T:p.C178C | 321;Name=lod=27 |  |  |  |  |  |  |  |  |  |  | chr1 | 169676568 | 169676568 | G | A | het | . | . | . | 92 | 0 | 0% | G | 34 | 4 | 10.53% | R | Somatic | 0.0064984 |
| intronic | DARS2 |  |  |  |  |  |  |  |  |  |  |  |  |  | chr1 | 173819439 | 173819439 | T | G | het | . | . | . | 18 | 0 | 0% | T | 10 | 6 | 37.50% | K | Somatic | 0.0059543 |
| intronic | RABGAP1L |  |  |  |  |  |  |  |  |  |  |  |  |  | chr1 | 174671222 | 174671222 | G | T | het | . | . | . | 76 | 1 | 1.30% | G | 45 | 5 | 10% | K | Somatic | 0.0346338 |
| intronic | ARPC5 |  |  |  |  |  |  |  |  |  |  |  |  |  | chr1 | 183604625 | 183604625 | T | G | het | . | . | . | 39 | 0 | 0% | T | 19 | 3 | 13.64% | K | Somatic | 0.0427897 |
| intronic | SWT1 |  |  |  |  |  |  |  |  |  |  |  |  |  | chr1 | 185200890 | 185200890 | T | C | het | . | . | . | 59 | 0 | 0% | T | 15 | 2 | 11.76% | Y | Somatic | 0.0477193 |
| intronic | ZBTB41 |  |  |  |  |  |  |  |  |  |  |  |  |  | chr1 | 197157576 | 197157576 | T | A | het | . | . | . | 54 | 0 | 0% | T | 14 | 2 | 12.50% | W | Somatic | 0.0496894 |
| intronic | CACNA1S |  |  | 265;Name=lod=16 |  |  |  |  |  |  |  |  |  |  | chr1 | 201009528 | 201009528 | A | C | het | . | . | . | 38 | 0 | 0% | A | 41 | 6 | 12.77% | M | Somatic | 0.0245512 |
| exonic | KIAA0922 | synonymous SNV | NM_001131007:c.G1098A:p.V366V |  |  |  |  |  |  |  |  |  |  |  | chr4 | 154506024 | 154506024 | G | A | het | . | . | . | 206 | 0 | 0% | G | 87 | 4 | 4.40% | R | Somatic | 0.0084128 |
| UTR5 | RPS6KC1 |  |  | 624;Name=lod=457 |  |  |  |  |  |  |  |  |  |  | chr1 | 213224744 | 213224744 | A | G | het | . | . | . | 41 | 0 | 0% | A | 38 | 5 | 11.63% | R | Somatic | 0.0311803 |
| exonic | RYR2 | synonymous SNV | NM_001035:c.G8742A:p.T2914T | 549;Name=lod=227 |  |  |  |  |  |  |  |  |  |  | chr1 | 237838058 | 237838058 | G | A | het | . | . | . | 75 | 0 | 0% | G | 26 | 3 | 10.34% | R | Somatic | 0.0200655 |
| exonic | FMN2 | synonymous SNV | NM_020066:c.T2967G:p.P989P |  | 0.9 |  |  |  |  |  |  |  |  |  | chr1 | 240371079 | 240371079 | T | G | het | . | . | . | 53 | 1 | 1.85% | T | 51 | 7 | 12.07% | K | Somatic | 0.0381778 |
| intronic | SDCCAG8 |  |  |  |  |  |  |  |  |  |  |  |  |  | chr1 | 243480241 | 243480241 | C | T | het | . | . | . | 49 | 0 | 0% | C | 23 | 3 | 11.54% | Y | Somatic | 0.0385043 |
| intronic | C1orf150 |  |  |  |  |  |  |  |  |  |  |  |  |  | chr1 | 247729224 | 247729224 | A | T | het | . | . | . | 42 | 0 | 0% | A | 33 | 4 | 10.81% | W | Somatic | 0.0439567 |
| exonic | MUC16 | nonsynonymous SNV | NM_024690:c.G37541C:p.S12514T |  |  | 0.64 | 0.64 | 0.64 | rs12981679 |  |  |  |  |  | chr19 | 9019605 | 9019605 | C | G | het | . | . | . | 151 | 0 | 0% | C | 88 | 4 | 4.35% | S | Somatic | 0.0197157 |
| splicing | IAH1(NM_001039613:exon4:c.445+2T>A) |  |  | 389;Name=lod=51 |  |  |  |  |  |  |  |  |  |  | chr2 | 9621578 | 9621578 | T | A | het | . | . | . | 45 | 0 | 0% | T | 33 | 4 | 10.81% | W | Somatic | 0.0377603 |
| intronic | ADAM17 |  |  |  |  |  |  |  |  |  |  |  |  |  | chr2 | 9658381 | 9658381 | G | T | het | . | . | . | 36 | 0 | 0% | G | 8 | 3 | 27.27% | K | Somatic | 0.0101758 |
| exonic | MSGN1 | nonsynonymous SNV | NM_001105569:c.A436C:p.T146P | 521;Name=lod=175 |  |  |  |  |  | 0.02 | 0.997 | 0.999115 | 0.99162 | 0.99997 | chr2 | 17998221 | 17998221 | A | C | het | . | . | . | 60 | 1 | 1.64% | A | 99 | 11 | 10% | M | Somatic | 0.0335794 |
| exonic | OTOF | nonsynonymous SNV | NM_194322:c.A2779C:p.T927P | 574;Name=lod=286 |  |  |  |  |  | 0.02 | 0.008 | 0.845129 | 0.593252 | 0.999487 | chr2 | 26687848 | 26687848 | T | G | het | . | . | . | 52 | 1 | 1.89% | T | 100 | 16 | 13.79% | K | Somatic | 0.0111792 |
| exonic | ALK | synonymous SNV | NM_004304:c.A3606G:p.G1202G | 546;Name=lod=220 |  |  |  |  |  |  |  |  |  |  | chr2 | 29443611 | 29443611 | T | C | het | lung | carcinoma | adenocarcinoma | 27 | 0 | 0% | T | 72 | 11 | 13.25% | Y | Somatic | 0.0378612 |
| exonic | USP34 | nonsynonymous SNV | NM_014709:c.G2815A:p.D939N | 670;Name=lod=707 |  |  |  |  |  | 0.1 | 0.912 | 0.999145 | 0.9962 | 1 | chr2 | 61561036 | 61561036 | C | T | het | . | . | . | 85 | 0 | 0% | C | 23 | 3 | 11.54% | Y | Somatic | 0.0117215 |
| exonic | EPAS1 | stopgain SNV | NM_001430:c.G358T:p.G120X | 567;Name=lod=268 |  |  |  |  |  | 0 | 0.735508 | 0.999684 | 1 | 1 | chr2 | 46583430 | 46583430 | G | T | het | . | . | . | 116 | 0 | 0% | G | 88 | 4 | 4.35% | K | Somatic | 0.0368814 |
| intergenic | MIR4436A(dist=45230),NONE(dist=NONE) |  |  |  |  |  |  |  |  |  |  |  |  |  | chr2 | 89157198 | 89157198 | T | C | het | . | . | . | 76 | 1 | 1.30% | T | 15 | 17 | 53.12% | Y | Somatic | 2.64E-10 |
| exonic | GPAT2 | nonsynonymous SNV | NM_207328:c.C2074T:p.R692C | 502;Name=lod=146 |  |  |  |  | rs76099874 | 0.01 | 0.192 | 0.999539 | 0.082535 | 0.990256 | chr2 | 96688929 | 96688929 | G | A | het | large_intestine | carcinoma | adenocarcinoma | 36 | 0 | 0% | G | 39 | 11 | 22% | R | Somatic | 0.0015269 |
| exonic | C2orf29 | nonsynonymous SNV | NM_017546:c.A409C:p.T137P | 767;Name=lod=1746 |  |  |  |  |  | 0.12 | 0.486 | 0.875557 | 0.998279 | 1 | chr2 | 101869835 | 101869835 | A | C | het | . | . | . | 29 | 0 | 0% | A | 26 | 7 | 21.21% | M | Somatic | 0.0086866 |
| exonic | PAX8 | nonsynonymous SNV | NM_003466:c.A665C:p.H222P | 596;Name=lod=353 |  |  |  |  |  | 0.01 | 0.822 | 0.996251 | 0.600313 | 0.999305 | chr2 | 113999240 | 113999240 | T | G | het | . | . | . | 32 | 0 | 0% | T | 115 | 13 | 10.16% | K | Somatic | 0.0482968 |
| exonic | GLI2 | synonymous SNV | NM_005270:c.C3693G:p.G1231G |  |  |  |  |  |  |  |  |  |  |  | chr2 | 121747183 | 121747183 | C | G | het | . | . | . | 55 | 0 | 0% | C | 36 | 4 | 10% | S | Somatic | 0.028707 |
| intronic | ERCC3 |  |  |  |  |  |  |  |  |  |  |  |  |  | chr2 | 128051611 | 128051611 | T | G | het | . | . | . | 28 | 0 | 0% | T | 20 | 4 | 16.67% | K | Somatic | 0.0392502 |
| intergenic | LOC401010(dist=16752),TUBA3D(dist=14361) |  |  |  | 0.98 |  |  |  |  |  |  |  |  |  | chr2 | 132219219 | 132219219 | G | T | het | . | . | . | 38 | 0 | 0% | G | 14 | 3 | 17.65% | K | Somatic | 0.0259196 |
| intergenic | LOC401010(dist=16763),TUBA3D(dist=14350) |  |  |  | 0.98 |  |  |  |  |  |  |  |  |  | chr2 | 132219230 | 132219230 | G | C | het | . | . | . | 42 | 0 | 0% | G | 13 | 3 | 18.75% | S | Somatic | 0.0181488 |
| exonic | C2orf27B | nonsynonymous SNV | NM_214461:c.C406T:p.H136Y |  | 0.99 |  |  |  | rs11489970 | 0 | 0.967 | 0.914696 | 5.02E-04 | 0.855768 | chr2 | 132552960 | 132552960 | G | A | het | . | . | . | 51 | 1 | 1.92% | G | 68 | 9 | 11.69% | R | Somatic | 0.0385743 |
| exonic | C2orf27B | nonsynonymous SNV | NM_214461:c.T287G:p.V96G |  | 0.99 |  |  |  |  | 0 | 0.693 | 0.04305 | 0.002702 | 0.603471 | chr2 | 132553079 | 132553079 | A | C | het | . | . | . | 30 | 0 | 0% | A | 40 | 6 | 13.04% | M | Somatic | 0.0428454 |
| ncRNA_exonic | MIR663B |  |  |  | 0.91 |  |  |  |  |  |  |  |  |  | chr2 | 133014587 | 133014587 | C | T | het | . | . | . | 55 | 1 | 1.79% | C | 47 | 8 | 14.55% | Y | Somatic | 0.0147519 |
| ncRNA_exonic | MIR663B |  |  |  | 0.91 |  |  |  |  |  |  |  |  |  | chr2 | 133014617 | 133014617 | A | G | het | . | . | . | 47 | 0 | 0% | A | 30 | 5 | 14.29% | R | Somatic | 0.0118977 |
| intronic | EPC2 |  |  |  |  |  |  |  |  |  |  |  |  |  | chr2 | 149522513 | 149522513 | C | T | het | . | . | . | 53 | 0 | 0% | C | 13 | 2 | 13.33% | Y | Somatic | 0.0460931 |
| exonic | KIF5C | synonymous SNV | NM_004522:c.C1251T:p.Y417Y |  |  |  |  |  |  |  |  |  |  |  | chr2 | 149829983 | 149829983 | C | T | het | . | . | . | 48 | 0 | 0% | C | 22 | 3 | 12% | Y | Somatic | 0.0369799 |
| exonic | CCDC148 | nonsynonymous SNV | NM_138803:c.C1169T:p.A390V | 532;Name=lod=193 |  |  |  |  |  | 0.1 | 0.001 | 0.961188 | 0.76857 | 0.932338 | chr2 | 159107366 | 159107366 | G | A | het | . | . | . | 87 | 1 | 1.14% | G | 25 | 3 | 10.71% | R | Somatic | 0.0431218 |
| exonic | DYNC1I2 | synonymous SNV | NM_001378:c.T1329C:p.D443D | 721;Name=lod=1131 |  |  |  |  | rs62184168 |  |  |  |  |  | chr2 | 172585298 | 172585298 | T | C | het | lung | carcinoma | adenocarcinoma | 71 | 0 | 0% | T | 27 | 4 | 12.90% | Y | Somatic | 0.0074043 |
| intronic | RAPGEF4 |  |  | 300;Name=lod=22 |  |  |  |  |  |  |  |  |  |  | chr2 | 173782729 | 173782729 | A | G | het | . | . | . | 50 | 0 | 0% | A | 12 | 2 | 14.29% | R | Somatic | 0.0451389 |
| exonic | TTN | nonsynonymous SNV | NM_003319:c.G25495A:p.A8499T | 624;Name=lod=460 |  |  |  |  |  |  |  |  |  |  | chr2 | 179472920 | 179472920 | C | T | het | . | . | . | 56 | 0 | 0% | C | 18 | 3 | 14.29% | Y | Somatic | 0.0181818 |
| exonic | BMPR2 | nonsynonymous SNV | NM_001204:c.C2439A:p.N813K | 737;Name=lod=1316 |  |  |  |  |  | 0.08 | 0.009 | 0.768277 | 0.007279 | 0.993096 | chr2 | 203420827 | 203420827 | C | A | het | . | . | . | 76 | 1 | 1.30% | C | 36 | 4 | 10% | M | Somatic | 0.0459269 |
| intronic | IKZF2 |  |  |  |  |  |  |  |  |  |  |  |  |  | chr2 | 213872837 | 213872837 | A | T | het | . | . | . | 25 | 0 | 0% | A | 6 | 3 | 33.33% | W | Somatic | 0.0140374 |
| intronic | MREG |  |  | 595;Name=lod=348 |  |  |  |  |  |  |  |  |  |  | chr2 | 216811839 | 216811839 | G | A | het | . | . | . | 51 | 0 | 0% | G | 13 | 2 | 13.33% | R | Somatic | 0.048951 |
| exonic | C20orf72 | synonymous SNV | NM_052865:c.T846C:p.D282D | 525;Name=lod=181 |  | 0.27 | 0.27 | 0.27 | rs28455091 |  |  |  |  |  | chr20 | 17968923 | 17968923 | T | C | het | . | . | . | 131 | 0 | 0% | T | 88 | 4 | 4.35% | Y | Somatic | 0.0278605 |
| intronic | CHRND |  |  |  |  |  |  |  |  |  |  |  |  |  | chr2 | 233390990 | 233390990 | A | C | het | . | . | . | 28 | 0 | 0% | A | 36 | 9 | 20% | M | Somatic | 0.009128 |
| exonic | RNPEPL1 | nonsynonymous SNV | NM_018226:c.A740C:p.H247P | 522;Name=lod=177 |  |  |  |  |  | 0.21 | 0.903 | 0.036222 | 0.012474 | 0.986807 | chr2 | 241512597 | 241512597 | A | C | het | . | . | . | 36 | 0 | 0% | A | 19 | 3 | 13.64% | M | Somatic | 0.0499093 |
| intronic | AGXT |  |  |  |  |  |  |  |  |  |  |  |  |  | chr2 | 241817607 | 241817607 | A | C | het | . | . | . | 37 | 0 | 0% | A | 59 | 7 | 10.61% | M | Somatic | 0.039306 |
| exonic | C2orf85 | nonsynonymous SNV | NM_173821:c.C560T:p.P187L |  |  |  |  |  |  | 0.13 | 0.001 | 0.002642 | 0.004216 | 0.564312 | chr2 | 242814267 | 242814267 | C | T | het | . | . | . | 55 | 0 | 0% | C | 22 | 3 | 12% | Y | Somatic | 0.0279942 |
| exonic | MFAP3L | nonsynonymous SNV | NM_001009554:c.C418G:p.L140V | 446;Name=lod=87 |  |  |  |  |  | 0 | 0.98 | 0.999513 | 0.863601 | 1 | chr4 | 170913032 | 170913032 | G | C | het | . | . | . | 118 | 0 | 0% | G | 154 | 7 | 4.35% | S | Somatic | 0.0201406 |
| intergenic | LRRC3B(dist=432741),NEK10(dist=72091) |  |  |  |  |  |  |  |  |  |  |  |  |  | chr3 | 27185006 | 27185006 | C | A | het | . | . | . | 64 | 0 | 0% | C | 16 | 2 | 11.11% | M | Somatic | 0.0460705 |
| intronic | EOMES |  |  |  |  |  |  |  |  |  |  |  |  |  | chr3 | 27760397 | 27760397 | C | T | het | . | . | . | 84 | 0 | 0% | C | 27 | 3 | 10% | Y | Somatic | 0.016884 |
| intronic | SACM1L |  |  |  |  |  |  |  |  |  |  |  |  |  | chr3 | 45764919 | 45764919 | A | T | het | . | . | . | 70 | 0 | 0% | A | 14 | 2 | 12.50% | W | Somatic | 0.0328317 |
| exonic | NBEAL2 | nonsynonymous SNV | NM_015175:c.A6529C:p.T2177P | 627;Name=lod=471 |  |  |  |  |  | 0 |  |  |  |  | chr3 | 47046780 | 47046780 | A | C | het | . | . | . | 57 | 1 | 1.72% | A | 46 | 6 | 11.54% | M | Somatic | 0.0413106 |
| exonic | CELSR3 | nonsynonymous SNV | NM_001407:c.A5770C:p.T1924P |  |  |  |  |  |  | 0.14 | 0.555628 | 0.900695 | 0.730844 | 0.946448 | chr3 | 48689463 | 48689463 | T | G | het | . | . | . | 45 | 0 | 0% | T | 47 | 8 | 14.55% | K | Somatic | 0.006543 |
| splicing | WDR6(NM_018031:exon1:c.190+2T>G) |  |  | 539;Name=lod=207 |  |  |  |  |  |  |  |  |  |  | chr3 | 49044966 | 49044966 | T | G | het | . | . | . | 31 | 0 | 0% | T | 53 | 7 | 11.67% | K | Somatic | 0.0477153 |
| exonic | HMGCS2 | nonsynonymous SNV | NM_001166107:c.G695A:p.R232H | 397;Name=lod=55 |  |  |  |  |  | 0.53 | 0.979 | 0.91047 | 0.985278 | 0.999855 | chr1 | 120301770 | 120301770 | C | T | het | lung | carcinoma | small_cell_carcinoma | 208 | 0 | 0% | C | 89 | 4 | 4.30% | Y | Somatic | 0.0087092 |
| intronic | MAPKAPK3 |  |  |  |  |  |  |  |  |  |  |  |  |  | chr3 | 50683232 | 50683232 | A | C | het | . | . | . | 56 | 1 | 1.75% | A | 47 | 9 | 16.07% | M | Somatic | 0.0075286 |
| exonic | ERC2 | stopgain SNV | NM_015576:c.T1991A:p.L664X | 654;Name=lod=608 |  |  |  |  |  | 0.03 |  |  |  |  | chr3 | 56041279 | 56041279 | A | T | het | . | . | . | 71 | 0 | 0% | A | 12 | 2 | 14.29% | W | Somatic | 0.0254902 |
| exonic | ERC2 | nonsynonymous SNV | NM_015576:c.T1990A:p.L664I | 654;Name=lod=608 |  |  |  |  |  | 0.17 |  |  |  |  | chr3 | 56041280 | 56041280 | A | T | het | . | . | . | 71 | 0 | 0% | A | 12 | 2 | 14.29% | W | Somatic | 0.0254902 |
| exonic | GXYLT2 | nonsynonymous SNV | NM_001080393:c.T754G:p.Y252D | 574;Name=lod=286 |  |  |  |  |  | 0 |  |  |  |  | chr3 | 73004402 | 73004402 | T | G | het | . | . | . | 67 | 1 | 1.47% | T | 48 | 7 | 12.73% | K | Somatic | 0.0145811 |
| intronic | CNTN3 |  |  |  |  |  |  |  |  |  |  |  |  |  | chr3 | 74385822 | 74385822 | C | A | het | . | . | . | 58 | 0 | 0% | C | 11 | 2 | 15.38% | M | Somatic | 0.0313883 |
| exonic | GBE1 | synonymous SNV | NM_000158:c.G1080A:p.T360T | 648;Name=lod=573 |  |  |  |  |  |  |  |  |  |  | chr3 | 81643087 | 81643087 | C | T | het | . | . | . | 46 | 0 | 0% | C | 8 | 2 | 20% | Y | Somatic | 0.0292208 |
| exonic | LSAMP | synonymous SNV | NM_002338:c.G930A:p.S310S | 657;Name=lod=626 |  |  |  |  |  |  |  |  |  |  | chr3 | 115529251 | 115529251 | C | T | het | . | . | . | 42 | 0 | 0% | C | 9 | 2 | 18.18% | Y | Somatic | 0.0399129 |
| splicing | TMEM39A(NM_018266:exon5:c.337-1G>T) |  |  | 638;Name=lod=521 |  |  |  |  |  |  |  |  |  |  | chr3 | 119171378 | 119171378 | C | A | het | . | . | . | 80 | 0 | 0% | C | 18 | 2 | 10% | M | Somatic | 0.0383838 |
| intronic | C3orf25 |  |  |  |  | 0.4 | 0.4 | 0.4 | rs6764765 |  |  |  |  |  | chr3 | 129120743 | 129120743 | A | G | het | . | . | . | 14 | 0 | 0% | A | 26 | 20 | 43.48% | R | Somatic | 0.0013379 |
| intronic | XRN1 |  |  |  |  |  |  |  |  |  |  |  |  |  | chr3 | 142094597 | 142094597 | C | A | het | . | . | . | 65 | 0 | 0% | C | 27 | 3 | 10% | M | Somatic | 0.0293321 |
| UTR5 | PLSCR4 |  |  | 333;Name=lod=30 |  |  |  |  |  |  |  |  |  |  | chr3 | 145939860 | 145939860 | T | G | het | . | . | . | 73 | 0 | 0% | T | 15 | 2 | 11.76% | K | Somatic | 0.0339576 |
| intronic | ARHGEF26 |  |  |  |  |  |  |  |  |  |  |  |  |  | chr3 | 153867264 | 153867264 | G | T | het | . | . | . | 74 | 0 | 0% | G | 18 | 2 | 10% | K | Somatic | 0.0434683 |
| intronic | IQCJ-SCHIP1,SCHIP1 |  |  | 716;Name=lod=1080 |  |  |  |  |  |  |  |  |  |  | chr3 | 159482217 | 159482217 | A | C | het | . | . | . | 34 | 0 | 0% | A | 23 | 4 | 14.81% | M | Somatic | 0.03363 |
| exonic | MACF1 | nonsynonymous SNV | NM_012090:c.G13934A:p.G4645D | 500;Name=lod=143 |  |  |  |  |  | 0.02 | 0.721622 | 0.999825 | 0.752176 | 0.999998 | chr1 | 39914288 | 39914288 | G | A | het | . | . | . | 119 | 0 | 0% | G | 67 | 3 | 4.29% | R | Somatic | 0.0494305 |
| intronic | SI |  |  |  |  |  |  |  |  |  |  |  |  |  | chr3 | 164737354 | 164737354 | A | T | het | . | . | . | 99 | 0 | 0% | A | 17 | 2 | 10.53% | W | Somatic | 0.0247718 |
| exonic | TNIK | synonymous SNV | NM_001161561:c.G1452A:p.V484V | 676;Name=lod=746 |  |  |  |  |  |  |  |  |  |  | chr3 | 170856049 | 170856049 | C | T | het | . | . | . | 55 | 0 | 0% | C | 24 | 3 | 11.11% | Y | Somatic | 0.0330285 |
| splicing | TNIK(NM_015028:exon9:c.640-1G>C,NM_001161566:exon9:c.640-1G>C,NM_001161561:exon9:c.640-1G>C,NM_001161565:exon9:c.640-1G>C,NM_001161560:exon9:c.640-1G>C,NM_001161562:exon9:c.640-1G>C,NM_001161563:exon9:c.640-1G>C,NM_001161564:exon9:c.640-1G>C) |  |  | 494;Name=lod=136 |  |  |  |  |  |  |  |  |  |  | chr3 | 170895170 | 170895170 | C | G | het | . | . | . | 41 | 0 | 0% | C | 10 | 2 | 16.67% | S | Somatic | 0.0478955 |
| exonic | KLHL6 | synonymous SNV | NM_130446:c.A1356C:p.A452A |  |  |  |  |  |  |  |  |  |  |  | chr3 | 183210490 | 183210490 | T | G | het | . | . | . | 50 | 1 | 1.96% | T | 58 | 8 | 12.12% | K | Somatic | 0.0399492 |
| exonic | GP5 | nonsynonymous SNV | NM_004488:c.A898C:p.T300P |  |  |  |  |  |  | 0.02 | 0.998 | 0.99448 | 0.840589 | 0.999199 | chr3 | 194118114 | 194118114 | T | G | het | . | . | . | 33 | 0 | 0% | T | 37 | 5 | 11.90% | K | Somatic | 0.0492873 |
| exonic | MUC4 | nonsynonymous SNV | NM_018406:c.G2857C:p.E953Q |  |  | 0.76 | 0.76 | 0.76 | rs13095016 | 0.33 |  |  |  |  | chr3 | 195515594 | 195515594 | C | G | het | . | . | . | 17 | 0 | 0% | C | 27 | 15 | 35.71% | S | Somatic | 0.0024733 |
| exonic | PIGZ | nonsynonymous SNV | NM_025163:c.G442C:p.A148P | 381;Name=lod=47 |  |  |  |  |  |  | 0.285 | 0.97872 | 0.597699 | 0.966747 | chr3 | 196675326 | 196675326 | C | G | het | . | . | . | 42 | 0 | 0% | C | 56 | 8 | 12.50% | S | Somatic | 0.0146766 |
| intronic | GRK4 |  |  |  |  |  |  |  |  |  |  |  |  |  | chr4 | 2988911 | 2988911 | A | G | het | . | . | . | 46 | 0 | 0% | A | 24 | 4 | 14.29% | R | Somatic | 0.0177947 |
| intronic | GRK4 |  |  |  |  |  |  |  |  |  |  |  |  |  | chr4 | 2988919 | 2988919 | C | T | het | . | . | . | 48 | 0 | 0% | C | 23 | 3 | 11.54% | Y | Somatic | 0.0401086 |
| intronic | PPP2R2C |  |  |  |  |  |  |  |  |  |  |  |  |  | chr4 | 6349531 | 6349531 | T | C | het | . | . | . | 20 | 0 | 0% | T | 14 | 4 | 22.22% | Y | Somatic | 0.041455 |
| exonic | CPZ | nonsynonymous SNV | NM_003652:c.C974G:p.A325G | 361;Name=lod=39 |  |  |  |  |  | 0.38 | 0.849 | 0.992921 | 0.129628 | 0.99655 | chr4 | 8608564 | 8608564 | C | G | het | . | . | . | 40 | 0 | 0% | C | 58 | 8 | 12.12% | S | Somatic | 0.019045 |
| intergenic | DEFB131(dist=45245),MIR548I2(dist=60304) |  |  | 596;Name=lod=353 | 0.95 |  |  |  | rs75869556 | 0.03 |  |  |  |  | chr4 | 9497485 | 9497485 | G | A | het | . | . | . | 19 | 0 | 0% | G | 28 | 7 | 20% | R | Somatic | 0.0379701 |
| exonic | DRD5 | nonsynonymous SNV | NM_000798:c.C222G:p.N74K | 633;Name=lod=499 | 0.95 |  |  |  |  | 1 | 0 | 0.900924 | 1.20E-05 | 0.999996 | chr4 | 9783875 | 9783875 | C | G | het | . | . | . | 60 | 1 | 1.64% | C | 46 | 6 | 11.54% | S | Somatic | 0.03562 |
| intronic | TBC1D19 |  |  |  |  |  |  |  |  |  |  |  |  |  | chr4 | 26737061 | 26737061 | A | T | het | . | . | . | 50 | 1 | 1.96% | A | 13 | 3 | 18.75% | W | Somatic | 0.0396357 |
| intronic | SPATA18 |  |  |  |  |  |  |  |  |  |  |  |  |  | chr4 | 52928353 | 52928353 | T | G | het | . | . | . | 97 | 0 | 0% | T | 34 | 5 | 12.82% | K | Somatic | 0.0015996 |
| intronic | CENPC1 |  |  |  |  |  |  |  |  |  |  |  |  |  | chr4 | 68374834 | 68374834 | C | A | het | . | . | . | 71 | 0 | 0% | C | 16 | 2 | 11.11% | M | Somatic | 0.0390705 |
| exonic | UGT2A2 | nonsynonymous SNV | NM_001105677:c.C554T:p.A185V | 395;Name=lod=54 |  |  |  |  |  | 0 |  |  |  |  | chr4 | 70504805 | 70504805 | G | A | het | . | . | . | 96 | 0 | 0% | G | 24 | 3 | 11.11% | R | Somatic | 0.0096656 |
| exonic | AFM | synonymous SNV | NM_001133:c.T1629A:p.L543L |  |  |  |  |  |  |  |  |  |  |  | chr4 | 74365927 | 74365927 | T | A | het | . | . | . | 78 | 0 | 0% | T | 18 | 2 | 10% | W | Somatic | 0.0399748 |
| intronic | RASSF6 |  |  |  |  |  |  |  |  |  |  |  |  |  | chr4 | 74464452 | 74464452 | A | T | het | . | . | . | 18 | 0 | 0% | A | 9 | 4 | 30.77% | W | Somatic | 0.0227237 |
| exonic | SHROOM3 | nonsynonymous SNV | NM_020859:c.G2721C:p.R907S |  |  |  |  |  |  | 0.04 | 0.994 | 0.98594 | 0.737035 | 0.946713 | chr4 | 77662047 | 77662047 | G | C | het | . | . | . | 53 | 0 | 0% | G | 12 | 2 | 14.29% | S | Somatic | 0.0411578 |
| exonic | BMP2K | nonsynonymous SNV | NM_198892:c.T1975C:p.S659P | 679;Name=lod=767 |  |  |  |  |  | 0.06 |  |  |  |  | chr4 | 79808351 | 79808351 | T | C | het | . | . | . | 50 | 0 | 0% | T | 24 | 3 | 11.11% | Y | Somatic | 0.0399863 |
| exonic | PTPN13 | nonsynonymous SNV | NM_006264:c.G1903A:p.V635M | 647;Name=lod=570 |  |  |  |  |  | 0 |  |  |  |  | chr4 | 87655500 | 87655500 | G | A | het | . | . | . | 51 | 0 | 0% | G | 11 | 2 | 15.38% | R | Somatic | 0.0386905 |
| intronic | AP1AR |  |  |  |  |  |  |  |  |  |  |  |  |  | chr4 | 113178908 | 113178908 | T | C | het | . | . | . | 51 | 0 | 0% | T | 13 | 2 | 13.33% | Y | Somatic | 0.048951 |
| exonic | BCAM | nonsynonymous SNV | NM_001013257:c.C100T:p.R34C |  |  |  |  |  |  | 0.02 | 0.631 | 0.99586 | 0.007048 | 0.863336 | chr19 | 45314499 | 45314499 | C | T | het | . | . | . | 98 | 0 | 0% | C | 135 | 6 | 4.26% | Y | Somatic | 0.0403257 |
| exonic | FAT4 | synonymous SNV | NM_024582:c.T2769G:p.G923G | 469;Name=lod=107 |  |  |  |  |  |  |  |  |  |  | chr4 | 126240335 | 126240335 | T | G | het | . | . | . | 131 | 1 | 0.76% | T | 39 | 5 | 11.36% | K | Somatic | 0.0039714 |
| exonic | LRBA | synonymous SNV | NM_001199282:c.T60G:p.G20G | 522;Name=lod=177 |  |  |  |  |  |  |  |  |  |  | chr4 | 151935735 | 151935735 | A | C | het | . | . | . | 34 | 0 | 0% | A | 25 | 7 | 21.88% | M | Somatic | 0.0043219 |
| exonic | LRBA | synonymous SNV | NM_001199282:c.A57G:p.G19G | 522;Name=lod=177 |  |  |  |  |  |  |  |  |  |  | chr4 | 151935738 | 151935738 | T | C | het | . | . | . | 35 | 0 | 0% | T | 29 | 5 | 14.71% | Y | Somatic | 0.0247591 |
| exonic | DDX60L | nonsynonymous SNV | NM_001012967:c.A4462G:p.N1488D |  |  | 0.43 | 0.43 | 0.43 | rs13110927 | 0.74 |  |  |  |  | chr4 | 169299528 | 169299528 | T | C | het | . | . | . | 32 | 0 | 0% | T | 10 | 3 | 23.08% | Y | Somatic | 0.020155 |
| exonic | SH3RF1 | nonsynonymous SNV | NM_020870:c.T1090G:p.L364V | 573;Name=lod=284 |  |  |  |  |  | 0.07 | 0.251 | 0.979584 | 0.999957 | 1 | chr4 | 170051286 | 170051286 | A | C | het | . | . | . | 40 | 0 | 0% | A | 16 | 4 | 20% | M | Somatic | 0.0099357 |
| intronic | SORBS2 |  |  | 295;Name=lod=21 |  |  |  |  |  |  |  |  |  |  | chr4 | 186556453 | 186556453 | C | A | het | . | . | . | 50 | 0 | 0% | C | 27 | 3 | 10% | M | Somatic | 0.0494158 |
| exonic | FAT1 | nonsynonymous SNV | NM_005245:c.G9597C:p.L3199F | 340;Name=lod=32 |  |  |  |  |  | 0.01 |  |  |  |  | chr4 | 187532796 | 187532796 | C | G | het | . | . | . | 52 | 0 | 0% | C | 10 | 2 | 16.67% | S | Somatic | 0.0327381 |
| exonic | CD86 | nonsynonymous SNV | NM_001206924:c.G592A:p.A198T |  |  | 0.33 | 0.33 | 0.33 | rs1129055 | 0.21 | 0 | 0.990001 | 0.004923 | 0.58267 | chr3 | 121838319 | 121838319 | G | A | het | . | . | . | 181 | 0 | 0% | G | 90 | 4 | 4.26% | R | Somatic | 0.0130805 |
| exonic | DUX2 | synonymous SNV | NM_012147:c.G957C:p.R319R | 462;Name=lod=101 | 1 |  |  |  |  |  |  |  |  |  | chr4 | 190996453 | 190996453 | G | C | het | . | . | . | 23 | 0 | 0% | G | 18 | 4 | 18.18% | S | Somatic | 0.0490956 |
| exonic | DUX2,DUX4L4 | synonymous SNV | NM_001177376:c.A759C:p.A253A | 519;Name=lod=171 | 1 |  |  |  |  |  |  |  |  |  | chr4 | 191012735 | 191012735 | A | C | het | . | . | . | 67 | 1 | 1.47% | A | 23 | 4 | 14.81% | M | Somatic | 0.0219903 |
| splicing | FYB(NM_001465:exon15:c.2072-2A>G,NM_001243093:exon15:c.2102-2A>G,NM_199335:exon14:c.1934-2A>G) |  |  |  |  |  |  |  |  |  |  |  |  |  | chr5 | 39122506 | 39122506 | T | C | het | . | . | . | 52 | 0 | 0% | T | 13 | 2 | 13.33% | Y | Somatic | 0.0474898 |
| intronic | PAIP1 |  |  |  |  |  |  |  |  |  |  |  |  |  | chr5 | 43536871 | 43536871 | T | C | het | . | . | . | 56 | 0 | 0% | T | 11 | 2 | 15.38% | Y | Somatic | 0.0332481 |
| intronic | ITGA2 |  |  |  |  |  |  |  |  |  |  |  |  |  | chr5 | 52358795 | 52358795 | C | T | het | . | . | . | 47 | 0 | 0% | C | 12 | 2 | 14.29% | Y | Somatic | 0.0497268 |
| intronic | ITGA2 |  |  |  |  |  |  |  |  |  |  |  |  |  | chr5 | 52358800 | 52358800 | G | T | het | . | . | . | 44 | 0 | 0% | G | 9 | 2 | 18.18% | K | Somatic | 0.037037 |
| exonic | OTP | nonsynonymous SNV | NM_032109:c.A182G:p.E61G | 578;Name=lod=298 |  |  |  |  |  | 0.14 | 0.004 | 0.998193 | 0.883679 | 0.999953 | chr5 | 76932911 | 76932911 | T | C | het | . | . | . | 91 | 0 | 0% | T | 24 | 3 | 11.11% | Y | Somatic | 0.0109585 |
| intronic | PCSK1 |  |  |  |  |  |  |  |  |  |  |  |  |  | chr5 | 95768514 | 95768514 | T | C | het | . | . | . | 60 | 0 | 0% | T | 43 | 5 | 10.42% | Y | Somatic | 0.0153612 |
| exonic | SHROOM1 | nonsynonymous SNV | NM_133456:c.T1370G:p.V457G |  |  |  |  |  |  | 0 | 0.847 | 0.974592 | 0.924898 | 0.234664 | chr5 | 132159983 | 132159983 | A | C | het | . | . | . | 40 | 0 | 0% | A | 42 | 5 | 10.64% | M | Somatic | 0.0415141 |
| intronic | PLAC8L1 |  |  |  |  | 0.16 | 0.16 | 0.16 | rs12187128 |  |  |  |  |  | chr5 | 145483713 | 145483713 | C | T | het | . | . | . | 61 | 0 | 0% | C | 24 | 3 | 11.11% | Y | Somatic | 0.0266549 |
| exonic | NUP153 | nonsynonymous SNV | NM_005124:c.A3604C:p.T1202P |  |  |  |  |  |  | 0.1 | 0.024 | 0.158114 | 0.044121 | 0.33031 | chr6 | 17626336 | 17626336 | T | G | het | . | . | . | 101 | 2 | 1.94% | T | 38 | 5 | 11.63% | K | Somatic | 0.0235787 |
| intronic | C6orf62 |  |  |  |  |  |  |  |  |  |  |  |  |  | chr6 | 24714693 | 24714693 | A | G | het | . | . | . | 84 | 1 | 1.18% | A | 43 | 5 | 10.42% | R | Somatic | 0.0230169 |
| exonic | ZNF184 | nonsynonymous SNV | NM_007149:c.G1514A:p.S505N |  |  |  |  |  |  | 0.96 | 0 | 0.036055 | 0.005342 | 0.891949 | chr6 | 27419824 | 27419824 | C | T | het | . | . | . | 270 | 4 | 1.46% | C | 76 | 10 | 11.63% | Y | Somatic | 1.65E-04 |
| exonic | OR2J3 | synonymous SNV | NM_001005216:c.G126T:p.L42L |  |  |  |  |  |  |  |  |  |  |  | chr6 | 29079793 | 29079793 | G | T | het | . | . | . | 52 | 0 | 0% | G | 13 | 2 | 13.33% | K | Somatic | 0.0474898 |
| exonic | OR12D3 | nonsynonymous SNV | NM_030959:c.A826T:p.M276L |  |  |  |  |  |  | 0.31 | 0.112 | 0.137395 | 0.135916 | 0.644487 | chr6 | 29342239 | 29342239 | T | A | het | . | . | . | 39 | 0 | 0% | T | 9 | 2 | 18.18% | W | Somatic | 0.044898 |
| exonic | CFB | nonsynonymous SNV | NM_001710:c.G2123A:p.R708K |  |  |  |  |  |  | 0.41 | 0.001 | 0.145536 | 0.213962 | 0.943233 | chr6 | 31919365 | 31919365 | G | A | het | . | . | . | 46 | 0 | 0% | G | 22 | 3 | 12% | R | Somatic | 0.0402414 |
| intronic | RPS18 |  |  |  |  |  |  |  |  |  |  |  |  |  | chr6 | 33240398 | 33240398 | C | T | het | . | . | . | 53 | 0 | 0% | C | 25 | 3 | 10.71% | Y | Somatic | 0.0383966 |
| intronic | CPNE5 |  |  |  |  |  |  |  |  |  |  |  |  |  | chr6 | 36733076 | 36733076 | A | C | het | . | . | . | 43 | 0 | 0% | A | 44 | 5 | 10.20% | M | Somatic | 0.0387758 |
| exonic | FGD2 | nonsynonymous SNV | NM_173558:c.T908G:p.V303G | 480;Name=lod=119 |  |  |  |  |  |  | 0.996 | 0.996344 | 0.790076 | 0.999615 | chr6 | 36982693 | 36982693 | T | G | het | . | . | . | 54 | 1 | 1.82% | T | 124 | 14 | 10.14% | K | Somatic | 0.0396672 |
| intronic | MRPS10 |  |  |  |  |  |  |  |  |  |  |  |  |  | chr6 | 42185475 | 42185475 | T | G | het | . | . | . | 25 | 0 | 0% | T | 56 | 9 | 13.85% | K | Somatic | 0.0452625 |
| intronic | CDC5L |  |  |  |  |  |  |  |  |  |  |  |  |  | chr6 | 44413383 | 44413383 | G | T | het | . | . | . | 119 | 0 | 0% | G | 18 | 2 | 10% | K | Somatic | 0.0198102 |
| intronic | CRISP2 |  |  |  |  |  |  |  |  |  |  |  |  |  | chr6 | 49667510 | 49667510 | T | A | het | . | . | . | 73 | 0 | 0% | T | 10 | 2 | 16.67% | W | Somatic | 0.0184874 |
| exonic | DST | nonsynonymous SNV | NM_015548:c.A5398G:p.S1800G | 539;Name=lod=207 |  |  |  |  |  | 0.09 |  |  |  |  | chr6 | 56437832 | 56437832 | T | C | het | . | . | . | 49 | 0 | 0% | T | 9 | 2 | 18.18% | Y | Somatic | 0.0310734 |
| intronic | DST |  |  | 387;Name=lod=50 |  |  |  |  |  | 0.71 |  |  |  |  | chr6 | 56467691 | 56467691 | C | T | het | . | . | . | 88 | 0 | 0% | C | 18 | 2 | 10% | Y | Somatic | 0.0328834 |
| intronic | DST |  |  | 317;Name=lod=26 |  |  |  |  |  | 0.1 |  |  |  |  | chr6 | 56469027 | 56469027 | C | T | het | . | . | . | 79 | 0 | 0% | C | 26 | 3 | 10.34% | Y | Somatic | 0.0178981 |
| intronic | ZNF451 |  |  |  |  |  |  |  |  |  |  |  |  |  | chr6 | 57006068 | 57006068 | T | C | het | . | . | . | 78 | 0 | 0% | T | 9 | 2 | 18.18% | Y | Somatic | 0.0140449 |
| intronic | COL9A1 |  |  |  |  |  |  |  |  |  |  |  |  |  | chr6 | 70966452 | 70966452 | T | G | het | . | . | . | 48 | 0 | 0% | T | 25 | 3 | 10.71% | K | Somatic | 0.0466003 |
| intronic | CYB5R4 |  |  |  |  |  |  |  |  |  |  |  |  |  | chr6 | 84645899 | 84645899 | G | T | het | . | . | . | 44 | 0 | 0% | G | 11 | 2 | 15.38% | K | Somatic | 0.0488722 |
| intronic | MDN1 |  |  |  |  |  |  |  |  |  |  |  |  |  | chr6 | 90497536 | 90497536 | G | A | het | . | . | . | 81 | 0 | 0% | G | 27 | 3 | 10% | R | Somatic | 0.0183035 |
| intronic | PPIL6 |  |  |  |  |  |  |  |  |  |  |  |  |  | chr6 | 109721417 | 109721417 | A | G | het | . | . | . | 55 | 0 | 0% | A | 12 | 2 | 14.29% | R | Somatic | 0.0387894 |
| exonic | TRDN | nonsynonymous SNV | NM_006073:c.G288T:p.M96I | 589;Name=lod=329 |  |  |  |  |  | 0.03 |  |  |  |  | chr6 | 123869702 | 123869702 | C | A | het | . | . | . | 44 | 0 | 0% | C | 8 | 2 | 20% | M | Somatic | 0.0314465 |
| intronic | HINT3 |  |  |  |  |  |  |  |  |  |  |  |  |  | chr6 | 126296171 | 126296171 | T | A | het | . | . | . | 62 | 0 | 0% | T | 13 | 2 | 13.33% | W | Somatic | 0.0358852 |
| intronic | EPB41L2 |  |  |  |  |  |  |  |  |  |  |  |  |  | chr6 | 131199419 | 131199419 | G | A | het | . | . | . | 52 | 0 | 0% | G | 12 | 2 | 14.29% | R | Somatic | 0.0424242 |
| exonic | OLIG3 | nonsynonymous SNV | NM_175747:c.A545C:p.H182P | 571;Name=lod=278 |  |  |  |  |  | 0.16 | 0.704 | 0.997901 | 0.995905 | 1 | chr6 | 137814763 | 137814763 | T | G | het | . | . | . | 46 | 0 | 0% | T | 32 | 4 | 11.11% | K | Somatic | 0.0336781 |
| exonic | SIAE | nonsynonymous SNV | NM_170601:c.A212G:p.K71R | 358;Name=lod=38 |  | 0.1 | 0.1 | 0.1 | rs12282107 | 0.07 | 0.018 | 0.974357 | 0.635199 | 0.998055 | chr11 | 124539273 | 124539273 | T | C | het | . | . | . | 134 | 0 | 0% | T | 113 | 5 | 4.24% | Y | Somatic | 0.0215012 |
| exonic | SYNJ2 | nonsynonymous SNV | NM_001178088:c.A1678C:p.T560P | 529;Name=lod=189 |  |  |  |  |  | 0.01 | 0.059 | 0.998282 | 0.259544 | 0.999992 | chr6 | 158497754 | 158497754 | A | C | het | . | . | . | 56 | 1 | 1.75% | A | 103 | 17 | 14.17% | M | Somatic | 0.0064156 |
| exonic | LFNG | synonymous SNV | NM_001166355:c.G168A:p.E56E |  |  |  |  |  |  |  |  |  |  |  | chr7 | 2552911 | 2552911 | G | A | het | . | . | . | 58 | 1 | 1.69% | G | 69 | 8 | 10.39% | R | Somatic | 0.0419124 |
| intronic | SLC29A4 |  |  |  | 0.95 |  |  |  |  |  |  |  |  |  | chr7 | 5336543 | 5336543 | C | A | het | . | . | . | 20 | 0 | 0% | C | 9 | 4 | 30.77% | M | Somatic | 0.0174731 |
| exonic | CYP2C18 | nonsynonymous SNV | NM_000772:c.G511T:p.G171C | 470;Name=lod=108 |  |  |  |  |  | 0 | 0.561896 | 0.922559 | 0.071761 | 0.992853 | chr10 | 96454703 | 96454703 | G | T | het | . | . | . | 145 | 0 | 0% | G | 91 | 4 | 4.21% | K | Somatic | 0.023615 |
| exonic | IPO5 | synonymous SNV | NM_002271:c.C2185T:p.L729L | 544;Name=lod=216 |  |  |  |  |  |  |  |  |  |  | chr13 | 98664575 | 98664575 | C | T | het | . | . | . | 172 | 0 | 0% | C | 91 | 4 | 4.21% | Y | Somatic | 0.0153773 |
| UTR3 | ZNF12 |  |  |  |  |  |  |  |  |  |  |  |  |  | chr7 | 6730448 | 6730448 | A | T | het | . | . | . | 60 | 0 | 0% | A | 12 | 2 | 14.29% | W | Somatic | 0.0336912 |
| UTR3 | ZNF12 |  |  |  |  |  |  |  |  |  |  |  |  |  | chr7 | 6730458 | 6730458 | G | A | het | . | . | . | 72 | 0 | 0% | G | 12 | 2 | 14.29% | R | Somatic | 0.0248974 |
| UTR3 | ZNF12 |  |  |  |  |  |  |  |  |  |  |  |  |  | chr7 | 6730464 | 6730464 | A | C | het | . | . | . | 79 | 0 | 0% | A | 16 | 2 | 11.11% | M | Somatic | 0.0328608 |
| UTR3 | ZNF12 |  |  |  |  |  |  |  |  |  |  |  |  |  | chr7 | 6730466 | 6730466 | A | G | het | . | . | . | 82 | 0 | 0% | A | 17 | 2 | 10.53% | R | Somatic | 0.0338614 |
| exonic | BCL10 | synonymous SNV | NM_003921:c.G486A:p.T162T |  |  |  |  |  |  |  |  |  |  |  | chr1 | 85733526 | 85733526 | C | T | het | . | . | . | 202 | 0 | 0% | C | 138 | 6 | 4.17% | Y | Somatic | 0.0048845 |
| exonic | YES1 | stopgain SNV | NM_005433:c.C832T:p.R278X | 650;Name=lod=582 |  |  |  |  |  |  | 0.715559 | 0.87839 | 1 | 0.999999 | chr18 | 743308 | 743308 | G | A | het | . | . | . | 148 | 0 | 0% | G | 115 | 5 | 4.17% | R | Somatic | 0.0171742 |
| intronic | SNX13 |  |  |  |  |  |  |  |  |  |  |  |  |  | chr7 | 17843008 | 17843008 | T | A | het | . | . | . | 57 | 0 | 0% | T | 13 | 2 | 13.33% | W | Somatic | 0.0410798 |
| intronic | DFNA5 |  |  |  |  |  |  |  |  |  |  |  |  |  | chr7 | 24756790 | 24756790 | A | G | het | . | . | . | 20 | 0 | 0% | A | 34 | 8 | 19.05% | R | Somatic | 0.0349088 |
| intronic | HOXA2 |  |  | 389;Name=lod=51 |  |  |  |  | rs73683593 |  |  |  |  |  | chr7 | 27141651 | 27141651 | T | C | het | . | . | . | 34 | 0 | 0% | T | 28 | 4 | 12.50% | Y | Somatic | 0.0498945 |
| exonic | ABCA13 | nonsynonymous SNV | NM_152701:c.A10191C:p.K3397N |  |  |  |  |  |  | 0.03 |  |  |  |  | chr7 | 48378039 | 48378039 | A | C | het | prostate | carcinoma | NS | 40 | 0 | 0% | A | 10 | 3 | 23.08% | M | Somatic | 0.0122087 |
| intronic | PSPH |  |  |  |  | 0.23 | 0.23 | 0.23 | rs77534057 |  |  |  |  |  | chr7 | 56087465 | 56087465 | G | A | het | . | . | . | 19 | 0 | 0% | G | 57 | 13 | 18.57% | R | Somatic | 0.0337593 |
| intronic | PSPH |  |  |  |  | 0.23 | 0.23 | 0.23 | rs77874436 |  |  |  |  |  | chr7 | 56087474 | 56087474 | A | G | het | . | . | . | 20 | 0 | 0% | A | 51 | 11 | 17.74% | R | Somatic | 0.0362985 |
| intergenic | LOC100130849(dist=58420),MIR4283-1(dist=15233) |  |  |  | 0.99 |  |  |  |  |  |  |  |  |  | chr7 | 57008259 | 57008259 | G | C | het | . | . | . | 35 | 0 | 0% | G | 32 | 5 | 13.51% | S | Somatic | 0.0311543 |
| exonic | ZNF107 | nonsynonymous SNV | NM_001013746:c.A1118G:p.E373G |  |  |  |  |  |  | 0.15 | 0.021 | 0.086491 | 0 | 0.587292 | chr7 | 64167800 | 64167800 | A | G | het | . | . | . | 383 | 7 | 1.79% | A | 125 | 19 | 13.19% | R | Somatic | 6.39E-07 |
| exonic | ZNF107 | synonymous SNV | NM_001013746:c.C1596T:p.A532A |  |  |  |  |  |  |  |  |  |  |  | chr7 | 64168278 | 64168278 | C | T | het | . | . | . | 317 | 5 | 1.55% | C | 87 | 10 | 10.31% | Y | Somatic | 3.13E-04 |
| exonic | VKORC1L1 | nonsynonymous SNV | NM_173517:c.T40G:p.W14G | 710;Name=lod=1020 | 0.97 |  |  |  |  | 0 | 0.999 | 0.976987 | 0.006204 | 1 | chr7 | 65338398 | 65338398 | T | G | het | . | . | . | 32 | 0 | 0% | T | 29 | 5 | 14.71% | K | Somatic | 0.0311355 |
| exonic | POR | nonsynonymous SNV | NM_000941:c.A1337C:p.H446P | 632;Name=lod=494 |  |  |  |  |  | 0 | 0.999 | 0.998077 | 0.999998 | 1 | chr7 | 75614464 | 75614464 | A | C | het | . | . | . | 70 | 1 | 1.41% | A | 33 | 4 | 10.81% | M | Somatic | 0.0459777 |
| intergenic | LOC100132832(dist=6108),CCDC146(dist=63471) |  |  |  | 0.98 |  |  |  |  |  |  |  |  |  | chr7 | 76688463 | 76688463 | A | G | het | . | . | . | 50 | 0 | 0% | A | 70 | 8 | 10.26% | R | Somatic | 0.0163998 |
| intergenic | LOC100132832(dist=6201),CCDC146(dist=63378) |  |  |  | 0.98 |  |  |  |  | 0.01 |  |  |  |  | chr7 | 76688556 | 76688556 | T | C | het | . | . | . | 57 | 0 | 0% | T | 159 | 19 | 10.67% | Y | Somatic | 0.0039799 |
| exonic | FZD1 | synonymous SNV | NM_003505:c.C762T:p.G254G | 506;Name=lod=152 |  |  |  |  |  |  |  |  |  |  | chr7 | 90894957 | 90894957 | C | T | het | . | . | . | 44 | 0 | 0% | C | 11 | 2 | 15.38% | Y | Somatic | 0.0488722 |
| intronic | ASB4 |  |  |  |  |  |  |  |  |  |  |  |  |  | chr7 | 95125052 | 95125052 | T | C | het | . | . | . | 29 | 0 | 0% | T | 21 | 4 | 16% | Y | Somatic | 0.0399999 |
| exonic | FBXL6 | synonymous SNV | NM_012162:c.C1053T:p.P351P |  |  |  |  |  |  |  |  |  |  |  | chr8 | 145580132 | 145580132 | G | A | het | . | . | . | 141 | 0 | 0% | G | 139 | 6 | 4.14% | R | Somatic | 0.0161224 |
| exonic | MUC17 | nonsynonymous SNV | NM_001040105:c.G11375T:p.S3792I |  |  |  |  |  |  |  | 0.889 | 0.962678 | 0.001669 | 0.86979 | chr7 | 100686072 | 100686072 | G | T | het | . | . | . | 129 | 2 | 1.53% | G | 47 | 6 | 11.32% | K | Somatic | 0.0077547 |
| intronic | POLR2J |  |  |  | 0.99 |  |  |  |  |  |  |  |  |  | chr7 | 102119224 | 102119224 | A | C | het | . | . | . | 59 | 1 | 1.67% | A | 82 | 20 | 19.61% | M | Somatic | 4.19E-04 |
| intronic | NAMPT |  |  |  |  |  |  |  |  |  |  |  |  |  | chr7 | 105912524 | 105912524 | G | A | het | . | . | . | 26 | 0 | 0% | G | 17 | 4 | 19.05% | R | Somatic | 0.0335548 |
| splicing | DOCK4(NM_014705:exon18:c.1744+1G>T) |  |  | 692;Name=lod=862 |  |  |  |  |  |  |  |  |  |  | chr7 | 111517085 | 111517085 | C | A | het | . | . | . | 70 | 0 | 0% | C | 17 | 2 | 10.53% | M | Somatic | 0.043667 |
| intronic | IQUB |  |  |  |  |  |  |  |  |  |  |  |  |  | chr7 | 123143456 | 123143456 | A | G | het | . | . | . | 69 | 0 | 0% | A | 17 | 2 | 10.53% | R | Somatic | 0.0446708 |
| intronic | FLNC |  |  |  |  |  |  |  |  |  |  |  |  |  | chr7 | 128482767 | 128482767 | A | G | het | . | . | . | 23 | 0 | 0% | A | 11 | 4 | 26.67% | R | Somatic | 0.0184922 |
| intronic | AKR1D1 |  |  |  |  | 0.72 | 0.72 | 0.72 | rs2120846 |  |  |  |  |  | chr7 | 137773291 | 137773291 | T | G | het | . | . | . | 46 | 0 | 0% | T | 30 | 4 | 11.76% | K | Somatic | 0.0293226 |
| intronic | TRIM24 |  |  |  |  |  |  |  |  |  |  |  |  |  | chr7 | 138189190 | 138189190 | C | T | het | . | . | . | 32 | 0 | 0% | C | 21 | 7 | 25% | Y | Somatic | 0.0030658 |
| intergenic | LOC730441(dist=392142),MTRNR2L6(dist=9921) |  |  |  | 0.94 |  |  |  |  |  |  |  |  |  | chr7 | 142364210 | 142364210 | A | C | het | . | . | . | 32 | 0 | 0% | A | 13 | 3 | 18.75% | M | Somatic | 0.0323774 |
| exonic | CLCN1 | nonsynonymous SNV | NM_000083:c.G152C:p.G51A |  |  |  |  |  | rs75643846 | 0.13 | 0.001 | 0.988519 | 0.016364 | 0.650654 | chr7 | 143013457 | 143013457 | G | C | het | . | . | . | 80 | 0 | 0% | G | 255 | 90 | 26.09% | S | Somatic | 5.23E-10 |
| intronic | CNTNAP2 |  |  |  |  |  |  |  |  |  |  |  |  |  | chr7 | 148106459 | 148106459 | A | T | het | . | . | . | 63 | 1 | 1.56% | A | 43 | 6 | 12.24% | W | Somatic | 0.0253976 |
| intronic | ESYT2 |  |  |  |  |  |  |  |  |  |  |  |  |  | chr7 | 158586300 | 158586300 | C | T | het | . | . | . | 56 | 0 | 0% | C | 18 | 3 | 14.29% | Y | Somatic | 0.0181818 |
| exonic | ZNF596 | nonsynonymous SNV | NM_001042415:c.T929A:p.L310H |  |  |  |  |  |  | 0.1 | 0 | 0.130308 | 5.32E-04 | 0.600628 | chr8 | 195776 | 195776 | T | A | het | . | . | . | 125 | 2 | 1.57% | T | 36 | 4 | 10% | W | Somatic | 0.0297549 |
| exonic | ZNF596 | synonymous SNV | NM_001042415:c.G942A:p.K314K | 366;Name=lod=41 |  |  |  |  |  |  |  |  |  |  | chr8 | 195789 | 195789 | G | A | het | . | . | . | 118 | 2 | 1.67% | G | 33 | 4 | 10.81% | R | Somatic | 0.0278655 |
| exonic | SGK223 | nonsynonymous SNV | NM_001080826:c.T1083G:p.S361R |  |  |  |  |  |  | 0.08 | 0.443808 | 0.005212 | 0.018933 | 0.984108 | chr8 | 8234836 | 8234836 | A | C | het | . | . | . | 68 | 1 | 1.45% | A | 44 | 5 | 10.20% | M | Somatic | 0.0441737 |
| intronic | LPL |  |  |  |  |  |  |  |  |  |  |  |  |  | chr8 | 19822769 | 19822769 | G | T | het | . | . | . | 73 | 0 | 0% | G | 31 | 4 | 11.43% | K | Somatic | 0.0097703 |
| exonic | RRS1 | nonsynonymous SNV | NM_015169:c.C424G:p.R142G | 674;Name=lod=731 |  |  |  |  |  | 0.36 | 0.998 | 0.998789 | 0.999458 | 1 | chr8 | 67341790 | 67341790 | C | G | het | . | . | . | 38 | 0 | 0% | C | 38 | 5 | 11.63% | S | Somatic | 0.0375698 |
| exonic | MYBL1 | nonsynonymous SNV | NM_001080416:c.G557A:p.R186K | 714;Name=lod=1065 |  |  |  |  |  | 0 |  |  |  |  | chr8 | 67507948 | 67507948 | C | T | het | . | . | . | 38 | 0 | 0% | C | 9 | 2 | 18.18% | Y | Somatic | 0.0467687 |
| intronic | PREX2 |  |  |  |  |  |  |  |  |  |  |  |  |  | chr8 | 68992850 | 68992850 | T | C | het | . | . | . | 77 | 0 | 0% | T | 16 | 2 | 11.11% | Y | Somatic | 0.0342665 |
| splicing | CNBD1(NM_173538:exon4:c.431+1G>A) |  |  | 366;Name=lod=41 |  |  |  |  |  |  |  |  |  |  | chr8 | 87951983 | 87951983 | G | A | het | . | . | . | 61 | 0 | 0% | G | 13 | 2 | 13.33% | R | Somatic | 0.0368421 |
| intronic | KIAA1429 |  |  |  |  |  |  |  |  |  |  |  |  |  | chr8 | 95547006 | 95547006 | T | A | het | . | . | . | 21 | 0 | 0% | T | 19 | 5 | 20.83% | W | Somatic | 0.0347892 |
| exonic | RGS22 | synonymous SNV | NM_015668:c.T2211A:p.I737I | 560;Name=lod=252 |  |  |  |  |  |  |  |  |  |  | chr8 | 101020753 | 101020753 | A | T | het | . | . | . | 101 | 2 | 1.94% | A | 40 | 5 | 11.11% | W | Somatic | 0.0273274 |
| splicing | PKHD1L1(NM_177531:exon76:c.12331-2A>G) |  |  | 515;Name=lod=165 |  |  |  |  |  |  |  |  |  |  | chr8 | 110535460 | 110535460 | A | G | het | . | . | . | 109 | 1 | 0.91% | A | 21 | 3 | 12.50% | R | Somatic | 0.0181661 |
| exonic;splicing | RAD21;RAD21 | nonsynonymous SNV | NM_006265:c.T815G:p.M272R | 564;Name=lod=262 |  |  |  |  |  | 0.29 | 0.60236 | 0.974533 | 0.977151 | 0.999035 | chr8 | 117868527 | 117868527 | A | C | het | . | . | . | 109 | 2 | 1.80% | A | 46 | 6 | 11.54% | M | Somatic | 0.0134767 |
| intronic | EFR3A |  |  | 559;Name=lod=250 |  |  |  |  |  |  |  |  |  |  | chr8 | 132962209 | 132962209 | T | A | het | . | . | . | 58 | 0 | 0% | T | 15 | 2 | 11.76% | W | Somatic | 0.049009 |
| exonic | EFR3A | nonsynonymous SNV | NM_015137:c.A484G:p.T162A | 622;Name=lod=450 |  |  |  |  |  | 0.28 |  |  |  |  | chr8 | 132962333 | 132962333 | A | G | het | . | . | . | 50 | 0 | 0% | A | 11 | 2 | 15.38% | R | Somatic | 0.0399386 |
| intronic | PTK2 |  |  | 336;Name=lod=31 |  |  |  |  |  |  |  |  |  |  | chr8 | 141780915 | 141780915 | G | A | het | . | . | . | 25 | 0 | 0% | G | 9 | 3 | 25% | R | Somatic | 0.028314 |
| exonic | EPPK1 | nonsynonymous SNV | NM_031308:c.A3332C:p.H1111P |  |  |  |  |  |  |  |  |  |  |  | chr8 | 144944090 | 144944090 | T | G | het | . | . | . | 30 | 0 | 0% | T | 30 | 6 | 16.67% | K | Somatic | 0.0214376 |
| exonic | PLEC | nonsynonymous SNV | NM_201378:c.T3434G:p.V1145G | 459;Name=lod=98 |  |  |  |  |  | 0 | 0.782024 | 0.998441 | 0.978278 | 0.989262 | chr8 | 145001858 | 145001858 | A | C | het | . | . | . | 27 | 0 | 0% | A | 12 | 4 | 25% | M | Somatic | 0.0147476 |
| intronic | PLEC |  |  |  |  |  |  |  |  |  |  |  |  |  | chr8 | 145012298 | 145012298 | A | G | het | . | . | . | 30 | 0 | 0% | A | 23 | 4 | 14.81% | R | Somatic | 0.0444293 |
| exonic | SPATC1 | nonsynonymous SNV | NM_198572:c.T1337G:p.V446G | 536;Name=lod=201 |  |  |  |  |  | 0 | 0.986 | 0.993114 | 0.369269 | 0.99823 | chr8 | 145096163 | 145096163 | T | G | het | . | . | . | 58 | 1 | 1.69% | T | 52 | 9 | 14.75% | K | Somatic | 0.0095921 |
| exonic | OPLAH | nonsynonymous SNV | NM_017570:c.A1787C:p.H596P | 467;Name=lod=105 |  |  |  |  |  | 0.27 |  |  |  |  | chr8 | 145111578 | 145111578 | T | G | het | . | . | . | 53 | 1 | 1.85% | T | 63 | 9 | 12.50% | K | Somatic | 0.0266396 |
| intronic | CPSF1 |  |  |  |  |  |  |  |  |  |  |  |  |  | chr8 | 145626507 | 145626507 | G | A | het | . | . | . | 48 | 0 | 0% | G | 21 | 3 | 12.50% | R | Somatic | 0.033937 |
| exonic | AQP7 | nonsynonymous SNV | NM_001170:c.T343C:p.Y115H | 259;Name=lod=15 | 0.97 | 0.19 | 0.19 | 0.19 | rs74668961 | 0.02 | 0.012 | 0.998419 | 0.999905 | 1 | chr9 | 33386465 | 33386465 | A | G | het | . | . | . | 11 | 0 | 0% | A | 29 | 11 | 27.50% | R | Somatic | 0.0485407 |
| exonic | CA9 | nonsynonymous SNV | NM_001216:c.A543C:p.E181D |  |  |  |  |  |  | 0.02 | 0.033 | 0.123018 | 0.881512 | 0.868791 | chr9 | 35675867 | 35675867 | A | C | het | . | . | . | 37 | 0 | 0% | A | 35 | 5 | 12.50% | M | Somatic | 0.0333037 |
| exonic | FAM75A4,FAM75A5,FAM75A7 | nonsynonymous SNV | NM_001113541:c.G3602A:p.S1201N |  | 1 |  |  |  |  | 0.04 |  |  |  |  | chr9 | 41321702 | 41321702 | C | T | het | . | . | . | 46 | 0 | 0% | C | 16 | 3 | 15.79% | Y | Somatic | 0.0221841 |
| ncRNA_exonic | LOC653501 |  |  |  | 1 |  |  |  | rs35662082 | 0.18 |  |  |  |  | chr9 | 41592344 | 41592344 | A | G | het | . | . | . | 75 | 1 | 1.32% | A | 29 | 4 | 12.12% | R | Somatic | 0.0286511 |
| intronic | CNTNAP3B |  |  |  | 1 |  |  |  |  |  |  |  |  |  | chr9 | 43915911 | 43915911 | G | T | het | . | . | . | 67 | 0 | 0% | G | 15 | 2 | 11.76% | K | Somatic | 0.0390132 |
| exonic | CTSK | nonsynonymous SNV | NM_000396:c.G470A:p.G157D | 584;Name=lod=315 |  |  |  |  |  | 0 | 0.954 | 0.998844 | 0.999997 | 1 | chr1 | 150776645 | 150776645 | C | T | het | . | . | . | 141 | 0 | 0% | C | 93 | 4 | 4.12% | Y | Somatic | 0.0265821 |
| exonic | UBQLN1 | stopgain SNV | NM_013438:c.A184T:p.K62X | 694;Name=lod=885 |  |  |  |  |  | 0 | 0.735243 | 0.998073 | 1 | 1 | chr9 | 86301067 | 86301067 | T | A | het | . | . | . | 52 | 0 | 0% | T | 13 | 2 | 13.33% | W | Somatic | 0.0474898 |
| intronic | NTRK2 |  |  | 676;Name=lod=742 |  |  |  |  |  |  |  |  |  |  | chr9 | 87342549 | 87342549 | C | T | het | . | . | . | 78 | 0 | 0% | C | 18 | 2 | 10% | Y | Somatic | 0.0399748 |
| exonic;splicing | AGTPBP1;AGTPBP1 | synonymous SNV | NM_015239:c.T291G:p.G97G | 456;Name=lod=95 |  |  |  |  |  |  |  |  |  |  | chr9 | 88292496 | 88292496 | A | C | het | . | . | . | 77 | 1 | 1.28% | A | 36 | 4 | 10% | M | Somatic | 0.0445032 |
| intronic | SPTLC1 |  |  |  |  |  |  |  |  |  |  |  |  |  | chr9 | 94809889 | 94809889 | A | C | het | . | . | . | 90 | 0 | 0% | A | 32 | 4 | 11.11% | M | Somatic | 0.0058851 |
| exonic;splicing | ABCA1;ABCA1 | stopgain SNV | NM_005502:c.A5383T:p.K1795X | 665;Name=lod=671 |  |  |  |  |  | 0.29 | 0.735272 | 0.99825 | 1 | 1 | chr9 | 107556791 | 107556791 | T | A | het | . | . | . | 39 | 0 | 0% | T | 22 | 6 | 21.43% | W | Somatic | 0.0037751 |
| intronic | CTNNAL1 |  |  |  |  |  |  |  |  |  |  |  |  |  | chr9 | 111735131 | 111735131 | G | T | het | . | . | . | 40 | 0 | 0% | G | 27 | 4 | 12.90% | K | Somatic | 0.0323836 |
| exonic | SVEP1 | synonymous SNV | NM_153366:c.T7626G:p.G2542G |  |  |  |  |  |  |  |  |  |  |  | chr9 | 113170254 | 113170254 | A | C | het | . | . | . | 67 | 1 | 1.47% | A | 40 | 5 | 11.11% | M | Somatic | 0.0361063 |
| exonic | KIAA0368 | nonsynonymous SNV | NM_001080398:c.A197G:p.E66G | 538;Name=lod=204 |  |  |  |  |  | 0.25 |  |  |  |  | chr9 | 114246716 | 114246716 | T | C | het | . | . | . | 50 | 0 | 0% | T | 23 | 4 | 14.81% | Y | Somatic | 0.0129685 |
| intronic | ASTN2 |  |  |  |  |  |  |  |  |  |  |  |  |  | chr9 | 119903798 | 119903798 | A | C | het | . | . | . | 54 | 1 | 1.82% | A | 36 | 5 | 12.20% | M | Somatic | 0.0493106 |
| exonic | MEGF9 | nonsynonymous SNV | NM_001080497:c.A439C:p.T147P |  |  |  |  |  |  | 0 |  |  |  |  | chr9 | 123476198 | 123476198 | T | G | het | . | . | . | 55 | 1 | 1.79% | T | 33 | 5 | 13.16% | K | Somatic | 0.037913 |
| intronic | DBH |  |  |  |  |  |  |  |  |  |  |  |  |  | chr9 | 136516721 | 136516721 | T | G | het | . | . | . | 50 | 1 | 1.96% | T | 77 | 10 | 11.49% | K | Somatic | 0.0403374 |
| splicing | BRD3(NM_007371:exon9:c.1407+2T>G) |  |  | 492;Name=lod=133 |  |  |  |  |  |  |  |  |  |  | chr9 | 136906880 | 136906880 | A | C | het | . | . | . | 27 | 0 | 0% | A | 32 | 6 | 15.79% | M | Somatic | 0.0334227 |
| exonic | FBXW5 | nonsynonymous SNV | NM_018998:c.C545A:p.S182Y | 374;Name=lod=44 |  |  |  |  |  | 0.03 | 0.984 | 0.998134 | 0.945676 | 0.999973 | chr9 | 139837129 | 139837129 | G | T | het | . | . | . | 66 | 0 | 0% | G | 14 | 2 | 12.50% | K | Somatic | 0.0361337 |
| exonic;splicing | FBXW5;FBXW5 | nonsynonymous SNV | NM_018998:c.A527C:p.D176A | 385;Name=lod=49 |  |  |  |  |  | 0.02 | 0.952 | 0.994884 | 0.992393 | 0.999985 | chr9 | 139837147 | 139837147 | T | G | het | . | . | . | 70 | 0 | 0% | T | 15 | 2 | 11.76% | K | Somatic | 0.0363539 |
| exonic | DPP7 | synonymous SNV | NM_013379:c.G912A:p.T304T |  |  |  |  |  | rs151014148 |  |  |  |  |  | chr9 | 140007216 | 140007216 | C | T | het | . | . | . | 29 | 0 | 0% | C | 21 | 4 | 16% | Y | Somatic | 0.0399999 |
| intronic | CACNA1B |  |  |  |  |  |  |  |  |  |  |  |  |  | chr9 | 140941531 | 140941531 | A | G | het | . | . | . | 17 | 0 | 0% | A | 15 | 5 | 25% | R | Somatic | 0.035568 |
| exonic | TUBB8 | synonymous SNV | NM_177987:c.C751A:p.R251R | 849;Name=lod=3750 | 0.97 |  |  |  |  |  |  |  |  |  | chr10 | 93581 | 93581 | G | T | het | . | . | . | 60 | 1 | 1.64% | G | 66 | 9 | 12% | K | Somatic | 0.0199739 |
| exonic | FBXO18 | nonsynonymous SNV | NM_178150:c.T2470G:p.Y824D | 512;Name=lod=160 |  |  |  |  |  | 0 | 0.999 | 0.998591 | 0.996321 | 1 | chr10 | 5966345 | 5966345 | T | G | het | . | . | . | 58 | 1 | 1.69% | T | 61 | 7 | 10.29% | K | Somatic | 0.0481884 |
| exonic | CACNB2 | synonymous SNV | NM_001167945:c.A39T:p.S13S |  |  |  |  |  |  |  |  |  |  |  | chr10 | 18439814 | 18439814 | A | T | het | . | . | . | 49 | 0 | 0% | A | 12 | 2 | 14.29% | W | Somatic | 0.046595 |
| exonic | ABI1 | synonymous SNV | NM_001178125:c.T660A:p.P220P | 399;Name=lod=56 |  |  |  |  |  |  |  |  |  |  | chr10 | 27040681 | 27040681 | A | T | het | . | . | . | 84 | 1 | 1.18% | A | 38 | 7 | 15.56% | W | Somatic | 0.0025076 |
| exonic | ABI1 | synonymous SNV | NM_001178125:c.G651A:p.P217P | 517;Name=lod=169 |  |  |  |  |  |  |  |  |  |  | chr10 | 27040690 | 27040690 | C | T | het | . | . | . | 74 | 0 | 0% | C | 35 | 4 | 10.26% | Y | Somatic | 0.0127744 |
| exonic | PLA2G4A | synonymous SNV | NM_024420:c.C2109T:p.N703N | 539;Name=lod=206 |  |  |  |  |  |  |  |  |  |  | chr1 | 186948595 | 186948595 | C | T | het | . | . | . | 213 | 0 | 0% | C | 93 | 4 | 4.12% | Y | Somatic | 0.0091809 |
| exonic | ZNF33B | nonsynonymous SNV | NM_006955:c.T1475C:p.I492T | 440;Name=lod=82 | 0.93 |  |  |  |  | 1 | 0.067 | 0.991816 | 0.005892 | 0.67976 | chr10 | 43088923 | 43088923 | A | G | het | . | . | . | 240 | 3 | 1.23% | A | 97 | 12 | 11.01% | R | Somatic | 9.12E-05 |
| intronic | BMS1 |  |  |  | 0.96 |  |  |  |  |  |  |  |  |  | chr10 | 43318525 | 43318525 | T | C | het | . | . | . | 227 | 4 | 1.73% | T | 105 | 13 | 11.02% | Y | Somatic | 2.88E-04 |
| exonic | PNPLA1 | nonsynonymous SNV | NM_001145716:c.T1306C:p.S436P |  |  | 0.64 | 0.64 | 0.64 | rs4713956 |  | 0 | 0.205575 | 4.60E-05 | 0.414396 | chr6 | 36275458 | 36275458 | T | C | het | . | . | . | 181 | 0 | 0% | T | 140 | 6 | 4.11% | Y | Somatic | 0.0074761 |
| exonic | SACS | nonsynonymous SNV | NM_014363:c.C2231T:p.A744V | 624;Name=lod=459 |  |  |  |  |  | 0.05 | 0 | 0.995387 | 0.972569 | 0.999997 | chr13 | 23915784 | 23915784 | G | A | het | . | . | . | 158 | 0 | 0% | G | 71 | 3 | 4.05% | R | Somatic | 0.0315544 |
| intronic | AGAP8,PARG |  |  | 483;Name=lod=123 | 1 |  |  |  |  |  |  |  |  |  | chr10 | 51240690 | 51240690 | C | G | het | . | . | . | 42 | 0 | 0% | C | 42 | 5 | 10.64% | S | Somatic | 0.0369556 |
| intronic | CDH23 |  |  |  |  |  |  |  |  |  |  |  |  |  | chr10 | 73405767 | 73405767 | T | G | het | . | . | . | 25 | 0 | 0% | T | 48 | 8 | 14.29% | K | Somatic | 0.0441638 |
| intronic | LIPJ |  |  |  |  |  |  |  |  |  |  |  |  |  | chr10 | 90356738 | 90356738 | G | C | het | . | . | . | 65 | 0 | 0% | G | 12 | 2 | 14.29% | S | Somatic | 0.0295359 |
| intronic | KIF20B |  |  |  |  | 0.37 | 0.37 | 0.37 | rs3758389 |  |  |  |  |  | chr10 | 91532432 | 91532432 | A | T | het | . | . | . | 68 | 0 | 0% | A | 18 | 2 | 10% | W | Somatic | 0.0496343 |
| exonic | LRRC8E | nonsynonymous SNV | NM_025061:c.C1753T:p.R585W | 538;Name=lod=204 |  |  |  |  |  | 0.01 | 0.936 | 0.99821 | 0.258574 | 0.998961 | chr19 | 7965160 | 7965160 | C | T | het | . | . | . | 116 | 0 | 0% | C | 95 | 4 | 4.04% | Y | Somatic | 0.0434847 |
| exonic | CNNM1 | nonsynonymous SNV | NM_020348:c.A247C:p.T83P |  |  |  |  |  |  | 0.17 |  |  |  |  | chr10 | 101089391 | 101089391 | A | C | het | . | . | . | 35 | 0 | 0% | A | 21 | 4 | 16% | M | Somatic | 0.0259415 |
| exonic | TNPO3 | nonsynonymous SNV | NM_001191028:c.C2068T:p.R690W | 532;Name=lod=193 |  |  |  |  |  | 0 | 0.985 | 0.798177 | 0.999833 | 0.999974 | chr7 | 128614935 | 128614935 | G | A | het | . | . | . | 113 | 0 | 0% | G | 97 | 4 | 3.96% | R | Somatic | 0.0480586 |
| exonic | DNMBP | synonymous SNV | NM_015221:c.A1905C:p.P635P |  |  |  |  |  |  |  |  |  |  |  | chr10 | 101715326 | 101715326 | T | G | het | . | . | . | 32 | 0 | 0% | T | 119 | 16 | 11.85% | K | Somatic | 0.0276917 |
| exonic | PSD | nonsynonymous SNV | NM_002779:c.T2537G:p.V846G | 527;Name=lod=184 |  |  |  |  |  | 0 | 0.969 | 0.975791 | 0.955939 | 0.999958 | chr10 | 104164673 | 104164673 | A | C | het | . | . | . | 30 | 0 | 0% | A | 46 | 10 | 17.86% | M | Somatic | 0.0100559 |
| exonic | EMX2 | nonsynonymous SNV | NM_001165924:c.A230C:p.H77P | 781;Name=lod=1985 |  |  |  |  |  | 0.11 | 0.999 | 0.984189 | 0.754868 | 1 | chr10 | 119303008 | 119303008 | A | C | het | . | . | . | 44 | 0 | 0% | A | 19 | 3 | 13.64% | M | Somatic | 0.0336538 |
| exonic | TACC2 | nonsynonymous SNV | NM_006997:c.G310A:p.E104K | 525;Name=lod=182 |  |  |  |  |  | 0 | 0.98 | 0.999606 | 0.969841 | 0.999085 | chr10 | 123970016 | 123970016 | G | A | het | . | . | . | 34 | 0 | 0% | G | 45 | 6 | 11.76% | R | Somatic | 0.0411783 |
| exonic | C10orf88 | nonsynonymous SNV | NM_024942:c.T391A:p.Y131N | 363;Name=lod=40 |  |  |  |  |  |  | 0.999 | 0.997008 | 0.767446 | 0.956018 | chr10 | 124711520 | 124711520 | A | T | het | . | . | . | 42 | 0 | 0% | A | 10 | 2 | 16.67% | W | Somatic | 0.0461216 |
| exonic | C1orf112 | nonsynonymous SNV | NM_018186:c.C799T:p.L267F | 463;Name=lod=102 |  |  |  |  |  | 0 | 0.687 | 0.979818 | 0.978338 | 0.999978 | chr1 | 169792564 | 169792564 | C | T | het | . | . | . | 208 | 0 | 0% | C | 98 | 4 | 3.92% | Y | Somatic | 0.0112602 |
| intronic | ADAM12 |  |  |  |  |  |  |  |  |  |  |  |  |  | chr10 | 127789601 | 127789601 | A | C | het | . | . | . | 51 | 0 | 0% | A | 39 | 5 | 11.36% | M | Somatic | 0.0187435 |
| exonic | LTBP3 | synonymous SNV | NM_001130144:c.G612A:p.Q204Q | 516;Name=lod=166 |  |  |  |  |  |  |  |  |  |  | chr11 | 65321571 | 65321571 | C | T | het | . | . | . | 122 | 0 | 0% | C | 98 | 4 | 3.92% | Y | Somatic | 0.0416158 |
| intronic | DUX2,DUX4L3,DUX4L5,DUX4L6,DUX4L7 |  |  |  | 1 |  |  |  |  |  |  |  |  |  | chr10 | 135488245 | 135488245 | A | C | het | . | . | . | 68 | 0 | 0% | A | 18 | 2 | 10% | M | Somatic | 0.0496343 |
| exonic | ZNF778 | nonsynonymous SNV | NM_182531:c.G1659T:p.Q553H | 422;Name=lod=69 |  | 0.74 | 0.74 | 0.74 | rs9921361 | 0.03 |  |  |  |  | chr16 | 89294439 | 89294439 | G | T | het | . | . | . | 182 | 0 | 0% | G | 98 | 4 | 3.92% | K | Somatic | 0.0160139 |
| intronic | B4GALNT4 |  |  |  |  |  |  |  |  |  |  |  |  |  | chr11 | 379857 | 379857 | C | T | het | . | . | . | 49 | 0 | 0% | C | 30 | 4 | 11.76% | Y | Somatic | 0.025237 |
| exonic | ROCK2 | nonsynonymous SNV | NM_004850:c.C1835T:p.A612V | 708;Name=lod=1003 |  |  |  |  |  | 0.05 | 0.001 | 0.998958 | 0.509688 | 0.999811 | chr2 | 11355067 | 11355067 | G | A | het | . | . | . | 179 | 0 | 0% | G | 123 | 5 | 3.91% | R | Somatic | 0.0120293 |
| intergenic | MUC2(dist=51072),MUC5B(dist=88806) |  |  |  |  |  |  |  |  |  |  |  |  |  | chr11 | 1155489 | 1155489 | C | G | het | . | . | . | 29 | 0 | 0% | C | 13 | 3 | 18.75% | S | Somatic | 0.0394644 |
| intronic | LMO1 |  |  |  |  |  |  |  |  |  |  |  |  |  | chr11 | 8284852 | 8284852 | T | G | het | . | . | . | 27 | 0 | 0% | T | 18 | 5 | 21.74% | K | Somatic | 0.0158815 |
| intronic | PLEKHA7 |  |  | 667;Name=lod=683 |  |  |  |  |  |  |  |  |  |  | chr11 | 16996317 | 16996317 | T | A | het | . | . | . | 47 | 0 | 0% | T | 8 | 2 | 20% | W | Somatic | 0.0281955 |
| exonic | USH1C | nonsynonymous SNV | NM_153676:c.A1807C:p.I603L | 490;Name=lod=131 |  |  |  |  |  | 0.68 | 0.001 | 0.99831 | 1.12E-04 | 0.829323 | chr11 | 17531109 | 17531109 | T | G | het | . | . | . | 17 | 0 | 0% | T | 40 | 10 | 20% | K | Somatic | 0.0414214 |
| UTR3 | LRRC4C |  |  | 721;Name=lod=1131 |  |  |  |  |  |  |  |  |  |  | chr11 | 40135867 | 40135867 | T | C | het | . | . | . | 101 | 0 | 0% | T | 18 | 3 | 14.29% | Y | Somatic | 0.0045048 |
| UTR3 | LRRC4C |  |  | 721;Name=lod=1131 |  |  |  |  |  |  |  |  |  |  | chr11 | 40135870 | 40135870 | T | A | het | . | . | . | 113 | 0 | 0% | T | 17 | 4 | 19.05% | W | Somatic | 4.66E-04 |
| exonic | OR4S1 | synonymous SNV | NM_001004725:c.G924A:p.E308E |  |  |  |  |  |  |  |  |  |  |  | chr11 | 48328698 | 48328698 | G | A | het | . | . | . | 76 | 0 | 0% | G | 25 | 3 | 10.71% | R | Somatic | 0.0179897 |
| intronic | MS4A13 |  |  |  |  |  |  |  |  |  |  |  |  |  | chr11 | 60285734 | 60285734 | C | A | het | . | . | . | 59 | 0 | 0% | C | 11 | 2 | 15.38% | M | Somatic | 0.0305164 |
| exonic | AHNAK | nonsynonymous SNV | NM_001620:c.A13456G:p.S4486G | 352;Name=lod=36 |  |  |  |  |  | 1 | 0 | 0.908173 | 1.00E-05 | 0.823627 | chr11 | 62288433 | 62288433 | T | C | het | . | . | . | 310 | 3 | 0.96% | T | 94 | 11 | 10.48% | Y | Somatic | 2.97E-05 |
| exonic | PPP2R5B | nonsynonymous SNV | NM_006244:c.A28C:p.T10P | 671;Name=lod=710 |  |  |  |  |  | 0.06 | 0.038 | 0.995269 | 0.048996 | 0.507563 | chr11 | 64693234 | 64693234 | A | C | het | . | . | . | 32 | 0 | 0% | A | 22 | 5 | 18.52% | M | Somatic | 0.0161254 |
| UTR5 | LRFN4 |  |  | 401;Name=lod=57 |  |  |  |  |  |  |  |  |  |  | chr11 | 66625174 | 66625174 | A | C | het | . | . | . | 28 | 0 | 0% | A | 11 | 4 | 26.67% | M | Somatic | 0.0110607 |
| intronic | PITPNM1 |  |  |  |  |  |  |  |  |  |  |  |  |  | chr11 | 67263094 | 67263094 | T | G | het | . | . | . | 23 | 0 | 0% | T | 13 | 4 | 23.53% | K | Somatic | 0.0260422 |
| exonic | AMOTL1 | nonsynonymous SNV | NM_130847:c.A1183C:p.T395P | 511;Name=lod=159 |  |  |  |  |  | 0.18 |  |  |  |  | chr11 | 94554757 | 94554757 | A | C | het | . | . | . | 64 | 1 | 1.54% | A | 38 | 5 | 11.63% | M | Somatic | 0.0358837 |
| intronic | MTMR2 |  |  |  |  |  |  |  |  |  |  |  |  |  | chr11 | 95583953 | 95583953 | C | A | het | . | . | . | 87 | 0 | 0% | C | 14 | 2 | 12.50% | M | Somatic | 0.0228441 |
| intronic | DYNC2H1 |  |  |  |  |  |  |  |  |  |  |  |  |  | chr11 | 103112211 | 103112211 | A | C | het | . | . | . | 72 | 0 | 0% | A | 16 | 2 | 11.11% | M | Somatic | 0.0382022 |
| exonic | OLFML2A | nonsynonymous SNV | NM_182487:c.C1672T:p.L558F | 409;Name=lod=61 |  |  |  |  |  | 0 | 0.993 | 0.999319 | 0.99884 | 1 | chr9 | 127572404 | 127572404 | C | T | het | . | . | . | 115 | 0 | 0% | C | 99 | 4 | 3.88% | Y | Somatic | 0.0483004 |
| intronic | SCN4B |  |  |  |  |  |  |  |  |  |  |  |  |  | chr11 | 118011841 | 118011841 | T | G | het | . | . | . | 61 | 1 | 1.61% | T | 93 | 14 | 13.08% | K | Somatic | 0.0078505 |
| exonic | BCL9L | synonymous SNV | NM_182557:c.A1329C:p.P443P |  |  |  |  |  |  |  |  |  |  |  | chr11 | 118773123 | 118773123 | T | G | het | . | . | . | 65 | 1 | 1.52% | T | 49 | 6 | 10.91% | K | Somatic | 0.0335166 |
| intronic | STT3A |  |  | 696;Name=lod=899 |  |  |  |  |  |  |  |  |  |  | chr11 | 125472846 | 125472846 | G | A | het | . | . | . | 81 | 0 | 0% | G | 18 | 2 | 10% | R | Somatic | 0.0376238 |
| ncRNA_intronic | LOC100288778 |  |  |  | 0.98 |  |  |  | rs2981830 |  |  |  |  |  | chr12 | 88403 | 88403 | T | C | het | . | . | . | 58 | 1 | 1.69% | T | 63 | 9 | 12.50% | Y | Somatic | 0.0192702 |
| ncRNA_exonic | LOC100288778 |  |  | 381;Name=lod=47 | 0.98 |  |  |  |  | 0.36 |  |  |  |  | chr12 | 90810 | 90810 | G | A | het | . | . | . | 27 | 0 | 0% | G | 30 | 11 | 26.83% | R | Somatic | 0.0020609 |
| intronic | DYRK4 |  |  |  |  |  |  |  |  |  |  |  |  |  | chr12 | 4700490 | 4700490 | T | G | het | . | . | . | 49 | 1 | 2% | T | 45 | 7 | 13.46% | K | Somatic | 0.0339349 |
| exonic | USP5 | nonsynonymous SNV | NM_001098536:c.A754C:p.T252P | 493;Name=lod=135 |  |  |  |  |  | 0 | 0.961 | 0.997906 | 0.999995 | 1 | chr12 | 6966040 | 6966040 | A | C | het | . | . | . | 24 | 0 | 0% | A | 59 | 12 | 16.90% | M | Somatic | 0.0234231 |
| exonic | PER3 | synonymous SNV | NM_016831:c.G3450A:p.G1150G |  |  |  |  |  |  |  |  |  |  |  | chr1 | 7897137 | 7897137 | G | A | het | . | . | . | 120 | 0 | 0% | G | 100 | 4 | 3.85% | R | Somatic | 0.0450292 |
| intergenic | SLC2A14(dist=25585),SLC2A3(dist=20744) |  |  |  | 0.94 |  |  |  |  |  |  |  |  |  | chr12 | 8051080 | 8051080 | G | C | het | . | . | . | 34 | 0 | 0% | G | 25 | 6 | 19.35% | S | Somatic | 0.0089139 |
| intronic | PZP |  |  |  |  |  |  |  |  |  |  |  |  |  | chr12 | 9334757 | 9334757 | A | C | het | . | . | . | 35 | 0 | 0% | A | 12 | 3 | 20% | M | Somatic | 0.0232143 |
| intronic | PZP |  |  |  |  | 0.07 | 0.07 | 0.07 | rs12814448 |  |  |  |  |  | chr12 | 9334761 | 9334761 | A | T | het | . | . | . | 30 | 0 | 0% | A | 6 | 7 | 53.85% | W | Somatic | 5.33E-05 |
| ncRNA_exonic | LOC642846 |  |  |  | 0.99 |  |  |  | rs2536755 | 0.29 |  |  |  |  | chr12 | 9447492 | 9447492 | T | C | het | . | . | . | 21 | 0 | 0% | T | 36 | 22 | 37.93% | Y | Somatic | 2.86E-04 |
| ncRNA_intronic | DDX12P |  |  |  | 0.99 |  |  |  |  |  |  |  |  |  | chr12 | 9580493 | 9580493 | G | C | het | . | . | . | 28 | 0 | 0% | G | 13 | 4 | 23.53% | S | Somatic | 0.0159737 |
| intronic | GSG1 |  |  |  |  |  |  |  |  |  |  |  |  |  | chr12 | 13248457 | 13248457 | T | G | het | . | . | . | 50 | 1 | 1.96% | T | 54 | 8 | 12.90% | K | Somatic | 0.0322816 |
| intronic | PYROXD1 |  |  |  |  | 0.38 | 0.38 | 0.38 | rs1963817 |  |  |  |  |  | chr12 | 21598264 | 21598264 | C | T | het | . | . | . | 59 | 0 | 0% | C | 20 | 3 | 13.04% | Y | Somatic | 0.0199977 |
| intronic | GOLT1B |  |  |  |  |  |  |  |  |  |  |  |  |  | chr12 | 21661283 | 21661283 | A | G | het | . | . | . | 61 | 0 | 0% | A | 14 | 2 | 12.50% | R | Somatic | 0.0410116 |
| intronic | ABCC9 |  |  |  |  |  |  |  |  |  |  |  |  |  | chr12 | 22063269 | 22063269 | T | A | het | . | . | . | 105 | 1 | 0.94% | T | 36 | 4 | 10% | W | Somatic | 0.0200548 |
| intronic | FGD4 |  |  |  |  |  |  |  |  |  |  |  |  |  | chr12 | 32735435 | 32735435 | A | C | het | . | . | . | 62 | 1 | 1.59% | A | 25 | 4 | 13.79% | M | Somatic | 0.0328419 |
| intronic | GXYLT1 |  |  |  |  |  |  |  |  |  |  |  |  |  | chr12 | 42499920 | 42499920 | G | A | het | . | . | . | 47 | 0 | 0% | G | 7 | 3 | 30% | R | Somatic | 0.0041012 |
| intronic | NELL2 |  |  |  |  | 0.19 | 0.19 | 0.19 | rs2290353 |  |  |  |  |  | chr12 | 45059237 | 45059237 | T | G | het | . | . | . | 46 | 0 | 0% | T | 11 | 3 | 21.43% | K | Somatic | 0.0106371 |
| intronic | CACNB3 |  |  |  |  |  |  |  |  |  |  |  |  |  | chr12 | 49217068 | 49217068 | T | G | het | . | . | . | 41 | 0 | 0% | T | 51 | 11 | 17.74% | K | Somatic | 0.0025483 |
| UTR3 | ARF3 |  |  |  |  |  |  |  |  |  |  |  |  |  | chr12 | 49332706 | 49332706 | T | G | het | . | . | . | 23 | 0 | 0% | T | 37 | 7 | 15.91% | K | Somatic | 0.0440644 |
| intronic | NACA |  |  |  |  |  |  |  |  |  |  |  |  |  | chr12 | 57112284 | 57112284 | T | G | het | . | . | . | 44 | 0 | 0% | T | 47 | 6 | 11.32% | K | Somatic | 0.0232323 |
| exonic | OTOGL | synonymous SNV | NM_173591:c.C6063T:p.S2021S | 534;Name=lod=198 |  |  |  |  |  |  |  |  |  |  | chr12 | 80752098 | 80752098 | C | T | het | . | . | . | 49 | 0 | 0% | C | 12 | 2 | 14.29% | Y | Somatic | 0.046595 |
| intronic | POC1B |  |  |  |  |  |  |  |  |  |  |  |  |  | chr12 | 89853406 | 89853406 | C | A | het | . | . | . | 35 | 0 | 0% | C | 40 | 7 | 14.89% | M | Somatic | 0.0165427 |
| intronic | NEDD1 |  |  | 446;Name=lod=87 |  |  |  |  |  |  |  |  |  |  | chr12 | 97339432 | 97339432 | C | T | het | . | . | . | 59 | 0 | 0% | C | 20 | 3 | 13.04% | Y | Somatic | 0.0199977 |
| intronic | NOS1 |  |  |  |  |  |  |  |  |  |  |  |  |  | chr12 | 117696190 | 117696190 | T | G | het | . | . | . | 32 | 0 | 0% | T | 35 | 5 | 12.50% | K | Somatic | 0.047029 |
| intronic | ZCCHC8 |  |  |  |  |  |  |  |  |  |  |  |  |  | chr12 | 122974017 | 122974017 | C | A | het | . | . | . | 55 | 0 | 0% | C | 29 | 4 | 12.12% | M | Somatic | 0.017548 |
| intronic | PSPC1 |  |  | 673;Name=lod=724 | 0.93 |  |  |  |  |  |  |  |  |  | chr13 | 20325613 | 20325613 | T | G | het | . | . | . | 51 | 0 | 0% | T | 13 | 2 | 13.33% | K | Somatic | 0.048951 |
| exonic | RNF17 | nonsynonymous SNV | NM_001184993:c.G3754A:p.A1252T |  |  |  |  |  |  | 0.59 | 0 | 0.820125 | 1.88E-04 | 0.913675 | chr13 | 25433294 | 25433294 | G | A | het | . | . | . | 108 | 0 | 0% | G | 27 | 3 | 10% | R | Somatic | 0.0094741 |
| exonic | SLC30A7 | nonsynonymous SNV | NM_001144884:c.G199A:p.D67N | 698;Name=lod=912 |  |  |  |  |  | 0 | 0.998 | 0.999675 | 0.999985 | 1 | chr1 | 101372424 | 101372424 | G | A | het | . | . | . | 192 | 0 | 0% | G | 102 | 4 | 3.77% | R | Somatic | 0.0154269 |
| intronic | MTIF3 |  |  |  |  |  |  |  |  |  |  |  |  |  | chr13 | 28011447 | 28011447 | T | A | het | . | . | . | 49 | 0 | 0% | T | 20 | 3 | 13.04% | W | Somatic | 0.0296948 |
| intronic | KATNAL1 |  |  |  |  |  |  |  |  |  |  |  |  |  | chr13 | 30829758 | 30829758 | A | C | het | . | . | . | 76 | 0 | 0% | A | 15 | 2 | 11.76% | M | Somatic | 0.0317906 |
| intronic | USPL1 |  |  |  |  |  |  |  |  |  |  |  |  |  | chr13 | 31195845 | 31195845 | T | A | het | . | . | . | 59 | 0 | 0% | T | 12 | 2 | 14.29% | W | Somatic | 0.0346271 |
| exonic | KL | nonsynonymous SNV | NM_004795:c.A280C:p.T94P | 660;Name=lod=641 |  |  |  |  |  | 0.02 | 0.994 | 0.994679 | 0.814584 | 0.99929 | chr13 | 33590858 | 33590858 | A | C | het | . | . | . | 57 | 0 | 0% | A | 34 | 4 | 10.53% | M | Somatic | 0.0231864 |
| intronic | KPNA3 |  |  |  |  |  |  |  |  |  |  |  |  |  | chr13 | 50299649 | 50299649 | C | A | het | . | . | . | 51 | 0 | 0% | C | 17 | 5 | 22.73% | M | Somatic | 0.0017532 |
| intronic | DIAPH3 |  |  |  |  |  |  |  |  |  |  |  |  |  | chr13 | 60557857 | 60557857 | G | T | het | . | . | . | 86 | 0 | 0% | G | 13 | 2 | 13.33% | K | Somatic | 0.0207921 |
| splicing | DIAPH3(NM_001042517:exon5:c.495+1G>T) |  |  | 695;Name=lod=892 |  |  |  |  |  |  |  |  |  |  | chr13 | 60667761 | 60667761 | C | A | het | . | . | . | 158 | 0 | 0% | C | 16 | 2 | 11.11% | M | Somatic | 0.0099351 |
| intronic | TGDS |  |  |  |  |  |  |  |  |  |  |  |  |  | chr13 | 95227111 | 95227111 | T | A | het | . | . | . | 72 | 0 | 0% | T | 24 | 3 | 11.11% | W | Somatic | 0.0186485 |
| intronic | NALCN |  |  |  |  |  |  |  |  | 0.01 |  |  |  |  | chr13 | 101769334 | 101769334 | G | A | het | . | . | . | 68 | 0 | 0% | G | 15 | 2 | 11.76% | R | Somatic | 0.0380952 |
| exonic | RPGRIP1 | nonsynonymous SNV | NM_020366:c.T1396C:p.S466P |  |  |  |  |  |  | 0.23 |  |  |  |  | chr14 | 21788265 | 21788265 | T | C | het | . | . | . | 58 | 0 | 0% | T | 49 | 6 | 10.91% | Y | Somatic | 0.011474 |
| intergenic | OR4E2(dist=457260),DAD1(dist=442309) |  |  |  |  |  |  |  |  | 0 |  |  |  |  | chr14 | 22591498 | 22591498 | C | A | het | . | . | . | 73 | 0 | 0% | C | 15 | 2 | 11.76% | M | Somatic | 0.0339576 |
| intronic | RCOR3 |  |  |  |  | 0.46 | 0.46 | 0.46 | rs3767377 |  |  |  |  |  | chr1 | 211452678 | 211452678 | G | A | het | . | . | . | 145 | 0 | 0% | G | 102 | 4 | 3.77% | R | Somatic | 0.0307681 |
| exonic | ACIN1 | synonymous SNV | NM_001164816:c.A1179C:p.P393P |  |  |  |  |  |  |  |  |  |  |  | chr14 | 23530745 | 23530745 | T | G | het | . | . | . | 64 | 1 | 1.54% | T | 60 | 7 | 10.45% | K | Somatic | 0.0342397 |
| exonic | SOX1 | synonymous SNV | NM_005986:c.G192A:p.G64G | 845;Name=lod=3606 |  |  |  |  |  |  |  |  |  |  | chr13 | 112722164 | 112722164 | G | A | het | . | . | . | 192 | 0 | 0% | G | 128 | 5 | 3.76% | R | Somatic | 0.0109708 |
| intronic | TGM1 |  |  |  |  |  |  |  |  |  |  |  |  |  | chr14 | 24729124 | 24729124 | A | C | het | . | . | . | 35 | 0 | 0% | A | 67 | 9 | 11.84% | M | Somatic | 0.0281943 |
| exonic | LTB4R2 | nonsynonymous SNV | NM_001164692:c.C145T:p.R49W | 440;Name=lod=82 |  |  |  |  |  | 0 | 0.998 | 0.974298 | 0.487438 | 0.893157 | chr14 | 24780015 | 24780015 | C | T | het | . | . | . | 69 | 0 | 0% | C | 27 | 3 | 10% | Y | Somatic | 0.0258848 |
| exonic | INSM2 | nonsynonymous SNV | NM_032594:c.C1517G:p.A506G | 259;Name=lod=15 |  |  |  |  |  | 0.03 | 0.003 | 0.998571 | 0.201517 | 0.996692 | chr14 | 36004975 | 36004975 | C | G | het | . | . | . | 114 | 2 | 1.72% | C | 46 | 7 | 13.21% | S | Somatic | 0.0045502 |
| intronic | PYGL |  |  |  |  |  |  |  |  |  |  |  |  |  | chr14 | 51375697 | 51375697 | G | A | het | . | . | . | 72 | 0 | 0% | G | 16 | 2 | 11.11% | R | Somatic | 0.0382022 |
| intronic | SLC35F4 |  |  |  |  | 0.23 | 0.23 | 0.23 | rs17724076 |  |  |  |  |  | chr14 | 58060862 | 58060862 | G | A | het | . | . | . | 47 | 0 | 0% | G | 21 | 4 | 16% | R | Somatic | 0.012296 |
| exonic | SIX6 | nonsynonymous SNV | NM_007374:c.A533C:p.N178T | 690;Name=lod=849 |  |  |  |  | rs78978726 | 0.02 | 0.214 | 0.999183 | 0.999988 | 1 | chr14 | 60976649 | 60976649 | A | C | het | . | . | . | 40 | 0 | 0% | A | 33 | 5 | 13.16% | M | Somatic | 0.0237762 |
| exonic | SIX4 | nonsynonymous SNV | NM_017420:c.G187T:p.A63S | 304;Name=lod=23 |  |  |  |  |  | 0.24 | 0 | 0.032581 | 0 | 0.331152 | chr14 | 61190606 | 61190606 | C | A | het | . | . | . | 70 | 0 | 0% | C | 14 | 2 | 12.50% | M | Somatic | 0.0328317 |
| intronic | SYNE2 |  |  |  |  |  |  |  |  |  |  |  |  |  | chr14 | 64634388 | 64634388 | C | T | het | . | . | . | 66 | 0 | 0% | C | 24 | 3 | 11.11% | Y | Somatic | 0.0225406 |
| UTR3 | SPTB |  |  |  |  |  |  |  |  |  |  |  |  |  | chr14 | 65216006 | 65216006 | G | C | het | . | . | . | 24 | 0 | 0% | G | 10 | 5 | 33.33% | S | Somatic | 0.0052157 |
| intronic | MAX |  |  | 676;Name=lod=742 |  |  |  |  |  |  |  |  |  |  | chr14 | 65568308 | 65568308 | A | G | het | . | . | . | 42 | 0 | 0% | A | 10 | 2 | 16.67% | R | Somatic | 0.0461216 |
| intronic | SIPA1L1 |  |  | 243;Name=lod=13 |  |  |  |  |  |  |  |  |  |  | chr14 | 72125041 | 72125041 | T | A | het | . | . | . | 91 | 0 | 0% | T | 26 | 3 | 10.34% | W | Somatic | 0.013011 |
| intronic | CCDC88C |  |  |  |  |  |  |  |  |  |  |  |  |  | chr14 | 91766430 | 91766430 | A | C | het | . | . | . | 29 | 0 | 0% | A | 18 | 4 | 18.18% | M | Somatic | 0.0292717 |
| intronic | CATSPERB |  |  |  |  |  |  |  |  |  |  |  |  |  | chr14 | 92126120 | 92126120 | C | T | het | . | . | . | 72 | 1 | 1.37% | C | 29 | 4 | 12.12% | Y | Somatic | 0.0318183 |
| upstream;downstream | SNORD114-4;SNORD114-3 |  |  |  |  |  |  |  |  |  |  |  |  |  | chr14 | 101420666 | 101420666 | C | T | het | . | . | . | 72 | 0 | 0% | C | 18 | 2 | 10% | Y | Somatic | 0.0453894 |
| exonic | CYFIP2 | nonsynonymous SNV | NM_001037332:c.C3656T:p.A1219V | 633;Name=lod=499 |  |  |  |  |  | 0.15 |  |  |  |  | chr5 | 156819902 | 156819902 | C | T | het | . | . | . | 161 | 0 | 0% | C | 129 | 5 | 3.73% | Y | Somatic | 0.0185538 |
| exonic | AHNAK2 | nonsynonymous SNV | NM_138420:c.G17093A:p.R5698Q | 385;Name=lod=49 |  |  |  |  |  | 0 |  |  |  |  | chr14 | 105404695 | 105404695 | C | T | het | . | . | . | 53 | 0 | 0% | C | 26 | 3 | 10.34% | Y | Somatic | 0.0412602 |
| exonic | MTA1 | nonsynonymous SNV | NM_004689:c.A1631C:p.H544P | 460;Name=lod=99 |  |  |  |  |  | 0.14 | 0.979 | 0.997494 | 0.929241 | 0.99999 | chr14 | 105932769 | 105932769 | A | C | het | . | . | . | 17 | 0 | 0% | A | 11 | 4 | 26.67% | M | Somatic | 0.0379588 |
| exonic | C14orf80 | nonsynonymous SNV | NM_001134876:c.A1028G:p.D343G |  |  |  |  |  |  | 0.69 |  |  |  |  | chr14 | 105965129 | 105965129 | A | G | het | . | . | . | 34 | 0 | 0% | A | 15 | 3 | 16.67% | R | Somatic | 0.0369231 |
| exonic | C15orf23 | nonsynonymous SNV | NM_001142761:c.T26C:p.L9P |  |  |  |  |  |  | 0.34 | 0 | 0.003656 | 0.001488 | 0.009296 | chr15 | 40675062 | 40675062 | T | C | het | . | . | . | 43 | 0 | 0% | T | 41 | 5 | 10.87% | Y | Somatic | 0.0330241 |
| exonic | SPG11 | nonsynonymous SNV | NM_001160227:c.G6611C:p.G2204A |  |  |  |  |  | rs79186522 | 0.34 | 0.006 | 0.95023 | 0.07104 | 0.92384 | chr15 | 44858101 | 44858101 | C | G | het | . | . | . | 32 | 0 | 0% | C | 18 | 4 | 18.18% | S | Somatic | 0.0231304 |
| exonic | CD276 | nonsynonymous SNV | NM_001024736:c.G1048A:p.A350T | 540;Name=lod=209 |  |  |  |  |  | 0.02 | 0.985 | 0.998798 | 0.962077 | 1 | chr15 | 73996314 | 73996314 | G | A | het | . | . | . | 55 | 0 | 0% | G | 26 | 4 | 13.33% | R | Somatic | 0.0135348 |
| intergenic | LOC727849(dist=7500),RPS17L(dist=15309) |  |  |  | 1 |  |  |  | rs1267670 | 0.18 |  |  |  |  | chr15 | 82805852 | 82805852 | C | T | het | . | . | . | 24 | 0 | 0% | C | 23 | 7 | 23.33% | Y | Somatic | 0.0114952 |
| intronic | AP3B2 |  |  |  |  |  |  |  |  |  |  |  |  |  | chr15 | 83330685 | 83330685 | T | G | het | . | . | . | 29 | 0 | 0% | T | 30 | 7 | 18.92% | K | Somatic | 0.0132198 |
| UTR3 | TCP1 |  |  |  |  | 0.63 | 0.63 | 0.63 | rs15982 |  |  |  |  |  | chr6 | 160199687 | 160199687 | T | C | het | . | . | . | 213 | 0 | 0% | T | 129 | 5 | 3.73% | Y | Somatic | 0.0081972 |
| ncRNA_intronic | GOLGA6L5 |  |  |  | 0.98 |  |  |  |  |  |  |  |  |  | chr15 | 85057542 | 85057542 | C | T | het | . | . | . | 38 | 0 | 0% | C | 40 | 7 | 14.89% | Y | Somatic | 0.0127418 |
| intronic | WDR90 |  |  |  |  |  |  |  |  |  |  |  |  |  | chr16 | 711279 | 711279 | A | C | het | . | . | . | 46 | 0 | 0% | A | 20 | 3 | 13.04% | M | Somatic | 0.0338016 |
| exonic | FLYWCH1 | nonsynonymous SNV | NM_020912:c.A1319G:p.E440G | 469;Name=lod=107 |  |  |  |  |  | 0.02 |  |  |  |  | chr16 | 2983789 | 2983789 | A | G | het | . | . | . | 29 | 0 | 0% | A | 25 | 5 | 16.67% | R | Somatic | 0.0284648 |
| intronic | RBFOX1 |  |  | 660;Name=lod=639 |  |  |  |  |  |  |  |  |  |  | chr16 | 7721510 | 7721510 | A | G | het | . | . | . | 83 | 0 | 0% | A | 25 | 3 | 10.71% | R | Somatic | 0.0147691 |
| exonic | CLEC16A | nonsynonymous SNV | NM_001243403:c.C619T:p.H207Y | 583;Name=lod=311 |  |  |  |  |  | 0.07 |  |  |  |  | chr16 | 11066815 | 11066815 | C | T | het | . | . | . | 65 | 0 | 0% | C | 27 | 4 | 12.90% | Y | Somatic | 0.0094718 |
| ncRNA_intronic | PKD1P1 |  |  |  | 0.99 |  |  |  |  |  |  |  |  |  | chr16 | 16411944 | 16411944 | C | T | het | . | . | . | 23 | 0 | 0% | C | 10 | 4 | 28.57% | Y | Somatic | 0.0151563 |
| exonic | SMG1 | nonsynonymous SNV | NM_015092:c.G783T:p.E261D | 747;Name=lod=1451 | 0.97 |  |  |  |  | 0.54 |  |  |  |  | chr16 | 18900733 | 18900733 | C | A | het | . | . | . | 43 | 0 | 0% | C | 9 | 2 | 18.18% | M | Somatic | 0.0384347 |
| exonic | TNRC6A | nonsynonymous SNV | NM_014494:c.T2410G:p.W804G | 587;Name=lod=325 |  |  |  |  |  | 0 | 0.999 | 0.998193 | 0.791295 | 1 | chr16 | 24802373 | 24802373 | T | G | het | . | . | . | 55 | 1 | 1.79% | T | 26 | 5 | 16.13% | K | Somatic | 0.0203003 |
| exonic | RABEP2 | nonsynonymous SNV | NM_024816:c.T1406G:p.V469G | 519;Name=lod=171 |  |  |  |  |  | 0.01 | 0 | 0.961188 | 0.391206 | 0.810164 | chr16 | 28917357 | 28917357 | A | C | het | . | . | . | 36 | 0 | 0% | A | 12 | 3 | 20% | M | Somatic | 0.0218487 |
| exonic | FUS | nonsynonymous SNV | NM_001170634:c.A1565G:p.E522G | 505;Name=lod=150 |  |  |  |  |  | 0.13 | 0.831 | 0.996674 | 0.176814 | 0.999867 | chr16 | 31202746 | 31202746 | A | G | het | . | . | . | 78 | 0 | 0% | A | 29 | 4 | 12.12% | R | Somatic | 0.0068325 |
| exonic | ARMC5 | nonsynonymous SNV | NM_001105247:c.T1799G:p.V600G |  |  |  |  |  |  | 0.02 | 0.881 | 0.997447 | 0.372135 | 0.982127 | chr16 | 31476143 | 31476143 | T | G | het | . | . | . | 26 | 0 | 0% | T | 19 | 4 | 17.39% | K | Somatic | 0.0417933 |
| exonic | RGS7 | nonsynonymous SNV | NM_002924:c.G314A:p.G105D | 648;Name=lod=572 |  |  |  |  |  | 0.01 | 0.251 | 0.998784 | 0.999576 | 1 | chr1 | 241099919 | 241099919 | C | T | het | . | . | . | 203 | 0 | 0% | C | 105 | 4 | 3.67% | Y | Somatic | 0.014365 |
| exonic | VPS4A | nonsynonymous SNV | NM_013245:c.T626G:p.V209G | 623;Name=lod=452 |  |  |  |  |  | 0 |  |  |  |  | chr16 | 69354049 | 69354049 | T | G | het | . | . | . | 47 | 0 | 0% | T | 43 | 5 | 10.42% | K | Somatic | 0.0295528 |
| ncRNA_exonic | PDXDC2P |  |  | 493;Name=lod=134 | 0.98 |  |  |  | rs71242416 |  |  |  |  |  | chr16 | 70048023 | 70048023 | T | C | het | . | . | . | 49 | 0 | 0% | T | 27 | 4 | 12.90% | Y | Somatic | 0.0198947 |
| intronic | CLEC18C |  |  |  | 0.99 |  |  |  | rs2650540 |  |  |  |  |  | chr16 | 70208347 | 70208347 | T | C | het | . | . | . | 52 | 1 | 1.89% | T | 39 | 6 | 13.33% | Y | Somatic | 0.0344841 |
| exonic | STARD13 | synonymous SNV | NM_001243466:c.G1161A:p.V387V | 577;Name=lod=296 |  |  |  |  |  |  |  |  |  |  | chr13 | 33703629 | 33703629 | C | T | het | . | . | . | 187 | 0 | 0% | C | 105 | 4 | 3.67% | Y | Somatic | 0.0177505 |
| exonic | STEAP4 | nonsynonymous SNV | NM_024636:c.C709T:p.R237C | 284;Name=lod=19 |  |  |  |  |  | 0 | 0.963 | 0.993705 | 0.988447 | 0.999943 | chr7 | 87912231 | 87912231 | G | A | het | . | . | . | 205 | 0 | 0% | G | 105 | 4 | 3.67% | R | Somatic | 0.0140007 |
| exonic | ZNF19 | synonymous SNV | NM_006961:c.G663A:p.E221E |  |  |  |  |  |  |  |  |  |  |  | chr16 | 71509787 | 71509787 | C | T | het | . | . | . | 110 | 1 | 0.90% | C | 36 | 4 | 10% | Y | Somatic | 0.0176548 |
| exonic | ZFHX3 | nonsynonymous SNV | NM_001164766:c.T4771C:p.S1591P | 540;Name=lod=209 |  |  |  |  |  |  | 0.722404 | 0.998588 | 0.957652 | 0.997898 | chr16 | 72829068 | 72829068 | A | G | het | . | . | . | 65 | 1 | 1.52% | A | 94 | 11 | 10.48% | R | Somatic | 0.0209219 |
| exonic | ZC3H18 | nonsynonymous SNV | NM_144604:c.T128G:p.V43G |  |  |  |  |  |  | 0.19 | 0.505941 | 0.163294 | 0.336344 | 0.947904 | chr16 | 88643659 | 88643659 | T | G | het | . | . | . | 36 | 0 | 0% | T | 61 | 10 | 14.08% | K | Somatic | 0.0131409 |
| exonic | ZNF778 | synonymous SNV | NM_182531:c.C1935T:p.Y645Y |  |  |  |  |  |  |  |  |  |  |  | chr16 | 89294715 | 89294715 | C | T | het | . | . | . | 82 | 0 | 0% | C | 45 | 5 | 10% | Y | Somatic | 0.0068498 |
| exonic | ZNF778 | nonsynonymous SNV | NM_182531:c.T1937A:p.I646K |  |  |  |  |  |  | 0.4 |  |  |  |  | chr16 | 89294717 | 89294717 | T | A | het | . | . | . | 77 | 1 | 1.28% | T | 45 | 6 | 11.76% | W | Somatic | 0.0152128 |
| exonic | NXN | nonsynonymous SNV | NM_001205319:c.T563G:p.V188G | 599;Name=lod=361 |  |  |  |  |  | 0 | 0.002 | 0.998185 | 0.999975 | 0.999897 | chr17 | 708421 | 708421 | A | C | het | . | . | . | 24 | 0 | 0% | A | 35 | 7 | 16.67% | M | Somatic | 0.0346414 |
| intronic | SLC13A5 |  |  |  |  |  |  |  |  |  |  |  |  |  | chr17 | 6596495 | 6596495 | G | A | het | . | . | . | 22 | 0 | 0% | G | 35 | 7 | 16.67% | R | Somatic | 0.0434282 |
| exonic | IGSF1 | nonsynonymous SNV | NM_001170962:c.C1009T:p.P337S |  |  |  |  |  |  | 0.18 | 0.87 | 0.121891 | 0.067538 | 0.300335 | chrX | 130416628 | 130416628 | G | A | het | . | . | . | 142 | 0 | 0% | G | 105 | 4 | 3.67% | R | Somatic | 0.0344568 |
| intronic | ARHGEF15 |  |  |  |  |  |  |  |  |  |  |  |  |  | chr17 | 8215973 | 8215973 | T | G | het | . | . | . | 25 | 0 | 0% | T | 39 | 7 | 15.22% | K | Somatic | 0.0402474 |
| exonic | MYH3 | nonsynonymous SNV | NM_002470:c.T4009C:p.S1337P | 531;Name=lod=192 |  |  |  |  |  |  | 0.157 | 0.923269 | 0.089836 | 0.839345 | chr17 | 10538847 | 10538847 | A | G | het | . | . | . | 89 | 1 | 1.11% | A | 220 | 32 | 12.70% | R | Somatic | 3.36E-04 |
| exonic | RNF19B | nonsynonymous SNV | NM_001127361:c.G950T:p.C317F | 645;Name=lod=555 |  |  |  |  |  | 0 | 1 | 0.999294 | 0.999185 | 1 | chr1 | 33413856 | 33413856 | C | A | het | . | . | . | 153 | 0 | 0% | C | 106 | 4 | 3.64% | M | Somatic | 0.0296316 |
| intronic | SMCR8 |  |  |  |  |  |  |  |  |  |  |  |  |  | chr17 | 18221479 | 18221479 | T | G | het | . | . | . | 67 | 1 | 1.47% | T | 71 | 8 | 10.13% | K | Somatic | 0.0287745 |
| exonic | PPFIBP1 | nonsynonymous SNV | NM_001198915:c.G418A:p.G140R | 475;Name=lod=114 | 0.93 |  |  |  |  | 0.03 | 0.289 | 0.990251 | 0.182481 | 0.997509 | chr12 | 27809636 | 27809636 | G | A | het | . | . | . | 190 | 0 | 0% | G | 106 | 4 | 3.64% | R | Somatic | 0.0174527 |
| intronic | RAB11FIP4 |  |  |  |  |  |  |  |  |  |  |  |  |  | chr17 | 29858586 | 29858586 | A | C | het | . | . | . | 46 | 0 | 0% | A | 75 | 10 | 11.76% | M | Somatic | 0.0108494 |
| ncRNA_exonic | MIR193A |  |  | 550;Name=lod=230 |  |  |  |  |  |  |  |  |  |  | chr17 | 29887039 | 29887039 | T | G | het | . | . | . | 35 | 0 | 0% | T | 26 | 4 | 13.33% | K | Somatic | 0.0404777 |
| splicing | KATNAL1(NM_032116:exon11:c.1148-1G>A,NM_001014380:exon11:c.1148-1G>A) |  |  | 535;Name=lod=200 |  |  |  |  |  |  |  |  |  |  | chr13 | 30784580 | 30784580 | C | T | het | . | . | . | 213 | 0 | 0% | C | 106 | 4 | 3.64% | Y | Somatic | 0.0129692 |
| intronic | SLFN13 |  |  |  |  |  |  |  |  |  |  |  |  |  | chr17 | 33770993 | 33770993 | G | T | het | . | . | . | 68 | 0 | 0% | G | 17 | 2 | 10.53% | K | Somatic | 0.0457097 |
| intronic | COL4A5 |  |  |  |  | 0.14 | 0.14 | 0.14 | rs1006269 |  |  |  |  |  | chrX | 107865895 | 107865895 | A | G | het | . | . | . | 194 | 0 | 0% | A | 133 | 5 | 3.62% | R | Somatic | 0.0118858 |
| intronic | TMEM101 |  |  | 259;Name=lod=15 |  |  |  |  |  |  |  |  |  |  | chr17 | 42090542 | 42090542 | A | C | het | . | . | . | 24 | 0 | 0% | A | 35 | 7 | 16.67% | M | Somatic | 0.0346414 |
| exonic | CDC27 | synonymous SNV | NM_001114091:c.T165C:p.Y55Y | 690;Name=lod=850 |  |  |  |  |  |  |  |  |  |  | chr17 | 45249369 | 45249369 | A | G | het | . | . | . | 79 | 1 | 1.25% | A | 34 | 5 | 12.82% | R | Somatic | 0.0142137 |
| intronic | ABI3 |  |  |  |  |  |  |  |  |  |  |  |  |  | chr17 | 47299857 | 47299857 | T | G | het | . | . | . | 37 | 0 | 0% | T | 51 | 6 | 10.53% | K | Somatic | 0.0445683 |
| exonic | SPATA20 | nonsynonymous SNV | NM_022827:c.A373C:p.T125P | 596;Name=lod=351 |  |  |  |  |  | 0 | 0.854 | 0.945428 | 0.945314 | 0.999996 | chr17 | 48626182 | 48626182 | A | C | het | large_intestine | carcinoma | adenocarcinoma | 51 | 1 | 1.92% | A | 48 | 9 | 15.79% | M | Somatic | 0.0119857 |
| intronic | PPM1E |  |  |  |  |  |  |  |  |  |  |  |  |  | chr17 | 57032949 | 57032949 | G | C | het | . | . | . | 67 | 0 | 0% | G | 13 | 2 | 13.33% | S | Somatic | 0.031617 |
| intronic | ABCA6 |  |  |  |  |  |  |  |  |  |  |  |  |  | chr17 | 67099146 | 67099146 | T | A | het | . | . | . | 48 | 0 | 0% | T | 12 | 3 | 20% | W | Somatic | 0.0114578 |
| exonic | CDC42EP4 | nonsynonymous SNV | NM_012121:c.T856G:p.W286G | 358;Name=lod=38 |  |  |  |  |  | 0 | 0.999 | 0.997076 | 0.172726 | 0.157181 | chr17 | 71281784 | 71281784 | A | C | het | . | . | . | 61 | 1 | 1.61% | A | 14 | 3 | 17.65% | M | Somatic | 0.0296439 |
| intronic | EVPL |  |  |  |  |  |  |  |  |  |  |  |  |  | chr17 | 74014975 | 74014975 | T | G | het | . | . | . | 31 | 0 | 0% | T | 19 | 4 | 17.39% | K | Somatic | 0.0279999 |
| exonic | ARL16 | nonsynonymous SNV | NM_001040025:c.T164G:p.L55R | 265;Name=lod=16 |  |  |  |  | rs79986591 | 0 |  |  |  |  | chr17 | 79650594 | 79650594 | A | C | het | . | . | . | 23 | 0 | 0% | A | 13 | 4 | 23.53% | M | Somatic | 0.0260422 |
| intronic | ASPSCR1 |  |  |  |  |  |  |  |  |  |  |  |  |  | chr17 | 79975011 | 79975011 | T | G | het | . | . | . | 45 | 0 | 0% | T | 19 | 3 | 13.64% | K | Somatic | 0.032147 |
| exonic | PQLC1 | nonsynonymous SNV | NM_001146343:c.T89G:p.V30G | 507;Name=lod=153 |  |  |  |  |  | 0 | 0.995 | 0.996952 | 0.999037 | 0.999997 | chr18 | 77710838 | 77710838 | A | C | het | . | . | . | 32 | 0 | 0% | A | 34 | 5 | 12.82% | M | Somatic | 0.0442213 |
| intronic | CDC34 |  |  |  |  |  |  |  |  |  |  |  |  |  | chr19 | 537198 | 537198 | A | C | het | . | . | . | 34 | 0 | 0% | A | 31 | 7 | 18.42% | M | Somatic | 0.0085671 |
| intronic | ADAMTSL5 |  |  |  |  |  |  |  | rs56891350 |  |  |  |  |  | chr19 | 1510333 | 1510333 | T | G | het | . | . | . | 32 | 0 | 0% | T | 45 | 6 | 11.76% | K | Somatic | 0.0477139 |
| exonic | FAM108A1 | nonsynonymous SNV | NM_001130111:c.T821G:p.V274G | 572;Name=lod=281 | 0.99 |  |  |  |  | 0 | 0.992 | 0.997015 | 0.999825 | 1 | chr19 | 1877311 | 1877311 | A | C | het | . | . | . | 82 | 0 | 0% | A | 36 | 6 | 14.29% | M | Somatic | 0.0011747 |
| exonic | CSNK1G2 | nonsynonymous SNV | NM_001319:c.T1039C:p.S347P |  |  |  |  |  |  | 0.07 | 0 | 0.001409 | 0.829661 | 0.54599 | chr19 | 1979787 | 1979787 | T | C | het | . | . | . | 38 | 0 | 0% | T | 30 | 4 | 11.76% | Y | Somatic | 0.0450782 |
| intronic | DOT1L |  |  |  |  |  |  |  |  |  |  |  |  |  | chr19 | 2214036 | 2214036 | T | G | het | . | . | . | 41 | 0 | 0% | T | 16 | 3 | 15.79% | K | Somatic | 0.0283168 |
| splicing | RANBP3(NM_003624:exon2:c.22+2T>G,NM_007320:exon2:c.22+2T>G,NM_007322:exon2:c.22+2T>G) |  |  | 586;Name=lod=322 |  |  |  |  |  |  |  |  |  |  | chr19 | 5978070 | 5978070 | A | C | het | . | . | . | 37 | 0 | 0% | A | 42 | 6 | 12.50% | M | Somatic | 0.0280586 |
| intronic | TRIP10 |  |  |  |  |  |  |  |  |  |  |  |  |  | chr19 | 6739846 | 6739846 | A | C | het | . | . | . | 37 | 0 | 0% | A | 38 | 5 | 11.63% | M | Somatic | 0.0400415 |
| exonic | MUC16 | synonymous SNV | NM_024690:c.A6867C:p.T2289T |  |  |  |  |  |  |  |  |  |  |  | chr19 | 9084948 | 9084948 | T | G | het | . | . | . | 70 | 1 | 1.41% | T | 22 | 4 | 15.38% | K | Somatic | 0.0174911 |
| exonic | KCNH8 | nonsynonymous SNV | NM_144633:c.A2678G:p.Q893R | 484;Name=lod=124 |  | 0.05 | 0.05 | 0.05 | rs33915638 | 0.19 | 0.006 | 0.998806 | 5.50E-04 | 0.936403 | chr3 | 19574945 | 19574945 | A | G | het | . | . | . | 130 | 0 | 0% | A | 107 | 4 | 3.60% | R | Somatic | 0.0436881 |
| exonic | CHIA | nonsynonymous SNV | NM_021797:c.G580A:p.A194T | 514;Name=lod=164 |  |  |  |  |  | 0 | 0.995 | 0.999321 | 0.931563 | 0.997196 | chr1 | 111861289 | 111861289 | G | A | het | . | . | . | 150 | 0 | 0% | G | 108 | 4 | 3.57% | R | Somatic | 0.0323702 |
| intronic | KRI1 |  |  |  |  | 0.06 | 0.06 | 0.06 | rs148315651 |  |  |  |  |  | chr19 | 10665932 | 10665932 | T | C | het | . | . | . | 79 | 0 | 0% | T | 41 | 44 | 51.76% | Y | Somatic | 1.71E-16 |
| intronic | KRI1 |  |  |  |  | 0.06 | 0.06 | 0.06 | rs117140161 |  |  |  |  |  | chr19 | 10665933 | 10665933 | G | C | het | . | . | . | 79 | 0 | 0% | G | 43 | 43 | 50% | S | Somatic | 7.30E-16 |
| intronic | KRI1 |  |  |  |  | 0.04 | 0.04 | 0.04 | rs114840087 |  |  |  |  |  | chr19 | 10665938 | 10665938 | C | T | het | . | . | . | 79 | 0 | 0% | C | 47 | 39 | 45.35% | Y | Somatic | 4.10E-14 |
| exonic | INTS2 | nonsynonymous SNV | NM_020748:c.C296T:p.A99V | 590;Name=lod=334 |  |  |  |  |  | 1 |  |  |  |  | chr17 | 60003734 | 60003734 | G | A | het | . | . | . | 162 | 0 | 0% | G | 109 | 4 | 3.54% | R | Somatic | 0.0276183 |
| exonic | CPZ | synonymous SNV | NM_003652:c.C204T:p.Y68Y | 536;Name=lod=202 |  |  |  |  |  |  |  |  |  |  | chr4 | 8602965 | 8602965 | C | T | het | . | . | . | 130 | 0 | 0% | C | 111 | 4 | 3.48% | Y | Somatic | 0.0471978 |
| exonic | MCM4 | nonsynonymous SNV | NM_005914:c.G2336A:p.G779D | 477;Name=lod=116 |  |  |  |  |  | 0 | 0.995 | 0.992789 | 0.999994 | 1 | chr8 | 48887493 | 48887493 | G | A | het | . | . | . | 157 | 0 | 0% | G | 111 | 4 | 3.48% | R | Somatic | 0.0309917 |
| exonic | ZBTB20 | nonsynonymous SNV | NM_001164342:c.A758T:p.Q253L | 533;Name=lod=195 |  |  |  |  |  | 0 | 0.982 | 0.998613 | 0.999232 | 1 | chr3 | 114070167 | 114070167 | T | A | het | . | . | . | 182 | 0 | 0% | T | 112 | 4 | 3.45% | W | Somatic | 0.0222358 |
| exonic | SERPINA9 | synonymous SNV | NM_175739:c.C1197T:p.G399G |  |  | 0.65 | 0.65 | 0.65 | rs2224418 |  |  |  |  |  | chr14 | 94929541 | 94929541 | G | A | het | . | . | . | 189 | 0 | 0% | G | 113 | 4 | 3.42% | R | Somatic | 0.0206971 |
| intronic | DAPK2 |  |  |  |  |  |  |  |  |  |  |  |  |  | chr15 | 64231391 | 64231391 | C | T | het | . | . | . | 148 | 0 | 0% | C | 141 | 5 | 3.42% | Y | Somatic | 0.0291621 |
| exonic | CDKL3 | nonsynonymous SNV | NM_001113575:c.A1673T:p.K558M | 580;Name=lod=304 |  |  |  |  |  | 0 |  |  |  |  | chr5 | 133638327 | 133638327 | T | A | het | . | . | . | 93 | 0 | 0% | T | 57 | 2 | 3.39% | W | Somatic | 0.1490938 |
| UTR3 | ZBTB48 |  |  |  |  |  |  |  | rs1042775 |  |  |  |  |  | chr1 | 6649279 | 6649279 | C | T | het | . | . | . | 136 | 0 | 0% | C | 115 | 4 | 3.36% | Y | Somatic | 0.0461507 |
| exonic | ZNF44 | synonymous SNV | NM_016264:c.A1847G:p.X616X |  |  |  |  |  |  |  |  |  |  |  | chr19 | 12383223 | 12383223 | T | C | het | . | . | . | 73 | 0 | 0% | T | 38 | 5 | 11.63% | Y | Somatic | 0.0060016 |
| exonic | ZNF44 | synonymous SNV | NM_016264:c.G1572A:p.R524R |  |  |  |  |  |  |  |  |  |  |  | chr19 | 12383498 | 12383498 | C | T | het | lung | carcinoma | squamous_cell_carcinoma | 93 | 0 | 0% | C | 31 | 4 | 11.43% | Y | Somatic | 0.0049081 |
| intronic | ZNF709 |  |  |  |  |  |  |  |  |  |  |  |  |  | chr19 | 12595392 | 12595392 | T | G | het | . | . | . | 30 | 0 | 0% | T | 15 | 3 | 16.67% | K | Somatic | 0.0471785 |
| intronic | ADAMTS9 |  |  |  |  | 0.02 | 0.02 | 0.02 | rs74534712 |  |  |  |  |  | chr3 | 64527501 | 64527501 | T | C | het | . | . | . | 204 | 0 | 0% | T | 116 | 4 | 3.33% | Y | Somatic | 0.0182258 |
| exonic | UNC13A | nonsynonymous SNV | NM_001080421:c.T3476G:p.V1159G | 601;Name=lod=368 |  |  |  |  |  | 0 |  |  |  |  | chr19 | 17741512 | 17741512 | A | C | het | . | . | . | 51 | 1 | 1.92% | A | 121 | 18 | 12.95% | M | Somatic | 0.0152096 |
| exonic | ELL | nonsynonymous SNV | NM_006532:c.A1459C:p.T487P |  |  |  |  |  |  | 0.07 | 0 | 0.767207 | 4.65E-04 | 0.448256 | chr19 | 18561293 | 18561293 | T | G | het | . | . | . | 45 | 0 | 0% | T | 30 | 4 | 11.76% | K | Somatic | 0.0308659 |
| exonic | TMEM161A | nonsynonymous SNV | NM_017814:c.T986G:p.V329G | 606;Name=lod=388 |  |  |  |  |  | 0 | 0.963 | 0.962849 | 0.898651 | 0.999813 | chr19 | 19231904 | 19231904 | A | C | het | . | . | . | 48 | 0 | 0% | A | 33 | 4 | 10.81% | M | Somatic | 0.0326183 |
| exonic | RFX6 | synonymous SNV | NM_173560:c.C2139A:p.S713S |  |  |  |  |  |  |  |  |  |  |  | chr6 | 117248443 | 117248443 | C | A | het | . | . | . | 172 | 0 | 0% | C | 118 | 4 | 3.28% | M | Somatic | 0.0287993 |
| ncRNA_intronic | CHKB-CPT1B |  |  |  |  | 0.36 | 0.36 | 0.36 | rs140514 |  |  |  |  |  | chr22 | 51018579 | 51018579 | A | G | het | . | . | . | 208 | 0 | 0% | A | 149 | 5 | 3.25% | R | Somatic | 0.0134162 |
| exonic | ZNF257 | nonsynonymous SNV | NM_033468:c.G830T:p.R277I | 385;Name=lod=49 |  |  |  |  |  | 0.24 |  |  |  |  | chr19 | 22271382 | 22271382 | G | T | het | large_intestine | carcinoma | adenocarcinoma | 260 | 4 | 1.52% | G | 56 | 7 | 11.11% | K | Somatic | 0.0012633 |
| UTR3 | ZNF99 |  |  |  | 0.91 |  |  |  |  | 0.39 |  |  |  |  | chr19 | 22939306 | 22939306 | A | C | het | . | . | . | 389 | 7 | 1.77% | A | 104 | 12 | 10.34% | M | Somatic | 1.31E-04 |
| exonic | SCPEP1 | nonsynonymous SNV | NM_021626:c.G700A:p.A234T | 361;Name=lod=39 |  |  |  |  |  | 0.01 | 0.121 | 0.999678 | 0.999869 | 1 | chr17 | 55072910 | 55072910 | G | A | het | . | . | . | 151 | 0 | 0% | G | 121 | 4 | 3.20% | R | Somatic | 0.0409677 |
| exonic | GPI | nonsynonymous SNV | NM_000175:c.A59C:p.H20P |  |  |  |  |  | rs137853586 | 0.28 | 0 | 0.972269 | 0.993293 | 0.99979 | chr19 | 34856230 | 34856230 | A | C | het | . | . | . | 63 | 1 | 1.56% | A | 61 | 7 | 10.29% | M | Somatic | 0.0377082 |
| UTR5 | ZNF302 |  |  |  | 0.9 |  |  |  |  |  |  |  |  |  | chr19 | 35169703 | 35169703 | C | T | het | . | . | . | 60 | 0 | 0% | C | 29 | 4 | 12.12% | Y | Somatic | 0.014015 |
| intronic | ZNF302 |  |  |  | 0.9 |  |  |  |  |  |  |  |  |  | chr19 | 35169772 | 35169772 | G | A | het | . | . | . | 56 | 0 | 0% | G | 19 | 3 | 13.64% | R | Somatic | 0.0202429 |
| exonic | ZNF599 | synonymous SNV | NM_001007248:c.G1290A:p.K430K | 440;Name=lod=82 |  |  |  |  |  |  |  |  |  |  | chr19 | 35250416 | 35250416 | C | T | het | . | . | . | 162 | 1 | 0.61% | C | 44 | 6 | 12% | Y | Somatic | 7.53E-04 |
| exonic | ZNF30 | nonsynonymous SNV | NM_001099437:c.C761T:p.T254I |  |  |  |  |  |  | 1 |  |  |  |  | chr19 | 35434628 | 35434628 | C | T | het | . | . | . | 93 | 0 | 0% | C | 19 | 4 | 17.39% | Y | Somatic | 0.0012367 |
| exonic | ZNF30 | synonymous SNV | NM_001099437:c.C765T:p.H255H | 383;Name=lod=48 |  |  |  |  |  |  |  |  |  |  | chr19 | 35434632 | 35434632 | C | T | het | . | . | . | 94 | 0 | 0% | C | 20 | 4 | 16.67% | Y | Somatic | 0.0013847 |
| exonic | ZNF30 | synonymous SNV | NM_001099437:c.A774G:p.E258E | 383;Name=lod=48 |  |  |  |  |  |  |  |  |  |  | chr19 | 35434641 | 35434641 | A | G | het | . | . | . | 95 | 0 | 0% | A | 22 | 5 | 18.52% | R | Somatic | 3.89E-04 |
| exonic | ZNF30 | nonsynonymous SNV | NM_001099437:c.G785A:p.G262E |  |  |  |  |  |  | 1 |  |  |  |  | chr19 | 35434652 | 35434652 | G | A | het | . | . | . | 94 | 1 | 1.05% | G | 25 | 3 | 10.71% | R | Somatic | 0.0365358 |
| exonic | ZNF30 | nonsynonymous SNV | NM_001099437:c.G790A:p.E264K | 251;Name=lod=14 |  |  |  |  |  | 1 |  |  |  |  | chr19 | 35434657 | 35434657 | G | A | het | . | . | . | 95 | 1 | 1.04% | G | 24 | 4 | 14.29% | R | Somatic | 0.0091667 |
| exonic | ZNF30 | synonymous SNV | NM_001099437:c.G1197A:p.K399K | 336;Name=lod=31 |  |  |  |  |  |  |  |  |  |  | chr19 | 35435064 | 35435064 | G | A | het | . | . | . | 128 | 2 | 1.54% | G | 43 | 7 | 14% | R | Somatic | 0.0020395 |
| exonic | ARHGAP33 | nonsynonymous SNV | NM_001172630:c.A2894C:p.H965P | 483;Name=lod=122 |  |  |  |  |  | 0 | 0.999 | 0.997807 | 0.265195 | 0.980374 | chr19 | 36278853 | 36278853 | A | C | het | . | . | . | 27 | 0 | 0% | A | 13 | 3 | 18.75% | M | Somatic | 0.0453772 |
| exonic | ZCRB1 | synonymous SNV | NM_033114:c.C300T:p.N100N | 671;Name=lod=714 |  |  |  |  |  |  |  |  |  |  | chr12 | 42711175 | 42711175 | G | A | het | . | . | . | 85 | 0 | 0% | G | 61 | 2 | 3.17% | R | Somatic | 0.1795367 |
| exonic | SLC35A3 | nonsynonymous SNV | NM_012243:c.C506T:p.A169V | 391;Name=lod=52 |  |  |  |  |  | 0.14 | 0 | 0.978424 | 0.974782 | 0.999993 | chr1 | 100476961 | 100476961 | C | T | het | . | . | . | 183 | 0 | 0% | C | 123 | 4 | 3.15% | Y | Somatic | 0.027384 |
| intronic | CAMK2G |  |  | 576;Name=lod=291 |  | 0.17 | 0.17 | 0.17 | rs2242255 |  |  |  |  |  | chr10 | 75597289 | 75597289 | T | A | het | . | . | . | 195 | 0 | 0% | T | 155 | 5 | 3.12% | W | Somatic | 0.0179615 |
| exonic | RUFY3 | synonymous SNV | NM_001037442:c.C1854T:p.S618S | 329;Name=lod=29 | 0.91 |  |  |  |  |  |  |  |  |  | chr4 | 71672367 | 71672367 | C | T | het | . | . | . | 207 | 0 | 0% | C | 124 | 4 | 3.12% | Y | Somatic | 0.0206976 |
| exonic | ZNF781 | nonsynonymous SNV | NM_152605:c.G689A:p.S230N |  |  |  |  |  |  | 0.38 | 0.0064 | 0.057144 | 3.97E-04 | 0.597643 | chr19 | 38160361 | 38160361 | C | T | het | . | . | . | 260 | 4 | 1.52% | C | 69 | 9 | 11.54% | Y | Somatic | 3.60E-04 |
| splicing | PQLC3(NM_152391:exon6:c.517+1G>A) |  |  | 329;Name=lod=29 |  |  |  |  |  |  |  |  |  |  | chr2 | 11315136 | 11315136 | G | A | het | . | . | . | 164 | 0 | 0% | G | 125 | 4 | 3.10% | R | Somatic | 0.0365959 |
| intronic | C5orf41 |  |  |  |  |  |  |  |  |  |  |  |  |  | chr5 | 172550218 | 172550218 | C | T | het | . | . | . | 162 | 0 | 0% | C | 127 | 4 | 3.05% | Y | Somatic | 0.0389469 |
| intronic | RYR1 |  |  |  |  |  |  |  |  |  |  |  |  |  | chr19 | 38934187 | 38934187 | T | C | het | . | . | . | 36 | 0 | 0% | T | 25 | 4 | 13.79% | Y | Somatic | 0.0350806 |
| exonic | LRFN1 | nonsynonymous SNV | NM_020862:c.G1792A:p.A598T |  |  |  |  |  |  | 0.83 |  |  |  |  | chr19 | 39798797 | 39798797 | C | T | het | . | . | . | 41 | 0 | 0% | C | 9 | 2 | 18.18% | Y | Somatic | 0.0414781 |
| exonic | HNRNPUL1 | nonsynonymous SNV | NM_007040:c.G206A:p.G69D | 707;Name=lod=1000 |  |  |  |  |  | 0.33 | 0.001 | 0.998234 | 0.316871 | 0.844315 | chr19 | 41770614 | 41770614 | G | A | het | . | . | . | 43 | 0 | 0% | G | 36 | 4 | 10% | R | Somatic | 0.0497328 |
| intronic | RABAC1 |  |  |  |  |  |  |  |  |  |  |  |  |  | chr19 | 42463111 | 42463111 | T | G | het | . | . | . | 24 | 0 | 0% | T | 9 | 3 | 25% | K | Somatic | 0.0308123 |
| exonic | ZNF404 | nonsynonymous SNV | NM_001033719:c.A1162G:p.T388A | 340;Name=lod=32 |  |  |  |  |  | 1 |  |  |  |  | chr19 | 44377195 | 44377195 | T | C | het | . | . | . | 110 | 1 | 0.90% | T | 14 | 2 | 12.50% | Y | Somatic | 0.0416348 |
| exonic | ZNF404 | synonymous SNV | NM_001033719:c.G1161A:p.K387K | 340;Name=lod=32 |  |  |  |  |  |  |  |  |  |  | chr19 | 44377196 | 44377196 | C | T | het | . | . | . | 111 | 1 | 0.89% | C | 15 | 2 | 11.76% | Y | Somatic | 0.0455274 |
| exonic | ZNF226 | synonymous SNV | NM_001032372:c.G2007A:p.K669K | 431;Name=lod=75 |  |  |  |  |  |  |  |  |  |  | chr19 | 44681422 | 44681422 | G | A | het | . | . | . | 278 | 3 | 1.07% | G | 116 | 15 | 11.45% | R | Somatic | 6.03E-06 |
| exonic | ZNF180 | nonsynonymous SNV | NM_013256:c.T1457C:p.I486T | 488;Name=lod=128 |  |  |  |  |  | 0.41 | 0.008 | 0.997832 | 0.587258 | 0.999664 | chr19 | 44981241 | 44981241 | A | G | het | . | . | . | 375 | 4 | 1.06% | A | 118 | 15 | 11.28% | R | Somatic | 1.27E-06 |
| exonic | CCDC8 | nonsynonymous SNV | NM_032040:c.T662G:p.V221G |  |  |  |  |  |  | 0 | 0.007 | 0.068229 | 0.320583 | 0.092325 | chr19 | 46915406 | 46915406 | A | C | het | . | . | . | 54 | 0 | 0% | A | 18 | 3 | 14.29% | M | Somatic | 0.0196964 |
| intronic | FAM83E |  |  |  |  |  |  |  |  |  |  |  |  |  | chr19 | 49113109 | 49113109 | T | G | het | . | . | . | 54 | 1 | 1.82% | T | 56 | 9 | 13.85% | K | Somatic | 0.0166901 |
| exonic | RCN3 | nonsynonymous SNV | NM_020650:c.A964C:p.T322P | 401;Name=lod=57 |  |  |  |  |  | 0.01 | 0.679 | 0.997096 | 0.938289 | 1 | chr19 | 50046447 | 50046447 | A | C | het | . | . | . | 47 | 0 | 0% | A | 43 | 6 | 12.24% | M | Somatic | 0.0150842 |
| exonic | PNKP | nonsynonymous SNV | NM_007254:c.C56G:p.A19G |  |  |  |  |  |  | 0.58 | 0.003 | 0.99238 | 1.36E-04 | 0.558954 | chr19 | 50370406 | 50370406 | G | C | het | . | . | . | 50 | 0 | 0% | G | 36 | 5 | 12.20% | S | Somatic | 0.0161145 |
| exonic | TBC1D17 | nonsynonymous SNV | NM_001168222:c.T269G:p.L90R | 516;Name=lod=167 |  |  |  |  |  | 0 | 0.967 | 0.965249 | 0.498055 | 0.999999 | chr19 | 50384573 | 50384573 | T | G | het | . | . | . | 49 | 1 | 2% | T | 51 | 10 | 16.39% | K | Somatic | 0.0104111 |
| intronic | TBC1D17 |  |  |  |  |  |  |  |  |  |  |  |  |  | chr19 | 50385981 | 50385981 | A | C | het | . | . | . | 32 | 0 | 0% | A | 15 | 3 | 16.67% | M | Somatic | 0.0416327 |
| intronic | SHANK1 |  |  |  |  |  |  |  |  |  |  |  |  |  | chr19 | 51165962 | 51165962 | T | G | het | . | . | . | 22 | 0 | 0% | T | 14 | 4 | 22.22% | K | Somatic | 0.0334829 |
| exonic | ACPT | nonsynonymous SNV | NM_033068:c.A871C:p.T291P | 533;Name=lod=196 |  |  |  |  |  | 0 | 0.998 | 0.997488 | 0.996739 | 1 | chr19 | 51297723 | 51297723 | A | C | het | . | . | . | 59 | 1 | 1.67% | A | 107 | 13 | 10.83% | M | Somatic | 0.0231483 |
| exonic | MANSC1 | synonymous SNV | NM_018050:c.C288T:p.Y96Y | 496;Name=lod=138 |  |  |  |  |  |  |  |  |  |  | chr12 | 12491430 | 12491430 | G | A | het | . | . | . | 180 | 0 | 0% | G | 128 | 4 | 3.03% | R | Somatic | 0.0311992 |
| exonic | GNA14 | synonymous SNV | NM_004297:c.G789A:p.S263S | 626;Name=lod=465 |  | 0.09 | 0.09 | 0.09 | rs1801259 |  |  |  |  |  | chr9 | 80040566 | 80040566 | C | T | het | . | . | . | 173 | 0 | 0% | C | 128 | 4 | 3.03% | Y | Somatic | 0.0341787 |
| exonic | ABCC9 | nonsynonymous SNV | NM_005691:c.G1988A:p.R663H | 243;Name=lod=13 |  |  |  |  | rs141999048 | 0.53 | 0.802 | 0.976124 | 0.998238 | 0.9981 | chr12 | 22035731 | 22035731 | C | T | het | . | . | . | 199 | 0 | 0% | C | 130 | 4 | 2.99% | Y | Somatic | 0.0255198 |
| exonic | INPP5F | nonsynonymous SNV | NM_014937:c.C1727G:p.S576C | 729;Name=lod=1218 |  |  |  |  |  | 0.03 | 0.985 | 0.998639 | 0.999381 | 0.999936 | chr10 | 121571308 | 121571308 | C | G | het | . | . | . | 189 | 0 | 0% | C | 131 | 4 | 2.96% | S | Somatic | 0.02936 |
| splicing | MYO3A(NM_017433:exon3:c.168+1G>A) |  |  | 409;Name=lod=61 |  |  |  |  |  |  |  |  |  |  | chr10 | 26241208 | 26241208 | G | A | het | . | . | . | 174 | 0 | 0% | G | 66 | 2 | 2.94% | R | Somatic | 0.078118 |
| exonic | ZNF432 | nonsynonymous SNV | NM_014650:c.C1373T:p.T458I |  |  |  |  |  |  | 0.5 | 0 | 0.077263 | 0.109577 | 0.612293 | chr19 | 52537559 | 52537559 | G | A | het | . | . | . | 225 | 3 | 1.32% | G | 61 | 8 | 11.59% | R | Somatic | 5.42E-04 |
| exonic | ZNF880 | nonsynonymous SNV | NM_001145434:c.G770A:p.R257Q |  |  |  |  |  | rs78052167 | 0.56 |  |  |  |  | chr19 | 52887603 | 52887603 | G | A | het | . | . | . | 150 | 2 | 1.32% | G | 37 | 6 | 13.95% | R | Somatic | 0.0016731 |
| ncRNA_exonic | ZNF137P |  |  |  |  |  |  |  |  |  |  |  |  |  | chr19 | 53100038 | 53100038 | A | G | het | . | . | . | 95 | 1 | 1.04% | A | 28 | 4 | 12.50% | R | Somatic | 0.0138095 |
| exonic | TERT | synonymous SNV | NM_001193376:c.C3033T:p.C1011C | 366;Name=lod=41 |  |  |  |  |  |  |  |  |  |  | chr5 | 1254556 | 1254556 | G | A | het | . | . | . | 168 | 0 | 0% | G | 132 | 4 | 2.94% | R | Somatic | 0.0390786 |
| exonic | ARHGAP10 | synonymous SNV | NM_024605:c.C216T:p.I72I | 454;Name=lod=93 |  |  |  |  |  |  |  |  |  |  | chr4 | 148743939 | 148743939 | C | T | het | . | . | . | 184 | 0 | 0% | C | 133 | 4 | 2.92% | Y | Somatic | 0.0323463 |
| exonic | ZNF665 | nonsynonymous SNV | NM_024733:c.T1181C:p.I394T |  |  |  |  |  |  | 0.44 |  |  |  |  | chr19 | 53668562 | 53668562 | A | G | het | . | . | . | 220 | 3 | 1.35% | A | 72 | 8 | 10% | R | Somatic | 0.0014313 |
| exonic | CNOT3 | nonsynonymous SNV | NM_014516:c.A968C:p.Y323S | 545;Name=lod=218 |  |  |  |  |  | 0.61 | 0.981 | 0.994139 | 0.179749 | 0.770304 | chr19 | 54651956 | 54651956 | A | C | het | . | . | . | 30 | 0 | 0% | A | 17 | 5 | 22.73% | M | Somatic | 0.0101325 |
| exonic | DDX43 | stopgain SNV | NM_018665:c.G549A:p.W183X | 251;Name=lod=14 |  |  |  |  |  | 0.37 | 0.735462 | 0.999528 | 1 | 0.999884 | chr6 | 74111694 | 74111694 | G | A | het | . | . | . | 190 | 0 | 0% | G | 133 | 4 | 2.92% | R | Somatic | 0.0300265 |
| exonic | ZNF784 | nonsynonymous SNV | NM_203374:c.T950G:p.V317G |  |  |  |  |  |  | 0 | 0.137 | 0.820539 | 0.030738 | 0.569584 | chr19 | 56133139 | 56133139 | A | C | het | . | . | . | 43 | 0 | 0% | A | 45 | 6 | 11.76% | M | Somatic | 0.0221188 |
| exonic | PEG3 | synonymous SNV | NM_001146186:c.T1887C:p.C629C |  |  |  |  |  |  |  |  |  |  |  | chr19 | 57327923 | 57327923 | A | G | het | . | . | . | 137 | 2 | 1.44% | A | 36 | 4 | 10% | R | Somatic | 0.0231512 |
| exonic | PEG3 | synonymous SNV | NM_001146186:c.A1884G:p.E628E |  |  |  |  |  |  |  |  |  |  |  | chr19 | 57327926 | 57327926 | T | C | het | . | . | . | 133 | 2 | 1.48% | T | 35 | 4 | 10.26% | Y | Somatic | 0.023351 |
| exonic | PEG3 | nonsynonymous SNV | NM_001146186:c.A1876T:p.M626L |  |  |  |  |  |  | 1 | 0.24 | 0.057408 | 0 | 0.217261 | chr19 | 57327934 | 57327934 | T | A | het | . | . | . | 131 | 2 | 1.50% | T | 34 | 4 | 10.53% | W | Somatic | 0.0225771 |
| exonic | PEG3 | nonsynonymous SNV | NM_001146186:c.G1872C:p.E624D | 317;Name=lod=26 |  |  |  |  |  | 0.32 | 0.009 | 0.206524 | 0.394308 | 0.974092 | chr19 | 57327938 | 57327938 | C | G | het | . | . | . | 124 | 2 | 1.59% | C | 29 | 6 | 17.14% | S | Somatic | 0.0014551 |
| exonic | PEG3 | synonymous SNV | NM_001146186:c.T1866C:p.G622G |  |  |  |  |  | rs145787021 |  |  |  |  |  | chr19 | 57327944 | 57327944 | A | G | het | . | . | . | 108 | 2 | 1.82% | A | 27 | 7 | 20.59% | R | Somatic | 6.03E-04 |
| exonic | PEG3 | nonsynonymous SNV | NM_001146186:c.G1860A:p.M620I |  |  | 0.001 | 0.001 | 0.001 | rs150420781 | 0.18 | 0.998 | 0.928884 | 0.600941 | 0.98493 | chr19 | 57327950 | 57327950 | C | T | het | . | . | . | 105 | 2 | 1.87% | C | 23 | 7 | 23.33% | Y | Somatic | 3.40E-04 |
| exonic | PEG3 | nonsynonymous SNV | NM_001146186:c.T1849C:p.F617L |  |  |  |  |  | rs141082433 | 0.59 | 0.012 | 0.161053 | 0.177939 | 0.806409 | chr19 | 57327961 | 57327961 | A | G | het | . | . | . | 111 | 2 | 1.77% | A | 15 | 6 | 28.57% | R | Somatic | 1.71E-04 |
| exonic | PEG3 | nonsynonymous SNV | NM_001146186:c.A1844G:p.N615S |  |  |  |  |  |  | 0.32 | 0.01 | 2.07E-04 | 0 | 0.445633 | chr19 | 57327966 | 57327966 | T | C | het | . | . | . | 116 | 2 | 1.69% | T | 17 | 6 | 26.09% | Y | Somatic | 2.29E-04 |
| exonic | PEG3 | nonsynonymous SNV | NM_001146186:c.C1840T:p.L614F |  |  |  |  |  |  | 0.36 | 0 | 0.790603 | 0.181829 | 0.398241 | chr19 | 57327970 | 57327970 | G | A | het | . | . | . | 116 | 2 | 1.69% | G | 17 | 6 | 26.09% | R | Somatic | 2.29E-04 |
| exonic | PEG3 | nonsynonymous SNV | NM_001146186:c.G1837A:p.A613T |  |  |  |  |  |  | 1 | 0 | 2.00E-05 | 0 | 0.098337 | chr19 | 57327973 | 57327973 | C | T | het | . | . | . | 110 | 2 | 1.79% | C | 17 | 6 | 26.09% | Y | Somatic | 2.96E-04 |
| exonic | PEG3 | synonymous SNV | NM_001146186:c.A1836C:p.P612P |  |  |  |  |  |  |  |  |  |  |  | chr19 | 57327974 | 57327974 | T | G | het | . | . | . | 109 | 2 | 1.80% | T | 17 | 6 | 26.09% | K | Somatic | 3.09E-04 |
| exonic | PEG3 | synonymous SNV | NM_001146186:c.C1830T:p.P610P |  |  |  |  |  |  |  |  |  |  |  | chr19 | 57327980 | 57327980 | G | A | het | . | . | . | 117 | 1 | 0.85% | G | 17 | 5 | 22.73% | R | Somatic | 3.39E-04 |
| exonic | PEG3 | nonsynonymous SNV | NM_001146186:c.G1826T:p.R609M |  |  |  |  |  |  | 0.14 | 0 | 0.798641 | 0.400447 | 0.849832 | chr19 | 57327984 | 57327984 | C | A | het | . | . | . | 111 | 1 | 0.89% | C | 19 | 4 | 17.39% | M | Somatic | 0.0029576 |
| exonic | PEG3 | synonymous SNV | NM_001146186:c.T1824C:p.F608F |  |  |  |  |  |  |  |  |  |  |  | chr19 | 57327986 | 57327986 | A | G | het | . | . | . | 110 | 0 | 0% | A | 18 | 4 | 18.18% | R | Somatic | 6.05E-04 |
| exonic | PEG3 | nonsynonymous SNV | NM_001146186:c.A1819G:p.T607A |  |  |  |  |  |  | 1 | 0 | 0.099076 | 0 | 0.068804 | chr19 | 57327991 | 57327991 | T | C | het | . | . | . | 112 | 0 | 0% | T | 20 | 3 | 13.04% | Y | Somatic | 0.0044165 |
| intergenic | SSX1(dist=34256),SSX3(dist=44728) |  |  |  | 0.96 |  |  |  |  |  |  |  |  |  | chrX | 48161135 | 48161135 | G | A | het | . | . | . | 213 | 0 | 0% | G | 135 | 4 | 2.88% | R | Somatic | 0.0236815 |
| exonic | FAM117B | nonsynonymous SNV | NM_173511:c.C1465T:p.H489Y | 696;Name=lod=897 |  |  |  |  |  | 0.28 | 0.998 | 0.998974 | 0.828377 | 1 | chr2 | 203630182 | 203630182 | C | T | het | . | . | . | 174 | 0 | 0% | C | 136 | 4 | 2.86% | Y | Somatic | 0.0385792 |
| exonic | ZNF460 | synonymous SNV | NM_006635:c.G915A:p.K305K |  |  | 0.16 | 0.16 | 0.16 | rs2041110 |  |  |  |  |  | chr19 | 57802824 | 57802824 | G | A | het | . | . | . | 176 | 0 | 0% | G | 138 | 4 | 2.82% | R | Somatic | 0.0388299 |
| intronic | HSP90AB1 |  |  |  |  |  |  |  |  |  |  |  |  |  | chr6 | 44219690 | 44219690 | C | T | het | . | . | . | 206 | 0 | 0% | C | 138 | 4 | 2.82% | Y | Somatic | 0.02703 |
| intronic | PARD3 |  |  |  |  |  |  |  |  |  |  |  |  |  | chr10 | 34985354 | 34985354 | G | A | het | . | . | . | 181 | 0 | 0% | G | 140 | 4 | 2.78% | R | Somatic | 0.037646 |
| exonic | C12orf66 | nonsynonymous SNV | NM_152440:c.C929G:p.A310G | 558;Name=lod=248 |  |  |  |  |  | 0.01 | 0.993 | 0.9998 | 0.999436 | 1 | chr12 | 64588031 | 64588031 | G | C | het | . | . | . | 184 | 0 | 0% | G | 142 | 4 | 2.74% | S | Somatic | 0.0374359 |
| exonic | SIN3A | nonsynonymous SNV | NM_001145357:c.G2758A:p.E920K | 731;Name=lod=1242 |  |  |  |  |  | 0.04 | 0.827 | 0.998891 | 0.999683 | 1 | chr15 | 75684676 | 75684676 | C | T | het | . | . | . | 180 | 0 | 0% | C | 142 | 4 | 2.74% | Y | Somatic | 0.0393161 |
| intronic | HGS |  |  |  |  |  |  |  |  |  |  |  |  |  | chr17 | 79658637 | 79658637 | G | A | het | . | . | . | 181 | 0 | 0% | G | 142 | 4 | 2.74% | R | Somatic | 0.0388352 |
| intronic | PKNOX1 |  |  |  |  |  |  |  |  |  |  |  |  |  | chr21 | 44430155 | 44430155 | C | T | het | . | . | . | 93 | 0 | 0% | C | 77 | 2 | 2.53% | Y | Somatic | 0.2095063 |
| exonic | ZNF256 | synonymous SNV | NM_005773:c.C1119T:p.H373H | 243;Name=lod=13 |  |  |  |  | rs145193105 |  |  |  |  |  | chr19 | 58453057 | 58453057 | G | A | het | . | . | . | 237 | 4 | 1.66% | G | 96 | 12 | 11.11% | R | Somatic | 2.66E-04 |
| exonic | SULF1 | synonymous SNV | NM_001128204:c.C567T:p.D189D | 707;Name=lod=1000 |  |  |  |  |  |  |  |  |  |  | chr8 | 70501209 | 70501209 | C | T | het | . | . | . | 167 | 0 | 0% | C | 157 | 4 | 2.48% | Y | Somatic | 0.0569479 |
| exonic | ZNF132 | synonymous SNV | NM_003433:c.T1479G:p.G493G | 300;Name=lod=22 |  |  |  |  |  |  |  |  |  |  | chr19 | 58945332 | 58945332 | A | C | het | . | . | . | 107 | 1 | 0.93% | A | 26 | 4 | 13.33% | M | Somatic | 0.0080035 |
| exonic | ZNF132 | nonsynonymous SNV | NM_003433:c.T1473A:p.D491E |  |  |  |  |  |  | 1 | 0.003 | 0.004236 | 3.26E-04 | 0.59621 | chr19 | 58945338 | 58945338 | A | T | het | . | . | . | 110 | 1 | 0.90% | A | 32 | 4 | 11.11% | W | Somatic | 0.0129499 |
| exonic | ZNF132 | synonymous SNV | NM_003433:c.A1464G:p.E488E |  |  |  |  |  |  |  |  |  |  |  | chr19 | 58945347 | 58945347 | T | C | het | . | . | . | 112 | 0 | 0% | T | 35 | 6 | 14.63% | Y | Somatic | 2.79E-04 |
| exonic | SNPH | nonsynonymous SNV | NM_014723:c.A1210T:p.M404L |  |  |  |  |  |  | 0.33 | 0.047 | 0.119666 | 1.30E-05 | 0.857431 | chr20 | 1286423 | 1286423 | A | T | het | . | . | . | 38 | 0 | 0% | A | 9 | 2 | 18.18% | W | Somatic | 0.0467687 |
| intronic | ZNF343 |  |  |  |  |  |  |  |  |  |  |  |  |  | chr20 | 2474151 | 2474151 | T | A | het | . | . | . | 103 | 2 | 1.90% | T | 45 | 6 | 11.76% | W | Somatic | 0.0153308 |
| intronic | MKKS |  |  | 317;Name=lod=26 |  | 0.21 | 0.21 | 0.21 | rs764266 |  |  |  |  |  | chr20 | 10389480 | 10389480 | T | A | het | . | . | . | 36 | 0 | 0% | T | 18 | 3 | 14.29% | W | Somatic | 0.0454545 |
| exonic | RALGAPA2 | synonymous SNV | NM_020343:c.C261T:p.F87F | 610;Name=lod=400 |  |  |  |  |  |  |  |  |  |  | chr20 | 20656889 | 20656889 | G | A | het | . | . | . | 79 | 1 | 1.25% | G | 23 | 3 | 11.54% | R | Somatic | 0.04488 |
| intronic | NINL |  |  |  |  |  |  |  |  |  |  |  |  |  | chr20 | 25471930 | 25471930 | T | G | het | . | . | . | 54 | 1 | 1.82% | T | 33 | 5 | 13.16% | K | Somatic | 0.0398395 |
| exonic | NCOA6 | nonsynonymous SNV | NM_001242539:c.A2803C:p.T935P | 483;Name=lod=122 |  |  |  |  |  | 0.18 | 0 | 0.916829 | 8.26E-04 | 0.957151 | chr20 | 33334722 | 33334722 | T | G | het | . | . | . | 49 | 0 | 0% | T | 12 | 2 | 14.29% | K | Somatic | 0.046595 |
| exonic | RBL1 | nonsynonymous SNV | NM_002895:c.G1961T:p.G654V | 629;Name=lod=482 |  |  |  |  |  | 0 | 0.933 | 0.999111 | 0.99988 | 1 | chr20 | 35663854 | 35663854 | C | A | het | . | . | . | 76 | 0 | 0% | C | 18 | 2 | 10% | M | Somatic | 0.0416667 |
| exonic | MATN4 | nonsynonymous SNV | NM_003833:c.G624T:p.Q208H | 489;Name=lod=129 |  |  |  |  |  | 0.03 | 0.003 | 0.9985 | 0.984288 | 0.992135 | chr20 | 43932887 | 43932887 | C | A | het | . | . | . | 193 | 0 | 0% | C | 163 | 4 | 2.40% | M | Somatic | 0.0454155 |
| intronic | HNF4A |  |  |  |  |  |  |  |  |  |  |  |  |  | chr20 | 43048528 | 43048528 | A | C | het | . | . | . | 62 | 1 | 1.59% | A | 74 | 9 | 10.84% | M | Somatic | 0.0261491 |
| exonic | LAMA5 | nonsynonymous SNV | NM_005560:c.T695G:p.V232G | 494;Name=lod=136 |  |  |  |  |  | 0 | 0.998 | 0.994618 | 0.998461 | 1 | chr20 | 60927128 | 60927128 | A | C | het | . | . | . | 69 | 0 | 0% | A | 44 | 6 | 12% | M | Somatic | 0.0045793 |
| intronic | UCKL1 |  |  |  |  |  |  |  |  |  |  |  |  |  | chr20 | 62571418 | 62571418 | G | A | het | . | . | . | 28 | 0 | 0% | G | 28 | 5 | 15.15% | R | Somatic | 0.0398941 |
| UTR3 | ZNF512B |  |  |  |  |  |  |  |  |  |  |  |  |  | chr20 | 62591221 | 62591221 | C | G | het | . | . | . | 61 | 1 | 1.61% | C | 34 | 6 | 15% | S | Somatic | 0.0138963 |
| intronic | GRIK1 |  |  |  |  |  |  |  |  |  |  |  |  |  | chr21 | 30910220 | 30910220 | A | G | het | . | . | . | 46 | 0 | 0% | A | 8 | 2 | 20% | R | Somatic | 0.0292208 |
| exonic | ITSN1 | stopgain SNV | NM_001001132:c.C1747T:p.R583X | 525;Name=lod=182 |  |  |  |  |  | 0.75 | 0.637848 | 0.981846 | 0.680489 | 0.999908 | chr21 | 35154360 | 35154360 | C | T | het | . | . | . | 164 | 0 | 0% | C | 136 | 3 | 2.16% | Y | Somatic | 0.095411 |
| exonic | IL17RA | nonsynonymous SNV | NM_014339:c.A2310C:p.R770S |  |  |  |  |  |  | 0.01 | 0.991 | 0.010279 | 0.263706 | 0.232909 | chr22 | 17590419 | 17590419 | A | C | het | . | . | . | 25 | 0 | 0% | A | 19 | 4 | 17.39% | M | Somatic | 0.0455083 |
| ncRNA_intronic | BCRP3,POM121L10P |  |  |  | 0.98 |  |  |  |  |  |  |  |  |  | chr22 | 25044113 | 25044113 | T | G | het | . | . | . | 53 | 1 | 1.85% | T | 78 | 15 | 16.13% | K | Somatic | 0.0047195 |
| intronic | SEC14L3 |  |  |  |  |  |  |  |  |  |  |  |  |  | chr22 | 30867850 | 30867850 | T | C | het | . | . | . | 58 | 0 | 0% | T | 98 | 11 | 10.09% | Y | Somatic | 0.0076103 |
| intronic | LIMK2 |  |  |  |  |  |  |  |  | 0.03 |  |  |  |  | chr22 | 31654428 | 31654428 | C | T | het | . | . | . | 48 | 0 | 0% | C | 12 | 2 | 14.29% | Y | Somatic | 0.0481227 |
| intronic | BPIFC |  |  |  |  |  |  |  |  |  |  |  |  |  | chr22 | 32843356 | 32843356 | C | T | het | . | . | . | 61 | 1 | 1.61% | C | 25 | 5 | 16.67% | Y | Somatic | 0.0132233 |
| exonic | MCM5 | nonsynonymous SNV | NM_006739:c.A496C:p.T166P | 591;Name=lod=335 |  |  |  |  |  | 0 | 0.958 | 0.996627 | 0.999996 | 1 | chr22 | 35802618 | 35802618 | A | C | het | . | . | . | 48 | 0 | 0% | A | 58 | 9 | 13.43% | M | Somatic | 0.0060803 |
| intronic | CACNG2 |  |  |  |  |  |  |  |  |  |  |  |  |  | chr22 | 37098383 | 37098383 | A | G | het | . | . | . | 70 | 1 | 1.41% | A | 41 | 5 | 10.87% | R | Somatic | 0.0341126 |
| exonic | MKL1 | nonsynonymous SNV | NM_020831:c.C1250G:p.T417R | 321;Name=lod=27 |  |  |  |  |  | 0 | 0.147 | 0.999396 | 0.969096 | 0.975517 | chr22 | 40815192 | 40815192 | G | C | het | . | . | . | 41 | 0 | 0% | G | 10 | 2 | 16.67% | S | Somatic | 0.0478955 |
| exonic | CCDC134 | nonsynonymous SNV | NM_024821:c.A134G:p.E45G | 561;Name=lod=255 |  |  |  |  |  | 0.01 | 0.735 | 0.980682 | 0.893768 | 0.999997 | chr22 | 42205913 | 42205913 | A | G | het | . | . | . | 29 | 0 | 0% | A | 69 | 10 | 12.66% | R | Somatic | 0.037205 |
| exonic | RRP7A | nonsynonymous SNV | NM_015703:c.G628A:p.G210S | 549;Name=lod=228 | 0.97 |  |  |  | rs3752506 | 0.48 | 0.014 | 0.898849 | 0.998614 | 0.987614 | chr22 | 42910241 | 42910241 | C | T | het | . | . | . | 51 | 0 | 0% | C | 42 | 6 | 12.50% | Y | Somatic | 0.0109515 |
| exonic | SELO | nonsynonymous SNV | NM_031454:c.T1629G:p.S543R |  |  |  |  |  |  | 0.01 | 0.799 | 0.183641 | 0.996753 | 0.996059 | chr22 | 50655246 | 50655246 | T | G | het | . | . | . | 38 | 0 | 0% | T | 29 | 4 | 12.12% | K | Somatic | 0.0421146 |
| intronic | PLXNB2 |  |  |  |  |  |  |  |  |  |  |  |  |  | chr22 | 50719151 | 50719151 | G | C | het | . | . | . | 21 | 0 | 0% | G | 10 | 4 | 28.57% | S | Somatic | 0.0191176 |
| exonic | VTCN1 | nonsynonymous SNV | NM_024626:c.G58T:p.A20S | 532;Name=lod=194 |  |  |  |  |  | 0 | 0.036 | 0.998511 | 0.018706 | 0.999965 | chr1 | 117712768 | 117712768 | C | A | het | . | . | . | 201 | 0 | 0% | C | 143 | 2 | 1.38% | M | Somatic | 0.1749183 |
| exonic | ACR | nonsynonymous SNV | NM_001097:c.T791G:p.V264G |  | 0.98 |  |  |  |  | 0.3 | 0.996 | 0.942856 | 0.463668 | 0.831165 | chr22 | 51183160 | 51183160 | T | G | het | . | . | . | 33 | 0 | 0% | T | 55 | 7 | 11.29% | K | Somatic | 0.0445061 |
| exonic | PLCXD1 | nonsynonymous SNV | NM_018390:c.A425G:p.E142G | 325;Name=lod=28 | 1 |  |  |  |  | 0.01 |  |  |  |  | chrX | 208197 | 208197 | A | G | het | . | . | . | 46 | 0 | 0% | A | 70 | 8 | 10.26% | R | Somatic | 0.0212979 |
| exonic | CSF2RA | nonsynonymous SNV | NM_172249:c.T670C:p.S224P |  | 1 |  |  |  |  | 0.05 |  |  |  |  | chrX | 1424362 | 1424362 | T | C | het | . | . | . | 29 | 0 | 0% | T | 89 | 12 | 11.88% | Y | Somatic | 0.0413623 |
| exonic | ARHGAP6 | synonymous SNV | NM_006125:c.T591G:p.G197G | 527;Name=lod=184 |  |  |  |  |  |  |  |  |  |  | chrX | 11272825 | 11272825 | A | C | het | . | . | . | 24 | 0 | 0% | A | 36 | 7 | 16.28% | M | Somatic | 0.0370542 |
| exonic | MAGEB18 | synonymous SNV | NM_173699:c.A171C:p.T57T |  |  |  |  |  |  |  |  |  |  |  | chrX | 26157273 | 26157273 | A | C | het | . | . | . | 42 | 0 | 0% | A | 49 | 6 | 10.91% | M | Somatic | 0.0293367 |
| intronic | DMD |  |  |  |  |  |  |  |  |  |  |  |  |  | chrX | 32632599 | 32632599 | C | A | het | . | . | . | 55 | 0 | 0% | C | 13 | 2 | 13.33% | M | Somatic | 0.0434783 |
| exonic | ZNF674 | nonsynonymous SNV | NM_001039891:c.G1583A:p.S528N |  |  |  |  |  |  | 0.38 |  |  |  |  | chrX | 46359441 | 46359441 | C | T | het | . | . | . | 111 | 2 | 1.77% | C | 45 | 6 | 11.76% | Y | Somatic | 0.0117006 |
| exonic | ZNF674 | synonymous SNV | NM_001039891:c.C1575T:p.Y525Y |  |  |  |  |  |  |  |  |  |  |  | chrX | 46359449 | 46359449 | G | A | het | . | . | . | 112 | 2 | 1.75% | G | 45 | 6 | 11.76% | R | Somatic | 0.0113208 |
| exonic | ZNF674 | synonymous SNV | NM_001039891:c.T1572C:p.P524P |  |  |  |  |  |  |  |  |  |  |  | chrX | 46359452 | 46359452 | A | G | het | . | . | . | 117 | 2 | 1.68% | A | 45 | 5 | 10% | R | Somatic | 0.0244864 |
| intronic | PHF16 |  |  |  |  |  |  |  |  |  |  |  |  |  | chrX | 46915444 | 46915444 | C | A | het | . | . | . | 62 | 0 | 0% | C | 27 | 3 | 10% | M | Somatic | 0.03233 |
| exonic | ZNF157 | synonymous SNV | NM_003446:c.A1065G:p.E355E | 425;Name=lod=71 |  |  |  |  |  |  |  |  |  |  | chrX | 47272537 | 47272537 | A | G | het | . | . | . | 58 | 0 | 0% | A | 24 | 3 | 11.11% | R | Somatic | 0.0296143 |
| exonic | ZNF157 | synonymous SNV | NM_003446:c.G1068A:p.K356K | 425;Name=lod=71 |  |  |  |  |  |  |  |  |  |  | chrX | 47272540 | 47272540 | G | A | het | . | . | . | 58 | 1 | 1.69% | G | 24 | 4 | 14.29% | R | Somatic | 0.0353535 |
| exonic | ZNF157 | synonymous SNV | NM_003446:c.T1074C:p.Y358Y |  |  |  |  |  |  |  |  |  |  |  | chrX | 47272546 | 47272546 | T | C | het | . | . | . | 59 | 0 | 0% | T | 22 | 3 | 12% | Y | Somatic | 0.0241384 |
| exonic | ZNF157 | nonsynonymous SNV | NM_003446:c.C1075G:p.Q359E |  |  |  |  |  |  | 1 | 0.004 | 0.828616 | 0.001294 | 0.822088 | chrX | 47272547 | 47272547 | C | G | het | . | . | . | 59 | 1 | 1.67% | C | 21 | 4 | 16% | S | Somatic | 0.0247589 |
| exonic | ZNF157 | synonymous SNV | NM_003446:c.A1086G:p.E362E |  |  |  |  |  |  |  |  |  |  |  | chrX | 47272558 | 47272558 | A | G | het | . | . | . | 67 | 0 | 0% | A | 26 | 3 | 10.34% | R | Somatic | 0.0255739 |
| intronic | DGKK |  |  |  |  |  |  |  |  |  |  |  |  |  | chrX | 50165431 | 50165431 | C | T | het | . | . | . | 84 | 0 | 0% | C | 27 | 3 | 10% | Y | Somatic | 0.016884 |
| intronic | FAM120C |  |  |  |  |  |  |  |  |  |  |  |  |  | chrX | 54186063 | 54186063 | A | G | het | . | . | . | 50 | 0 | 0% | A | 34 | 4 | 10.53% | R | Somatic | 0.0316546 |
| exonic | POU3F4 | nonsynonymous SNV | NM_000307:c.A329C:p.H110P | 589;Name=lod=330 |  |  |  |  |  | 0.05 | 0.228 | 0.99823 | 0.980569 | 0.999688 | chrX | 82763661 | 82763661 | A | C | het | . | . | . | 43 | 0 | 0% | A | 49 | 6 | 10.91% | M | Somatic | 0.0275405 |
| exonic | RPS6KA6 | nonsynonymous SNV | NM_014496:c.C1624A:p.R542S | 701;Name=lod=942 |  |  |  |  |  | 0 | 1 | 0.987324 | 0.999998 | 1 | chrX | 83357197 | 83357197 | G | T | het | . | . | . | 76 | 0 | 0% | G | 17 | 2 | 10.53% | K | Somatic | 0.0382979 |
| intronic | DIAPH2 |  |  |  |  |  |  |  |  |  |  |  |  |  | chrX | 96638889 | 96638889 | A | C | het | . | . | . | 92 | 1 | 1.08% | A | 43 | 6 | 12.24% | M | Somatic | 0.0069748 |
| UTR3 | 6-Sep |  |  |  |  |  |  |  |  |  |  |  |  |  | chrX | 118750554 | 118750554 | A | T | het | . | . | . | 33 | 0 | 0% | A | 19 | 5 | 20.83% | W | Somatic | 0.0101512 |
| exonic | SOX3 | synonymous SNV | NM_005634:c.A84C:p.L28L | 343;Name=lod=33 |  |  |  |  |  |  |  |  |  |  | chrX | 139587142 | 139587142 | T | G | het | . | . | . | 47 | 0 | 0% | T | 45 | 5 | 10% | K | Somatic | 0.0328765 |
| exonic | PLXNB3 | nonsynonymous SNV | NM_005393:c.T452G:p.V151G |  |  |  |  |  |  | 0.02 | 0.8 | 0.995057 | 0.553822 | 0.797002 | chrX | 153032734 | 153032734 | T | G | het | . | . | . | 18 | 0 | 0% | T | 16 | 5 | 23.81% | K | Somatic | 0.035343 |
|  |  |  |  |  |  |  |  |  |  |  |  |  |  |  |  |  |  |  |  |  |  |  |  |  |  |  |  |  |  |  |  |  |  |

| **Table S8: The somatic SNV of skin tumor tissue.** | | | | | | | | |  |  |  |  |  |  |  |  |  |  |  |  |  |  |  |  |  |  |  |  |  |  |  |  |  |
| --- | --- | --- | --- | --- | --- | --- | --- | --- | --- | --- | --- | --- | --- | --- | --- | --- | --- | --- | --- | --- | --- | --- | --- | --- | --- | --- | --- | --- | --- | --- | --- | --- | --- |
| **Func** | **Gene** | **ExonicFunc** | **AAChange** | **Conserved** | **SegDup** | **1000G_ALL** | **1000G_ALL** | **1000G_ALL** | **dbSNP137** | **SIFT** | **PolyPhen2** | **LJB_PhyloP** | **LJB_MutationTaster** | **LJB_LRT** | **Chr** | **Start** | **End** | **Ref** | **Obs** | **Genotype** | **Cosmic** |  |  | **normal_reads1** | **normal_reads2** | **normal_var_freq** | **normal_gt** | **tumor_reads1** | **tumor_reads2** | **tumor_var_freq** | **tumor_gt** | **somatic_status** | **somatic_p_value** |
| exonic | GABRD | nonsynonymous SNV | NM_000815:c.G1241A:p.G414D | 235;Name=lod=12 |  |  |  |  |  | 0.41 | 0.472 | 0.998314 | 0.948997 | 0.024083 | chr1 | 1961603 | 1961603 | G | A | het | . | . | . | 317 | 0 | 0% | G | 405 | 5 | 1.22% | R | Somatic | 0.0564419 |
[truncated: 629,576 more chars]
